# STARD: How many lymph nodals needed to be dissected in corpus carcinoma?

**DOI:** 10.1097/MD.0000000000010260

**Published:** 2018-04-20

**Authors:** Shuping Zhao, Dehua Ma, Yu Huang, Lei Zhang, Yuan Cao, Yawen Wang

**Affiliations:** a Qingdao Women and Children Binomial Model from the SEER Database Strict; b The Affiliated Hospital of Qingdao University, Qingdao, Shandong Province, China.

**Keywords:** beta-binominal model, corpus carcinoma, lymph nodes examination, quantification, SEER database

## Abstract

During corpus carcinoma surgery, there is uncertainty as to how many lymph nodes should be dissected and examined to determine lymph invasion.

In this study, we evaluated a beta-binominal model in data extracted from the Surveillance, Epidemiology, and End Results (SEER) database, which contains 22,372 complete records. We quantified the relationship between examined node number and the probability of missing invaded nodes. Survival curves were used for further validation.

We found that for stage T1-T4, 1, 10, 23, and 37 lymph nodes, respectively, needed to be examined to minimize the missing positive nodal probability (1-nodal staging score, NSS) to less than 5%. A hypothetical lymph node examination rate was calculated. Survival rate of T2 and T3 stage samples was significantly associated with NSS, but T1 and T4 sample survival rate was not.

The currently dissected nodal should be reduced to 1 to 2 for T1, remains to 10 for T2, and increases to 23 for T3.

## Abstract 2

Supplemental Digital Content is available in the text

## 1 Introduction

Uterine cancer is among the most prevalent cancers in women. According to a recent study, 63,400 new uterine cancer cases and 21,800 related deaths were reported in China in 2015.^[1]^ Corpus carcinoma is one of the most important subtypes of uterine cancer. Radiation is currently recommended for advanced corpus carcinoma with lymph invasion.^[2,3]^ Patients receiving radiation have a significantly reduced recurrence and metastasis rate, and disease-free survival is greater.^[4,5]^ However, side effects of radiation have been widely reported, including impaired fertility,^[6]^ secondary malignancy,^[7,8]^ and lung metastasis.^[9]^ Thus, accurate diagnosis of lymph invasion is critical to guide adjuvant therapy.

On the one hand, apparently, adequate lymph node dissection/examination is necessary for staging, as the invaded lymph nodes have less chance to be missed. On the other hand, node dissection reduces immune activity of the affected region, because the well-known role of lymph nodes in immune system. Thus, a balancing of diagnostic accuracy and life quality is critical. A retrospective study of 12,333 patients found that extensive lymph node dissection significantly improved the survival of intermediate and high-risk patients.^[10]^ A multicenter retrospective study revealed that extended lymph nodes dissection did not significantly enhanced the survival of ductal adenocarcinoma of the head of the pancreas.^[11]^ However, the number of nodes to examine the probability of missing invasive positive nodes at various stages has not yet been reported.

In this study, we used a beta-binomial model to study the relationships between the possibility of missing positive lymph nodes at various primary tumor stages, using lymph examination information from the Surveillance, Epidemiology, and End Results (SEER) database (N = 22,372). We found that the minimum number of nodes examined for T1-T4 were 1, 10, 23, and 37, respectively. The currently dissected nodal should be reduced to 1 to 2 for T1, remains to 10 for T2, and increases to 23 for T3, while diagnosis-oriented lymph nodes dissection is not recommended for T4.

## 2 Materials and methods

### 2.1 Data collection

The SEER database covers 26% of the population in the United States (https://seer.cancer.gov/). In this study, corpus carcinoma patients identified as primary cancer and malignant cancer were chosen for further analysis (no metastasis, secondary, or benign site). Patients without complete records of primary tumor stage (T staging in TNM stage), and regional node examination, or positive nodes were excluded. The T1a-c, T2a-c, T3a-c, and T4a-c stages were combined as T1, T2, T3, and T4 stages. In all, 22,372 patients were enrolled in this study (Table S2). The ethnic approval is not needed for this study because none of the authors participated in the raw data collection. The ethnic approval is given by the SEER database.

### 2.2 Model assumptions

A beta-binomial distribution model was used to evaluate the possibility of missing the invasion-positive lymph nodes, using total lymph nodes examined and the number of positive nodes. In this study, true positive (TP) means that the lymph node was truly invaded with cancer cells. True negative (TN) means status was uninvaded. False-negative (FN) samples were those with invaded lymph nodes, with none of the invaded lymph nodes having been examined.

Three hypotheses were employed in this model:

1) All node examinations were correct.
2) The distribution of lymph nodes was exchangeable (independent and identically distributed). That means any examined lymph nodes have the same chance to be invaded, which enables us to calculate the invasion possibility.
3) The sensitivity of TP and FN was the same, which enables us to generalize the results to pathologically node-negative samples. Sensitivities only can be calculated in node-positive samples.

### 2.3 Model development and coefficient evaluation

1) The proportion of the number of positive lymph nodes (non-N0 stage) and the number of total nodes dissected/examined was used to estimate the coefficients of beta-binomial distribution (α and β). In this step, samples used were limited to samples with at least 1 lymph node examined.

2) False-negative rates were estimated according to the model and coefficient estimated, in the overall datasets and subdatasets (primary tumor stage, T1-T4), and the observed and corrected prevalence was calculated as follows: 
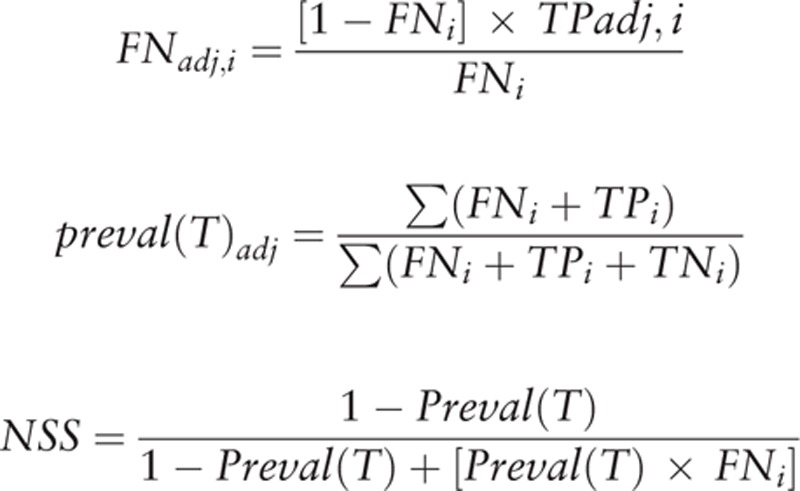


where FN_adj,k_ indicates adjusted FN rates; FN is observed FN rate; TP_adj,k_ is the TP rate; T indicates the primary tumor stage.

3) Considering overall survival information is independent from lymph node dissection and nodal staging score, we used it for model validation. Tumors in various T stages were divided into quartiles using a nodal staging score, which represents that an individual is correctively diagnosed as lymph invade negative. Survival differences in the 4 subgroups were calculated using the log-rank test.

### 2.4 Statistical analysis

Statistical analyses were carried out with R packages. VGAM (v1.0–3) and bbmle (v1.0.18) were employed to estimate parameters α and β in the beta-binomial model. Survival differences among samples in quantiles were estimated with R package “survival” (Kaplan–Meier method).

## 3 Results

### 3.1 Data profile

After removing incomplete records from the SEER database, we enrolled 22,372 subjects. Detailed data regarding primary tumor stage (T-staging), age (stratified at age 60), nodal invasion rate, and number of nodes examined are displayed in Table 1. More than 90% patients were diagnosed as primary stage T1 and T2. Sample numbers in T4 stage were limited (N = 212, less than 1%). The lymph invasion rate rapidly increased with primary tumor stage. The median number of examined nodes in T1-T4 ranged from 8 to 11, and the mean number of examined nodes ranged from 10.08 to 14.14.

**Table 1:**
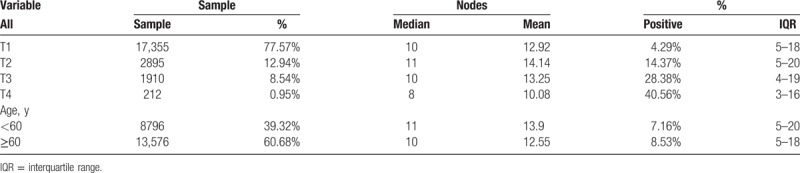
Nodal examination information in SEER database.

### 3.2 Missing invaded lymph node rate in overall data

**Figure 1:**
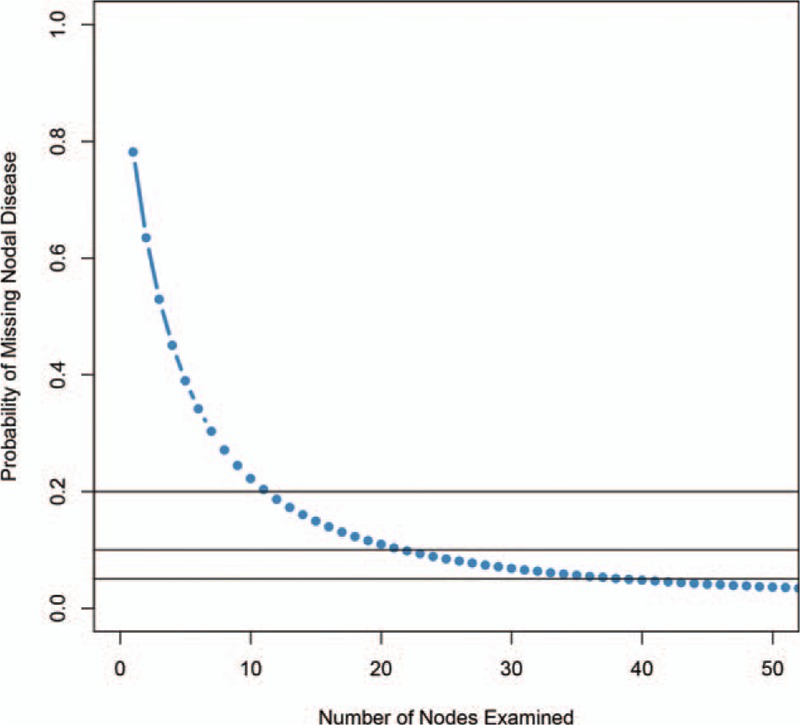
Missing node probability and examined nodes number in the overall data.

Two parameters, the beta-binominal model, α and β, were estimated to be 1.4131 [95% confidence interval (95% CI) 1.2823–1.5617] and 5.0827 (95% CI 4.4931–5.7645), respectively. The overall probability of missing nodal invasion was evaluated (Fig. 1). The probability of missing positive lymph nodes decreased with increasing number of examined nodes. When only 1 lymph node was dissected/examined, the probability of missing a positive node was 78.24%. At least 12 lymph nodes needed to be examined to minimize the probability of missing positive nodes to less than 20%, and at least 22 nodes needed to be examined to reduce the probability to 10%, and 39 nodes needed to be examined to reduce the probability to 5% (Table S1). According to the dataset, the median examined node number was 11, and the corresponding missing positive nodal probability was 20.36%, suggesting that the current node examination number is inadequate for the overall dataset.

### 3.3 False negative diagnostic rate in T1-T4

**Figure 2:**
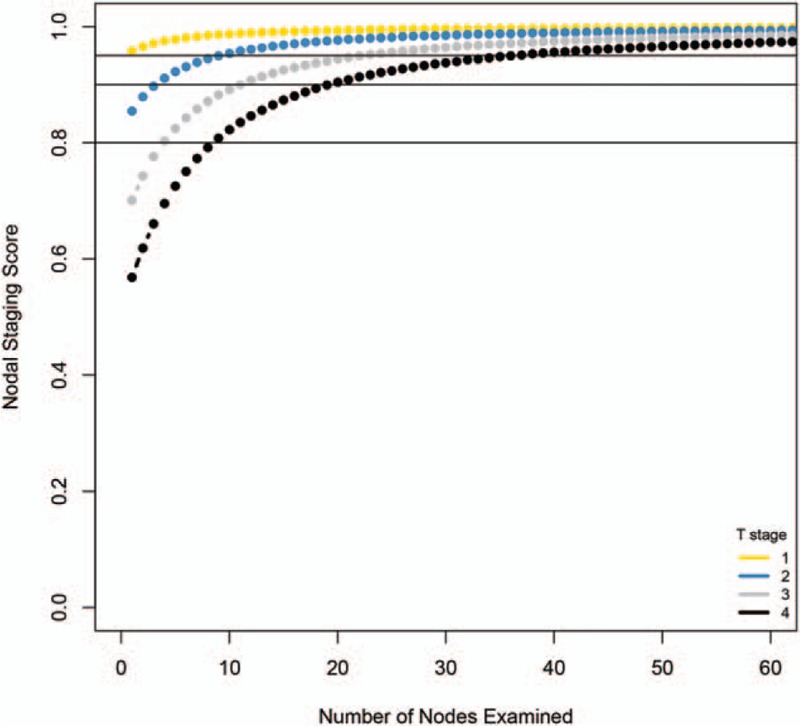
Nodal staging score and examined node number in various tumor stages.

Primary tumor stage was an important indicator for node examination (Fig. 2). The missing node probability was estimated for various primary tumor stages (T1-T4, Table S1). When 1 lymph node was examined, the probability of missing was 4.23%, 14.50%, 29.86%, and 43.14% for T1-T4, respectively. To minimize the probability of missing nodes to below 5%, the least numbers of examined nodes were 1, 10, 23, and 37, for T1, T2, T3, and T4, respectively. When the number of examined node numbers were 10, 11, 10, and 8 (the current median value of examined nodes for T1-T4), the probabilities of missing positive nodes were 1.24%, 4.23%, 10.81%, and 20.84% for T1-T4, respectively.

Combining current node positivity rates in various primary tumor stages and theoretical probabilities, we calculated corrected lymph invasion rates (Table 2). The corrected node-positive rates were 1.16%, 3.58%, 7.33%, and 9.13% higher than the observed rates. This suggests that the current implemented node examination number is adequate for T1-T2, but inadequate for T3.

**Table 2:**
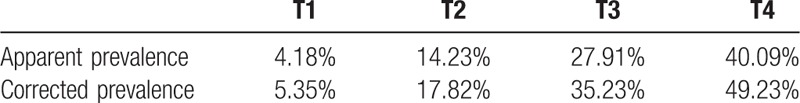
Observed and corrected nodal invasion rates in primary tumor stages.

### 3.4 Nodal staging score and survival

**Figure 3:**
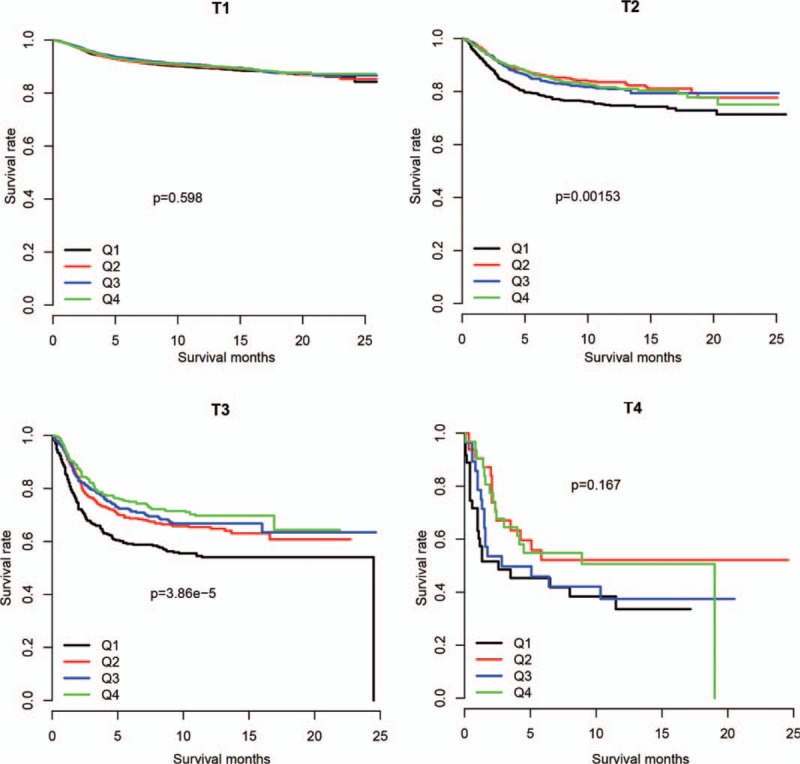
Survival of corpus carcinoma patients within quartiles (Q1-Q4) in various primary tumor stages. Q1 represents the lowest quartile NSS score and Q4 represents the highest.

Follow-up information was not used for model development and is independent from node staging score. Thus, we used it for validation. The node staging score in N0 stage primary tumor was divided by quantiles, and survival difference was compared (Fig. 3). The nodal staging score was significantly associated with survival in the T2N0 and T3N0 groups, but was not significant in the T1N0 or T4N0 stages, consistent with our previous result.

## 4 Discussion

Lymph nodes invasion is an important process for cancer metastasis,^[12]^ both biologically and clinically. Thus, lymph node invasion is strongly associated with relapse and decreased overall survival in corpus carcinoma.^[13,14]^ Hence, lymph node invasion is crucial for therapeutic decision-making.^[15]^ Adequate nodal dissection and examination significantly improves survival in corpus carcinoma.^[10]^ On the contrary, excessive lymph nodes dissection burdened the surgeon, weaken the immune system, and reduce the life quality. Thus, it is critical to quantify the number of lymph nodes to be dissected. Even though works for other cancers in quantification were reported, corpus carcinoma was not reported yet.

We implemented a beta-binominal model to evaluate data from the SEER database, including 22,372 patients. We showed that the probabilities of missing nodes were 1.24%, 4.23%, 10.81%, and 20.84% for T1-T4, respectively, using current median examined nodes as a reference. To reach 95% accuracy, at least 1, 10, 23, and 37 nodes need to be examined in T1-T4, respectively, suggesting that the currently node examination number is excessive for T1, adequate for T2, and insufficient for T3-T4. The survival information also supports this result. As lymph node dissection is excessive for T1, the NSS does not contribute to survival; the lymph examination is moderate for T2-T3, so NSS contributes survival. According to the result, we suggest that fewer lymph nodes be examined (1–2 suggested), and 10 and 23 lymph nodes should be examined for T1-T3 patients. Lymph node examination is not recommended for T4 patients because it is nearly impossible to reduce the probability of missing nodes to less than 5%.

In our beta-binominal model, we employed the following hypotheses: First, each lymph node examined has the same chance of invasion. This hypothesis agreed with previous studies in other cancers.^[16–19]^ The second assumption was that all node examinations were correct. This is a reasonable hypothesis because the SEER database was constructed by an expert pathologist.

There are several limitations to this study. It was retrospective, though it included several centers. Important clinical variables, such as drug usage and time to metastasis/recurrence, were not available. This may introduce bias as a result of absent enrollment controls. In addition, the T4 sample size was small. Finally, the coefficients solved according to the SEER database require further validation. This is a hypothesis-driven model, and not a machine learning model.

## Author contributions

**Conceptualization:** Shuping Zhao, Dehua Ma, Yuan Cao.

**Data curation:** Shuping Zhao, Yu Huang, Lei Zhang.

**Formal analysis:** Shuping Zhao, Yawen Wang.

**Funding acquisition:** Lei Zhang.

**Investigation:** Shuping Zhao.

**Methodology:** Yuan Cao.

**Software:** Lei Zhang, Yawen Wang.

**Writing – original draft:** Shuping Zhao, Dehua Ma, Yu Huang, Lei Zhang.

**Writing – review & editing:** Shuping Zhao, Dehua Ma, Lei Zhang, Yuan Cao, Yawen Wang.

## Supplementary Material

Supplemental Digital Content
