# STARD: How many lymph nodals needed to be dissected in corpus carcinoma?: Erratum

**DOI:** 10.1097/MD.0000000000010769

**Published:** 2018-05-11

In the article, “STARD: How many lymph nodals needed to be dissected in corpus carcinoma?”,^[1]^ which appeared in Volume 97, Issue 16 of *Medicine*, the author names and affiliation orders should be:

Dehua MA, MD^a^, Shuping Zhao, MD^b^, Yu Huang, MD^b^, Lei Zhang, MD^b^, Yuan Cao, MD^b^, Yawen Wang, MD^b^

^a^The Affiliated Hospital of Qingdao University, Qingdao, Shandong Province, China, 266000.

^b^Qingdao Women and Children Binomial Model From the SEER Database Strict, Qingdao, Shandong Province, China, 266000.

## References

[1] Zhao S Ma D Huang Y STARD: How many lymph nodals needed to be dissected in corpus carcinoma? . 2018 97:e10260. 10.1097/MD.0000000000010260 PMC5916645 29668578

