# Supplementary material for: STARD: How many lymph nodals needed to be dissected in corpus carcinoma?
Source: Medicine (Baltimore). 2018 Apr 20;97(16):e0260. doi: 10.1097/MD.0000000000010260 (PMC5916645; doi:10.1097/MD.0000000000010260)
Supplement: Supplemental Digital Content [file medi-97-e0260-s001.doc]

| Nodes | NSS_all | NSS_T1 | NSS_T2 | NSS_T3 | NSS_T4 |
| --- | --- | --- | --- | --- | --- |
| 1 | 0.21754 | 0.957675 | 0.85498 | 0.701427 | 0.568609 |
| 2 | 0.365048 | 0.965378 | 0.879012 | 0.743262 | 0.618945 |
| 3 | 0.470659 | 0.970969 | 0.897064 | 0.776418 | 0.660829 |
| 4 | 0.549432 | 0.975182 | 0.911019 | 0.80314 | 0.695956 |
| 5 | 0.610094 | 0.978452 | 0.922065 | 0.825006 | 0.72566 |
| 6 | 0.658022 | 0.98105 | 0.930984 | 0.843142 | 0.750986 |
| 7 | 0.696695 | 0.983157 | 0.938307 | 0.858369 | 0.772746 |
| 8 | 0.728453 | 0.984894 | 0.944408 | 0.87129 | 0.791582 |
| 9 | 0.754925 | 0.986347 | 0.949554 | 0.882361 | 0.807998 |
| 10 | 0.777274 | 0.987576 | 0.953942 | 0.891929 | 0.822398 |
| 11 | 0.796353 | 0.988628 | 0.957721 | 0.900264 | 0.835103 |
| 12 | 0.812801 | 0.989537 | 0.961003 | 0.907575 | 0.846375 |
| 13 | 0.827104 | 0.990329 | 0.963874 | 0.914029 | 0.856427 |
| 14 | 0.839636 | 0.991024 | 0.966405 | 0.91976 | 0.865434 |
| 15 | 0.850692 | 0.991637 | 0.968649 | 0.924877 | 0.873538 |
| 16 | 0.860507 | 0.992183 | 0.970649 | 0.929467 | 0.880861 |
| 17 | 0.86927 | 0.99267 | 0.972442 | 0.933603 | 0.887503 |
| 18 | 0.877132 | 0.993108 | 0.974056 | 0.937347 | 0.893549 |
| 19 | 0.88422 | 0.993503 | 0.975516 | 0.940747 | 0.89907 |
| 20 | 0.890637 | 0.993861 | 0.976842 | 0.943846 | 0.904127 |
| 21 | 0.89647 | 0.994186 | 0.97805 | 0.946682 | 0.908774 |
| 22 | 0.901791 | 0.994483 | 0.979154 | 0.949283 | 0.913055 |
| 23 | 0.906661 | 0.994756 | 0.980168 | 0.951676 | 0.917009 |
| 24 | 0.911133 | 0.995006 | 0.9811 | 0.953885 | 0.920669 |
| 25 | 0.91525 | 0.995236 | 0.98196 | 0.955927 | 0.924066 |
| 26 | 0.919053 | 0.995449 | 0.982755 | 0.957821 | 0.927225 |
| 27 | 0.922573 | 0.995646 | 0.983493 | 0.959581 | 0.930169 |
| 28 | 0.925839 | 0.995829 | 0.984178 | 0.96122 | 0.932917 |
| 29 | 0.928877 | 0.995999 | 0.984816 | 0.96275 | 0.935488 |
| 30 | 0.931709 | 0.996158 | 0.985412 | 0.96418 | 0.937897 |
| 31 | 0.934353 | 0.996306 | 0.985969 | 0.965519 | 0.940157 |
| 32 | 0.936827 | 0.996445 | 0.986491 | 0.966775 | 0.942282 |
| 33 | 0.939146 | 0.996575 | 0.98698 | 0.967956 | 0.944283 |
| 34 | 0.941323 | 0.996697 | 0.98744 | 0.969067 | 0.946169 |
| 35 | 0.943371 | 0.996812 | 0.987873 | 0.970114 | 0.94795 |
| 36 | 0.945299 | 0.99692 | 0.988281 | 0.971102 | 0.949633 |
| 37 | 0.947118 | 0.997022 | 0.988666 | 0.972036 | 0.951226 |
| 38 | 0.948836 | 0.997118 | 0.989031 | 0.97292 | 0.952736 |
| 39 | 0.950461 | 0.99721 | 0.989375 | 0.973758 | 0.954168 |
| 40 | 0.952 | 0.997296 | 0.989702 | 0.974552 | 0.955528 |
| 41 | 0.953459 | 0.997378 | 0.990012 | 0.975306 | 0.956822 |
| 42 | 0.954843 | 0.997456 | 0.990306 | 0.976023 | 0.958052 |
| 43 | 0.956159 | 0.99753 | 0.990586 | 0.976706 | 0.959225 |
| 44 | 0.957411 | 0.9976 | 0.990852 | 0.977356 | 0.960343 |
| 45 | 0.958603 | 0.997667 | 0.991106 | 0.977975 | 0.96141 |
| 46 | 0.959739 | 0.997731 | 0.991348 | 0.978567 | 0.962429 |
| 47 | 0.960822 | 0.997792 | 0.991579 | 0.979132 | 0.963403 |
| 48 | 0.961857 | 0.99785 | 0.991799 | 0.979672 | 0.964336 |
| 49 | 0.962846 | 0.997906 | 0.99201 | 0.980188 | 0.965228 |
| 50 | 0.963792 | 0.997959 | 0.992212 | 0.980683 | 0.966084 |
| 51 | 0.964698 | 0.99801 | 0.992406 | 0.981157 | 0.966904 |
| 52 | 0.965566 | 0.998059 | 0.992591 | 0.981612 | 0.967691 |
| 53 | 0.966397 | 0.998106 | 0.992768 | 0.982048 | 0.968447 |
| 54 | 0.967196 | 0.998151 | 0.992939 | 0.982467 | 0.969173 |
| 55 | 0.967962 | 0.998194 | 0.993103 | 0.982869 | 0.969872 |
| 56 | 0.968698 | 0.998235 | 0.99326 | 0.983257 | 0.970543 |
| 57 | 0.969406 | 0.998275 | 0.993412 | 0.983629 | 0.97119 |
| 58 | 0.970087 | 0.998313 | 0.993557 | 0.983987 | 0.971813 |
| 59 | 0.970742 | 0.99835 | 0.993698 | 0.984333 | 0.972414 |
| 60 | 0.971373 | 0.998386 | 0.993833 | 0.984666 | 0.972993 |
| 61 | 0.971982 | 0.99842 | 0.993963 | 0.984987 | 0.973552 |
| 62 | 0.972568 | 0.998453 | 0.994089 | 0.985296 | 0.974091 |
| 63 | 0.973134 | 0.998485 | 0.99421 | 0.985595 | 0.974612 |
| 64 | 0.97368 | 0.998516 | 0.994327 | 0.985884 | 0.975115 |
| 65 | 0.974208 | 0.998545 | 0.99444 | 0.986163 | 0.975602 |
| 66 | 0.974718 | 0.998574 | 0.994549 | 0.986433 | 0.976073 |
| 67 | 0.975211 | 0.998602 | 0.994655 | 0.986694 | 0.976528 |
| 68 | 0.975687 | 0.998629 | 0.994757 | 0.986946 | 0.976969 |
| 69 | 0.976148 | 0.998655 | 0.994856 | 0.987191 | 0.977396 |
| 70 | 0.976595 | 0.99868 | 0.994952 | 0.987427 | 0.97781 |
| 71 | 0.977027 | 0.998704 | 0.995045 | 0.987657 | 0.978211 |
| 72 | 0.977446 | 0.998728 | 0.995135 | 0.987879 | 0.9786 |
| 73 | 0.977852 | 0.998751 | 0.995222 | 0.988095 | 0.978977 |
| 74 | 0.978246 | 0.998773 | 0.995306 | 0.988304 | 0.979343 |
| 75 | 0.978628 | 0.998794 | 0.995388 | 0.988507 | 0.979698 |

ID T_stage n_total n_positive

1708 T3 1 1

4282 T1 15 0

7651 T1 5 0

8242 T1 7 0

8621 T1 20 0

1028279 T1 7 0

1033909 T1 2 0

1038646 T1 21 0

1044908 T1 1 0

1764208 T1 3 0

1771147 T1 9 0

1781702 T4 2 0

1788830 T1 5 0

1789319 T1 4 0

1805887 T1 34 5

1810976 T1 7 0

1817223 T1 6 0

1827874 T2 20 0

1828478 T2 1 0

1829471 T1 5 0

1830089 T1 10 0

1830241 T1 9 0

1830800 T1 6 0

1831761 T1 1 0

1832136 T1 3 0

1834097 T1 46 0

1834674 T1 2 0

1835392 T1 19 0

1836995 T1 26 0

1837106 T1 30 0

1838500 T1 10 0

1838515 T1 20 0

1838729 T1 35 0

1838810 T2 13 0

1839456 T1 26 0

1841963 T1 13 0

1843192 T2 23 0

1844705 T1 45 0

1845095 T1 7 0

1845292 T3 12 0

1845500 T2 31 0

1845539 T1 25 0

1845851 T1 4 0

1846897 T1 3 0

1847329 T1 28 0

1847866 T1 4 0

1848572 T1 9 0

1848751 T3 3 0

1849283 T1 32 0

1849801 T1 15 0

1849924 T1 20 0

1850575 T1 29 0

1851163 T1 6 0

1851257 T1 23 0

1851680 T1 4 0

1851917 T1 30 0

1852717 T1 1 0

1853287 T1 5 0

1853440 T1 30 0

1854111 T1 9 0

1855588 T1 2 0

1856414 T1 3 0

1856627 T1 4 0

1858260 T1 8 0

1861446 T1 13 0

1861931 T1 36 0

1862073 T1 11 0

1862551 T2 5 1

1862986 T1 5 0

1863392 T1 2 0

1865571 T1 5 0

1865813 T1 29 0

1865821 T1 9 0

1865903 T1 34 0

1867364 T1 5 0

1867389 T1 5 0

1867737 T1 32 0

1868645 T1 26 0

1868686 T1 30 0

1869247 T1 1 0

1869408 T1 2 0

1869643 T1 17 0

1869703 T1 9 0

1871154 T2 34 1

1871757 T1 3 0

1872275 T1 12 0

1872473 T2 14 0

1872627 T1 22 0

1876210 T1 31 0

1876762 T1 6 0

1877531 T2 2 0

1877680 T1 7 0

1878280 T1 5 0

1879348 T1 6 0

1879374 T1 2 0

1879666 T1 15 5

1879791 T1 16 0

1880480 T1 19 0

1880610 T1 5 0

1882047 T1 24 0

1883416 T1 6 0

1883436 T1 42 0

1884340 T1 2 0

1885011 T1 3 0

1885274 T1 2 0

1885848 T1 1 0

1886696 T2 3 0

1886816 T1 2 0

1888696 T1 1 0

1889211 T1 25 0

1889387 T2 15 0

1889649 T1 16 0

1889794 T1 5 0

1890805 T1 7 0

1891355 T3 4 0

1891675 T1 10 0

1892405 T2 28 0

1892510 T1 3 0

1892621 T1 1 0

1893957 T1 6 0

1894576 T1 5 0

1895395 T3 5 0

1896840 T1 5 0

1896968 T1 1 0

1897729 T2 2 0

1898363 T1 2 0

1898471 T1 7 0

1899068 T1 10 0

1899219 T1 12 0

1899683 T3 2 0

1900214 T1 1 0

1900861 T1 28 0

1901024 T1 13 0

1901275 T1 18 0

1901401 T1 3 0

1901805 T1 27 2

1902016 T3 4 0

1902442 T1 7 0

1902564 T1 25 0

1902762 T1 3 0

1903704 T3 4 0

1904349 T1 11 0

1904794 T1 1 0

1905453 T2 2 0

1906738 T1 14 0

1906929 T1 12 0

1907257 T1 14 0

1907319 T3 23 0

1908383 T1 10 0

1910269 T1 5 0

1910571 T1 3 0

1910755 T1 28 0

1911837 T1 23 0

1911956 T1 27 0

1912372 T2 10 0

1912684 T1 3 0

1912763 T1 27 0

1914114 T1 4 0

1914276 T1 5 0

1914314 T1 10 0

1914454 T1 13 0

1916388 T1 34 0

1916916 T1 14 0

1917146 T2 16 2

1917699 T1 18 1

1917799 T2 20 0

1919972 T3 17 0

1920217 T1 10 0

2625404 T2 13 0

2625812 T1 55 0

2626403 T2 3 0

2626445 T1 1 0

2628031 T1 1 0

2628450 T2 25 5

2628528 T1 7 0

2628957 T1 36 0

2629217 T1 22 0

2629274 T1 7 0

2629611 T1 7 0

2630498 T2 6 0

2631924 T1 3 0

2632152 T1 53 0

2632884 T1 26 0

2633092 T1 15 0

2633245 T1 21 0

2633314 T1 33 2

2633399 T1 8 0

2633415 T1 2 0

2633420 T1 19 0

2634283 T1 11 0

2634378 T1 1 0

2634895 T1 3 0

2635921 T1 6 0

2635992 T2 16 0

2636778 T1 1 0

2637140 T1 1 0

2638230 T1 18 0

2638312 T1 2 0

2638384 T2 7 2

2638830 T3 17 0

2639147 T1 16 0

2639604 T1 4 0

2640184 T1 4 0

2640309 T1 35 0

2641497 T2 23 0

2642026 T2 52 0

2642624 T1 5 0

2643204 T1 14 0

2643707 T2 2 1

2643738 T1 11 0

2643954 T1 1 0

2643967 T1 5 0

2644304 T1 38 0

2644787 T1 24 0

2644872 T1 55 0

2644892 T1 33 0

2645382 T1 28 0

2646268 T1 17 0

2646690 T1 6 0

2647180 T1 7 0

2648226 T2 14 0

2648688 T1 10 0

2648852 T1 7 0

2649208 T1 1 0

2650419 T1 16 0

2652126 T1 37 0

2652149 T1 17 0

2652243 T1 1 0

2653270 T2 1 0

2654100 T1 16 0

2654522 T1 23 0

2655127 T1 5 0

2655429 T1 6 0

2656372 T2 15 0

2656588 T1 21 0

2656993 T1 24 0

2657241 T1 22 0

2657300 T1 15 0

2657991 T1 16 0

2658103 T1 13 0

2658130 T1 2 0

2658240 T1 6 0

2658442 T2 3 0

2658972 T1 3 0

2660017 T1 5 0

2660179 T1 8 0

2660520 T1 34 0

2661460 T1 9 0

2662918 T2 7 0

2662920 T2 2 0

2663020 T3 1 0

2663219 T1 50 0

2663564 T1 2 0

2663738 T1 8 0

2663774 T1 17 0

2664537 T1 54 0

2664543 T2 9 0

2665293 T1 2 0

2665373 T1 11 0

2666007 T2 4 0

2668885 T1 6 0

2670341 T1 14 0

2671192 T2 5 0

2671834 T1 23 0

2672466 T1 25 0

2672613 T1 5 0

2673426 T1 1 0

2675143 T1 19 0

2675290 T1 8 0

2675385 T1 51 0

2675427 T1 10 0

2675519 T1 12 0

2677311 T1 4 0

2677693 T1 20 0

2678480 T1 4 0

2678515 T1 6 0

2679355 T1 22 0

2679578 T2 31 0

2679955 T1 5 0

2680570 T2 40 0

2680734 T1 18 0

2680835 T1 13 0

2680850 T1 20 0

2681013 T1 27 0

2681020 T1 31 0

2681721 T2 19 0

2681950 T1 26 0

2681975 T1 19 0

2682035 T1 1 0

2682355 T1 12 0

2682480 T1 19 0

2682772 T2 22 0

2683026 T2 52 1

2683288 T1 6 0

2683422 T1 1 0

2683618 T1 24 0

2683741 T1 4 0

2683944 T1 12 0

2684329 T1 21 0

2684724 T1 1 0

2685653 T1 4 0

2685985 T1 21 0

2686134 T1 43 0

2686409 T1 6 0

2686546 T1 33 0

2686791 T1 14 0

2686959 T2 6 0

2687409 T1 3 0

2687804 T1 12 0

2688274 T1 43 0

2688536 T1 3 1

2688670 T1 24 0

2691065 T3 17 13

2691404 T1 9 0

2692079 T1 10 0

2692374 T1 31 0

2692444 T1 3 0

2692859 T1 15 0

2692953 T1 27 0

2694039 T1 4 0

2694237 T1 4 0

2694595 T1 9 0

2694927 T1 16 0

2694984 T1 22 0

2695477 T1 16 0

2695483 T1 20 0

2695597 T1 10 0

2695976 T1 16 0

2697774 T1 31 0

2698045 T1 17 0

2698821 T1 14 1

2699167 T1 12 0

2699356 T1 10 0

2700624 T1 2 0

2700721 T1 18 0

2701631 T1 4 0

2702553 T1 22 0

2703757 T1 15 0

2703973 T1 14 0

2704201 T1 1 0

2704537 T2 2 0

2704723 T1 11 2

2705154 T1 12 0

2705533 T1 5 0

2705643 T3 8 1

2706209 T3 10 1

2706477 T1 16 0

2706605 T1 9 0

2707810 T3 8 0

2707866 T1 4 0

2709078 T1 1 0

2709969 T1 8 0

2710001 T1 15 1

2710184 T1 19 0

2710453 T3 4 0

2711863 T2 16 0

2713219 T1 4 0

2713450 T1 13 0

2713842 T1 5 0

2714220 T1 4 0

2714646 T1 37 0

2714899 T1 16 0

2715029 T1 3 0

2715234 T1 37 0

2715823 T1 14 0

2716204 T1 14 0

2716509 T1 11 0

2717527 T1 10 0

2717741 T1 11 0

2719013 T1 3 2

2719108 T1 20 0

2720966 T1 2 0

2721562 T1 31 0

2721752 T1 25 4

2721788 T1 3 0

2721939 T1 13 0

2722193 T1 22 0

2722326 T1 2 0

2722385 T1 6 0

2722491 T1 21 0

2724001 T1 5 1

2724010 T2 24 0

2724133 T1 6 0

2725131 T1 5 0

2726522 T1 4 0

2726757 T1 2 0

2726780 T1 5 0

2726838 T1 5 0

2728446 T1 41 0

2728833 T1 4 0

2728988 T1 1 0

2729087 T2 13 0

2730599 T1 20 0

2730718 T2 3 0

2731507 T1 12 0

2731737 T3 6 1

2731766 T2 4 0

2731938 T1 9 0

2732055 T1 13 0

2732191 T1 4 0

2732204 T1 1 0

2732631 T1 8 0

2732772 T1 20 0

2733831 T1 28 0

2734355 T1 7 0

2734957 T3 13 0

2735197 T1 14 0

2735353 T1 2 0

2735565 T2 11 0

2735806 T1 15 0

2736860 T1 1 0

2736891 T1 5 0

2737656 T1 30 0

2737797 T2 4 0

2738052 T1 37 0

2738123 T1 10 0

2738167 T1 14 0

2738560 T2 14 0

2738997 T1 3 0

2739099 T1 31 0

2739258 T3 1 0

2739589 T1 3 0

2740184 T1 34 0

2740916 T4 2 0

2741179 T1 10 0

2741454 T2 4 0

2741553 T1 19 0

2741639 T1 5 0

2741923 T1 3 0

2742115 T1 4 0

2742315 T1 40 0

2742425 T2 11 0

2742503 T1 13 0

2742619 T1 49 0

2742654 T1 19 0

2742721 T1 4 0

2743325 T1 3 0

2743424 T1 5 0

2743643 T1 26 0

2743844 T1 37 0

2744605 T1 2 0

2746045 T1 34 0

2746223 T2 1 0

2746577 T1 14 0

2746615 T1 14 3

2746691 T1 11 0

2746724 T2 16 0

2746802 T2 52 0

2747167 T1 27 0

2747444 T1 2 0

2747529 T1 29 0

2747958 T1 4 0

2748845 T1 4 0

2748993 T3 2 0

2750433 T1 19 0

2750822 T1 33 0

2751768 T1 23 0

2752162 T2 47 4

2752304 T1 18 0

2752413 T2 34 0

2752970 T1 7 0

2753610 T2 21 0

2754119 T2 2 0

2754166 T2 40 0

2754553 T1 15 0

2754797 T1 6 2

2754825 T1 16 0

2754848 T2 8 0

2755285 T1 14 0

2755333 T1 11 0

2755979 T1 36 0

2756684 T1 11 0

2756852 T1 11 0

2757943 T1 13 0

2758071 T1 3 0

2758487 T1 2 0

2758912 T1 22 0

2759247 T1 23 0

2759557 T1 11 0

2759624 T1 14 0

2761930 T1 4 0

2762937 T1 29 0

2763525 T1 3 0

2764146 T1 7 0

2764770 T1 3 0

2765157 T1 12 0

2765316 T1 6 0

2765727 T1 3 0

2765992 T1 12 0

2766031 T1 37 0

2766363 T1 1 0

2766428 T1 3 0

2766503 T1 24 0

2767057 T2 5 0

2767083 T1 4 0

2767929 T1 21 0

2767978 T1 7 0

2767979 T1 9 0

2769212 T1 5 0

2769275 T1 51 0

2769468 T1 21 0

2769624 T1 7 0

2769690 T2 6 0

2769738 T3 13 0

2770201 T1 14 0

2770389 T1 19 0

2770848 T1 8 4

2771309 T1 25 0

2771565 T1 36 0

2771641 T1 5 0

2772026 T1 14 0

2772057 T1 3 0

2772377 T1 13 0

2772451 T1 30 0

2772669 T1 12 0

2773318 T1 3 0

2773696 T1 2 0

2773864 T1 4 0

2774716 T1 35 0

2775204 T1 4 0

2776061 T1 18 0

2776626 T1 1 0

2777481 T1 3 0

2777638 T1 1 0

2777680 T1 25 0

2777782 T1 18 0

2778763 T2 10 0

2779260 T1 1 0

2779543 T1 1 0

2779572 T1 23 0

2779720 T1 36 0

2779730 T1 10 0

2780137 T1 9 0

2780469 T1 4 0

2780977 T1 4 0

2781391 T1 9 0

2782034 T1 6 0

2783169 T1 8 0

2783526 T1 12 0

2784260 T1 17 0

2784928 T1 55 0

2785401 T1 20 2

2785843 T1 4 0

2786246 T1 20 0

2786254 T1 30 0

2786275 T1 16 0

2786708 T1 28 0

2786785 T1 10 0

2786922 T1 6 0

2787173 T2 3 0

2787418 T1 6 0

2787942 T1 1 0

2788777 T3 23 2

2789059 T3 12 0

2789139 T1 14 0

2789216 T1 20 0

2789764 T1 16 0

2790049 T1 4 0

2790085 T1 32 0

2790856 T1 19 0

2791373 T1 15 0

2791436 T1 12 0

2791920 T1 9 0

2792000 T1 3 0

2792239 T1 90 0

2792600 T1 1 0

2793287 T1 4 0

2793292 T1 3 0

2793578 T1 41 0

2793590 T1 15 0

2793855 T1 4 0

2794363 T1 7 0

2794591 T2 15 0

2794665 T1 53 0

2794703 T2 26 0

2794911 T2 1 0

3500124 T1 18 0

3500177 T1 29 0

3501138 T1 16 0

3501167 T1 25 0

3501686 T1 6 0

3501810 T1 3 0

3501816 T3 2 0

3501928 T1 26 0

3502072 T2 13 1

3502280 T1 35 0

3502419 T1 12 0

3502533 T1 14 0

3502837 T1 13 0

3503734 T1 16 0

3504317 T1 16 0

3504564 T3 6 0

3504695 T1 3 0

3504738 T1 6 0

3504853 T1 18 0

3505029 T1 7 0

3505088 T1 25 0

3505179 T3 21 0

3505235 T2 10 0

3505828 T1 11 0

3505897 T3 6 0

3505925 T1 7 1

3506003 T1 9 0

3506031 T2 10 0

3506149 T1 8 0

3506187 T1 3 0

3506191 T1 14 0

3506227 T1 11 0

3506274 T3 38 2

3506285 T1 5 0

3506674 T2 21 0

3506720 T1 3 0

3508043 T1 13 0

3508344 T3 42 0

3508403 T1 15 0

3508770 T3 22 0

3508815 T1 21 0

3509604 T1 12 0

3510469 T1 8 0

3510566 T1 21 1

3510910 T1 45 0

3510912 T1 16 0

3511191 T1 34 0

3511355 T3 33 1

3511449 T2 26 0

3511785 T1 27 0

3511926 T1 4 0

3512026 T1 28 0

3512433 T1 40 0

3513163 T1 35 1

3513199 T1 12 0

3513481 T1 45 0

3515062 T1 5 0

3515540 T3 34 8

3515773 T1 7 0

3515798 T1 39 0

3515863 T1 6 0

3515928 T1 8 0

3516555 T3 25 3

3516692 T1 17 0

3516737 T1 25 0

3517078 T1 20 0

3517153 T1 17 0

3517549 T1 15 0

3517643 T1 10 0

3517691 T1 17 0

3518401 T1 3 0

3518748 T1 20 0

3518809 T2 3 0

3518943 T1 17 0

3519183 T1 2 0

3520109 T1 10 0

3520598 T1 13 0

3520688 T1 10 0

3520810 T2 7 0

3520905 T1 29 0

3521232 T1 12 0

3521491 T1 4 0

3521526 T1 7 0

3521538 T1 19 0

3521617 T1 3 0

3523048 T1 28 0

3523262 T2 21 0

3523270 T2 16 0

3523664 T1 37 0

3523680 T1 5 0

3524250 T1 39 0

3524293 T2 6 0

3524381 T1 36 0

3524472 T1 25 0

3524475 T1 17 0

3524633 T1 3 0

3524918 T1 17 0

3525165 T1 8 0

3525275 T1 32 0

3525565 T1 6 0

3526034 T1 27 0

3526139 T1 4 0

3526306 T1 24 0

3526341 T1 6 0

3526538 T1 11 0

3526805 T1 8 0

3526830 T1 4 0

3526838 T2 42 0

3527014 T1 2 0

3527194 T1 47 0

3527410 T1 17 0

3527542 T1 1 0

3527949 T3 13 11

3528199 T1 4 0

3528381 T1 2 0

3528423 T1 3 0

3528722 T1 8 0

3529038 T2 7 0

3529164 T1 3 0

3529191 T1 6 0

3529233 T1 12 0

3529247 T1 12 0

3529248 T1 39 0

3529265 T1 23 0

3529290 T1 1 0

3529343 T1 7 0

3529414 T1 30 0

3529478 T1 18 0

3529500 T1 90 0

3529695 T2 13 0

3529867 T2 23 0

3529945 T1 16 0

3530188 T1 21 0

3530260 T3 7 2

3530314 T2 20 0

3530330 T1 23 0

3530505 T1 38 0

3530528 T1 10 0

3530547 T1 24 0

3530568 T2 26 0

3530626 T2 5 0

3530641 T3 5 0

3530693 T1 25 0

3530797 T1 26 0

3530879 T2 4 0

3530954 T1 15 0

3531015 T1 1 0

3531049 T1 22 0

3531071 T1 3 0

3531112 T1 10 0

3531228 T1 2 0

3531295 T1 24 0

3531304 T1 12 0

3531327 T1 19 0

3531351 T2 3 0

3531357 T1 4 0

3531361 T1 15 0

3531374 T1 6 0

3531408 T2 6 0

3531418 T1 11 0

3531437 T1 14 0

3531455 T1 5 0

3531467 T1 9 0

3532523 T1 7 0

3532580 T1 71 0

3532656 T2 39 0

3532935 T2 22 0

3533385 T1 13 0

3533890 T1 22 0

3533938 T1 11 0

3533995 T1 23 0

3534041 T1 6 0

3534046 T2 17 0

3534064 T1 4 0

3534105 T1 14 0

3534119 T1 12 0

3534124 T1 23 0

3534770 T1 5 0

3534989 T1 12 0

3535096 T1 8 0

3535802 T1 19 0

3536041 T1 25 0

3536466 T1 11 0

3536472 T1 14 0

3536568 T1 20 0

3536592 T1 32 0

3536668 T2 18 0

3536725 T3 17 6

3536809 T1 21 0

3536901 T2 12 0

3537103 T1 23 0

3537260 T1 23 0

3538263 T1 5 0

3538403 T3 9 3

3538530 T1 29 0

3538743 T2 25 1

3539138 T1 47 0

3539206 T1 25 0

3539254 T2 39 0

3539272 T1 13 0

3539278 T1 10 0

3539518 T1 16 0

3540233 T1 8 0

3540241 T1 24 0

3540293 T1 28 0

3540372 T1 12 0

3540379 T1 5 0

3540380 T1 50 0

3540419 T2 1 0

3541274 T2 7 0

3541746 T2 13 0

3541887 T1 24 0

3542005 T1 14 0

3542321 T2 13 0

3542705 T1 18 0

3542707 T1 16 0

3542960 T1 5 0

3543221 T3 4 0

3543568 T1 2 0

3543648 T1 31 0

3544085 T1 14 0

3544218 T1 15 0

3544558 T1 14 0

3545299 T1 26 0

3545983 T1 18 0

3546165 T1 27 0

3546171 T1 5 0

3546181 T1 1 0

3546202 T1 10 0

3546562 T1 23 0

3546641 T1 8 0

3546759 T1 2 0

3546826 T1 14 0

3546853 T1 7 0

3546902 T2 22 1

3546937 T1 4 0

3547178 T1 1 0

3547289 T1 11 0

3548512 T4 3 0

3548603 T1 19 0

3549335 T1 25 0

3549928 T1 9 0

3550662 T1 4 0

3550671 T1 2 0

3550897 T2 14 0

3550939 T2 15 0

3551152 T3 28 0

3551384 T1 6 1

3551596 T1 2 0

3551802 T1 13 0

3552843 T1 9 0

3553119 T2 58 0

3553362 T1 33 0

3553770 T1 9 0

3553889 T3 2 0

3554015 T1 3 0

3554382 T1 8 0

3554934 T1 16 0

3556207 T1 25 0

3556254 T1 20 0

3556357 T1 2 0

3556556 T3 2 0

3556658 T1 9 0

3556682 T1 11 0

3556699 T1 23 0

3556711 T1 22 0

3556732 T1 23 0

3556738 T1 14 0

3556740 T1 19 0

3556755 T1 21 0

3557435 T1 20 0

3557522 T1 13 0

3557598 T1 1 0

3557608 T1 22 0

3557622 T2 21 0

3557633 T3 13 0

3557794 T1 18 0

3558144 T1 10 0

3558481 T1 22 0

3558866 T1 19 0

3559107 T1 7 0

3559312 T1 6 0

3559454 T1 8 0

3559987 T1 4 0

3560161 T1 12 0

3560366 T1 3 0

3560435 T1 11 0

3560504 T1 11 0

3561130 T1 11 0

3561413 T1 48 0

3561584 T1 7 0

3562008 T1 15 0

3562113 T1 12 0

3562784 T1 9 0

3563253 T1 9 0

3563259 T1 25 0

3563271 T1 20 0

3563282 T1 4 0

3563359 T1 2 0

3563393 T2 11 0

3563454 T1 7 0

3563829 T1 7 0

3563969 T1 20 0

3564257 T1 8 0

3564278 T1 14 0

3564464 T1 1 0

3565050 T1 13 0

3565656 T1 3 0

3566046 T1 8 0

3566117 T1 3 0

3566778 T2 29 0

3566809 T1 12 0

3566837 T1 8 0

3567568 T1 8 0

3568642 T1 4 0

3568740 T1 12 0

3568787 T3 10 1

3568944 T2 5 0

3569129 T1 20 0

3569414 T1 22 0

3569766 T1 26 0

3569886 T1 36 0

3570219 T1 7 0

3570360 T3 2 0

3570895 T2 32 0

3571326 T1 2 0

3571571 T1 35 0

3572353 T1 21 0

3572850 T1 3 0

3573066 T1 6 0

3573363 T1 14 0

3573402 T1 11 0

3573481 T1 5 0

3574283 T1 14 0

3574405 T1 10 0

3574587 T1 17 0

3574895 T1 21 0

3575044 T1 18 0

3576005 T2 19 0

3576274 T3 8 0

3576480 T1 27 0

3577094 T1 22 0

3577119 T1 15 0

3577179 T1 2 0

3577462 T1 27 0

3577593 T1 17 0

3577678 T1 15 0

3577730 T1 50 0

3577882 T1 8 0

3578188 T2 13 0

3578411 T1 27 0

3578520 T1 1 0

3578657 T1 22 0

3578852 T1 10 0

3579576 T1 19 0

3579838 T1 3 0

3579895 T1 13 0

3579994 T1 10 0

3580235 T1 30 0

3580501 T1 13 0

3580940 T1 9 0

3581231 T1 17 0

3581507 T1 9 0

3582054 T1 11 0

3582292 T1 19 0

3582593 T1 17 0

3582737 T1 3 0

3582867 T1 25 0

3582870 T1 41 0

3582906 T2 1 0

3583021 T1 6 0

3583087 T1 33 0

3583351 T3 22 1

3583476 T1 31 0

3583521 T1 33 0

3583558 T1 26 0

3583756 T1 16 0

3583943 T1 27 0

3583948 T1 32 0

3584297 T1 6 0

3584551 T3 7 0

3584624 T1 13 0

3586727 T1 34 0

3586749 T1 12 0

3586990 T1 11 0

3587055 T1 13 0

3587062 T1 10 0

3587172 T1 6 0

3587645 T1 2 0

3588625 T1 3 0

3588938 T1 13 0

3589446 T1 3 0

3589522 T1 6 1

3589861 T3 8 0

3590263 T1 18 0

3590609 T1 3 0

3590901 T1 26 0

3592282 T1 15 0

3592539 T1 12 0

3592551 T1 7 0

3592579 T1 2 0

3592593 T2 20 0

3592618 T1 10 0

3592637 T1 15 0

3593143 T1 6 0

3593349 T1 11 0

3594079 T1 7 0

3594172 T1 11 0

3594245 T1 4 1

3594492 T1 10 0

3594657 T1 12 0

3595077 T3 40 1

3595825 T2 17 0

3597304 T1 17 0

3597455 T3 4 0

3597536 T1 32 1

3598343 T1 8 0

3599894 T1 1 0

3600216 T1 19 0

3600464 T1 11 0

3601378 T1 32 0

3601400 T1 8 0

3601437 T1 18 8

3601450 T1 19 0

3601481 T1 35 0

3601636 T1 8 0

3601661 T1 19 0

3601675 T1 6 0

3601723 T2 31 0

3601770 T1 6 0

3601790 T1 9 0

3601809 T3 1 0

3601827 T1 10 0

3601840 T2 5 0

3602192 T1 8 0

3602588 T1 19 0

3603138 T1 5 0

3603408 T1 12 0

3603496 T2 21 1

3603798 T1 34 0

3603819 T2 4 0

3603822 T1 20 0

3603841 T2 9 0

3603903 T1 4 0

3603923 T1 4 0

3603937 T1 2 0

3604094 T1 22 0

3604929 T1 4 0

3605415 T1 6 4

3605483 T3 28 1

3605612 T1 19 0

3605800 T1 22 0

3606030 T1 20 0

3606390 T3 10 0

3606548 T1 16 0

3607067 T1 8 0

3607102 T1 14 0

3607263 T1 11 0

3607657 T1 28 0

3609428 T1 17 0

3609596 T1 4 0

3609749 T2 21 0

3609994 T1 60 0

3610331 T2 5 0

3610363 T1 3 0

3610400 T1 6 0

3610410 T1 31 0

3610701 T1 25 0

3610852 T1 21 0

3611586 T1 6 0

3612197 T1 60 0

3612200 T1 29 0

3612212 T1 25 0

3612660 T1 7 0

3612665 T1 15 0

3612715 T1 7 3

3612719 T1 7 0

3613016 T1 16 0

3613155 T1 26 0

3613164 T1 1 0

3613220 T1 4 0

3613230 T1 13 0

3613261 T1 11 0

3613299 T2 12 0

3613898 T3 14 0

3614488 T1 1 1

3614666 T1 2 0

3614862 T1 17 0

3614978 T1 20 0

3615040 T1 12 0

3615878 T1 4 0

3616332 T1 18 0

3616454 T1 7 0

3616521 T3 39 2

3616556 T1 29 0

3617061 T1 4 0

3617497 T1 33 0

3617717 T1 24 0

3617772 T3 41 0

3617815 T1 17 0

3618191 T2 19 0

3618569 T2 31 0

3618958 T1 35 0

3619805 T1 4 0

3619929 T1 35 0

3620086 T3 47 0

3620349 T2 8 0

3621570 T1 7 0

3621588 T1 11 0

3622226 T1 30 0

3622298 T1 13 0

3622397 T3 5 0

3622695 T1 13 0

3622720 T1 11 0

3622766 T1 7 0

3622780 T2 6 0

3622798 T1 5 0

3622806 T3 7 0

3622882 T1 28 0

3623153 T1 16 0

3623225 T3 3 0

3623402 T1 7 0

3624373 T2 10 0

3624445 T1 11 0

3625563 T1 12 0

3625697 T1 33 0

3625858 T1 5 0

3625992 T1 17 0

3626301 T1 31 0

3626541 T1 9 0

3626611 T1 68 0

3627150 T2 8 0

3627165 T1 9 0

3628170 T1 7 0

3628347 T2 17 0

3628604 T2 28 0

3628955 T1 12 0

3629146 T1 15 0

3629364 T2 5 0

3629375 T1 38 0

3629393 T1 22 0

3630035 T1 25 0

3630790 T1 4 0

3630874 T2 26 0

3630992 T2 4 0

3631021 T1 24 1

3631339 T1 5 0

3631343 T1 40 0

3631352 T1 3 0

3631406 T2 2 0

3631422 T1 11 0

3631459 T1 28 0

3631481 T1 17 0

3632705 T1 2 0

3632723 T1 17 1

3632821 T1 29 0

3633372 T1 2 0

3633726 T1 3 0

3633767 T3 19 4

3633801 T1 4 0

3633906 T1 4 0

3633928 T2 3 0

3634129 T1 19 0

3634141 T1 6 0

3634182 T1 28 0

3634187 T1 36 2

3634257 T1 17 0

3634449 T1 8 0

3634644 T1 39 0

3634664 T1 31 0

3634681 T1 6 0

3634778 T1 25 0

3634784 T1 2 0

3634806 T1 13 0

3634838 T1 17 0

3634843 T1 32 0

3634882 T2 17 0

3634885 T1 9 0

3634901 T1 18 0

3635513 T1 30 0

3635727 T1 42 1

3635756 T1 28 0

3635802 T1 12 0

3635865 T1 42 0

3635909 T1 2 0

3636124 T1 33 0

3636206 T1 27 0

3636216 T1 18 0

3636351 T1 9 0

3636597 T1 29 0

3636641 T2 22 0

3636794 T3 36 0

3639592 T3 18 0

3639596 T1 23 0

3641493 T1 3 0

3641517 T1 17 0

3641675 T1 37 0

3641679 T1 8 0

3641708 T1 11 0

3641716 T1 21 0

3641719 T2 31 0

3641722 T1 29 2

3641725 T1 5 0

3641729 T2 60 0

3641734 T1 32 0

3641760 T1 17 0

3641772 T1 16 0

3641812 T1 3 0

3641816 T2 15 1

3641848 T1 14 0

3641947 T3 13 0

3641963 T1 66 0

3641970 T3 3 0

3642383 T1 17 0

3642421 T1 16 0

3642505 T1 17 0

3642697 T1 1 0

3643874 T1 14 0

3644504 T1 23 0

3644935 T1 4 0

3645233 T1 22 0

3645572 T1 28 0

3645707 T2 16 0

3645836 T1 34 0

3646597 T1 24 0

3646816 T2 26 0

3647246 T1 10 0

3647848 T1 7 0

3647904 T1 17 0

3647940 T1 9 0

3647971 T1 5 0

3648175 T3 10 0

3648219 T1 22 0

3648475 T1 4 0

3648577 T2 9 0

3649462 T3 17 0

3649599 T1 29 0

3650727 T1 25 0

3650881 T1 28 0

3650999 T1 9 0

3651250 T3 3 0

3652068 T3 15 0

3652084 T1 10 0

3652088 T1 20 0

3652851 T1 5 0

3652972 T1 26 0

3653160 T3 36 0

3653319 T1 41 0

3653525 T1 37 0

3653601 T1 10 0

3653863 T1 21 0

3653945 T2 5 0

3653962 T1 41 0

3654015 T1 16 0

3654020 T1 8 0

3654060 T1 23 0

3654066 T1 14 0

3654256 T1 7 0

3654357 T1 5 0

3654883 T1 17 0

3655315 T1 21 0

3655329 T1 6 0

3655348 T1 20 0

3655427 T3 11 0

3655597 T1 4 0

3655669 T1 12 0

3655727 T1 16 1

3655791 T1 35 0

3655937 T2 8 0

3656666 T1 22 0

3656709 T2 13 0

3656721 T1 23 0

3656738 T1 27 0

3656839 T1 3 0

3656930 T1 20 0

3657123 T1 25 0

3657151 T1 21 0

3657158 T1 13 0

3657166 T1 1 0

3657174 T3 36 0

3657192 T1 2 0

3657196 T1 7 0

3657214 T1 31 1

3657765 T1 4 0

3657782 T1 5 0

3658213 T1 7 0

3658578 T1 8 0

3659540 T1 18 0

3659546 T1 2 0

3660107 T1 20 0

3660114 T1 28 0

3660123 T1 28 0

3660125 T2 9 0

3660176 T1 3 0

3660443 T1 16 0

3660448 T1 30 0

3660485 T1 13 0

3660500 T1 8 0

3660513 T1 16 0

3660835 T1 4 0

3661037 T2 20 0

3661050 T1 3 0

3661139 T1 28 0

3661156 T1 3 0

3661441 T1 3 0

3661565 T3 15 0

3662140 T1 15 0

3662424 T1 15 0

3662474 T1 28 0

3662512 T1 13 0

3662525 T1 16 0

3662673 T1 2 0

3663390 T1 15 0

3663536 T1 12 0

3663847 T1 3 0

3664132 T1 14 0

3664435 T1 2 0

3664641 T1 14 0

3664712 T1 4 0

3664864 T1 10 0

3664873 T1 15 0

3664886 T2 15 0

3664904 T1 2 0

3664923 T1 3 0

3664946 T1 13 1

3664965 T1 7 0

3664975 T1 6 0

3664999 T1 2 0

3665034 T1 6 0

3665197 T1 21 0

3665219 T1 22 0

3665353 T2 58 0

3665501 T2 4 0

3665613 T1 9 0

3666004 T1 11 0

3666983 T2 26 0

3668563 T1 32 0

3668568 T1 6 0

3668574 T1 33 0

3668780 T1 21 2

3668796 T1 21 0

3668941 T1 50 0

3669279 T1 16 0

3669323 T1 14 0

3669327 T1 16 0

3669337 T1 17 0

3669344 T1 1 0

3669398 T1 15 0

3669765 T1 27 0

3670224 T1 3 0

3670560 T1 20 0

3670609 T1 29 0

3670687 T1 6 0

3670741 T1 47 0

3670971 T1 5 0

3671039 T1 10 0

4375005 T1 4 0

4375177 T1 18 0

4375370 T1 18 0

4375926 T1 15 0

4376538 T1 13 0

4377033 T1 23 0

4377078 T1 14 0

4377208 T1 23 0

4377483 T1 11 0

4377750 T1 16 0

4377803 T1 11 0

4377945 T1 37 0

4378103 T1 25 0

4378164 T1 3 0

4378486 T1 23 0

4378847 T1 20 0

4378878 T1 36 0

4379418 T1 22 0

4379608 T1 10 0

4380350 T1 2 0

4380357 T1 12 0

4380362 T1 10 0

4380437 T1 32 0

4380446 T3 28 0

4380521 T3 37 0

4380554 T3 17 0

4380564 T1 7 0

4380575 T1 3 0

4380929 T3 9 3

4381597 T1 4 0

4381612 T1 20 0

4381659 T1 8 0

4381814 T1 29 0

4382293 T2 27 0

4382436 T1 24 0

4382503 T1 27 0

4382556 T3 15 0

4382929 T1 30 0

4383046 T1 3 0

4383821 T1 10 0

4384054 T1 31 0

4384104 T1 8 0

4384478 T1 19 0

4384613 T2 15 0

4385066 T1 17 0

4385177 T1 25 0

4385464 T1 8 0

4385469 T1 21 0

4385566 T1 23 0

4385812 T1 3 0

4386112 T1 38 0

4386326 T1 13 0

4386332 T3 35 0

4386426 T2 8 0

4386553 T1 6 0

4386597 T2 16 0

4386776 T2 27 0

4386886 T1 29 0

4386963 T1 21 0

4387016 T1 8 0

4387048 T1 11 0

4387069 T1 38 0

4387076 T1 16 0

4387088 T1 6 0

4387097 T1 20 0

4387380 T1 12 0

4387535 T2 18 0

4387720 T3 37 0

4387840 T1 6 0

4388079 T3 13 0

4388670 T1 3 0

4388727 T1 26 0

4388729 T1 26 0

4388912 T1 13 0

4389062 T1 1 0

4389095 T1 25 0

4389218 T1 2 0

4389246 T2 8 0

4389256 T1 21 0

4389295 T1 16 0

4389302 T1 37 0

4389319 T1 20 0

4389334 T1 8 0

4389344 T2 14 0

4389595 T1 31 1

4389668 T1 16 0

4389737 T1 3 0

4390033 T1 2 0

4390578 T1 7 0

4390790 T1 12 0

4390895 T1 14 0

4390935 T1 14 0

4390981 T1 23 0

4390994 T1 18 0

4391037 T1 16 0

4391136 T1 14 0

4391293 T1 27 0

4391315 T1 23 0

4391689 T1 24 0

4392171 T1 19 0

4392322 T1 5 0

4392559 T1 6 0

4392671 T1 19 0

4392700 T1 56 0

4392717 T1 4 0

4392720 T1 4 0

4392746 T1 52 0

4392921 T1 45 0

4392957 T2 18 0

4393017 T1 16 0

4393309 T1 12 0

4393393 T2 14 0

4393631 T1 48 0

4393890 T2 12 0

4394015 T1 25 0

4394143 T1 11 0

4394222 T1 46 0

4394224 T3 34 0

4394877 T2 2 0

4395015 T1 21 0

4395021 T1 9 0

4395145 T1 37 0

4395409 T1 5 0

4395541 T3 4 0

4395738 T1 37 0

4396424 T4 28 0

4396721 T1 19 0

4396780 T1 6 0

4396928 T1 14 0

4397427 T1 30 0

4397548 T1 26 0

4397583 T1 23 0

4397660 T1 33 0

4397668 T1 12 0

4397701 T1 2 0

4397754 T1 24 0

4397922 T1 12 0

4398360 T2 13 0

4399031 T1 32 0

4399051 T1 6 0

4399269 T1 21 0

4399359 T2 2 0

4399958 T1 15 1

4400241 T3 6 0

4400300 T1 4 0

4400313 T1 6 0

4400477 T1 18 0

4400622 T3 7 0

4400748 T3 7 0

4401528 T1 14 0

4401615 T1 34 0

4401649 T1 13 0

4401760 T1 6 0

4401775 T1 27 0

4401797 T1 4 0

4401855 T1 14 0

4401922 T1 21 0

4402475 T1 13 0

4402634 T1 14 0

4402813 T1 7 0

4403639 T3 10 0

4404551 T2 13 0

4404614 T1 4 0

4404631 T3 19 2

4404841 T1 3 0

4405067 T1 22 0

4405180 T2 39 0

4405220 T1 12 0

4405478 T2 33 0

4405563 T3 55 0

4405826 T1 7 0

4405942 T1 6 0

4406353 T1 8 0

4406547 T1 10 0

4406663 T1 3 0

4406753 T1 35 0

4407030 T1 20 0

4407070 T1 32 0

4407071 T1 3 0

4407725 T1 23 0

4407836 T1 17 0

4407910 T1 11 0

4407985 T1 40 0

4408074 T3 10 7

4408243 T1 43 0

4408540 T1 17 0

4408561 T3 4 0

4408777 T1 22 0

4408824 T2 17 0

4408827 T1 2 0

4408902 T1 21 0

4409176 T1 18 0

4409238 T1 10 0

4409240 T1 7 0

4409380 T1 4 0

4409400 T1 19 0

4409431 T1 9 0

4409807 T1 27 0

4410075 T2 49 0

4410207 T1 7 0

4410261 T1 7 0

4410324 T2 61 0

4410334 T3 2 0

4410396 T2 47 2

4410435 T1 31 0

4410462 T3 33 0

4410489 T1 9 0

4410516 T1 19 0

4410549 T1 22 0

4410607 T3 8 0

4410852 T3 10 0

4410886 T1 6 0

4410969 T1 24 0

4411359 T1 42 0

4411568 T1 27 0

4412087 T2 4 0

4412093 T1 20 0

4412115 T1 12 0

4412122 T1 7 0

4412148 T1 3 0

4412162 T1 37 0

4412173 T3 13 2

4412187 T3 2 0

4412189 T1 5 0

4412208 T1 12 0

4412214 T1 43 0

4412244 T1 13 0

4412258 T1 17 0

4412265 T1 12 0

4412404 T1 5 0

4412427 T1 3 0

4412496 T1 17 0

4412829 T1 11 0

4412838 T2 28 1

4413048 T1 14 0

4413677 T1 17 0

4413930 T1 21 0

4414087 T1 8 0

4414801 T1 24 0

4414879 T1 24 0

4415026 T1 2 0

4415881 T1 26 0

4416236 T1 6 0

4416266 T1 7 0

4416759 T3 5 0

4416864 T1 13 0

4416919 T1 12 0

4416979 T1 39 0

4417173 T1 25 0

4417369 T2 23 0

4417551 T1 2 0

4417560 T3 13 3

4417599 T1 7 0

4417606 T1 16 0

4417697 T1 24 0

4417829 T1 17 0

4418233 T1 2 0

4418754 T2 19 0

4418815 T1 3 0

4418827 T1 17 0

4419073 T1 12 0

4419075 T3 27 0

4419425 T1 1 0

4419523 T1 9 0

4419579 T1 4 0

4419749 T1 30 0

4419995 T1 51 0

4419997 T1 25 0

4419999 T1 8 0

4420020 T1 33 0

4420037 T2 18 2

4420636 T1 7 0

4420677 T1 1 0

4420990 T1 22 0

4421423 T1 1 0

4421552 T1 26 2

4421586 T1 1 0

4421587 T1 2 0

4421594 T1 4 0

4421747 T1 1 0

4421879 T1 27 0

4422055 T1 36 0

4422071 T1 7 0

4422219 T2 40 0

4422977 T1 26 0

4423310 T1 17 0

4423315 T1 22 0

4424048 T1 20 0

4424835 T3 16 0

4424857 T1 19 0

4425033 T2 7 0

4425211 T2 1 1

4425519 T1 35 0

4426090 T2 7 0

4426325 T1 26 0

4426410 T1 7 0

4426853 T2 12 0

4427148 T1 28 0

4427260 T1 13 0

4427323 T1 30 0

4427330 T1 6 0

4427404 T2 49 0

4427486 T1 8 0

4427513 T1 6 0

4427556 T3 6 0

4427703 T1 28 0

4427943 T1 18 0

4428458 T2 3 0

4428534 T3 6 2

4428544 T2 31 0

4428740 T1 20 0

4428921 T1 20 0

4428947 T1 18 0

4429124 T1 35 0

4429371 T1 8 0

4429872 T1 6 0

4430188 T1 6 0

4430241 T1 19 0

4430353 T1 22 0

4430830 T1 12 0

4430939 T1 27 0

4430974 T1 4 0

4430992 T1 9 0

4431044 T1 19 0

4432384 T1 9 0

4433896 T2 16 0

4433916 T1 50 0

4434136 T1 23 0

4434166 T1 12 0

4434225 T1 23 0

4434561 T1 18 0

4434605 T1 36 0

4434667 T1 41 0

4434669 T1 10 0

4434933 T3 3 0

4434972 T1 26 0

4435486 T1 13 0

4435491 T1 24 0

4435496 T1 40 0

4435499 T1 37 0

4435579 T1 33 0

4435580 T1 36 0

4435591 T1 28 0

4435722 T1 30 0

4435776 T1 23 0

4435779 T1 20 0

4435793 T1 9 0

4435796 T1 29 0

4435830 T1 24 1

4435874 T1 13 0

4436428 T1 3 0

4436832 T1 9 0

4437199 T1 18 0

4437811 T1 5 0

4437846 T1 2 0

4437868 T1 5 0

4437921 T1 15 0

4437942 T1 14 0

4438022 T1 10 0

4438077 T1 38 0

4438115 T1 19 0

4438937 T1 22 0

4439101 T3 28 1

4439310 T1 29 0

4441145 T4 8 0

4441157 T1 9 0

4441221 T1 9 0

4441647 T3 5 0

4442117 T1 24 0

4442162 T1 10 0

4442164 T1 22 0

4442294 T2 8 0

4443247 T1 23 0

4444460 T1 13 0

4445075 T1 16 0

4445076 T1 46 0

4445208 T1 12 0

4445330 T2 13 0

4446381 T1 37 0

4446477 T1 8 0

4446509 T1 19 0

4446554 T1 15 0

4446992 T3 8 0

4447224 T1 20 0

4447356 T1 34 0

4447632 T2 16 0

4447635 T1 15 2

4447653 T1 19 0

4447705 T1 15 0

4447722 T1 13 0

4449296 T1 9 1

4449302 T1 17 0

4449303 T1 24 0

4449323 T1 5 0

4449360 T1 27 0

4450849 T1 2 0

4451058 T1 32 0

4454159 T1 1 0

4454239 T1 23 0

4454597 T2 13 0

4454872 T1 23 0

4458704 T1 40 0

4459249 T1 7 0

4459598 T1 19 0

4460263 T1 7 0

4460320 T2 3 0

4460326 T3 21 0

4461084 T1 27 0

4461132 T3 9 0

4461153 T1 33 0

4461470 T1 9 0

4461484 T2 10 0

4461722 T2 19 0

4461802 T1 25 0

4461830 T2 18 0

4461863 T1 25 0

4461866 T1 17 0

4461967 T1 17 0

4462086 T1 15 0

4462181 T1 44 0

4462287 T1 18 0

4462350 T1 17 0

4462965 T1 15 0

4463036 T3 17 0

4463274 T1 17 0

4463352 T1 1 0

4463460 T1 11 0

4463616 T1 21 0

4463671 T1 8 0

4463759 T3 21 0

4463858 T1 2 0

4463887 T1 6 0

4464095 T1 4 0

4464105 T1 17 0

4464489 T1 33 0

4464563 T1 9 0

4464711 T1 4 0

4464832 T2 17 0

4464934 T1 34 0

4465145 T1 22 0

4465156 T1 42 0

4465165 T3 18 3

4465585 T1 11 0

4466071 T1 31 0

4466081 T1 12 0

4466810 T1 4 0

4467070 T3 38 0

4467294 T1 49 0

4469122 T3 33 0

4469657 T1 30 0

4469792 T1 28 0

4469824 T2 36 0

4469843 T1 26 0

4470323 T1 20 0

4470863 T3 5 0

4474783 T1 27 1

4474907 T1 23 0

4474948 T1 16 0

4474951 T1 14 0

4474954 T2 8 2

4474990 T3 18 0

4474993 T1 12 0

4475097 T1 4 0

4475099 T2 24 3

4475201 T1 11 0

4475568 T1 55 2

4475675 T3 27 1

4475767 T1 13 0

4475783 T1 31 0

4475788 T2 1 0

4475796 T1 2 0

4475863 T1 12 0

4475872 T1 31 0

4475929 T1 50 0

4475935 T1 38 0

4475943 T1 11 0

4475962 T1 12 0

4476002 T1 21 0

4476013 T2 10 0

4476015 T1 27 0

4476026 T1 7 0

4476096 T1 1 0

4476108 T2 22 0

4476113 T1 10 0

4476161 T1 7 0

4476181 T3 8 0

4476190 T3 47 9

4476235 T1 23 0

4476250 T1 27 0

4476391 T1 22 0

4476463 T1 12 0

4476504 T1 1 0

4476507 T1 3 0

4476718 T1 14 0

4476744 T1 6 0

4476911 T2 22 0

4477289 T1 1 0

4477356 T3 13 0

4477366 T1 18 0

4477380 T1 12 0

4477936 T1 17 1

4477946 T1 5 0

4477960 T1 3 0

4477966 T1 11 0

4477995 T1 7 0

4478037 T1 13 0

4478155 T2 34 0

4478208 T1 7 0

4478329 T1 10 0

4478330 T1 7 0

4478336 T3 8 0

4478389 T1 5 0

4478395 T3 4 0

4478400 T3 40 0

4478406 T1 23 0

4478621 T1 2 0

4478626 T1 18 0

4478988 T1 6 0

4478992 T1 9 0

4478994 T1 15 0

4478996 T1 12 0

4479062 T1 6 0

4479136 T1 21 0

4479186 T1 23 0

4479201 T1 16 0

4479211 T1 8 0

4479251 T1 20 0

4479252 T1 18 0

4479255 T1 9 0

4479268 T3 14 0

4479273 T1 17 0

4479278 T1 8 0

4479440 T1 7 0

4479596 T1 32 0

4479627 T1 11 0

4479630 T1 32 0

4479636 T1 24 0

4479649 T2 16 0

4479664 T1 34 0

4479954 T1 3 0

4480313 T1 4 0

4480317 T1 11 0

4480354 T4 4 0

4480355 T2 10 0

4480592 T1 7 0

4480595 T1 37 0

4480628 T1 32 0

4480630 T1 7 0

4480632 T1 12 0

4480635 T1 15 0

4481875 T1 22 0

4482144 T1 35 0

4482162 T1 60 0

4482164 T1 14 0

4482820 T1 46 0

4482876 T1 21 0

4483006 T2 10 0

4483026 T2 11 0

4483029 T1 20 0

4487012 T1 24 0

4491408 T2 39 0

4491433 T2 22 0

4491448 T1 21 0

4491452 T1 11 0

4491904 T1 14 0

4517262 T1 31 0

7013115 T1 1 0

8337498 T1 7 0

8341550 T1 6 0

8341745 T1 15 0

8341766 T1 3 0

8341875 T1 12 0

8342227 T1 32 0

8342919 T1 12 1

8343586 T1 2 0

8343724 T1 5 0

8344024 T1 2 0

8344085 T1 3 0

8344186 T1 3 0

8344715 T1 3 0

8344935 T1 22 0

8346175 T1 1 0

8348074 T2 1 0

8348110 T1 1 0

8348383 T2 10 0

8349149 T1 19 0

8349335 T1 2 0

8349422 T1 1 0

8351129 T1 3 0

8353198 T1 5 0

8353871 T1 2 0

8354335 T1 7 0

8354450 T1 7 0

8354655 T1 2 0

8354678 T1 9 0

8355123 T1 1 0

8355189 T1 11 0

8357550 T1 6 0

8357719 T1 9 0

8357783 T1 7 0

8357818 T1 3 0

8358199 T1 2 0

8358296 T1 2 0

8359123 T1 1 0

8361084 T1 5 0

8361590 T1 1 0

8362155 T1 3 0

8362287 T1 7 0

8363068 T1 2 0

8363476 T1 1 0

8364566 T1 12 0

8365559 T1 5 0

8365702 T1 16 0

8365725 T1 1 0

8366289 T1 5 0

8366759 T1 5 0

8367462 T1 10 0

8368058 T2 6 0

8369247 T1 21 0

8369396 T1 1 0

8369736 T2 12 0

8370459 T1 10 0

8371911 T1 1 0

8372183 T2 1 0

8373409 T1 7 0

8374113 T1 7 0

8374122 T1 6 0

8374211 T2 6 0

8375134 T1 6 0

8375729 T1 3 0

8375806 T1 3 0

8376441 T1 7 0

8379625 T1 8 0

8379689 T1 3 0

8379761 T1 4 0

8379781 T1 5 0

8379790 T1 1 0

8379967 T1 11 0

8380492 T1 15 0

8381069 T1 2 0

8382169 T1 1 0

8382925 T2 5 0

8383131 T1 6 0

9334893 T1 3 0

9335003 T1 9 0

9335493 T1 5 0

9336034 T1 2 0

9336480 T1 12 0

9339158 T1 1 0

9339350 T2 5 0

9340551 T1 8 0

9342511 T1 1 0

9342625 T1 1 0

9342693 T3 10 0

9344108 T1 5 0

9344329 T1 3 0

9345538 T2 7 0

9347595 T1 8 0

9348466 T1 4 0

9349077 T1 26 0

9349239 T1 1 0

9349479 T2 1 0

9350751 T1 3 0

9352342 T1 2 0

9352637 T1 18 0

9353083 T3 1 0

9353175 T1 6 0

9354069 T2 6 0

9354339 T1 3 0

9355010 T1 12 0

9355725 T1 2 0

9355767 T1 1 0

9355844 T1 1 0

9355852 T1 2 0

9355856 T1 9 0

9356215 T1 6 0

9357058 T1 2 0

9357805 T1 6 0

9359071 T2 4 0

9359313 T1 9 0

9360260 T1 2 0

9363336 T1 2 0

9364122 T1 5 1

9364275 T1 4 0

9365768 T1 1 1

9366243 T1 18 0

9366336 T3 1 1

9366622 T1 4 0

9367259 T2 1 0

9368966 T1 2 0

9369123 T1 2 0

9369350 T1 5 0

9369817 T1 3 0

9371862 T1 1 0

9372006 T4 2 0

9372063 T1 10 0

9372171 T1 1 0

9374681 T1 9 0

9374705 T1 1 0

9375102 T1 3 0

9375308 T1 1 0

9375327 T1 42 0

9375975 T2 5 0

9376531 T1 9 0

9377302 T1 2 0

9378244 T1 1 0

9378470 T2 23 0

9378514 T1 6 0

9378590 T2 10 0

9379667 T1 35 0

9381627 T1 1 0

9383418 T3 17 0

9383685 T1 12 0

9383775 T1 4 0

9384134 T1 4 0

9384174 T1 8 0

9384236 T1 26 0

9384639 T1 1 0

9386131 T1 1 0

9387198 T3 2 0

9387336 T1 3 0

9387365 T1 19 0

9387419 T1 5 0

9387457 T1 2 0

9388039 T1 4 0

9388308 T1 3 0

9388942 T1 1 0

9391138 T1 3 0

9391356 T1 2 0

9391500 T2 1 0

9392771 T2 1 0

9393230 T1 4 0

9393385 T1 1 0

9393830 T1 21 0

9393946 T1 10 0

9395409 T1 1 0

9397741 T1 1 0

9400691 T1 1 0

9400951 T1 3 0

9401746 T1 3 0

9402697 T1 2 0

9403000 T1 3 0

9403723 T1 6 0

9406702 T1 2 0

9407327 T1 2 0

9408516 T1 5 0

9408946 T1 3 0

9409165 T2 1 0

9409297 T1 2 0

9411082 T2 1 1

9414317 T1 10 0

9417658 T1 2 0

9417687 T2 14 0

9417754 T1 7 0

9418095 T1 1 0

9419102 T1 4 0

9421291 T1 1 0

9421459 T1 2 0

9421702 T1 1 0

9421707 T1 6 0

9421757 T1 3 0

9422444 T2 5 0

9422646 T1 4 0

9422973 T1 7 0

9422987 T1 11 0

9423008 T1 3 0

9423117 T1 12 0

9423126 T1 4 0

9423162 T1 2 0

9423179 T1 5 0

9423736 T1 2 0

9423783 T1 3 0

9424616 T1 21 0

9424780 T1 9 0

9425176 T1 7 2

9425455 T1 12 0

9425460 T2 19 0

9425696 T1 3 0

9427746 T3 5 4

9427751 T1 11 0

9428355 T1 25 0

9428547 T1 9 0

9428886 T1 18 0

9428922 T1 4 0

9428934 T2 4 0

9428938 T1 4 0

9429070 T1 9 0

9429372 T1 5 0

9430411 T1 45 0

9430412 T1 4 1

9430518 T1 8 0

9431106 T1 5 3

9432363 T1 7 0

9434061 T1 10 0

9434070 T1 4 0

9434434 T1 18 1

9434610 T1 8 0

9435264 T1 6 0

9435664 T1 2 0

9436188 T1 1 0

9436669 T1 4 0

9437796 T1 1 0

9438968 T1 19 0

9441283 T1 4 1

9441583 T3 2 0

9442343 T2 8 1

9442351 T1 2 0

9442553 T1 1 0

9442733 T1 9 0

9442914 T1 3 3

9443612 T1 2 0

9443616 T1 10 0

9443810 T2 18 0

9443879 T1 10 0

9444178 T1 3 0

9444768 T1 2 0

9445087 T1 17 0

9446362 T1 1 0

9446395 T1 24 0

9447084 T2 8 0

9447087 T1 3 0

9447088 T1 6 0

9447098 T1 4 0

9447100 T1 7 0

9448413 T1 2 0

9448763 T1 32 0

9448874 T1 2 0

9449169 T1 3 0

9449491 T1 7 0

9450338 T1 3 0

9450927 T2 19 0

9451128 T1 2 0

9451143 T1 6 0

9451671 T1 1 0

9451799 T1 10 0

9453004 T2 15 3

9453066 T1 7 0

9453171 T1 10 0

9453624 T3 2 0

9453758 T1 6 0

9453994 T1 5 0

9456253 T1 1 0

9457161 T3 6 0

9457321 T1 6 0

9457360 T1 2 0

9457881 T1 11 0

9457930 T1 3 0

9457988 T4 15 0

9458546 T1 3 0

9458639 T1 7 0

9460193 T1 10 0

9460227 T2 27 0

9463310 T1 10 0

9463438 T2 7 0

9463747 T2 10 0

9463782 T1 2 0

9465309 T1 2 0

9466547 T1 4 0

9466677 T1 2 1

9466794 T1 2 0

9467391 T2 14 0

9467554 T1 22 0

9467555 T3 8 0

9467997 T1 36 0

9468901 T1 3 0

9470077 T1 3 0

9472539 T1 14 0

9472542 T1 7 0

9473348 T1 4 0

9473354 T2 5 0

9473781 T1 8 0

9473789 T1 17 0

9474628 T1 11 0

9475669 T1 9 0

9475925 T1 13 0

9479867 T1 3 0

9480023 T1 2 0

9480278 T1 8 0

9480433 T1 11 0

9480524 T1 3 0

9480896 T3 2 0

9481179 T1 3 0

9481181 T2 6 0

9481242 T1 2 0

9481511 T1 7 0

9481889 T1 2 0

9481984 T1 7 0

9482118 T2 1 0

9482575 T2 13 0

9483150 T1 3 0

9483889 T1 2 0

9483891 T1 27 0

9484339 T1 13 0

9484346 T3 16 1

9484392 T1 40 0

9484830 T1 11 0

9485036 T2 17 0

9487969 T1 5 0

9488273 T1 28 0

9488952 T1 5 0

9488955 T1 1 0

9488966 T1 12 0

9488983 T1 11 0

9489696 T1 17 0

9489938 T1 2 0

9491070 T2 8 1

9491198 T1 4 0

9493470 T1 7 0

9493471 T1 6 0

9493740 T1 9 0

9494007 T1 8 0

9494101 T1 3 0

9495198 T1 6 0

9495211 T1 3 0

9496303 T1 35 0

9496745 T1 7 0

9496754 T1 2 0

9497050 T1 3 0

9497051 T1 8 0

9497312 T1 18 0

9497322 T1 27 0

9498736 T1 3 0

9498811 T1 3 0

9498917 T1 23 0

9501833 T1 6 0

9502267 T1 2 0

9502337 T1 16 0

9502342 T1 4 0

9504074 T2 1 0

9504558 T1 4 0

9504561 T1 5 0

9504563 T1 2 0

9504737 T1 8 0

9504878 T1 9 0

9504879 T1 4 0

9506959 T1 4 0

9506979 T1 10 0

9507274 T1 4 0

9507710 T1 9 0

9507714 T1 7 0

9507905 T1 5 0

9509602 T2 4 0

9509795 T1 9 0

9511622 T1 3 0

9511626 T1 4 0

9511648 T1 1 0

9511654 T1 1 0

9511918 T1 2 1

9511919 T1 6 0

9511922 T1 4 0

9512203 T1 17 0

9512663 T1 9 0

9513734 T1 6 0

9513889 T1 2 0

9514147 T1 5 0

9514314 T1 6 0

9516022 T1 4 0

9516096 T1 1 0

9519351 T1 6 0

9520183 T1 5 0

9520413 T2 14 0

9520534 T1 13 0

9520537 T1 6 0

9520931 T1 30 0

9520939 T1 6 0

9521021 T1 10 0

9522690 T1 1 0

9523374 T1 1 0

9523713 T1 6 0

9525718 T1 3 0

9526632 T1 1 0

9527206 T1 25 0

9527268 T1 6 0

9527458 T1 21 0

9527476 T1 15 0

9527859 T1 21 0

9528249 T1 1 0

9528259 T1 2 0

9528265 T1 7 0

9528284 T1 9 0

9528387 T1 1 0

9529545 T1 10 0

9529550 T2 16 0

9529667 T1 3 0

9529699 T1 12 0

9531307 T1 15 0

9531321 T2 1 0

9532604 T1 5 0

9533079 T1 6 0

9533667 T1 25 0

9534336 T1 11 0

9534365 T1 8 0

9535128 T1 4 0

9535258 T1 7 0

9535261 T1 7 0

9535364 T1 2 0

9535396 T2 16 1

9536081 T1 3 0

9538112 T1 11 0

9538145 T4 15 0

9540664 T1 5 0

9541260 T1 4 0

9541263 T1 1 0

9546221 T2 8 0

9546244 T1 1 0

9546256 T1 3 1

9546635 T1 5 0

9547203 T1 18 0

9547206 T3 17 0

9547269 T1 8 0

10500011 T1 5 0

10501041 T1 7 0

10501068 T1 1 0

10501069 T2 7 0

10501072 T1 6 0

10501079 T1 2 0

10501083 T1 2 0

10501093 T1 17 0

10501099 T1 17 0

10501679 T2 2 0

10501872 T1 4 1

10502388 T3 13 0

10504320 T1 8 0

10504428 T1 20 0

10504822 T1 11 0

10504823 T1 7 0

10505003 T1 2 0

10505689 T4 21 0

10506477 T2 5 0

10506851 T1 7 0

10507087 T1 3 0

10509216 T1 18 0

10510180 T1 7 0

10510182 T3 16 0

10510721 T1 13 0

10510764 T2 8 2

10511341 T2 11 0

10511485 T1 4 0

10511513 T1 15 0

10513410 T1 13 1

10513865 T1 6 0

10514180 T2 61 0

10514181 T1 10 0

10514414 T1 2 0

10514427 T1 4 0

10514430 T1 2 0

10514507 T1 3 0

10514508 T1 4 0

10514533 T1 4 0

10514582 T2 26 0

10515034 T1 9 0

10515075 T1 4 0

10515222 T2 12 0

10517399 T1 5 0

10518030 T2 6 0

10519033 T1 27 0

10519166 T1 3 0

10519272 T1 6 0

10519440 T1 1 0

10519779 T1 2 0

10519841 T1 3 0

10519843 T1 13 0

10519891 T1 3 0

10520411 T2 18 0

10520800 T1 20 0

10520832 T1 11 0

10520835 T2 12 0

10521321 T1 21 0

10521328 T2 13 0

10521374 T1 10 0

10521471 T2 25 0

10521784 T1 8 0

10521789 T1 13 0

10521799 T1 21 0

10521804 T1 10 1

10522054 T3 7 0

10522064 T1 11 0

10522069 T1 14 0

10522544 T1 1 0

10522613 T1 10 0

10522656 T1 9 0

10524947 T1 4 0

10524964 T1 21 0

10525806 T2 6 0

10525880 T3 3 0

10525912 T3 8 0

10525913 T1 26 0

10527132 T1 5 0

10527646 T1 7 0

10527649 T2 3 0

10528241 T1 16 0

10528878 T1 1 0

10528895 T1 22 0

10528905 T1 16 0

10528915 T1 12 0

10531377 T1 5 0

10531386 T2 1 0

10531395 T1 3 0

10531400 T1 4 0

10532090 T2 3 0

10532334 T2 27 0

10532439 T3 4 0

10532442 T1 10 0

10534114 T1 5 0

10534216 T1 12 0

10534402 T1 1 0

10535240 T1 4 0

10535740 T1 11 0

10535982 T1 15 0

10536667 T1 22 0

10537048 T2 1 0

10537250 T1 13 0

10537496 T1 4 0

10541354 T2 16 0

10541652 T2 12 0

10541827 T1 37 0

10541940 T1 15 0

10542010 T1 17 0

10542065 T1 1 0

10542315 T1 5 0

10542377 T1 3 0

10542749 T1 5 0

10543026 T1 14 0

10543082 T1 17 0

10543594 T3 7 0

10544146 T1 10 0

10544176 T3 30 0

10545170 T2 23 2

10545596 T1 19 0

10547099 T2 4 0

10547128 T1 28 0

10547184 T1 14 0

10547274 T1 3 0

10547862 T1 5 0

10548690 T1 12 0

10549046 T1 13 0

10549122 T1 11 0

10550490 T1 17 0

10551161 T2 6 0

10552044 T2 7 0

10552157 T3 8 0

10552592 T1 14 0

10552696 T1 7 0

10552731 T1 15 0

10552784 T1 17 0

10552885 T1 23 0

10553037 T1 5 0

10553040 T1 15 0

10553073 T1 6 0

10553076 T1 3 0

10553209 T1 3 0

10553584 T1 8 0

10554996 T1 5 0

10555396 T1 5 0

10555521 T1 2 0

10556065 T1 10 1

10556684 T1 3 0

10558060 T1 12 0

10558117 T1 10 0

10558189 T1 8 0

10558511 T1 3 0

10558895 T1 33 0

10559006 T1 4 0

10559155 T1 5 0

10559206 T1 23 0

10559246 T1 15 0

10559266 T1 8 0

10559359 T1 9 0

10559362 T1 19 0

10559458 T1 12 0

10559840 T1 17 0

10560773 T1 7 0

10560916 T1 3 0

10560961 T1 19 0

10561085 T1 24 0

10561145 T1 25 1

10561164 T1 1 0

10561199 T1 10 0

10561208 T1 2 0

10561688 T1 1 0

10561876 T1 10 0

10561953 T2 9 0

10562702 T1 1 0

10562927 T1 5 0

10563062 T1 21 0

10563095 T1 2 0

10563138 T1 2 0

10564453 T1 32 0

10564857 T1 20 0

10564934 T1 41 0

10565387 T1 2 0

10565446 T1 20 0

10565864 T1 20 0

10565985 T1 10 0

10566333 T1 16 0

10568077 T1 3 0

10568079 T1 3 0

10568399 T2 6 0

10568524 T1 1 0

10568558 T2 2 0

10568592 T2 2 0

10572147 T2 11 0

10572235 T1 2 0

10574497 T1 4 0

10574786 T2 1 1

10574923 T1 31 0

10575544 T1 2 0

10576370 T2 6 0

10576570 T1 5 0

10576614 T1 10 0

10576652 T1 12 0

10577789 T1 22 0

10580192 T1 3 0

10581267 T1 8 0

10581781 T2 15 0

10582679 T3 50 8

10582705 T1 9 0

10582730 T2 12 0

10582745 T1 19 0

10582804 T1 11 0

10583139 T1 9 0

10583218 T1 21 0

10583448 T1 23 0

10586248 T1 3 0

10586811 T3 13 0

10587148 T1 23 0

10587211 T1 14 0

10587259 T3 25 0

10587299 T1 11 0

10587331 T1 21 0

10587339 T1 10 0

10587546 T1 2 0

10587646 T3 20 0

10588610 T1 9 0

10589469 T1 11 0

10589654 T1 5 0

10589822 T2 14 0

10589941 T1 15 0

10590488 T1 11 0

10590813 T1 1 0

10590868 T1 12 0

10591290 T2 2 0

10591635 T1 1 0

10592144 T1 18 0

10592234 T1 5 0

10592274 T1 9 0

10592886 T2 16 0

10595046 T1 2 2

10595331 T1 20 0

10595520 T1 4 0

10595914 T2 2 0

10596376 T1 3 0

10597631 T2 5 0

10597971 T1 6 0

10598395 T1 18 0

10598430 T1 25 0

10598454 T1 13 0

10598723 T1 31 0

10598799 T2 19 0

10598817 T2 28 0

10598851 T1 10 0

10598907 T1 17 0

10599106 T3 28 0

10599216 T1 26 0

10599259 T1 36 0

10599275 T1 14 0

10599280 T2 11 0

10599332 T1 19 0

10599386 T1 37 0

10599992 T1 11 0

10600591 T1 5 0

10600842 T1 5 0

10601065 T1 29 0

10601183 T1 1 0

10601189 T1 11 0

10602071 T1 10 0

10602111 T1 19 0

10602173 T2 22 4

10602432 T1 5 0

10602574 T2 4 0

10602673 T1 5 0

10602730 T2 6 0

10603240 T1 4 0

10603482 T1 6 0

10603794 T1 7 0

10604899 T1 21 0

10605382 T1 4 0

10605776 T1 2 0

10606151 T1 10 0

10606466 T3 29 0

10606513 T1 31 0

10606606 T2 10 0

10606736 T2 12 0

10606748 T1 2 0

10606775 T1 22 0

10606884 T1 12 0

10606952 T1 26 0

10607016 T1 7 0

10607033 T1 10 0

10607038 T1 24 0

10607266 T1 4 0

10607369 T1 5 0

10607473 T1 23 0

10607500 T1 15 0

10608691 T2 5 0

10608913 T1 5 0

10609143 T1 22 0

10609200 T2 3 0

10609215 T1 4 0

10609218 T1 11 0

10609573 T1 14 0

10609896 T1 13 0

10610196 T1 11 0

10610306 T1 4 0

10612086 T1 16 0

10612123 T1 10 0

10612239 T2 8 0

10612452 T1 20 0

10612456 T1 24 0

10614046 T1 7 0

10614311 T1 7 0

10614630 T1 14 0

10614892 T1 13 0

10615039 T1 13 0

10615930 T3 8 0

10616998 T1 13 0

10617085 T1 35 0

10617183 T1 10 0

10617385 T1 5 1

10617684 T2 22 0

10618055 T3 21 0

10618762 T1 9 0

10618789 T1 18 0

10619117 T4 8 0

10619354 T1 10 0

10619377 T1 12 0

10619496 T1 1 0

10620664 T1 19 0

10620708 T2 18 0

10621901 T1 11 0

10621985 T1 11 0

10623397 T1 1 0

10623950 T3 7 0

10624217 T1 10 0

10624867 T1 33 0

10624927 T1 24 0

10625299 T1 1 0

10625906 T1 31 0

10625913 T1 15 0

10626191 T1 16 0

10626555 T1 7 0

10626698 T1 7 0

10627030 T3 7 4

10627453 T1 17 0

10628594 T1 4 0

10629280 T1 7 0

10629796 T1 4 0

10629847 T1 1 0

10629924 T1 22 0

10630007 T1 16 0

10630307 T1 1 0

10630335 T3 17 0

10630381 T1 9 0

10630464 T1 14 0

10630480 T1 14 0

10630495 T1 7 0

10630527 T1 13 0

10630616 T1 9 0

10630947 T1 19 0

10630975 T1 16 0

10631085 T1 11 0

10631184 T1 16 0

10631338 T1 35 0

10631417 T2 13 0

10631451 T1 27 0

10631533 T2 25 0

10631552 T1 10 4

10631589 T1 14 0

10631677 T1 26 0

10631680 T2 13 0

10632315 T1 11 0

10632561 T1 1 0

10632865 T1 12 0

10634162 T1 22 0

10635389 T1 3 0

10635403 T1 20 0

10635714 T2 18 0

10635746 T1 12 0

10636006 T2 18 8

10639122 T1 17 0

10639164 T1 6 0

10639388 T1 6 0

10641416 T1 10 0

10642723 T1 24 0

10642919 T1 17 0

10643145 T1 6 0

10643157 T1 6 0

10644617 T1 16 0

10644713 T1 13 0

10644949 T1 12 0

10645121 T1 24 0

10645260 T1 26 0

10645271 T1 14 0

10645284 T3 17 0

10645307 T1 14 0

10645342 T1 22 0

10645482 T1 14 0

10645781 T1 8 0

10645917 T2 8 0

10646162 T1 1 0

10646365 T1 1 0

10646665 T1 13 0

10646689 T3 33 0

10646819 T1 14 0

10648312 T1 2 0

10649204 T1 5 0

10649454 T3 2 0

10650088 T2 15 0

10650352 T1 17 0

10650719 T2 13 0

10650817 T1 10 0

10650856 T1 7 0

10651048 T1 9 0

10651081 T3 8 0

10653459 T1 12 0

10653856 T1 20 0

10654288 T1 11 0

10654359 T1 13 0

10654682 T1 42 0

10654712 T1 5 0

10655040 T1 39 0

10655376 T1 7 4

10655403 T1 17 0

10655789 T1 1 0

10655804 T1 13 0

10656252 T1 19 0

10656255 T2 13 0

10656445 T1 6 0

10656506 T3 16 0

10656526 T1 5 0

10656744 T1 6 0

10656834 T2 10 0

10656867 T4 17 0

10656893 T1 7 0

10656972 T1 18 0

10657199 T1 5 0

10657223 T3 32 0

10657241 T1 27 0

10657289 T3 25 0

10657325 T1 16 0

10657689 T1 10 0

10657980 T2 36 0

10659224 T1 14 0

10659529 T1 2 0

10659675 T1 12 0

10659810 T1 16 0

10660662 T2 20 0

10660762 T1 16 0

10660765 T1 25 0

10660774 T2 39 0

10660939 T1 29 0

10661252 T3 24 0

10661848 T1 26 0

10662817 T3 6 0

10663273 T2 10 0

10663856 T1 11 0

10664212 T1 1 0

10664534 T2 16 0

10664589 T1 9 0

10664652 T1 25 0

10665033 T1 10 1

10665115 T1 11 0

10665176 T1 1 0

10665191 T1 2 0

10665281 T1 2 1

10665316 T3 5 1

10666900 T1 22 0

10667255 T1 8 0

10667370 T1 24 0

10667430 T1 10 0

10668282 T2 10 0

10668993 T1 10 0

10669060 T2 23 0

10669336 T1 3 0

10669352 T1 13 0

10669373 T1 21 0

10669396 T1 11 0

10669699 T1 26 0

10670648 T1 12 0

10670946 T1 2 0

10670957 T1 2 0

10671163 T2 4 0

10671505 T1 18 0

10672060 T1 10 0

10672157 T1 14 0

10672318 T1 19 0

10672455 T2 14 0

10672510 T1 26 0

10672522 T1 26 0

10672584 T1 20 0

10672593 T1 19 0

10672696 T1 14 0

10672709 T1 41 0

10672878 T1 8 0

10672923 T1 26 0

10672944 T1 42 1

10673113 T4 13 0

10673115 T1 11 0

10673176 T1 1 0

10673502 T2 8 0

10673581 T1 19 0

10673610 T1 21 0

10673837 T1 12 0

10673960 T3 22 0

10674065 T1 15 0

10674216 T1 14 0

10674306 T1 21 0

10674478 T1 8 0

10674824 T2 16 0

10674908 T2 16 0

10674952 T1 22 0

10675011 T1 2 0

10675337 T4 20 0

10675355 T1 13 0

10675661 T2 7 0

10675713 T1 19 0

10675842 T3 21 1

10675894 T1 11 0

10676366 T2 12 0

10676642 T2 2 0

10676686 T1 5 0

10676894 T1 9 0

10677949 T1 18 0

10677965 T1 22 0

10677968 T1 24 0

10678148 T1 23 0

10678162 T1 20 0

10678222 T1 14 0

10678223 T1 10 0

10678693 T1 14 0

10678703 T2 12 0

10679192 T2 38 0

10679219 T1 21 0

10679686 T1 11 0

10679764 T1 28 0

10680303 T1 21 0

10680465 T1 17 0

10680473 T1 6 0

10680744 T1 20 1

10680822 T1 16 0

10680964 T1 7 0

10680983 T1 4 0

10680998 T1 10 0

10681131 T1 9 5

10681288 T1 7 0

10681317 T1 7 0

10681529 T1 9 0

10681853 T2 28 1

10681928 T2 8 0

10682033 T1 14 0

10682061 T1 29 0

10682561 T1 12 0

10682615 T1 19 0

10683429 T1 3 0

10683681 T1 1 0

10683993 T1 6 0

10684047 T1 7 0

10684348 T1 9 0

10684360 T1 9 0

10684893 T1 4 0

10686580 T1 5 0

10687254 T1 19 0

10687513 T1 13 0

10687735 T1 10 0

10687814 T1 3 0

10687861 T1 4 1

10687997 T1 4 0

10688272 T1 8 0

10688654 T1 1 0

10688886 T1 6 0

10689183 T1 1 0

10689317 T1 10 0

10690066 T1 15 0

10690193 T1 21 0

10690219 T3 8 1

10690283 T1 14 0

10690313 T1 8 0

10690333 T1 17 0

10690552 T1 12 0

10691044 T1 12 0

10691368 T1 15 0

10691482 T2 45 0

10691600 T1 21 0

10691609 T1 39 0

10691620 T1 15 0

10692151 T2 35 0

10692162 T3 18 0

10692195 T1 14 0

10692635 T2 37 0

10695081 T1 7 0

10695336 T3 14 0

10695340 T1 8 0

10695487 T1 33 0

10695626 T1 32 0

10695743 T3 43 0

10695812 T1 22 0

10695817 T1 35 0

10696061 T1 2 0

10696099 T1 22 0

10696175 T2 16 0

10696198 T1 22 0

10696204 T1 25 0

10696261 T1 22 0

10696497 T2 29 0

10696526 T3 20 0

10696550 T1 28 0

10696623 T1 18 0

10696635 T1 26 0

10696641 T1 29 0

10696718 T1 21 0

10697264 T1 13 0

10697283 T1 22 0

10697351 T1 26 0

10697429 T3 16 2

10697570 T1 3 0

10697885 T3 34 5

10698232 T1 8 0

10698247 T1 21 0

10699405 T3 9 0

10699713 T3 14 0

10699774 T1 12 0

10699848 T1 8 0

10700434 T1 21 0

10700535 T1 24 0

10700659 T1 8 0

10700716 T2 11 0

10701255 T1 37 0

10701379 T2 7 0

10701885 T1 14 0

10702384 T3 26 4

10702539 T2 25 2

10702556 T1 26 0

10702695 T1 25 0

10702698 T1 13 0

10702823 T1 31 0

10702895 T1 6 0

10702902 T1 14 0

10703005 T1 22 0

10703110 T1 15 0

10703214 T1 6 0

10704101 T1 12 0

10704135 T1 20 0

10704698 T1 7 0

10704924 T1 18 0

10705691 T3 9 0

10705710 T1 3 0

10706100 T2 25 0

10706150 T1 38 0

10706779 T1 17 0

10706865 T2 6 0

10706959 T1 2 0

10707030 T1 12 0

10707046 T1 10 0

10707364 T1 15 0

10707384 T1 27 0

10707475 T1 14 0

10707720 T1 15 0

10707763 T1 3 0

10707911 T2 3 0

10707942 T1 13 0

10708032 T1 16 0

10708103 T1 2 0

10708178 T1 15 0

10708248 T3 2 0

10708259 T1 1 0

10708588 T1 29 0

10708591 T1 35 0

10708601 T1 5 0

10708675 T3 25 0

10708751 T1 29 0

10708780 T1 10 0

10708806 T2 17 0

10708913 T2 25 0

10709959 T1 13 0

10710337 T3 8 0

10710551 T1 7 0

10710557 T1 25 0

10710561 T1 22 0

10710564 T1 16 0

10710570 T3 12 0

10710872 T1 3 0

10711104 T1 14 0

10711224 T1 9 0

10711662 T1 24 0

10712410 T2 5 0

10712664 T1 8 0

10712856 T3 17 0

10713171 T1 2 0

10713328 T1 2 0

10713483 T1 49 5

10713577 T1 1 0

10713725 T1 23 0

10713921 T2 16 0

10714182 T1 1 0

10714616 T1 9 0

10715021 T1 11 0

10715068 T1 10 0

10715155 T1 10 0

10715250 T1 20 0

10715365 T3 18 0

10715476 T3 14 0

10715538 T1 41 0

10715702 T1 7 0

11667393 T3 33 0

11667625 T2 5 0

11668047 T3 3 0

11668054 T2 1 0

11668196 T1 7 0

11668381 T2 7 1

11668509 T1 28 0

11668560 T2 33 0

11668593 T1 12 0

11668985 T1 12 0

11669037 T1 71 0

11669086 T1 17 0

11669194 T1 16 0

11669195 T1 47 0

11670548 T1 9 0

11670579 T1 11 1

11670619 T1 3 0

11670709 T1 11 0

11670877 T1 33 0

11671092 T1 20 0

11671420 T1 18 0

11671475 T1 8 0

11671484 T1 23 0

11671753 T1 18 0

11671764 T1 26 0

11672398 T1 5 0

11672416 T1 11 0

11672607 T1 4 0

11672965 T2 15 0

11673072 T1 15 0

11673103 T2 21 0

11673112 T3 21 0

11673168 T1 12 0

11673178 T1 29 0

11673331 T3 16 0

11673482 T4 24 1

11673909 T1 8 0

11674038 T1 24 0

11674044 T1 45 0

11674157 T1 28 0

11674286 T1 20 0

11674364 T1 25 0

11674579 T1 21 0

11674648 T3 18 0

11675157 T1 23 0

11675220 T1 26 0

11675837 T1 31 0

11675935 T1 2 0

11676075 T1 12 0

11676544 T1 23 0

11676566 T1 29 0

11676728 T1 10 0

11676791 T2 5 0

11676936 T1 25 2

11677563 T1 10 0

11677976 T1 18 0

11678041 T1 18 0

11678049 T1 23 1

11678191 T2 28 0

11678232 T2 17 0

11678252 T1 29 0

11678413 T1 20 0

11678448 T3 33 5

11678489 T1 21 0

11678494 T1 27 0

11678540 T1 11 0

11678635 T1 11 0

11678644 T1 21 0

11678784 T2 12 0

11678791 T1 16 0

11678892 T1 24 0

11679017 T1 21 0

11679165 T1 19 0

11679274 T1 24 0

11679469 T3 19 0

11679471 T1 36 0

11679567 T3 15 1

11679621 T2 15 0

11679646 T1 15 0

11679661 T1 10 0

11679904 T1 13 0

11680012 T1 17 0

11680433 T1 16 0

11680751 T1 1 0

11681098 T1 23 0

11681407 T1 7 0

11681437 T2 16 0

11681971 T1 1 0

11681983 T1 14 0

11682065 T3 3 0

11682527 T1 7 0

11682838 T1 17 0

11683188 T2 15 0

11683511 T1 29 0

11683518 T3 12 0

11683532 T1 21 0

11683645 T1 12 0

11683687 T1 5 0

11684504 T1 22 0

11684532 T1 11 0

11685066 T1 32 0

11685496 T1 20 0

11685580 T1 3 0

11686003 T1 55 0

11686162 T1 22 0

11686210 T1 22 0

11686387 T1 34 0

11686398 T1 29 0

11687195 T1 19 0

11687215 T2 34 0

11687240 T4 19 1

11687371 T2 31 1

11687522 T3 42 0

11687545 T1 13 0

11687574 T1 10 0

11687598 T1 14 0

11687602 T1 49 0

11687607 T1 26 0

11687659 T1 17 0

11688101 T1 4 0

11688431 T1 6 0

11688524 T1 20 0

11688876 T1 21 0

11689641 T1 16 0

11689992 T2 12 0

11690847 T1 18 0

11690857 T1 39 0

11691024 T3 23 0

11691388 T1 17 0

11691989 T1 10 0

11692706 T2 4 0

11692767 T1 22 0

11692950 T1 18 0

11693111 T1 7 0

11693125 T1 1 0

11693833 T3 19 0

11693840 T1 29 0

11693847 T1 25 0

11695597 T1 27 0

11698046 T1 18 0

11698183 T1 40 0

11698543 T1 22 0

11704017 T1 10 0

11706166 T1 17 0

11706194 T1 39 0

11706223 T1 13 0

11706845 T1 8 0

11706881 T1 10 0

11706901 T2 1 0

11707277 T1 4 0

11707286 T1 34 0

11707370 T1 7 0

11707822 T1 16 0

11708030 T1 5 0

11708083 T1 8 0

11708158 T3 6 0

11708190 T1 13 0

11708213 T1 1 0

11708228 T2 24 0

11708644 T1 18 0

11708665 T1 5 0

11708897 T1 19 0

11708907 T1 8 0

11709116 T1 3 0

11709234 T4 5 0

11709669 T1 17 0

11709884 T1 2 0

11710236 T3 11 2

11710288 T1 12 0

11710462 T1 10 0

11710517 T1 20 0

11710568 T2 16 0

11710637 T1 20 0

11710672 T1 24 0

11710698 T1 9 0

11710733 T3 16 0

11710765 T1 2 0

11710887 T1 25 3

11711075 T1 6 0

11711099 T1 20 0

11711171 T2 15 0

11711174 T1 12 0

11711438 T1 5 0

11711765 T1 1 0

11711786 T1 18 0

11712108 T1 15 1

11712711 T1 21 0

11713040 T1 1 0

11713102 T1 21 0

11713118 T1 31 0

11713561 T2 14 0

11713595 T1 45 0

11713672 T1 21 0

11713812 T1 7 0

11714179 T3 11 0

11714616 T1 4 0

11714622 T1 9 0

11714781 T1 20 0

11714910 T1 14 0

11715399 T3 31 0

11715420 T1 34 0

11715713 T1 14 1

11716004 T1 17 0

11716249 T1 27 0

11716474 T2 1 0

11716993 T1 9 0

11717062 T1 3 0

11717201 T1 1 0

11717246 T1 29 0

11717256 T2 25 0

11717284 T1 11 0

11717426 T1 11 0

11717961 T1 10 1

11718418 T3 12 0

11718745 T1 12 0

11719050 T1 17 0

11719286 T1 10 0

11719335 T3 16 0

11719372 T1 18 0

11719417 T1 23 0

11719881 T1 27 0

11720167 T1 1 0

11720196 T1 16 0

11720198 T2 11 0

11720692 T1 13 0

11720711 T1 16 2

11720899 T1 39 0

11720978 T1 4 0

11721168 T1 23 0

11721402 T3 15 0

11721635 T1 12 0

11721767 T1 17 0

11721885 T1 61 0

11722201 T1 19 0

11722527 T1 4 0

11722554 T1 10 0

11722738 T1 11 0

11723027 T1 29 0

11723036 T1 28 0

11723459 T2 22 0

11723546 T1 32 0

11723609 T1 19 0

11723787 T1 28 0

11723789 T1 19 0

11723992 T1 45 0

11723999 T1 25 0

11724095 T1 23 0

11724121 T3 18 0

11724201 T3 60 0

11724217 T3 21 0

11724272 T1 24 0

11724273 T1 11 0

11724331 T1 43 0

11724417 T1 8 0

11724426 T1 32 0

11724447 T1 26 0

11724545 T1 22 0

11724664 T1 25 0

11724685 T1 37 0

11724702 T1 28 0

11724738 T1 25 0

11724744 T2 29 0

11724846 T1 20 0

11724944 T1 30 0

11725089 T2 9 0

11725241 T1 25 0

11725412 T3 22 3

11725422 T1 26 0

11725564 T1 28 0

11725621 T1 7 0

11725652 T1 39 0

11725658 T1 12 0

11725662 T1 28 0

11725855 T1 15 0

11725884 T1 22 0

11725994 T2 11 0

11725996 T1 29 0

11726062 T1 19 0

11726072 T1 27 0

11726318 T1 4 0

11726340 T1 20 0

11726343 T1 42 0

11726389 T1 27 0

11726584 T2 18 0

11726980 T2 7 0

11727045 T1 16 0

11727349 T3 35 0

11727517 T3 16 0

11727918 T1 23 0

11727942 T1 29 1

11728032 T1 14 0

11728064 T3 24 0

11728302 T1 23 0

11728479 T1 6 0

11728593 T1 3 0

11728600 T1 22 0

11728664 T1 36 0

11728815 T1 17 0

11728940 T1 8 0

11729176 T1 25 0

11729257 T1 34 0

11729427 T3 15 2

11729649 T1 21 0

11733433 T1 47 0

11733435 T1 5 0

11733790 T1 34 0

11737047 T1 10 0

11737428 T1 4 0

11737501 T1 15 0

11784782 T1 26 0

13052878 T2 13 0

14004936 T1 18 0

14118508 T1 8 0

14144982 T1 25 0

14174593 T1 8 0

14201839 T1 13 0

14202118 T3 11 1

15353189 T1 3 0

15360143 T3 22 0

15362155 T2 1 0

15364926 T1 2 0

15365253 T1 8 0

15367072 T1 1 0

15369354 T1 14 0

15371049 T1 4 0

15371465 T2 2 0

15373122 T1 1 0

15373508 T2 4 0

15374023 T1 24 0

15376907 T1 13 0

15377631 T1 1 0

15378027 T1 4 0

15378259 T2 4 0

15379861 T1 7 0

15379924 T1 4 0

15379944 T1 5 0

15380867 T2 6 0

15381273 T1 4 0

15382045 T1 3 0

15382263 T3 4 0

15382413 T2 1 0

15382415 T3 1 0

15383494 T1 9 0

15383949 T2 7 0

15384266 T1 9 0

15384886 T2 3 0

15385047 T1 2 0

15386164 T1 8 0

15386651 T1 6 0

15389560 T1 5 0

15390194 T3 10 2

15390457 T2 3 0

15391829 T1 2 0

15391834 T1 2 0

15392024 T1 4 0

15392563 T1 2 0

15393832 T1 23 0

15394591 T1 10 0

15396244 T1 1 0

15396298 T1 1 0

15396326 T1 2 0

15396358 T1 3 0

15397325 T2 2 0

15397598 T1 10 0

15399487 T1 1 0

15399582 T1 2 0

15400084 T1 11 0

15400099 T1 2 0

15400291 T1 2 0

15400319 T1 7 0

15400997 T1 3 0

15401423 T1 1 0

15405150 T1 11 0

15405167 T1 20 0

15405409 T1 3 0

15407335 T1 9 0

15408205 T1 8 0

15408334 T1 5 0

15408917 T1 19 0

15408938 T1 3 0

15408982 T1 11 0

15409200 T1 2 0

15409626 T1 1 0

15409715 T1 4 0

15411486 T1 6 0

15412664 T1 36 0

15412748 T2 5 0

15412750 T1 1 0

16333461 T1 1 0

16333468 T1 11 0

16333508 T1 2 0

16333551 T2 9 0

16333566 T1 4 0

16334533 T1 14 0

16334551 T2 18 0

16334586 T1 4 0

16335949 T1 33 0

16337448 T1 2 0

16337882 T1 15 0

16338327 T1 3 2

16338489 T1 3 0

16339767 T2 3 0

16339828 T1 3 0

16340461 T2 7 0

16341297 T1 6 0

16341353 T1 5 0

16342284 T1 5 0

16342290 T1 5 0

16342759 T1 5 0

16343896 T1 7 0

16343899 T1 13 0

16343900 T1 3 0

16344467 T1 5 0

16345449 T1 3 0

16345786 T1 16 0

16345846 T2 4 0

16346052 T2 1 0

16346528 T1 16 0

16346846 T1 9 0

16347189 T1 8 0

16348104 T1 4 0

16348200 T1 22 0

16348226 T1 15 0

16348448 T1 8 0

16349630 T1 1 0

16350215 T2 4 0

16350243 T1 29 0

16350333 T1 8 0

16350356 T1 4 0

16350623 T1 3 0

16350758 T1 1 0

16351157 T1 13 0

16351164 T1 9 0

16351368 T1 5 0

16352089 T1 13 0

16352090 T1 10 0

16352669 T1 14 0

16352685 T1 10 0

16353145 T1 10 0

16353410 T1 13 0

16354407 T1 7 0

16354977 T1 4 0

16355898 T1 2 0

16356719 T1 11 0

16356809 T1 12 0

16357823 T1 28 0

16358392 T2 25 0

16358701 T3 12 0

16359008 T1 2 0

16359342 T1 14 0

16359508 T3 3 1

16359513 T1 19 0

16359575 T1 6 0

16360382 T1 12 0

16360887 T1 10 0

16361448 T1 24 0

16361895 T1 13 0

16362964 T1 10 0

16363096 T1 20 0

16363105 T1 4 0

16363107 T2 13 0

16363114 T1 8 0

16363135 T1 29 6

16364669 T2 3 0

16365157 T1 28 0

16367091 T1 7 0

16367747 T1 25 0

16367750 T1 2 0

16367806 T1 6 0

16367890 T1 6 0

16367900 T1 2 0

16368319 T2 14 0

16369351 T1 5 2

16369359 T1 24 0

16370351 T1 2 0

16370354 T1 10 0

16370564 T1 7 0

16370567 T1 4 0

16370608 T3 6 0

16371317 T1 16 0

16371342 T1 12 0

16371708 T1 2 0

16373158 T1 11 2

16373206 T1 14 0

16373303 T1 2 0

16373317 T1 2 0

16373538 T2 19 0

16373669 T1 1 0

16374036 T1 11 0

16374037 T1 22 0

16374041 T1 9 0

16374563 T1 5 0

16374570 T1 15 0

16374859 T1 4 0

16374864 T2 19 0

16375105 T1 6 0

16375108 T1 8 0

16375478 T1 4 0

16375488 T1 11 0

16375494 T1 7 0

16375500 T1 4 0

16375501 T3 23 1

16376000 T2 17 1

16376004 T1 1 0

16376010 T1 4 0

16376290 T1 16 0

16377482 T1 18 0

16378322 T1 5 0

16378636 T1 5 0

16378836 T1 7 0

16379907 T2 5 1

16379914 T1 20 0

16380128 T1 21 0

16380628 T1 15 0

16380946 T1 7 0

16381954 T1 24 0

16382022 T1 20 0

16382071 T2 17 0

16382244 T2 16 0

16382251 T1 3 0

16382254 T1 1 0

16382268 T1 5 0

16382279 T2 14 0

16382911 T2 6 0

16383336 T2 1 0

16384114 T1 17 0

16384251 T1 6 0

16384915 T1 6 0

16384921 T1 5 0

16385896 T2 15 0

16385945 T1 1 0

16386928 T1 4 0

16386957 T1 5 0

16386961 T1 10 0

16387040 T1 5 0

16387052 T1 11 0

16387543 T1 4 0

16387653 T2 7 0

16388884 T1 3 0

16390663 T1 5 0

16390807 T1 3 0

16390947 T1 4 0

16392088 T1 3 0

16392590 T1 9 0

16393095 T1 5 0

16393247 T2 25 0

16393308 T1 8 0

16394724 T2 2 0

16395279 T1 4 0

16395505 T1 5 0

16396091 T2 23 3

16398541 T1 5 0

16399051 T1 20 0

16399202 T1 3 0

16399660 T1 4 0

16399892 T1 14 0

16399893 T2 2 0

16399898 T1 6 0

16399925 T1 8 0

16400880 T1 37 0

16400899 T1 6 0

16400971 T1 6 0

16400983 T1 10 0

16401368 T1 12 0

16401766 T1 7 0

16401773 T2 24 0

16402722 T1 6 0

16402750 T2 42 0

16402765 T1 7 0

16403137 T1 3 0

16404485 T2 2 0

16406198 T1 3 0

16407224 T2 25 1

16407751 T1 7 0

16407769 T1 5 0

16407802 T2 6 0

16409137 T1 5 0

16409151 T1 6 0

16409171 T1 4 0

16409964 T1 2 0

16410198 T1 9 0

16411392 T2 4 0

16412012 T2 9 0

16412749 T1 12 0

16412838 T1 2 0

16412845 T1 11 0

16413154 T1 14 0

16414526 T1 4 0

16414926 T1 16 0

16415050 T1 5 0

16416057 T1 1 0

16416534 T1 5 0

16417632 T1 12 0

16418383 T1 23 0

16418482 T1 6 0

16418488 T1 14 0

16418915 T1 11 0

16419177 T1 19 0

16421240 T1 9 0

16421704 T1 13 0

16422108 T1 22 0

16422599 T1 3 0

16422606 T2 1 0

16423182 T1 8 0

16423184 T1 6 0

16423193 T2 9 0

16423205 T1 2 0

16423210 T1 1 0

16425069 T2 8 0

16425072 T1 7 0

16425079 T1 8 0

16425805 T1 16 0

16426214 T2 8 0

16426220 T1 11 0

16426227 T1 33 0

16426802 T1 6 0

16426803 T1 7 0

16426813 T1 6 0

16426820 T2 17 0

16427099 T1 8 0

16428119 T3 2 0

16428179 T1 19 0

16428387 T1 20 0

16428389 T1 8 0

16428970 T1 1 0

16428973 T1 10 0

16428977 T1 11 0

16429322 T1 12 0

16429900 T1 2 0

16430085 T1 16 0

16430910 T2 1 0

16430922 T2 11 0

16431607 T1 8 0

16431878 T3 14 2

16432291 T1 4 0

16433033 T3 7 0

16434219 T1 11 0

16434455 T2 2 0

16434459 T1 4 0

16435819 T2 10 0

16436025 T1 7 0

16436051 T1 5 0

16436463 T1 4 0

16436465 T1 20 0

16436471 T1 7 0

16438055 T1 9 0

16438878 T1 10 0

16440108 T2 1 0

16441759 T1 34 0

16441772 T1 8 0

16442143 T1 9 0

16442312 T2 1 0

16442315 T1 29 0

16442323 T1 2 0

16443468 T1 13 0

16443552 T1 5 0

16445074 T1 2 0

16445252 T1 7 0

16445612 T1 11 0

16445620 T1 3 0

16445632 T1 7 0

16446178 T1 6 0

16446182 T1 3 0

16446948 T2 9 0

16446949 T1 9 0

16448018 T1 14 0

16448054 T1 1 0

16448532 T2 9 0

16449036 T1 32 0

16449151 T1 39 0

16449224 T1 7 0

16449245 T3 14 0

16449297 T1 13 0

16449670 T1 11 0

16449676 T1 17 0

16449806 T1 3 0

16450508 T2 7 0

16451111 T1 10 0

16452131 T1 21 0

16452168 T1 1 0

16452772 T1 6 1

16453016 T1 6 0

16453042 T1 13 0

16455044 T1 2 0

16455422 T1 16 0

16455724 T1 23 1

16456137 T2 2 0

16456979 T1 20 0

16456984 T1 9 0

16457075 T2 8 0

16457293 T2 26 2

16457583 T1 1 0

16457601 T1 19 0

16457613 T1 3 0

16458248 T1 10 0

16458267 T1 7 0

16458281 T1 3 0

16458291 T1 42 0

16459178 T1 5 0

16459187 T1 3 0

16460210 T2 9 0

16460240 T1 1 0

16460268 T3 15 0

16460270 T1 9 0

16460306 T1 2 0

16461147 T2 19 0

16461171 T3 5 0

16461651 T1 12 0

16461654 T1 5 0

16461657 T2 21 0

16461846 T1 4 0

16461854 T1 10 0

16461863 T1 13 0

16462541 T1 13 0

16462683 T1 9 0

16463412 T1 1 0

16463418 T1 2 0

16463430 T1 4 0

16464474 T1 1 0

16464483 T1 39 0

16465008 T1 13 0

16465422 T1 6 0

16465750 T1 2 0

16465756 T1 20 0

16465890 T1 12 0

16466032 T1 5 0

16466861 T1 22 0

16466868 T1 2 0

16467568 T1 6 0

16467571 T1 7 0

16467719 T2 52 0

16467722 T1 2 0

16468122 T1 25 0

16468874 T1 17 0

16469402 T1 9 0

16469403 T1 6 0

16469588 T1 9 0

16469769 T2 8 0

16470085 T1 9 0

16470178 T2 10 0

16470197 T3 2 0

16470736 T1 3 0

16470739 T1 12 0

16471464 T1 3 0

16471466 T1 6 0

16472001 T2 11 0

16472650 T2 13 0

16472657 T1 1 0

16473661 T1 13 0

16473663 T1 1 0

16473664 T1 21 0

16473666 T1 25 0

16473669 T1 8 0

16474089 T1 3 0

16474989 T1 4 0

16475440 T1 4 0

16477328 T1 5 0

16478075 T1 5 0

16478494 T1 8 0

16479850 T1 2 0

16480639 T1 14 0

16481281 T1 8 0

16481552 T1 9 0

16481555 T1 2 0

16482138 T2 2 0

16483323 T1 6 5

16483329 T1 8 0

16483332 T1 12 0

16483343 T1 13 0

16484334 T1 9 0

16484747 T1 7 0

16484751 T1 8 0

16485941 T1 9 0

16486134 T1 3 0

16486143 T1 6 0

16486616 T1 11 0

16487825 T2 20 0

16487828 T2 7 0

16487830 T1 1 0

16487832 T1 1 0

16487838 T3 7 0

16488747 T3 4 0

16488750 T1 2 0

16489511 T1 2 0

16489533 T1 10 0

16489901 T1 6 0

16489964 T1 7 0

16490241 T3 21 0

16490273 T1 25 0

16490711 T1 6 0

16490714 T1 7 0

16491808 T1 14 0

16492484 T1 21 0

16492485 T1 21 0

16492927 T1 7 0

16493283 T1 13 0

16493741 T4 1 0

16494175 T1 7 0

16494498 T1 2 0

16495361 T1 1 0

16496395 T1 9 0

16496398 T1 20 0

16496400 T3 2 0

16496402 T1 21 0

16497252 T1 19 0

16497257 T1 1 0

16497260 T1 3 0

16497860 T1 4 0

16497862 T1 7 0

16497868 T1 13 0

16500343 T2 21 0

16500345 T1 5 0

16500352 T1 2 0

16501434 T1 14 0

16501492 T2 8 0

16501494 T1 11 0

16502252 T1 19 0

16502267 T1 15 0

16502934 T1 18 0

16502936 T1 5 0

16503853 T1 18 0

16504414 T1 8 0

16504608 T1 4 0

16506030 T1 50 0

16506692 T1 2 0

16507151 T1 3 0

16507405 T1 12 0

16508245 T2 7 0

16508731 T1 2 0

16508896 T1 19 0

16508900 T1 6 0

16509362 T3 34 0

16509409 T1 3 0

16510259 T1 4 0

16512788 T1 6 5

16513617 T1 3 0

16513623 T1 3 0

16513629 T1 16 0

16514693 T2 10 0

16514904 T1 4 0

16515733 T1 11 0

16516046 T1 7 0

16516049 T1 3 0

16516060 T1 16 0

16517412 T2 13 0

16519080 T1 6 0

16519266 T1 3 0

16519373 T1 4 0

16520130 T2 1 0

16521975 T2 29 0

16522935 T1 5 0

16523737 T3 6 0

16524926 T1 2 0

16524930 T1 4 0

16525424 T1 9 0

16525627 T1 12 0

16525635 T1 34 0

16525660 T1 1 0

16526101 T1 22 0

16527598 T1 7 0

16527721 T1 5 0

16528538 T1 9 0

16528747 T1 1 0

16530185 T1 12 0

16530744 T1 18 0

16531105 T1 20 0

16532188 T1 22 0

16532368 T1 10 0

16534046 T1 16 0

16534059 T1 11 0

16534066 T1 11 0

16534668 T1 6 0

16535352 T1 9 0

16535515 T1 17 0

16535537 T1 12 0

16536855 T1 9 0

16537475 T1 9 0

16538451 T1 38 0

16538453 T1 5 0

16538455 T1 8 0

16538484 T1 5 0

16539718 T2 24 0

16541131 T1 2 0

16541135 T1 3 0

16541739 T1 7 0

16541740 T1 4 0

16541747 T1 17 0

16542659 T1 7 0

16542864 T1 1 0

16543032 T1 9 0

16543675 T2 6 1

16544429 T1 13 0

16544432 T1 6 0

16544615 T1 19 0

16544836 T1 1 0

16544840 T1 16 0

16544851 T1 24 0

16545129 T2 10 0

16546026 T2 24 0

16546403 T1 15 0

16548061 T1 15 0

16548064 T1 13 0

16548065 T1 25 0

16548164 T2 9 6

16548530 T1 7 0

16549547 T1 24 1

16549836 T1 6 0

16549942 T1 10 0

16550875 T1 18 0

16551019 T3 3 0

16551384 T1 5 1

16551415 T1 18 0

16551700 T1 11 0

16552770 T3 13 0

16552772 T1 1 0

16552773 T1 24 0

16552819 T1 4 0

16554295 T2 13 0

16554297 T1 10 0

16554466 T1 1 0

16555574 T2 4 0

16555643 T2 13 0

16555855 T1 2 0

16556845 T1 26 0

16556849 T1 29 0

16557780 T1 6 0

16557783 T1 1 0

16557795 T1 2 0

16559016 T2 6 0

16559192 T2 13 0

16559391 T1 34 0

16559897 T1 11 0

16559899 T1 3 0

16559901 T1 2 0

16560324 T1 4 0

16560327 T1 3 0

16561417 T1 19 0

16561418 T1 1 0

16561428 T1 34 1

16561451 T1 12 0

16562869 T1 5 0

16563116 T2 6 0

16563419 T1 1 0

16563420 T1 4 0

16563422 T1 10 4

16563423 T1 14 0

16563440 T1 5 0

16565090 T2 5 0

16565094 T1 3 1

16565096 T1 4 0

16566287 T3 2 0

16567995 T1 3 0

16568074 T1 10 0

16568101 T1 13 0

16568135 T1 9 0

16569186 T1 3 0

16569233 T1 3 0

16569387 T1 22 0

16569417 T2 19 1

16569456 T2 18 0

16570325 T1 5 0

16570327 T1 9 0

16570348 T1 1 0

16571923 T1 3 0

16573125 T1 8 0

16573896 T2 1 0

16573912 T1 17 0

16574195 T1 11 0

16574206 T1 5 0

16574209 T3 17 0

16574628 T1 26 0

16574632 T1 33 0

16574867 T1 6 0

16575383 T1 14 0

16575389 T1 15 0

16575418 T2 5 0

16575762 T2 15 0

16575788 T1 7 0

16576197 T2 5 0

17500064 T1 17 0

17500067 T1 7 0

17500402 T1 6 0

17500407 T1 15 0

17500408 T1 18 0

17500436 T1 28 0

17501789 T3 17 0

17501923 T1 5 0

17501927 T1 2 0

17501933 T1 2 0

17502164 T3 2 0

17503370 T1 30 0

17503374 T1 3 0

17503384 T1 13 0

17503606 T1 5 0

17504772 T1 13 0

17504797 T1 7 0

17504804 T1 19 0

17504820 T1 7 0

17505677 T1 15 0

17505690 T1 21 0

17506002 T1 7 0

17507515 T1 18 0

17507996 T2 6 0

17508419 T1 6 0

17508504 T1 4 1

17508770 T2 19 0

17508791 T2 1 0

17508795 T2 21 0

17509391 T1 13 0

17509950 T1 28 0

17511195 T3 9 0

17511649 T2 6 0

17511670 T1 3 0

17512080 T1 2 0

17512110 T1 25 0

17512839 T1 26 0

17512860 T1 31 0

17512862 T2 2 0

17513260 T1 3 0

17513273 T1 19 0

17513915 T1 12 0

17513942 T1 16 0

17514578 T1 22 3

17515868 T2 6 0

17516662 T3 1 0

17517733 T1 10 0

17517819 T1 3 0

17518038 T1 1 0

17518997 T1 5 0

17519330 T2 9 0

17520320 T1 4 0

17520355 T1 12 0

17520357 T1 9 0

17520408 T2 8 0

17520416 T1 3 0

17520721 T1 28 0

17520724 T2 10 0

17522072 T1 3 0

17522074 T1 5 0

17522078 T1 1 0

17522540 T1 13 0

17522543 T1 3 0

17523253 T1 12 0

17523411 T1 9 0

17523756 T1 9 0

17523760 T1 2 0

17523764 T1 15 0

17523768 T1 15 0

17523770 T1 7 0

17523817 T2 9 0

17524569 T1 4 0

17524583 T1 7 0

17526622 T2 6 0

17526663 T1 2 0

17526708 T2 4 0

17528015 T1 7 0

17528673 T1 5 0

17529032 T1 13 0

17530041 T1 28 0

17530110 T1 27 0

17530166 T1 5 2

17530205 T1 14 0

17530211 T1 13 0

17530541 T2 15 0

17530709 T1 2 0

17531335 T1 2 0

17531960 T1 16 0

17532070 T3 4 0

17532306 T1 1 0

17532631 T1 11 0

17532960 T2 2 0

17532969 T1 4 0

17533431 T1 11 0

17533550 T1 18 0

17533971 T3 4 0

17534698 T1 8 0

17536395 T1 2 0

17536450 T1 6 0

17536504 T1 9 0

17536817 T1 13 0

17536969 T1 1 0

17537213 T1 13 0

17537541 T1 2 0

17537587 T1 8 0

17537838 T1 25 0

17538250 T1 11 0

17538399 T3 16 0

17539416 T2 16 0

17539782 T1 2 0

17540340 T3 8 0

17540462 T2 4 0

17540980 T1 9 0

17541123 T1 6 0

17541292 T3 12 0

17541313 T1 11 0

17541553 T1 7 0

17541667 T1 2 0

17542177 T1 1 0

17542222 T2 6 2

17542773 T1 12 0

17544517 T1 14 1

17544882 T4 2 0

17545567 T1 12 0

17545941 T2 14 0

17546157 T1 13 6

17546166 T1 12 0

17546601 T1 15 0

17546725 T1 5 0

17546797 T1 11 0

17546902 T1 13 0

17546979 T1 18 0

17547428 T1 7 0

17547511 T1 7 0

17548562 T1 24 0

17548584 T1 17 0

17548632 T1 3 0

17548730 T1 15 0

17549132 T1 18 0

17549221 T2 7 0

17549320 T1 31 0

17550661 T1 8 0

17550883 T1 6 0

17550959 T2 8 0

17551018 T1 5 0

17551305 T1 12 0

17551347 T1 4 0

17551363 T1 6 0

17551507 T1 2 0

17551512 T1 19 0

17551742 T1 19 0

17551797 T1 1 0

17551853 T1 13 0

17551934 T1 6 0

17552129 T1 9 0

17552175 T1 1 0

17553140 T1 5 0

17553227 T3 1 1

17554235 T1 5 0

17554277 T1 12 0

17554491 T1 20 0

17555029 T1 5 0

17555084 T1 3 0

17555097 T1 2 0

17555710 T1 9 0

17556075 T2 12 0

17556426 T1 6 0

17557217 T1 7 0

17557879 T1 7 0

17558100 T1 16 0

17558319 T1 15 0

17559048 T1 3 0

17559285 T2 14 0

17559338 T1 14 0

17559724 T1 27 0

17560247 T1 6 0

17560622 T1 18 0

17560767 T1 5 0

17561173 T1 5 0

17561566 T1 10 0

17561895 T1 18 0

17563081 T1 19 0

17563197 T1 9 0

17563944 T2 23 0

17564701 T1 8 0

17564852 T1 2 0

17565547 T1 23 0

17565594 T1 7 0

17565674 T1 13 0

17565789 T1 10 0

17565852 T1 9 0

17565872 T3 31 0

17566180 T2 22 0

17566460 T3 5 0

17566555 T1 21 0

17566595 T1 32 0

17566736 T1 3 0

17566994 T1 2 0

17567065 T2 32 0

17567254 T3 36 1

17567678 T1 3 0

17567809 T1 19 0

17567833 T1 5 0

17567890 T1 4 0

17568152 T1 3 0

17568210 T2 7 0

17568638 T1 10 0

17569294 T1 10 0

17569647 T1 12 0

17570126 T1 9 0

17570266 T3 11 0

17570532 T1 8 0

17570557 T1 17 0

17571259 T1 2 0

17571684 T1 7 0

17571756 T1 12 0

17572269 T2 7 0

17572292 T1 24 2

17573077 T3 18 0

17573079 T1 16 0

17573089 T1 4 0

17573817 T2 7 0

17574211 T1 3 0

17574212 T1 16 0

17574213 T1 8 0

17574302 T1 9 0

17575055 T3 7 1

17575529 T1 14 0

17575967 T2 17 0

17575972 T2 1 0

17575973 T3 23 1

17576605 T2 4 0

17577053 T1 1 0

17577068 T1 3 0

17577073 T1 5 0

17577077 T1 7 0

17578543 T3 12 0

17578552 T1 5 0

17578553 T1 1 0

17578583 T1 5 0

17578591 T1 20 0

17578595 T1 5 0

17580162 T2 20 0

17580188 T1 9 0

17580927 T1 7 0

17581211 T1 12 0

17581242 T1 20 0

17582337 T2 31 0

17582339 T1 31 0

17582342 T1 8 2

17582775 T1 11 0

17582776 T1 5 0

17583843 T1 11 0

17583856 T1 45 0

17585073 T1 1 0

17585080 T1 4 0

17585091 T1 1 0

17585580 T1 6 0

17585581 T1 18 0

17585604 T1 18 0

17585723 T1 9 0

17585793 T1 9 0

17585808 T2 18 0

17586210 T1 5 0

17586420 T1 13 0

17586510 T1 18 0

17586785 T3 5 0

17586949 T1 3 0

17587761 T1 10 0

17587773 T1 1 0

17588505 T2 9 0

17589737 T1 25 0

17589746 T1 2 0

17590673 T1 22 0

17591193 T1 6 0

17591228 T1 7 0

17593123 T1 17 0

17593124 T1 19 0

17593128 T2 17 0

17593133 T1 5 0

17593137 T1 13 0

17593138 T1 3 0

17593143 T2 6 0

17593148 T2 1 0

17593150 T1 16 0

17593152 T2 32 0

17593158 T1 4 0

17593167 T2 9 0

17593170 T1 21 0

17594249 T1 14 0

17594256 T1 8 0

17594264 T1 2 0

17595549 T1 4 0

17595552 T1 4 0

17595554 T2 15 2

17596629 T1 13 0

17597333 T1 3 0

17597335 T2 20 0

17597337 T1 7 0

17597338 T2 1 0

17597339 T1 7 0

17597342 T1 11 0

17598191 T1 3 0

17598457 T1 10 0

17599967 T1 15 0

17601294 T1 34 0

17601655 T2 21 0

17601896 T2 8 0

17602968 T1 19 0

17602972 T1 18 0

17602979 T1 1 0

17602984 T1 14 0

17603425 T1 21 0

17604858 T1 2 0

17605482 T1 7 0

17605788 T2 1 0

17605789 T1 12 0

17605797 T1 12 0

17605805 T1 7 0

17605813 T1 14 0

17605816 T1 13 0

17605818 T1 2 0

17606950 T1 17 0

17607735 T1 14 0

17607976 T1 20 0

17607981 T1 1 0

17607982 T1 1 0

17607998 T1 15 0

17608008 T1 6 0

17608014 T1 21 0

17609224 T1 5 0

17610625 T1 25 0

17610632 T1 20 0

17610643 T1 10 0

17610645 T1 6 0

17610646 T4 15 0

17610651 T1 1 0

17611345 T2 5 0

17611745 T1 6 0

17611754 T1 12 0

17611759 T1 6 0

17611762 T3 18 0

17611834 T1 13 0

17613044 T1 27 0

17613432 T1 28 0

17613437 T1 14 0

17613448 T1 4 0

17613450 T1 3 0

17613453 T1 27 0

17613454 T1 5 0

17613461 T2 19 3

17613467 T3 21 0

17613470 T1 31 0

17614251 T1 32 0

17614254 T1 8 0

17614796 T1 12 0

17614798 T1 23 0

17614799 T2 1 0

17614817 T1 18 0

17614819 T1 14 0

17614822 T1 9 0

17614827 T1 2 0

17617447 T1 3 0

17617449 T2 14 0

17617462 T1 5 0

17617463 T1 5 0

17617474 T1 2 0

17617487 T1 9 0

17617499 T3 1 0

17618717 T1 16 0

17618720 T1 23 0

17618732 T1 7 0

17618737 T1 2 0

17618743 T1 14 0

17618745 T1 24 0

17618750 T2 7 0

17618753 T1 2 0

17618757 T1 1 0

17618758 T2 13 0

17620180 T1 9 0

17620287 T1 30 1

17620664 T1 1 0

17620875 T1 8 0

17621317 T1 39 0

17621320 T1 11 0

17621932 T1 19 0

17622238 T1 4 0

17622240 T1 2 0

17622250 T1 11 0

17622253 T1 14 1

17622262 T2 8 0

17623679 T1 4 0

17625275 T1 33 0

17625279 T1 2 0

17625282 T1 12 0

17625286 T2 1 0

17625310 T1 7 0

17625312 T1 21 4

17625314 T1 4 0

17625316 T1 9 0

17625318 T1 8 0

17625320 T2 39 0

17625328 T1 1 0

17626353 T1 26 0

17626359 T1 2 0

17627149 T1 10 0

17627576 T1 22 0

17627581 T1 3 0

17627587 T2 6 0

17628551 T1 15 0

17628553 T1 23 0

17628556 T1 5 0

17628560 T1 6 0

17628563 T1 15 0

17629204 T1 2 0

17629432 T1 19 0

17629721 T2 1 0

17629827 T1 4 0

17630575 T1 4 0

17631680 T2 32 0

17632413 T1 22 3

17632434 T2 6 0

17632475 T1 14 0

17632517 T2 19 0

17632527 T1 8 0

17632878 T1 7 0

17632888 T1 1 1

17632898 T1 11 0

17632899 T1 27 0

17632900 T1 12 0

17632906 T1 9 0

17632918 T1 20 0

17633678 T1 12 0

17633843 T1 23 0

17634478 T3 16 0

17635379 T1 12 0

17635391 T1 4 0

17635400 T2 10 0

17635406 T1 15 0

17636420 T1 4 0

17636929 T1 10 0

17637984 T1 4 0

17637985 T1 6 0

17637986 T2 2 0

17637987 T1 19 0

17637993 T1 2 0

17637996 T1 3 0

17637999 T2 4 0

17638000 T1 13 0

17639032 T1 7 0

17639667 T1 15 0

17640473 T1 6 0

17640827 T1 2 0

17640833 T2 8 0

17640839 T1 2 0

17642133 T1 3 0

17642144 T1 6 0

17642514 T1 10 0

17642706 T1 17 0

17642887 T1 20 0

17643189 T1 17 0

17643197 T1 3 0

17643199 T1 10 0

17643201 T1 15 0

17643204 T1 15 0

17643211 T3 19 0

17643212 T1 26 0

17643231 T2 3 0

17643612 T3 9 0

17643618 T1 10 0

17643619 T1 3 0

17644806 T2 25 3

17645549 T2 2 0

17645556 T1 3 0

17645566 T1 32 0

17645569 T1 2 0

17646270 T1 7 0

17646274 T1 3 0

17646275 T1 21 0

17647293 T1 15 0

17647296 T1 4 0

17647315 T1 17 0

17647326 T1 4 0

17648641 T1 22 1

17648644 T1 6 0

17648649 T1 9 0

17648652 T1 9 0

17648698 T1 21 0

17649546 T1 27 0

17649550 T1 6 0

17650543 T1 9 0

17650548 T1 27 0

17650553 T2 9 0

17650571 T1 10 0

17651278 T1 24 0

17651857 T1 12 0

17652711 T3 5 0

17652715 T1 4 0

17652716 T2 5 0

17652730 T1 22 0

17653766 T3 11 0

17653774 T1 9 0

17653779 T1 10 0

17653786 T1 16 0

17653797 T1 19 1

17653801 T1 1 0

17654979 T2 17 0

17654982 T1 10 0

17655610 T1 14 0

17655624 T1 18 0

17655625 T1 10 0

17655630 T2 11 1

17655632 T1 1 0

17655639 T1 8 0

17655647 T1 18 0

17656289 T1 23 0

17656320 T1 15 0

17656386 T1 14 0

17656415 T1 7 0

17657414 T1 18 0

17657417 T1 1 0

17657426 T1 6 0

17657429 T2 3 0

17657432 T3 15 0

17657434 T1 16 0

17657449 T2 15 0

17658209 T1 9 0

17658214 T1 7 0

17658218 T1 6 0

17658230 T1 6 0

17658238 T2 4 0

17659661 T1 40 1

17659825 T1 11 0

17659829 T1 4 0

17659838 T2 11 3

17659845 T2 33 0

17659849 T1 18 0

17659868 T1 5 0

17660526 T1 10 0

17660532 T1 11 0

17660912 T1 20 0

17661245 T1 7 0

17661259 T1 4 0

17661263 T1 4 0

17661266 T1 20 0

17661274 T1 14 0

17661277 T3 13 0

17662355 T1 19 0

17663181 T2 12 0

17663185 T1 13 0

17663186 T3 12 0

17664993 T2 8 0

17665727 T1 4 0

17666852 T1 18 0

17666855 T1 2 0

17666858 T2 14 1

17666873 T1 9 0

17666875 T1 3 0

17667741 T1 2 0

17667795 T1 9 0

17668118 T2 21 1

17668588 T1 24 0

17668806 T1 12 0

17668810 T3 9 0

17668854 T2 8 0

17668857 T1 2 1

17668866 T1 7 0

17670215 T2 30 0

17671349 T1 3 0

17671353 T1 4 0

17671358 T1 26 0

17671362 T1 6 0

17671365 T1 8 0

17671366 T1 2 0

17671369 T1 3 0

17671375 T1 9 0

17671391 T2 13 0

17671393 T1 18 0

17672168 T1 10 0

17672272 T2 12 0

17672282 T1 24 0

17672343 T1 18 0

17672345 T1 18 0

17672354 T1 15 0

17673246 T1 24 0

17674278 T1 12 0

17675378 T2 1 0

17675380 T1 8 0

17675381 T1 5 0

17675388 T1 1 0

17676506 T1 6 0

17676511 T1 11 0

17679463 T1 19 0

17680611 T3 6 6

17681341 T1 10 0

17681344 T1 8 0

17681354 T3 11 0

17681355 T2 16 0

17681356 T1 15 0

17682002 T1 4 0

17682003 T1 12 0

17682006 T1 10 0

17682007 T3 23 0

17682231 T1 12 0

17682567 T2 5 0

17683127 T1 2 0

17683131 T3 8 0

17683141 T2 8 0

17683146 T1 17 0

17683150 T2 6 0

17683154 T2 7 0

17683156 T3 39 0

17683161 T1 11 2

17684585 T2 10 0

17684591 T1 3 0

17684596 T1 12 0

17684598 T1 12 0

17684608 T1 20 0

17685157 T4 20 0

17685504 T2 4 0

17685835 T2 22 0

17685838 T1 8 0

17687099 T3 8 5

17687102 T2 30 0

17687114 T1 7 0

17687130 T1 14 7

17687133 T2 22 0

17687134 T1 4 0

17687144 T2 10 0

17687145 T1 3 0

17687154 T2 9 0

17687156 T1 16 0

17687158 T1 24 0

17687589 T1 10 0

17687591 T1 28 0

17688191 T2 17 0

17688192 T2 15 0

17689123 T2 4 0

17689459 T1 46 0

17689467 T1 6 0

17689470 T2 17 0

17689481 T1 11 0

17690494 T2 17 0

17690510 T1 6 0

17690513 T1 6 0

17690514 T1 9 0

17690518 T1 5 0

17690519 T1 9 0

17690521 T2 2 0

17690535 T1 23 0

17690541 T2 14 1

17691487 T1 30 0

17691500 T1 11 0

17692007 T3 5 0

17692024 T1 18 0

17692025 T1 9 1

17693002 T1 2 0

17693005 T1 9 0

17693292 T1 13 0

17693301 T1 9 0

17693304 T2 21 0

17693322 T1 9 0

17693324 T1 8 0

17693326 T1 7 0

17693332 T3 13 0

17693341 T1 18 0

17694192 T2 7 0

17694705 T1 4 0

17694790 T2 13 0

17694793 T1 1 0

17694903 T1 13 0

17694939 T3 42 3

17694976 T1 14 0

17695094 T1 42 0

17695408 T1 7 0

17695410 T2 9 1

17695422 T2 20 0

17695426 T1 14 0

17695429 T1 22 0

17695433 T1 11 0

17695436 T1 16 0

17695437 T1 9 0

17695440 T2 12 1

17695450 T2 30 0

17696126 T1 8 0

17696201 T1 11 0

17696235 T1 12 0

17696646 T1 12 0

17696648 T1 4 0

17696651 T1 4 0

17696652 T1 12 0

17696663 T1 15 0

17697771 T1 33 0

17697777 T3 2 0

17697781 T3 29 0

17698265 T1 2 0

17698689 T1 25 0

17698691 T1 11 0

17698694 T3 6 2

17698697 T2 3 0

17698700 T1 5 0

17698704 T1 1 0

17698711 T1 5 0

17698725 T1 17 0

17698727 T1 19 0

17699503 T1 38 0

17699509 T2 15 0

17699762 T1 5 0

17699770 T1 7 0

17699780 T1 2 0

17699783 T1 9 0

17699785 T1 16 0

17699798 T3 17 0

17699802 T1 14 0

17699807 T1 22 0

17700584 T1 4 0

17700886 T1 2 0

17700917 T1 24 0

17700920 T1 19 0

17702035 T1 6 0

17702040 T1 2 0

17702472 T1 22 0

17702473 T1 4 0

17703297 T1 5 1

17703299 T1 12 0

17704397 T1 1 0

17704409 T2 11 0

17704420 T1 7 0

17704448 T1 33 0

17705484 T1 26 0

17705821 T1 9 0

17705947 T1 5 0

17706496 T1 11 0

17706564 T1 6 0

17707062 T1 5 0

17708393 T1 5 0

17708571 T2 16 0

17708616 T3 11 0

17708722 T1 10 0

17708941 T1 9 0

17709394 T1 2 0

17709638 T1 3 0

17709850 T2 4 0

17709960 T1 4 0

17710612 T3 15 0

17710613 T1 10 0

17710619 T3 24 0

17710620 T1 20 0

17710622 T1 2 0

17710637 T1 19 0

17710641 T1 5 0

17710645 T1 14 0

17710646 T1 9 0

17711498 T1 1 0

17712239 T1 6 0

17712241 T2 25 0

17712259 T1 4 0

17712270 T3 8 0

17713519 T2 20 0

17713522 T1 14 0

17713829 T1 24 0

17713969 T1 7 0

17714533 T2 15 0

17714716 T1 7 0

17714725 T1 26 3

17715459 T1 27 0

17715810 T1 3 0

17716169 T1 7 0

17716434 T1 12 0

17716443 T4 1 0

17716448 T1 10 0

17716450 T2 12 0

17716454 T1 6 0

17717166 T1 11 0

17717167 T1 14 0

17718433 T1 10 0

17718437 T3 6 3

17718440 T1 4 0

17718447 T1 5 0

17718449 T2 9 0

17718456 T1 7 0

17718459 T1 7 0

17718460 T2 4 0

17718468 T1 13 0

17718471 T1 18 0

17719970 T1 8 1

17719972 T2 12 0

17719974 T3 22 0

17719976 T1 7 0

17719978 T1 11 0

17719985 T1 25 0

17719999 T1 1 0

17720004 T1 12 0

17722072 T1 12 0

17722076 T1 4 0

17723038 T1 19 0

17723613 T1 22 0

17723618 T3 25 0

17724297 T1 1 0

17724750 T3 10 0

17725125 T1 14 0

17725504 T1 15 0

17726429 T2 25 0

17726432 T1 6 0

17726434 T1 2 0

17726435 T1 10 0

17726792 T2 5 0

17727632 T1 10 0

17728624 T1 5 0

17728625 T1 9 0

17728630 T1 2 0

17728636 T1 8 0

17729511 T1 9 0

17730231 T1 3 0

17730504 T1 2 0

17730639 T2 18 0

17730720 T3 17 0

17731412 T1 8 0

17731414 T1 6 0

17731417 T3 12 0

17731423 T1 4 0

17731432 T1 9 0

17731435 T1 6 0

17731438 T1 14 0

17733022 T1 8 0

17733026 T1 4 0

17733027 T1 7 0

17734154 T2 4 1

17734848 T1 11 0

17734856 T1 6 0

17734859 T1 34 0

17734861 T1 15 0

17735164 T2 15 0

17735553 T2 3 0

17735628 T2 10 0

17735942 T1 5 0

17735945 T3 1 1

17736316 T1 29 0

17736319 T2 24 0

17736325 T1 17 0

17736328 T1 7 0

17736646 T1 3 0

17736884 T1 6 0

17737351 T1 2 0

17737355 T1 6 0

17738024 T1 11 0

17738027 T3 3 0

17738315 T1 3 0

17738318 T1 24 0

17738768 T1 25 0

17738839 T1 16 0

17739243 T1 11 0

17739246 T1 8 0

17739605 T1 4 0

17740214 T1 31 0

17740220 T1 2 0

17740424 T1 4 0

17740430 T1 21 0

17740970 T1 4 0

17740982 T3 9 3

17741378 T1 27 0

17741381 T1 12 0

17741696 T1 11 0

17741913 T2 3 0

17741915 T1 16 0

17741916 T2 14 0

17741921 T1 7 0

17742305 T1 11 0

17742308 T2 15 0

17742728 T1 5 2

17743039 T1 15 0

17743080 T1 11 0

17743444 T1 7 0

17743596 T1 8 0

17743602 T1 13 0

17744130 T1 20 0

17744142 T1 3 0

17744154 T2 16 0

18666894 T1 12 0

18666898 T1 9 0

18667631 T1 9 3

18667632 T1 9 0

18667927 T1 15 0

18668328 T3 6 0

18668688 T1 17 0

18669805 T2 6 0

18671206 T1 26 0

18671208 T2 5 0

18671866 T1 14 0

18671873 T1 2 0

18671877 T1 26 0

18672279 T1 9 0

18672282 T1 3 0

18672285 T1 4 0

18672514 T1 24 0

18672787 T2 11 0

18672820 T1 42 0

18672821 T1 13 0

18672870 T1 6 0

18674195 T2 14 0

18674220 T1 14 0

18674289 T1 6 0

18674294 T2 21 0

18674308 T1 4 0

18674374 T1 10 3

18674413 T1 4 0

18674437 T1 15 0

18674438 T1 3 0

18675496 T1 15 0

18675502 T1 6 0

18675507 T1 27 0

18675510 T1 10 0

18676477 T1 13 0

18676486 T1 2 0

18677138 T1 5 0

18677139 T3 22 2

18677140 T1 25 0

18677143 T1 12 0

18678301 T3 14 2

18678311 T1 6 0

18678630 T1 3 0

18678863 T1 18 0

18678865 T1 20 0

18679119 T1 17 0

18679143 T1 19 1

18679277 T2 14 0

18679437 T2 3 0

18679438 T1 4 0

18679443 T2 4 0

18679851 T2 15 0

18680262 T1 9 0

18681363 T2 11 0

18681594 T1 2 0

18681606 T1 13 0

18681609 T1 10 0

18681617 T1 3 0

18681623 T1 31 0

18682367 T2 18 0

18682466 T1 13 5

18682524 T3 5 0

18682921 T1 4 0

18683095 T1 10 0

18683295 T3 8 0

18684183 T1 4 0

18684497 T1 4 0

18684700 T1 8 0

18684704 T1 10 0

18684707 T1 5 0

18684711 T1 17 0

18684715 T2 11 0

18684718 T1 10 0

18684719 T1 20 0

18684954 T1 7 0

18684955 T1 16 0

18685186 T1 16 1

18685487 T1 21 0

18685490 T1 10 0

18685692 T1 1 0

18686146 T3 24 4

18686153 T2 7 3

18686275 T1 10 0

18686611 T1 17 1

18686625 T1 3 0

18686629 T2 35 1

18687391 T1 17 0

18687393 T1 14 0

18687493 T2 19 1

18687904 T1 26 0

18687908 T1 5 0

18687909 T1 4 0

18687910 T3 17 4

18687913 T1 19 0

18688288 T2 8 0

18688291 T2 16 2

18688292 T1 9 1

18688295 T2 8 0

18688873 T2 18 0

18689210 T1 5 0

18689395 T1 10 0

18689398 T1 10 0

18689403 T1 17 0

18689405 T1 2 0

18689919 T1 9 0

18689921 T2 3 0

18689927 T1 16 0

18689930 T1 4 0

18690314 T1 9 0

18690664 T2 7 0

18690850 T1 8 0

18691309 T2 5 0

18691633 T2 4 0

18692071 T1 5 0

18692594 T1 32 0

18692598 T1 17 0

18693188 T1 10 0

18693501 T3 17 0

18693503 T2 5 0

18693981 T2 12 0

18693982 T1 17 0

18694728 T3 17 0

18694736 T1 24 0

18695279 T1 10 0

18695453 T1 5 0

18695461 T3 15 0

18695487 T1 19 0

18695496 T1 19 0

18695529 T2 7 0

18695541 T1 16 0

18695545 T2 14 0

18696519 T1 20 0

18697718 T2 15 0

18697721 T1 19 0

18697722 T2 18 0

18697725 T1 15 0

18697728 T1 10 0

18697729 T3 10 0

18697733 T1 10 0

18697736 T1 10 0

18698318 T4 3 0

18698446 T1 13 0

18698596 T1 17 0

18699230 T1 11 0

18699251 T2 7 0

18699337 T3 25 0

18699347 T3 25 0

18700071 T1 2 0

18700073 T2 16 1

18700290 T2 42 0

18700295 T1 13 0

18700300 T1 2 0

18700302 T1 28 0

18700891 T3 27 5

18700896 T3 12 0

18701029 T2 17 0

18701261 T3 12 0

18701797 T2 14 0

18702870 T1 31 0

18703371 T1 16 0

18703374 T1 9 0

18703378 T1 7 0

18703381 T1 9 0

18703585 T1 21 0

18704199 T1 22 0

18705522 T3 22 0

18705525 T2 22 0

18705534 T1 14 0

18705542 T2 5 0

18705549 T1 13 0

18705554 T1 17 0

18705558 T1 23 0

18706453 T1 20 0

18706701 T1 20 0

18706703 T1 6 0

18706970 T1 17 0

18707198 T2 10 0

18707201 T1 17 0

18707710 T1 25 0

18707711 T1 20 0

18708656 T2 13 0

18708657 T1 17 0

18708664 T2 14 0

18708665 T1 3 0

18708839 T1 22 0

18710339 T1 17 0

18710532 T1 7 0

18711711 T1 10 0

18711720 T1 2 0

18711723 T3 23 0

18712180 T2 4 0

18712327 T1 16 0

18712953 T1 36 0

18712954 T2 33 0

18712956 T3 1 0

18712958 T1 25 0

18713314 T1 7 0

18713315 T2 15 0

18713672 T1 39 0

18713674 T1 11 0

18713741 T1 3 0

18713742 T2 4 0

18714027 T3 9 0

18714031 T1 2 0

18714429 T1 8 0

18714435 T1 21 0

18714817 T1 14 0

18714824 T1 15 0

18714826 T1 19 0

18715095 T1 12 0

18715099 T1 7 0

18715356 T1 10 0

18715358 T2 1 1

18715360 T1 17 0

18715891 T1 12 0

18715894 T3 24 0

18716144 T1 17 0

18716720 T1 8 0

18716722 T1 13 0

18716727 T2 30 0

18717390 T1 23 0

18717732 T1 27 0

18718481 T1 18 0

18718541 T3 1 1

18719067 T2 17 0

18719406 T2 18 0

18719749 T1 12 0

18719827 T1 16 0

18719829 T1 23 0

18719831 T4 13 0

18719832 T1 9 0

18719835 T2 20 0

18720612 T1 30 0

18721185 T2 11 0

18721360 T1 8 0

18721539 T1 25 1

18721541 T2 11 0

18721807 T1 39 1

18721858 T2 8 0

18722153 T1 29 0

18722156 T1 24 0

18722371 T1 12 2

18722504 T3 14 0

18722507 T1 7 0

18722511 T1 28 0

18723213 T3 4 0

18723215 T1 11 0

18723218 T1 9 0

18723242 T1 8 0

18723376 T1 7 0

18723382 T1 2 0

18723384 T1 4 0

18723386 T1 11 0

18723390 T1 6 0

18723393 T2 12 0

18723397 T1 3 0

18723407 T1 8 0

18723409 T1 12 0

18724482 T1 18 0

18724484 T3 23 0

18725083 T1 24 0

18725087 T1 6 0

18725089 T2 23 0

18725369 T1 17 0

18725370 T4 9 4

18725371 T1 10 0

18725374 T1 5 0

18725375 T1 10 0

18725753 T1 11 0

18725875 T1 7 0

18726103 T1 4 0

18726106 T1 2 0

18726109 T1 5 0

18726111 T1 10 1

18727042 T3 19 0

18727046 T3 15 4

18727049 T1 11 0

18727052 T3 10 1

18727871 T1 4 0

18727872 T1 2 0

18727875 T1 20 0

18727881 T1 19 0

18727891 T1 29 0

18727894 T2 33 0

18727896 T1 1 0

18728491 T1 16 0

18728496 T1 2 0

18728762 T2 16 0

18728965 T1 21 0

18728968 T1 9 0

18728973 T1 4 0

18729789 T1 30 0

18729992 T1 24 0

18729996 T2 7 0

18729998 T1 7 0

18730066 T1 13 0

18730078 T1 24 0

18730332 T1 27 0

18730343 T1 1 0

18730676 T2 19 0

18730679 T1 28 0

18730770 T2 27 0

18730896 T1 10 0

18730952 T1 2 0

18731014 T1 21 0

18732222 T3 5 1

18732608 T1 3 0

18732610 T3 64 0

18733200 T2 15 0

18733275 T2 5 0

18733285 T1 3 0

18733288 T1 14 0

18734195 T1 3 0

18734203 T1 22 0

18734206 T1 3 0

18734209 T1 10 0

18734212 T3 11 4

18734318 T2 13 0

18734437 T2 14 1

18735096 T2 42 0

18735176 T1 2 0

18735609 T1 8 0

18735612 T3 17 0

18736068 T1 4 0

18736254 T1 3 0

18736257 T1 18 0

18736953 T1 8 0

18736955 T2 35 0

18737462 T1 9 0

18737856 T1 11 1

18738094 T1 10 0

18738097 T1 12 0

18738588 T2 10 0

18738958 T1 21 0

18739252 T1 22 0

18739255 T1 10 0

18739750 T2 5 0

18741359 T1 24 0

18741360 T1 7 0

18741363 T1 35 0

18741366 T1 11 0

18742392 T2 16 0

18742819 T1 7 0

18743147 T2 34 1

18743396 T1 14 0

18743398 T3 19 1

18744205 T1 1 0

18744276 T1 8 0

18744896 T2 39 0

18745001 T1 31 0

18745230 T1 19 0

18745238 T1 17 0

18745510 T1 20 0

18745907 T1 13 0

18745967 T1 9 0

18746397 T1 4 0

18746685 T3 2 0

18747017 T2 1 0

18747142 T2 18 0

18747323 T1 21 0

18747418 T1 32 0

18749331 T3 3 1

18749371 T1 20 0

18749395 T1 15 0

18749400 T1 4 0

18750433 T2 11 0

18750435 T2 28 0

18751218 T1 14 0

18751760 T1 12 0

18751771 T1 8 0

18751774 T1 7 0

18751775 T1 9 0

18751778 T1 5 0

18751780 T1 13 0

18751782 T1 5 0

18755821 T1 3 0

18755832 T1 10 0

18757380 T1 7 0

18758218 T2 12 0

18760512 T1 5 0

18760514 T1 8 0

18760994 T2 18 0

18761865 T1 5 0

18764733 T1 46 0

18766327 T1 6 0

18766330 T1 10 0

18766954 T2 9 0

18766963 T1 14 0

18767651 T1 11 0

18848890 T1 13 0

19854640 T1 27 0

19902047 T1 15 0

19902048 T1 13 0

21081489 T1 6 0

21081661 T2 35 0

21082022 T1 8 0

21082954 T2 1 0

21084852 T1 3 0

21085407 T2 48 0

21085941 T1 16 0

21086640 T1 9 0

21087072 T1 30 0

21087696 T1 5 0

21088275 T1 6 0

21088785 T3 2 1

21088847 T1 16 0

21089423 T1 28 0

21089535 T1 10 0

21090090 T1 11 0

21090225 T1 15 0

21090241 T1 3 0

21092407 T1 2 0

21092445 T1 5 0

21093701 T1 12 0

21093762 T1 2 0

21094583 T1 10 0

21094803 T1 2 0

21095559 T1 9 0

21095699 T1 13 0

21096847 T1 21 0

21097585 T1 8 0

21098154 T1 9 0

21098953 T3 15 0

21099540 T3 4 0

21099585 T1 24 0

21099657 T1 17 0

21100439 T1 7 0

21101102 T1 11 0

21101306 T1 8 0

21101448 T1 7 0

21101702 T1 3 0

21101942 T1 5 0

21102201 T1 2 0

21102241 T1 34 0

21102264 T1 9 0

21103746 T1 27 22

21104257 T1 5 1

21104316 T1 3 0

21105093 T1 14 0

21106009 T1 2 0

21106064 T1 7 0

21106651 T1 10 0

21107018 T1 5 0

21107246 T1 4 0

21107869 T1 18 0

21108564 T1 15 0

21109314 T1 9 0

21109657 T1 13 0

21109889 T1 12 0

21110006 T1 3 0

21110034 T1 20 0

21110186 T1 18 0

21111411 T1 11 0

21112753 T1 11 0

21112794 T1 4 0

21112798 T1 4 0

21112861 T1 12 0

21112888 T1 5 0

21113588 T1 8 0

21113871 T2 10 0

21113905 T1 5 0

21114943 T1 4 0

21115698 T1 6 0

21115738 T1 6 0

21116174 T1 12 0

21116411 T1 12 0

21116496 T1 18 0

21116547 T1 4 0

21116683 T1 8 0

21116751 T1 5 0

21117635 T2 18 0

21117698 T1 10 0

21117738 T1 20 0

21118549 T1 6 0

21118762 T1 25 0

21118845 T1 3 0

21118862 T1 1 0

21119060 T1 25 0

21119562 T1 22 0

21120241 T1 25 0

21120646 T2 3 0

21120782 T1 7 0

21120833 T1 13 0

21120881 T2 19 0

21121330 T2 31 0

21122371 T1 8 0

21122834 T1 5 0

21123315 T1 2 0

21123606 T1 6 0

21123742 T1 2 0

21123768 T1 4 0

21123784 T1 8 0

21123867 T1 5 0

21124326 T1 14 0

21124349 T1 7 0

21124466 T1 4 0

21124555 T1 1 0

21124756 T1 4 0

21124865 T1 16 0

21125481 T1 13 0

21125907 T1 7 0

21126344 T1 6 0

21126346 T1 17 0

21126640 T1 7 0

21127087 T1 4 0

21127540 T1 27 0

21130146 T1 1 0

21130812 T1 7 0

21130953 T1 14 0

21131860 T1 12 0

21131882 T1 2 0

21132260 T1 15 0

21132735 T1 6 0

21132743 T2 2 0

21132819 T1 6 0

21132910 T1 14 0

21133036 T2 5 0

21133785 T1 3 0

21133980 T1 5 0

21134356 T2 5 0

21134510 T2 10 0

21134563 T1 22 0

21134891 T4 5 0

21134910 T1 10 0

21135960 T1 6 0

21136043 T1 11 0

21136101 T1 6 0

21137511 T1 7 0

21137827 T1 4 0

21137953 T1 7 0

21138365 T1 1 1

21138516 T1 4 0

21139105 T3 3 0

21139407 T1 7 0

21139848 T1 30 0

21140618 T2 11 0

21140921 T1 15 0

21141525 T1 4 0

21141808 T2 6 0

21141885 T1 15 0

21143809 T1 4 0

21144063 T1 6 0

21144135 T1 2 0

21144989 T1 14 0

21145469 T1 5 0

21145612 T1 1 0

21147222 T1 7 0

21149051 T1 8 0

21150288 T1 5 0

21150890 T1 1 0

21151246 T1 4 0

21152361 T3 4 0

21152362 T1 8 0

21152574 T1 14 0

21153626 T1 4 3

21153754 T1 3 0

21154198 T1 3 0

21154419 T1 1 0

21155076 T1 1 0

21155138 T1 3 2

21155773 T1 13 0

21155819 T1 7 0

21156491 T1 5 0

21158360 T1 9 0

21158656 T1 17 0

21160816 T1 2 0

21161072 T3 1 0

21161683 T1 9 0

21162448 T1 10 0

21162876 T1 6 0

21163120 T1 2 0

21163144 T2 2 0

21163466 T1 16 0

21164432 T1 17 0

21164712 T1 4 0

21165546 T3 5 0

21166127 T1 3 0

21166308 T1 5 0

21166700 T1 2 0

21168848 T1 4 0

21168909 T1 3 0

21169290 T3 13 1

21169578 T1 15 0

21169656 T1 2 0

21170122 T1 1 0

21170510 T3 3 0

21170713 T4 1 1

21171282 T1 10 0

21172195 T1 3 0

21172583 T1 5 0

21172681 T1 7 0

21173510 T1 3 0

21173706 T1 2 0

21174097 T1 6 0

21174163 T3 7 1

21174420 T3 3 1

21175255 T2 10 0

21175309 T1 9 0

21175385 T1 16 0

22000166 T2 3 1

22000290 T3 6 0

22000542 T2 8 0

22000637 T1 3 0

22000701 T1 1 0

22002168 T1 18 0

22003464 T1 4 0

22003490 T1 18 0

22004079 T1 2 0

22004159 T1 1 0

22004685 T1 8 0

22004773 T1 5 0

22004886 T1 33 0

22004947 T1 13 0

22005009 T1 9 6

22006522 T1 19 0

22007550 T3 23 0

22007798 T1 1 0

22007977 T1 2 0

22008256 T2 27 0

22008343 T1 12 2

22008498 T1 21 0

22008589 T1 19 0

22008722 T1 3 0

22008727 T1 7 0

22008953 T1 1 0

22009492 T1 4 0

22009933 T3 14 0

22010804 T1 3 0

22010864 T1 2 0

22011143 T2 35 2

22011338 T2 3 0

22011996 T1 3 0

22012088 T1 4 0

22012119 T3 7 0

22012727 T1 3 0

22013163 T1 14 0

22014619 T1 1 0

22014682 T1 1 0

22014763 T1 9 0

22014783 T1 8 0

22014785 T1 6 0

22016137 T1 12 0

22016872 T1 1 1

22017526 T1 1 0

22018122 T1 1 0

22018338 T2 12 0

22018350 T3 6 0

22018565 T1 3 0

22018954 T3 2 0

22019211 T1 13 0

22019481 T1 1 0

22019599 T1 8 0

22020322 T1 1 0

22020399 T1 3 0

22020958 T1 3 0

22022011 T3 3 0

22023325 T1 30 0

22023446 T1 4 0

22023661 T3 21 3

22023747 T2 20 0

22023765 T1 2 0

22023991 T1 2 0

22024052 T3 18 0

22024362 T1 15 0

22024616 T1 13 0

22024729 T1 28 0

22026033 T1 10 0

22027610 T1 1 0

22028463 T1 4 0

22028514 T3 13 0

22028626 T1 20 1

22029024 T1 7 0

22029267 T1 23 0

22030411 T1 2 0

22030442 T1 43 0

22031042 T2 15 0

22031384 T1 1 0

22031507 T1 2 0

22031558 T2 3 0

22031764 T1 10 0

22032642 T1 9 0

22033512 T1 5 0

22033559 T2 21 0

22033913 T1 2 0

22034307 T1 18 0

22034592 T1 9 0

22035415 T1 3 0

22035432 T1 16 0

22035944 T1 4 0

22036066 T2 13 0

22036093 T2 3 1

22036112 T1 5 0

22036182 T1 3 0

22036484 T1 18 0

22036619 T1 2 0

22037170 T1 2 0

22037348 T1 2 0

22037481 T1 25 0

22037523 T1 9 0

22037808 T3 9 1

22038044 T2 4 0

22038125 T1 16 0

22038134 T1 1 0

22038142 T1 11 0

22038231 T1 11 0

22038860 T2 4 2

22039764 T1 9 0

22039802 T1 23 0

22039904 T1 23 0

22040163 T1 10 0

22040489 T1 27 0

22040702 T2 17 0

22040893 T1 12 0

22040961 T1 4 0

22041064 T1 1 0

22041282 T1 7 0

22041287 T1 6 0

22042633 T1 1 0

22043094 T1 22 0

22044142 T1 2 0

22044179 T1 20 0

22044418 T2 8 3

22044710 T1 26 0

22044868 T1 9 0

22044953 T1 43 0

22044999 T1 2 0

22045106 T1 3 0

22045257 T1 16 0

22045613 T1 12 0

22046168 T1 27 0

22046467 T1 11 0

22046493 T2 33 0

22046569 T1 17 2

22046829 T1 3 0

22046916 T1 4 0

22047212 T1 7 0

22047284 T1 28 0

22048102 T1 8 0

22048293 T1 36 0

22048391 T1 16 0

22048524 T3 3 1

22048651 T2 6 3

22048691 T1 9 0

22048883 T1 14 0

22048907 T1 33 0

22048991 T3 6 1

22049160 T3 6 0

22049596 T1 6 0

22049743 T1 2 0

22049949 T1 23 0

22050639 T1 17 0

22052038 T1 7 0

22052370 T1 24 0

22052425 T1 22 0

22052454 T1 18 0

22052455 T1 10 0

22052470 T1 14 0

22052509 T1 1 0

22053019 T1 2 0

22053023 T1 6 0

22053038 T1 1 0

22053216 T1 2 0

23086555 T1 2 0

24314107 T1 5 0

24315773 T1 1 0

24316920 T1 2 0

24323208 T1 1 0

24323327 T1 3 0

24323537 T1 2 0

24324788 T2 4 0

24326789 T1 6 0

24326860 T2 15 0

24326865 T1 3 0

24327896 T1 4 0

24328037 T1 5 0

24328805 T1 4 0

24329342 T1 5 0

24329456 T2 10 0

24330042 T2 5 0

24331694 T1 3 0

24331745 T1 7 0

24332863 T1 2 0

24333323 T1 36 0

24333964 T1 12 0

24335368 T1 6 0

24335612 T1 1 1

24336040 T1 8 0

24338440 T2 1 0

24338771 T1 2 0

24338790 T2 2 0

24338815 T2 23 0

24340612 T1 2 0

24342688 T2 31 0

24343870 T1 2 0

24343877 T1 2 0

24343897 T1 9 0

24343970 T3 29 0

24347172 T1 13 0

24347656 T1 13 0

24347827 T1 1 0

24347976 T1 6 0

24348268 T1 6 0

24348635 T2 1 0

24349245 T1 8 0

24349423 T1 2 0

24350071 T1 5 0

24351078 T1 4 0

24351362 T1 18 0

24351405 T1 5 0

24351747 T1 11 0

24351766 T1 4 0

24352535 T1 4 0

24353004 T1 2 0

24353163 T1 3 0

24353566 T1 12 0

24353990 T1 9 0

24354315 T2 6 0

24354399 T1 1 0

24354408 T1 1 0

24354999 T1 3 0

24356246 T2 8 0

24357322 T1 6 0

24358089 T1 10 0

24358244 T1 7 0

24360035 T1 5 0

24360594 T2 5 0

24360622 T2 4 0

24361162 T1 6 0

24361954 T1 2 0

24361961 T1 1 0

24362057 T3 32 0

24362867 T2 10 0

24363534 T1 3 0

24363544 T1 2 0

24363645 T1 9 0

24364392 T3 18 0

24364466 T1 4 0

24365200 T1 11 0

24365333 T1 5 0

24365751 T1 1 0

24365833 T2 7 0

24366537 T1 4 0

24366968 T1 5 0

24367108 T1 2 0

24367336 T1 13 0

24367947 T2 6 0

24368399 T1 2 0

24369262 T1 2 0

24373606 T1 5 0

24374001 T1 2 0

24374166 T2 2 0

24375020 T1 20 0

24375296 T1 2 0

24375589 T1 5 0

24375694 T1 14 0

24375780 T1 1 0

24375880 T2 8 0

24375905 T1 2 0

24375949 T1 8 0

24375959 T1 9 0

24376332 T1 3 0

24377045 T1 4 0

24377101 T1 6 0

24378035 T3 4 0

24378351 T3 1 0

24379485 T2 8 0

24379590 T3 1 0

24379612 T1 25 0

24379861 T1 8 0

24379888 T1 32 0

24380630 T1 1 0

24380967 T1 8 0

24381185 T1 4 0

24381481 T1 5 0

24381562 T1 9 0

24382991 T2 4 0

24383319 T1 33 0

24384450 T1 27 0

24386607 T1 2 0

24386625 T1 2 0

24386842 T1 20 0

24388085 T1 2 0

24388312 T1 2 0

24388536 T1 3 0

24388549 T1 1 0

24388651 T1 4 0

24388657 T1 6 0

24388665 T1 9 0

24388670 T1 11 0

24388945 T1 6 0

24389305 T1 6 0

24389325 T1 4 0

24390064 T1 8 0

24390306 T1 16 0

24390514 T1 4 0

24390525 T1 15 0

24390959 T2 4 0

24392597 T1 14 0

24392794 T1 16 0

24392941 T1 12 0

24393178 T1 8 0

24393414 T2 2 0

24393795 T1 2 0

24394344 T2 4 0

24394362 T1 9 0

24394425 T1 5 0

24394857 T2 7 0

24394867 T1 4 0

24395215 T1 2 0

24395357 T1 8 0

24395418 T1 2 0

24395544 T1 10 0

24395998 T1 18 0

24396087 T1 3 0

24396255 T1 10 0

24397102 T1 3 0

24398016 T2 13 0

24398114 T1 6 0

24398263 T2 1 0

24399103 T1 11 0

24399467 T1 48 0

24400551 T1 8 0

24400586 T1 8 0

24400662 T1 4 0

24400951 T1 4 0

24401086 T1 4 0

24401195 T1 13 0

24401298 T1 20 0

24401326 T1 7 0

24401391 T2 7 0

24401409 T1 1 0

24401442 T2 13 0

24401686 T3 5 0

24402247 T1 5 0

24402316 T2 3 0

24402889 T1 1 0

24404375 T1 2 0

24406005 T1 2 0

24406399 T1 1 0

24406534 T1 7 0

24406978 T1 11 0

24407671 T1 8 0

24407786 T1 1 0

24407915 T1 7 0

24408300 T1 1 0

24408497 T1 2 0

24409521 T1 4 0

24409884 T1 4 0

24409887 T1 2 0

24410149 T1 1 0

24410240 T3 3 0

24410682 T1 4 0

24410686 T1 9 0

24410729 T1 2 0

24411213 T1 3 0

24411345 T3 7 1

24411588 T1 11 0

24411654 T1 7 0

24412888 T1 24 0

24413934 T1 5 0

24414011 T1 1 0

24414791 T2 1 1

24414975 T1 5 0

24417449 T1 6 0

24417604 T1 1 0

24417662 T2 8 0

24417743 T1 4 0

25400232 T1 14 0

25400255 T1 7 0

25400942 T1 1 0

25401334 T1 42 0

25401713 T1 25 0

25401729 T1 49 0

25402050 T1 10 0

25403132 T2 9 0

25403775 T1 1 0

25403862 T1 4 0

25403995 T1 2 0

25404645 T1 5 0

25405046 T2 9 0

25405056 T1 8 0

25405361 T1 8 0

25405441 T1 1 0

25405469 T1 4 0

25405471 T1 2 0

25406354 T1 3 0

25407215 T1 1 0

25407487 T1 10 0

25407881 T1 9 0

25407929 T1 3 0

25409405 T1 1 0

25409425 T1 3 0

25410837 T1 9 0

25411301 T1 2 0

25411445 T1 44 0

25412161 T1 10 0

25412300 T1 2 0

25413052 T1 26 0

25413575 T1 2 0

25413835 T1 5 0

25413896 T1 3 0

25414483 T1 18 0

25414504 T1 2 0

25414588 T2 4 0

25414623 T1 5 0

25415325 T1 5 0

25415338 T1 1 1

25415382 T1 4 0

25415568 T1 1 0

25415662 T1 14 0

25415784 T1 8 0

25415790 T1 2 0

25415868 T1 25 0

25416237 T2 1 0

25416660 T1 3 0

25416775 T1 2 0

25417100 T2 8 8

25417540 T1 9 0

25417577 T1 4 0

25417663 T1 6 0

25417666 T1 9 0

25417762 T1 4 0

25417908 T1 5 0

25418942 T1 4 0

25420306 T1 4 0

25420463 T1 20 0

25421770 T2 32 0

25421877 T1 4 0

25421910 T1 8 0

25421980 T1 3 0

25422009 T1 6 0

25422222 T2 3 0

25423339 T2 4 0

25423373 T1 9 0

25423457 T1 7 0

25423585 T1 7 0

25423593 T1 2 0

25424165 T1 4 0

25424649 T1 3 0

25424690 T1 22 0

25424896 T1 11 0

25425357 T1 6 0

25425454 T1 11 0

25425516 T1 3 0

25425899 T1 6 0

25426156 T1 4 0

25426305 T1 15 0

25426563 T1 10 0

25427321 T1 3 0

25427408 T1 2 0

25427470 T1 5 0

25427517 T1 40 0

25427596 T1 4 0

25427635 T1 4 0

25428794 T2 9 0

25430419 T1 3 0

25430628 T1 4 0

25431220 T1 4 0

25432455 T1 8 0

25432465 T1 7 0

25432869 T1 1 0

25433143 T1 7 0

25433278 T1 2 0

25433737 T1 9 0

25433759 T1 7 0

25434934 T1 7 0

25434951 T1 10 0

25434952 T1 4 0

25435105 T1 4 0

25435441 T1 3 0

25435907 T1 1 0

25436526 T1 7 0

25436629 T1 1 0

25437558 T1 8 0

25438216 T2 1 0

25438470 T1 2 0

25439247 T1 7 0

25439444 T1 3 0

25439508 T1 2 0

25439730 T1 3 0

25440822 T1 3 0

25441330 T1 1 0

25441993 T1 8 0

25442450 T2 3 0

25442893 T1 7 0

25443188 T1 2 0

25443424 T1 3 0

25443533 T1 3 0

25444430 T1 6 0

25444732 T1 11 0

25444825 T1 21 0

25444983 T2 19 0

25445093 T1 9 0

25445304 T1 12 0

25445360 T2 17 0

25445383 T1 1 0

25445594 T1 8 0

25445730 T1 3 0

25445918 T1 3 0

25446607 T1 9 0

25446743 T1 6 0

25446758 T1 2 0

25447249 T1 62 0

25447832 T1 5 0

25448404 T1 12 0

25449303 T2 13 0

25449546 T1 4 0

25449640 T2 2 0

25449817 T1 6 0

25451908 T1 6 0

25452060 T1 5 0

25452562 T1 7 0

25452777 T1 4 0

25453519 T1 14 0

25453684 T1 15 0

25454085 T1 13 0

25454126 T1 4 0

25454662 T1 12 0

25455672 T1 6 0

25456071 T1 6 0

25456703 T1 12 0

25456968 T1 10 0

25458188 T1 5 0

25458572 T1 7 0

25458597 T2 9 0

25459033 T2 12 0

25462270 T1 8 0

25462367 T1 3 0

25462429 T1 5 0

25464035 T1 1 0

25464077 T1 1 0

25464484 T1 2 0

25464704 T1 3 0

25465439 T1 15 0

25467319 T1 7 0

25467384 T3 4 0

25467604 T1 5 0

25468058 T1 1 0

25471138 T1 30 0

25471953 T2 5 0

25471960 T1 4 0

25472849 T1 7 0

25472932 T1 1 0

25473868 T2 1 0

25474385 T1 15 0

25474598 T1 5 0

25475210 T1 26 0

25475565 T1 4 0

25477630 T1 5 0

25478599 T1 10 0

25479020 T1 10 0

25479233 T3 14 0

25479509 T1 18 0

25479674 T1 4 0

25480205 T1 26 0

25480678 T1 6 0

25480773 T1 2 0

25480836 T1 2 0

25481358 T2 3 0

25481973 T1 22 0

25482403 T1 5 1

25483792 T1 3 0

25484462 T1 6 0

25484505 T1 6 0

25485411 T1 11 0

25485819 T1 5 0

25486522 T1 22 0

25487142 T1 2 0

25487311 T1 2 0

25487417 T1 22 0

25487815 T1 9 0

25487997 T1 2 0

25488024 T1 5 0

25488065 T1 5 0

25488347 T1 14 0

25488785 T1 3 0

25488965 T1 1 0

25489613 T1 6 0

25492854 T1 10 0

25493465 T1 9 0

25493779 T1 16 0

25494935 T2 1 0

25494999 T1 28 0

25495158 T1 10 0

25496043 T1 18 0

25497811 T1 9 0

25498133 T1 3 0

25498879 T1 6 0

25499335 T1 10 0

25499461 T1 21 0

25502013 T1 3 0

25502101 T1 3 0

25502239 T1 3 0

25502307 T1 1 0

25502358 T1 3 0

25502565 T1 23 0

25503482 T1 23 0

25504156 T1 28 1

25504571 T1 4 0

25504906 T1 4 0

25505035 T2 1 0

25505516 T1 11 0

25505588 T1 2 0

25506120 T3 6 0

25506220 T1 8 0

25506271 T2 11 0

25506355 T1 7 0

25506366 T1 3 0

25506434 T1 11 0

25506638 T1 11 0

25506829 T1 4 0

25507774 T1 1 0

25508071 T1 1 0

25508813 T2 1 1

25508820 T1 17 0

25508850 T1 14 0

25508861 T3 45 0

25509143 T1 13 0

25509255 T2 5 0

25509279 T2 5 3

25509313 T2 10 0

25509633 T1 1 0

25509811 T1 23 0

25510043 T1 17 0

25510375 T3 5 0

25510652 T1 8 0

25510988 T1 1 0

25511921 T1 2 0

25512633 T2 1 0

25513443 T1 2 0

25513639 T1 3 0

25514665 T1 16 0

25515000 T1 3 0

25515239 T1 4 0

25515574 T1 15 0

25516033 T1 8 0

25516187 T1 2 0

25516533 T1 21 0

25517814 T1 2 0

25518027 T2 3 0

25518233 T2 10 1

25518385 T1 8 0

25518783 T1 11 0

25518845 T2 7 0

25520337 T1 11 0

25521055 T1 5 0

25521068 T1 1 0

25521170 T2 6 0

25521419 T1 7 0

25521722 T1 4 0

25521835 T1 2 0

25523028 T1 9 0

25524921 T1 4 0

25524970 T2 3 2

25525320 T1 23 0

25525968 T1 4 0

25526116 T1 4 0

25526149 T1 9 0

25526209 T1 3 0

25526327 T1 7 0

25526342 T1 8 0

25527999 T1 10 0

25528021 T1 15 0

25528031 T1 38 0

25528330 T1 22 0

25528938 T1 8 0

25528964 T1 5 0

25529003 T2 13 0

25529619 T2 4 0

25530172 T1 1 0

25530498 T1 11 1

25530519 T1 1 0

25531635 T1 1 0

25531689 T1 4 0

25531786 T1 19 0

25533123 T1 3 0

25533211 T1 3 0

25533601 T1 2 0

25533615 T1 1 0

25533856 T1 2 0

25534086 T1 5 0

25534094 T1 2 0

25534122 T1 4 0

25534179 T1 4 0

25534607 T1 9 0

25534964 T2 11 0

25535336 T2 8 0

25535419 T1 1 0

25535764 T1 4 0

25535810 T1 2 0

25535952 T1 4 0

25536507 T1 10 0

25536973 T1 11 0

25537401 T3 4 0

25537565 T1 4 0

25537737 T1 10 0

25537814 T1 4 0

25537820 T1 21 0

25537939 T2 5 0

25538075 T1 7 0

25538592 T2 44 2

25538734 T1 60 0

25538815 T1 6 0

25539004 T1 17 0

25539313 T1 15 0

25539783 T1 12 0

25540142 T1 2 0

25541972 T1 9 0

25542984 T1 1 0

25544482 T1 6 0

25544780 T2 1 0

25545207 T1 4 0

25545215 T1 5 0

25545546 T1 4 0

25546238 T1 2 0

25546294 T1 24 0

25547639 T3 8 0

25548262 T1 1 0

25548302 T1 4 0

25548560 T3 10 0

25548721 T2 19 1

25550366 T1 3 0

25550477 T1 18 0

25550557 T1 20 0

25550912 T1 5 0

25551091 T2 5 0

25551292 T1 3 0

25551404 T1 4 0

25551565 T1 9 0

25551780 T1 4 0

25552109 T1 8 0

25552176 T1 5 0

25552202 T2 11 1

25552255 T1 1 0

25552602 T2 2 0

25553286 T1 6 0

25554345 T2 13 0

25554448 T1 8 0

25554478 T1 9 0

25554536 T1 76 4

25554636 T1 6 0

25554639 T1 9 0

25554690 T1 1 0

25556213 T1 3 0

25556360 T1 7 0

25556426 T1 4 0

25556455 T1 6 0

25556860 T1 13 0

25556900 T1 15 0

25556917 T3 12 0

25557131 T1 2 0

25557518 T1 7 0

25557597 T1 7 0

25557600 T1 1 0

25557696 T1 2 0

25557776 T1 11 0

25557935 T2 5 0

25558822 T1 1 0

25559395 T1 2 0

25559904 T2 12 0

25560725 T1 4 0

25561206 T1 2 0

25563153 T1 4 0

25563159 T1 6 0

25563226 T1 8 0

25563451 T1 3 0

25563696 T1 2 0

25564052 T2 3 0

25564199 T2 12 0

25564594 T1 13 0

25564648 T1 4 0

25565463 T1 2 0

25565699 T1 5 0

25565759 T1 7 0

25565880 T2 10 0

25566022 T1 11 0

25566031 T1 4 0

25567054 T1 2 0

25567180 T2 8 0

25568142 T1 5 0

25568158 T1 1 0

25568938 T2 2 0

25569000 T1 10 0

25569448 T2 4 0

25570181 T1 1 0

25571547 T1 3 0

25571913 T1 7 0

25572196 T1 2 0

25572217 T1 4 0

25572263 T1 6 0

25572806 T1 2 0

25572853 T1 5 0

25573118 T1 1 0

25573139 T1 8 0

25573225 T1 6 0

25573498 T1 5 0

25574084 T1 8 0

25574235 T1 2 0

25574821 T1 3 0

25575199 T1 4 0

25575247 T1 3 0

25577549 T1 9 0

25577615 T1 2 0

25577805 T1 5 0

25578388 T2 26 0

25578393 T1 6 0

25578535 T2 4 0

25578549 T1 14 0

25580137 T1 12 0

25580363 T2 2 0

25581710 T1 5 0

25581833 T1 1 0

25582297 T1 8 0

25583001 T1 25 0

25583308 T1 2 0

25583313 T1 4 0

25584831 T1 3 0

25585749 T1 6 0

25585795 T1 18 0

25587065 T1 8 0

25587536 T1 13 0

25588078 T1 47 0

25588416 T1 3 0

25589108 T1 5 0

25589874 T2 25 0

25589988 T1 16 0

25589991 T1 4 0

25590292 T1 4 0

25590606 T1 4 0

25590773 T1 5 0

25591072 T2 19 1

25591170 T1 6 0

25591227 T1 5 0

25592217 T1 5 0

25592232 T1 2 0

25592512 T1 39 0

25592541 T1 10 0

25592574 T1 12 0

25593261 T1 13 0

25593703 T1 7 0

25593905 T3 3 0

25594423 T1 5 0

25594424 T1 1 0

25595148 T2 2 0

25595278 T1 4 0

25595316 T1 6 0

25595720 T1 3 0

25595745 T1 13 0

25595777 T2 1 0

25595788 T1 6 0

25596137 T1 4 0

25596241 T1 21 0

25596394 T1 3 0

25596792 T1 9 0

25596884 T1 23 0

25597172 T1 9 0

25597661 T1 1 0

25598165 T1 8 0

25598174 T1 4 0

25598195 T3 7 0

25598206 T1 1 0

25598228 T1 6 0

25598343 T1 6 0

25598738 T1 3 0

25598771 T1 6 0

25599488 T1 15 0

25599735 T2 8 0

25599875 T1 19 0

25599937 T1 11 0

25600038 T1 2 0

25600090 T1 17 0

25600123 T3 1 0

25600130 T1 8 0

25600177 T1 4 0

25601252 T1 3 0

25601253 T1 15 0

25601873 T1 14 0

25602074 T1 4 0

25602076 T1 5 0

25602106 T1 5 0

25602130 T1 8 0

25602154 T1 8 0

25602701 T3 2 0

25602981 T1 6 0

25603606 T3 2 0

25604490 T1 11 0

25604868 T1 11 0

25604900 T1 20 0

25605364 T1 11 0

25605554 T1 10 0

25605862 T1 5 0

25606640 T1 2 0

25606891 T3 1 0

25607127 T1 9 0

25607193 T3 6 0

25607316 T1 6 0

25607667 T1 3 0

25607739 T1 2 0

25607763 T1 23 3

25609528 T1 4 0

25609940 T1 14 0

25609989 T1 6 0

25610462 T1 6 0

25610818 T1 2 0

25610837 T1 11 0

25610903 T1 5 0

25611850 T1 3 0

25611965 T1 2 0

25612104 T1 1 0

25612477 T1 4 0

25612792 T1 7 0

25613513 T1 5 0

25613567 T1 3 0

25613651 T1 16 0

25613673 T2 38 0

25613839 T1 3 0

25614433 T3 6 0

25614516 T1 2 0

25614693 T1 2 0

25614741 T1 4 0

25615149 T1 7 0

25615266 T1 1 0

25615719 T1 23 0

25615807 T1 2 0

25615990 T2 7 0

25616047 T1 11 0

25616116 T1 5 0

25616262 T1 17 0

25616474 T1 21 0

25616609 T1 1 1

25616796 T1 8 0

25617005 T1 2 0

25617086 T1 6 0

25617264 T1 2 0

26600647 T2 11 0

26601026 T1 43 0

26601354 T1 1 0

26601382 T1 22 0

26601455 T1 9 0

26601479 T1 4 0

26601595 T1 22 0

26601601 T1 4 0

26601609 T1 3 0

26601622 T1 7 0

26601646 T1 6 0

26601704 T2 7 1

26602641 T1 2 0

26602701 T1 3 0

26602922 T1 17 0

26603056 T1 4 0

26603162 T1 1 0

26603206 T1 5 0

26603581 T1 28 0

26604057 T1 6 0

26604242 T2 5 0

26604324 T1 6 0

26604435 T1 5 0

26605680 T2 40 1

26605897 T1 6 0

26606053 T1 6 0

26606077 T1 50 0

26606398 T1 10 0

26606434 T1 31 0

26606782 T2 5 4

26607132 T1 3 0

26607766 T1 29 1

26608837 T1 9 0

26609275 T1 28 0

26609463 T1 3 2

26609671 T1 2 0

26610702 T1 6 2

26611317 T1 5 0

26611400 T1 3 0

26611418 T1 10 0

26611421 T1 4 0

26611472 T1 9 0

26611481 T1 2 0

26611484 T1 10 0

26611501 T1 7 0

26611502 T1 9 0

26611510 T1 1 0

26612262 T1 15 0

26612572 T1 1 0

26613318 T1 13 0

26613329 T1 2 0

26613420 T1 5 0

26613450 T1 7 0

26613592 T1 4 0

26614149 T1 1 0

26614791 T1 11 0

26615071 T1 13 0

26615291 T3 13 0

26615484 T1 4 0

26615486 T1 7 1

26615521 T2 10 0

26615833 T1 12 0

26615847 T3 10 0

26616124 T2 13 0

26617887 T1 8 0

26617900 T1 26 0

26617974 T1 12 0

26618407 T1 38 0

26618554 T1 14 0

26619468 T3 2 0

26619693 T3 12 0

26620274 T1 14 0

26620617 T1 5 0

26620767 T1 2 0

26621097 T1 13 0

26621561 T1 2 0

26621807 T3 27 2

26623034 T1 2 0

26623149 T1 5 0

26623174 T1 7 0

26623477 T1 15 0

26623820 T3 12 3

26623974 T1 5 0

26623977 T1 4 0

26623985 T1 4 0

26624194 T1 23 0

26624636 T1 13 0

26624923 T2 10 0

26624932 T1 2 0

26624937 T1 7 0

26624950 T1 8 0

26625425 T1 15 0

26626118 T2 5 0

26626221 T1 2 0

26626469 T2 1 0

26626651 T2 7 0

26627665 T1 25 0

26627682 T1 3 0

26627841 T1 7 0

26628222 T1 2 0

26629420 T1 6 0

26629680 T1 3 0

26629734 T1 5 0

26629817 T1 5 0

26630646 T1 1 0

26631301 T3 2 0

26631594 T1 3 0

26632144 T1 7 0

26632417 T1 5 0

26632486 T1 35 0

26632644 T1 14 0

26632795 T1 3 0

26633134 T1 2 0

26633205 T1 1 0

26633283 T1 13 0

26633520 T1 3 0

26633560 T1 18 0

26633685 T1 8 0

26633752 T1 9 0

26634435 T1 4 0

26636272 T1 14 0

26636466 T1 12 0

26636502 T1 16 0

26636807 T1 11 0

26636858 T1 11 0

26636922 T3 6 0

26636967 T1 20 0

26637175 T1 9 0

26637282 T1 36 0

26637528 T1 11 0

26637664 T1 4 0

26637689 T2 26 0

26637777 T2 2 0

26638665 T2 13 0

26638839 T1 1 0

26639428 T2 27 0

26639727 T1 38 0

26639885 T1 7 0

26640059 T1 5 0

26640140 T1 5 0

26640312 T1 23 0

26640440 T1 11 0

26640501 T1 11 0

26640808 T1 7 0

26640811 T1 8 0

26640862 T1 6 0

26641004 T1 8 0

26641056 T1 3 0

26641080 T3 1 0

26641111 T1 6 0

26641178 T1 5 0

26642032 T1 42 0

26642758 T1 5 0

26643373 T1 2 0

26645108 T3 1 0

26645348 T2 11 0

26645358 T1 22 0

26645485 T1 4 0

26645563 T1 21 0

26645981 T1 8 0

26646268 T1 9 0

26646318 T1 8 0

26646775 T1 3 0

26647428 T1 9 0

26647779 T1 3 0

26648232 T1 20 0

26648553 T1 6 0

26648773 T1 13 0

26648870 T1 19 0

26648881 T1 14 0

26649086 T1 19 0

26649163 T1 6 0

26649209 T1 6 0

26649685 T2 8 0

26649807 T1 5 0

26650228 T4 3 0

26650358 T1 21 0

26650519 T1 18 0

26650553 T1 27 0

26650639 T4 18 0

26650640 T1 9 0

26651192 T1 5 0

26651951 T1 6 0

26651953 T3 5 0

26652571 T1 3 0

26652610 T1 1 0

26652938 T1 17 0

26653027 T1 9 0

26653273 T1 1 0

26653355 T1 5 0

26653387 T1 3 0

26653561 T1 3 0

26653912 T1 8 0

26654025 T2 11 0

26654426 T1 49 0

26654466 T1 35 0

26654678 T1 43 0

26654751 T1 10 0

26654914 T1 15 0

26654929 T2 17 0

26655077 T1 28 0

26655093 T1 4 0

26655240 T1 4 0

26655251 T1 10 0

26655318 T3 8 0

26655467 T1 2 0

26655641 T1 8 0

26655774 T1 2 0

26655923 T1 1 0

26656333 T3 3 0

26657265 T1 21 0

26657711 T3 15 0

26658363 T1 3 0

26658450 T1 21 0

26658868 T2 11 0

26659205 T1 2 0

26659257 T1 14 0

26659781 T2 2 0

26660373 T3 2 0

26661085 T2 5 0

26661596 T3 8 0

26661954 T1 3 0

26662019 T1 5 0

26662364 T1 5 0

26663214 T1 23 0

26663337 T3 11 0

26663514 T1 3 0

26663671 T1 2 0

26663825 T1 12 0

26664322 T1 4 0

26664434 T1 3 0

26665090 T1 12 2

26665112 T1 20 0

26665152 T1 8 0

26665262 T1 13 0

26665445 T1 10 0

26665460 T1 2 0

26665490 T1 3 0

26665532 T1 13 0

26665634 T1 2 0

26665712 T1 6 0

26665907 T1 4 0

26666402 T1 3 0

26666432 T1 6 0

26666579 T3 8 0

26666805 T1 6 0

26667591 T1 9 0

26667594 T1 28 0

26667609 T1 4 0

26667829 T1 2 0

26668227 T1 4 0

26668438 T1 22 0

26668995 T1 3 0

26669106 T1 2 0

26669316 T1 7 0

26669431 T1 11 0

26669653 T1 4 0

26669877 T1 24 0

26670170 T2 7 0

26670176 T1 2 0

26670557 T1 9 0

26670678 T2 39 0

26671150 T1 22 0

26671198 T2 13 0

26671203 T1 6 0

26671239 T3 7 0

26671357 T1 18 0

26671360 T1 21 0

26671372 T1 20 0

26671404 T3 4 0

26671431 T1 14 0

26671512 T1 14 0

26671720 T1 11 0

26671767 T3 24 0

26673043 T2 15 0

26673434 T1 4 0

26673618 T1 17 0

26673773 T1 17 0

26676756 T1 2 0

26677613 T2 4 0

26677748 T1 5 0

26677994 T1 17 0

26678209 T1 16 0

26678395 T1 3 0

26678399 T1 26 0

26678610 T1 13 0

26678616 T1 3 0

26678704 T1 1 0

26678737 T1 12 0

26678770 T2 11 0

26679346 T2 11 3

26679717 T1 16 0

26680099 T2 2 0

26680159 T2 6 0

26680253 T1 1 0

26680512 T1 11 0

26680617 T1 47 0

26682094 T1 2 0

26682281 T1 4 0

26682527 T1 1 0

26682858 T1 28 0

26682887 T2 19 0

26682955 T4 6 0

26682966 T1 6 0

26682993 T1 21 0

26683094 T1 6 0

26683312 T1 5 0

26683315 T1 18 0

26683342 T1 10 0

26684200 T1 5 0

26684504 T1 4 0

26684888 T1 17 0

26685020 T1 5 0

26685084 T1 16 0

26685116 T1 9 0

26685152 T2 8 0

26685287 T1 8 0

26685423 T1 1 0

26685672 T1 17 0

26685785 T1 5 0

26685941 T1 4 0

26686046 T1 2 0

26686152 T3 2 0

26686318 T2 2 0

26686467 T1 8 0

26687503 T1 24 0

26688362 T1 14 0

26688458 T1 24 0

26688467 T1 3 0

26689179 T1 16 0

26689849 T1 3 0

26690027 T1 27 0

26690472 T1 1 0

26690667 T2 5 0

26691205 T1 4 0

26691271 T1 3 0

26691325 T1 5 0

26691763 T1 12 0

26691895 T3 6 0

26692092 T1 2 0

26692632 T1 3 0

26693166 T4 24 17

26693402 T1 20 0

26693499 T1 46 0

26693735 T2 10 0

26694160 T1 26 0

26694446 T1 7 0

26694680 T1 6 0

26694772 T1 27 0

26694874 T1 6 0

26695402 T1 3 0

26695912 T1 4 0

26695936 T1 6 1

26696283 T2 19 0

26696712 T1 22 0

26696777 T1 33 0

26697568 T1 2 0

26697630 T1 4 0

26698518 T1 11 0

26698566 T2 24 0

26699181 T1 7 0

26699862 T1 5 0

26700505 T1 6 0

26700784 T1 34 0

26703582 T1 1 0

26704511 T1 6 0

26704817 T1 5 0

26704845 T1 1 0

26704861 T1 15 0

26704875 T1 22 0

26706031 T1 16 0

26706281 T1 15 0

26706356 T1 16 0

26706382 T1 14 0

26706497 T2 28 0

26706797 T2 6 0

26706804 T3 20 0

26706977 T1 3 0

26707217 T1 2 0

26707362 T1 3 0

26707382 T1 7 0

26707433 T1 5 0

26707506 T2 27 1

26707690 T1 3 0

26707797 T1 4 0

26707834 T1 2 0

26707870 T1 2 0

26708027 T1 10 0

26708285 T2 5 0

26708470 T1 15 0

26708484 T1 19 0

26708588 T1 29 0

26708626 T1 4 0

26708635 T3 13 0

26708955 T1 6 0

26709076 T1 3 0

26709135 T1 7 0

26709166 T1 5 0

26709177 T1 9 0

26709257 T1 3 0

26709316 T1 13 0

26709569 T1 37 0

26709727 T1 3 0

26710075 T1 7 0

26710434 T1 5 0

26710544 T1 6 0

26710975 T1 35 0

26711074 T1 44 0

26711290 T1 10 0

26711355 T1 5 1

26711379 T1 4 0

26711519 T1 30 0

26711637 T2 22 0

26711705 T1 5 0

26711855 T1 8 0

26711951 T1 4 3

26712473 T1 14 0

26712718 T1 3 0

26712747 T1 4 0

26712750 T1 4 0

26713121 T1 25 0

26713442 T1 3 0

26713619 T1 14 0

26713753 T1 21 0

26713947 T1 5 0

26713973 T2 14 0

26714681 T1 22 0

26714795 T1 19 0

26714923 T1 9 0

26715107 T1 20 0

26715445 T1 12 1

26716016 T3 17 1

26716108 T1 4 0

26716236 T1 5 0

26716255 T1 2 0

26716363 T3 1 0

26716414 T1 1 0

26716636 T1 10 0

26716665 T3 36 0

26716928 T1 4 0

26717531 T1 29 0

26717581 T3 13 0

26717600 T1 4 0

26717893 T1 10 0

26718159 T1 43 0

26718196 T1 10 1

26718398 T1 4 0

26718441 T1 4 0

26718661 T1 21 0

26718892 T1 4 0

26719239 T1 13 0

26719245 T2 12 0

26719365 T1 2 0

26719525 T1 25 0

26719771 T1 24 0

26719787 T1 20 0

26719817 T3 15 0

26719822 T1 32 0

26719834 T1 13 0

26719901 T1 15 0

26720023 T1 14 0

26720119 T1 4 0

26720454 T1 23 0

26720711 T1 7 0

26720730 T3 16 0

26720938 T2 3 0

26720957 T1 11 0

26721074 T1 24 0

26721098 T1 14 0

26721132 T1 36 0

26721311 T2 7 0

26721431 T1 2 2

26722030 T1 16 0

26722509 T1 9 0

26722661 T1 34 0

26722826 T1 14 0

26723449 T1 75 0

26723688 T1 2 0

26724018 T1 5 0

26724058 T1 4 0

26724064 T1 5 0

26724190 T3 11 0

26724359 T1 2 0

26724629 T3 14 0

26727431 T2 4 0

26727854 T1 11 0

26728846 T2 6 0

26730766 T1 5 0

26731016 T1 7 0

26731110 T2 5 0

26731129 T3 2 0

26731135 T1 6 0

26731190 T1 8 0

26731290 T1 2 0

26731336 T1 4 0

26731642 T1 8 0

26731645 T1 4 0

26731850 T1 2 0

26732006 T1 1 0

26732022 T1 9 0

26732036 T3 9 0

26732232 T1 7 0

26732322 T1 3 0

26732449 T4 2 0

26732474 T1 14 0

26733659 T2 31 0

26734832 T3 38 0

26736222 T2 1 1

26737094 T1 11 0

26737309 T2 8 0

26744323 T1 10 0

26745543 T1 3 0

26746537 T1 2 0

26818939 T1 36 0

29097633 T3 10 0

29099923 T1 3 0

29107360 T1 10 0

29107439 T1 16 0

29108153 T3 1 0

29109021 T2 19 0

29109360 T1 6 0

29110159 T1 2 0

29110921 T1 12 0

29111561 T1 6 0

29112437 T1 3 0

29113041 T1 4 0

29113611 T1 5 0

29114533 T1 7 0

29114829 T1 1 0

29115818 T1 3 0

29116026 T1 4 0

29116290 T1 1 0

29116291 T1 2 0

29117161 T1 4 0

29117185 T1 2 0

29117607 T1 6 0

29118417 T1 2 0

29118770 T1 13 0

29118888 T1 3 0

29118903 T2 16 0

29119140 T2 1 0

29119933 T1 7 0

29119973 T1 12 0

29120217 T1 3 0

29120251 T1 3 0

29120980 T1 8 0

29121029 T1 3 0

29121242 T2 3 1

29121893 T1 1 0

29121920 T1 5 0

29122856 T1 8 0

29123440 T1 4 0

29123648 T1 3 0

29124742 T1 14 0

29124798 T1 15 0

29125597 T1 1 0

29126115 T1 1 0

29126354 T1 6 0

29126394 T1 1 0

29126795 T1 4 0

29128800 T1 1 0

29130188 T1 17 0

29130930 T1 1 0

29131522 T1 18 0

29131562 T1 3 0

29132046 T2 5 0

29133051 T1 4 0

29134212 T1 19 1

29134232 T1 9 0

29135172 T1 17 0

29135174 T1 40 0

29135224 T1 4 0

29135487 T2 3 0

29135567 T3 5 2

29136057 T1 12 0

29138839 T1 2 0

29139229 T1 7 0

29139904 T2 2 1

29140038 T2 26 0

29140179 T1 2 0

29140773 T1 4 0

29140895 T1 7 0

29141335 T1 4 0

29141817 T2 2 0

29143191 T1 9 0

29144220 T1 3 0

29145069 T1 1 0

29146660 T1 1 0

29147250 T1 12 0

29147256 T2 5 0

29148010 T1 3 0

29148565 T1 10 0

29149911 T3 3 0

29150414 T1 1 0

29155986 T1 1 0

29156459 T1 4 0

29156498 T1 6 0

29159550 T1 7 0

29160813 T2 1 0

29161668 T1 1 0

29161882 T1 1 0

29163399 T2 1 0

29163522 T1 4 0

29163577 T1 3 0

29163772 T2 7 0

29164673 T1 3 0

29165159 T2 3 0

29166277 T1 10 0

29166355 T2 1 0

29167324 T1 9 0

29168089 T1 2 0

29168376 T1 9 0

29169074 T2 2 0

29169865 T1 17 0

29170962 T1 10 0

29172029 T1 4 0

29173711 T2 7 0

29174759 T1 4 0

29175753 T1 3 0

29176036 T1 5 0

29176984 T2 11 0

29178337 T1 6 0

29181381 T1 3 0

29181868 T1 7 0

29182628 T1 1 0

29183770 T2 8 0

29184292 T1 16 0

29185544 T1 3 0

29185978 T1 2 0

29186290 T1 2 0

29186973 T1 2 0

29187104 T1 14 0

29187158 T1 3 0

29187426 T1 2 0

29188706 T1 9 0

29188911 T1 3 0

29189017 T1 5 0

29189800 T1 2 0

29189919 T1 4 0

29190294 T1 1 0

29191290 T1 1 0

29191403 T1 20 0

29191839 T1 3 0

29193484 T1 5 0

29194189 T1 2 0

29194278 T1 23 0

29195224 T2 8 0

29195550 T1 10 0

29195770 T3 1 0

29196098 T1 5 0

29196759 T2 4 0

29196968 T1 9 1

29197334 T1 7 0

29199637 T3 1 0

29199713 T2 2 0

29200384 T1 1 0

29201485 T1 2 0

29201527 T2 4 1

29202648 T1 3 0

29203465 T1 3 0

29204502 T1 3 0

29204657 T1 4 0

29204661 T1 6 0

29205893 T1 1 0

29206275 T1 6 0

29206547 T1 3 0

29206569 T1 4 0

29207228 T1 1 0

29207402 T1 5 0

29208016 T1 2 0

29208198 T1 6 1

29208367 T1 2 0

29209322 T1 6 0

29209371 T1 2 0

29209704 T2 5 0

29210648 T1 2 0

29210940 T2 5 0

29212642 T1 16 0

29213377 T1 3 0

29213569 T1 2 0

29214717 T1 29 0

29214917 T2 14 2

29215505 T1 3 0

29216300 T1 22 0

29216893 T1 2 0

29217140 T1 5 0

29217823 T1 3 0

29217874 T1 6 0

29217978 T1 2 0

29218310 T2 14 0

29218925 T1 13 0

29219425 T1 1 0

29220155 T1 2 0

29221452 T1 2 0

29222088 T2 3 0

29222392 T1 1 0

29222793 T1 13 0

29223679 T2 4 0

29223866 T1 5 0

29223919 T1 1 0

29224214 T2 3 0

29225138 T2 4 0

29225297 T1 14 0

29225576 T1 3 0

29225605 T1 2 0

29225607 T2 4 0

29226682 T1 4 0

29226963 T1 2 0

29227144 T1 7 0

29227168 T2 3 2

29227327 T1 1 0

29227365 T1 8 0

29227415 T1 1 0

29227491 T1 2 0

29228011 T2 17 0

29228168 T1 2 0

29229086 T1 1 0

29229359 T1 3 0

29229428 T1 36 0

29229689 T1 12 0

29230467 T1 2 0

29230943 T1 3 0

29231412 T1 1 0

29231419 T1 3 0

29231472 T1 2 0

29231646 T1 17 0

29232883 T2 24 0

29233131 T3 2 0

29234076 T1 3 0

29235088 T1 2 0

29235252 T1 4 0

29237347 T1 4 0

29237609 T2 24 0

29237676 T1 14 0

29239639 T1 4 0

30000008 T1 2 0

30000140 T1 2 0

30000241 T1 1 0

30001138 T3 4 0

30001170 T1 1 0

30002149 T1 18 1

30002563 T2 38 0

30002642 T1 12 0

30003718 T1 17 0

30004556 T3 12 0

30004904 T1 4 0

30006128 T1 2 0

30006336 T2 5 0

30006903 T2 12 0

30007317 T1 3 0

30007941 T1 5 0

30008153 T1 10 0

30009007 T2 2 0

30009131 T1 1 0

30009808 T1 18 0

30011706 T1 13 0

30012863 T1 1 0

30012960 T1 2 0

30013337 T3 26 0

30013356 T1 15 0

30013424 T1 8 0

30013706 T2 6 0

30014236 T1 2 0

30014717 T1 14 0

30014732 T2 28 2

30015158 T2 2 0

30015165 T1 1 0

30015303 T1 7 0

30016303 T2 35 0

30016452 T1 2 0

30016805 T2 15 1

30018071 T1 10 0

30018131 T1 14 0

30018425 T1 2 0

30018454 T1 12 0

30018552 T1 6 0

30018644 T1 4 0

30020504 T1 2 0

30021038 T2 3 0

30021449 T1 5 0

30021921 T1 3 0

30022115 T1 2 0

30022174 T1 2 0

30022271 T1 3 0

30022303 T1 4 0

30022437 T1 5 0

30022694 T1 1 0

30023202 T1 2 0

30023903 T1 2 0

30023944 T1 12 0

30024075 T3 21 0

30024177 T1 2 0

30024269 T1 23 0

30024493 T1 2 0

30024685 T1 3 0

30025388 T1 2 0

30026444 T1 16 0

30026471 T2 24 0

30026830 T1 2 0

30026993 T2 4 0

30027017 T1 24 0

30027110 T1 3 0

30027191 T1 1 0

30027277 T2 3 1

30027622 T3 5 0

30028354 T1 1 0

30028676 T2 3 0

30028881 T2 10 0

30028897 T1 11 0

30028941 T1 28 0

30029104 T1 4 0

30029290 T1 3 0

30029655 T1 5 0

30029775 T1 2 0

30030298 T1 3 0

30032274 T1 2 0

30032764 T2 1 0

30032856 T1 1 0

30032942 T1 2 0

30033027 T1 22 0

30034286 T1 1 0

30034443 T1 4 0

30034733 T3 2 0

30035142 T1 25 0

30035674 T1 4 0

30035682 T2 6 0

30035901 T1 16 0

30035959 T1 4 0

30036615 T1 2 0

30036717 T1 3 0

30036846 T3 4 1

30037060 T1 5 0

30037187 T1 2 0

30037739 T1 2 0

30037987 T3 1 0

30038159 T1 2 0

30038180 T1 3 0

30038539 T1 6 0

30038566 T1 28 0

30039173 T1 9 0

30039548 T1 8 0

30039802 T2 1 0

30040361 T1 5 0

30040447 T1 1 0

30040718 T2 2 0

30040779 T1 1 0

30041030 T1 1 0

30041499 T1 14 0

30041603 T1 4 0

30041796 T2 4 0

30041804 T1 5 0

30041953 T1 7 0

30042116 T1 3 0

30042143 T1 1 0

30042170 T1 2 0

30042206 T2 24 0

30042208 T1 13 0

30042450 T1 1 0

30042475 T3 4 1

30042733 T1 8 0

30043698 T1 36 0

30043874 T1 3 0

30045084 T1 3 0

30045803 T1 2 0

30046284 T1 7 0

30046623 T1 9 1

30046882 T1 27 0

30046907 T3 10 0

30047081 T1 8 0

30047215 T1 3 0

30048904 T1 1 0

30049053 T1 17 0

30049253 T1 19 0

30049344 T1 4 0

30049910 T1 15 0

30050290 T1 5 0

30050410 T2 7 0

30050604 T1 4 0

30050634 T1 41 0

30050822 T1 7 0

30050974 T1 1 0

30051158 T1 3 0

30051319 T1 7 0

30051438 T1 22 0

30051456 T1 17 0

30051621 T1 2 0

30051734 T1 2 0

30052307 T2 1 0

30052481 T1 2 0

30052489 T1 5 0

30052516 T2 2 0

30052536 T1 1 0

30052967 T1 25 0

30053119 T1 19 0

30053352 T1 7 0

30053555 T1 18 0

30054236 T1 5 0

30054382 T1 2 0

30055287 T3 15 0

30055616 T1 3 0

30056920 T4 42 17

30057515 T3 6 0

30057673 T1 15 0

30058020 T2 6 1

30058236 T1 7 0

30058484 T1 3 0

30058528 T1 2 0

30058771 T1 2 0

30059138 T1 1 0

30059920 T2 3 0

30059981 T1 8 0

30060718 T1 6 0

30061340 T1 3 0

30062101 T2 34 0

30062199 T1 8 1

30062212 T1 9 0

30062961 T3 1 0

30062970 T1 10 0

30063243 T1 9 0

30063568 T1 49 0

30063817 T1 1 0

30064219 T3 28 0

30064819 T1 17 0

30065206 T1 6 0

30065691 T1 2 0

30065906 T1 1 0

30067017 T1 10 0

30067499 T1 4 0

30068265 T1 12 0

30068277 T1 22 0

30073414 T3 4 0

30075443 T1 28 0

30075745 T1 7 0

30075746 T2 9 0

30075848 T1 44 1

30079868 T2 4 0

30085864 T1 13 0

30215711 T3 4 0

31003404 T1 22 0

31046575 T2 4 0

31055287 T1 14 0

31194074 T1 1 0

31196972 T1 56 0

31204295 T1 22 0

32208267 T1 9 0

32252034 T1 13 0

32252913 T3 6 0

32254348 T1 14 0

32255812 T1 8 0

32256395 T1 6 0

32256720 T1 4 0

32256890 T1 4 0

32257283 T3 5 0

32257807 T1 9 0

32257808 T2 3 0

32258329 T1 17 0

32258941 T1 47 0

32258959 T1 24 0

32259233 T1 6 0

32259535 T2 1 1

32260075 T1 4 0

32260619 T1 42 0

32261335 T1 13 0

32261433 T1 41 0

32261678 T1 17 0

32262654 T1 3 0

32263171 T1 17 0

32263172 T1 16 0

32263174 T1 22 0

32263436 T1 1 0

32264925 T1 4 0

32265213 T1 22 0

32265492 T1 1 0

32267137 T1 21 0

32267645 T1 1 0

32267744 T1 5 0

32268174 T1 4 0

32269162 T1 3 0

32269583 T1 24 0

32269744 T1 16 0

32269950 T1 12 0

32270945 T1 1 0

32271277 T1 22 0

32271796 T1 21 0

32272076 T2 9 0

32272105 T1 1 0

32272428 T1 2 0

32274442 T3 15 0

32274761 T1 2 0

32275025 T1 27 0

32275675 T1 5 0

32276095 T1 1 0

32276247 T1 9 0

32276303 T1 9 0

32276502 T1 14 0

32276586 T2 19 0

32276982 T1 12 0

32277216 T1 27 0

32277611 T1 5 0

32278043 T1 10 0

32278102 T1 3 0

32278467 T1 2 0

32279127 T1 10 0

32279129 T1 13 0

32279130 T1 27 0

32279497 T1 9 0

32279502 T1 15 0

32279713 T1 25 0

32281034 T3 3 0

32281182 T1 10 0

32281385 T1 14 0

32281388 T1 24 0

32282187 T1 9 0

32282326 T1 17 0

32282601 T1 11 0

32282933 T1 4 0

32282980 T1 29 0

32283124 T1 18 0

32283883 T1 8 0

32284272 T1 4 0

32284422 T2 25 0

32284890 T1 24 0

32284951 T1 22 0

32285064 T1 18 0

32285490 T2 1 0

32287001 T1 2 0

32287144 T1 44 0

32287278 T1 31 0

32287281 T3 4 0

32287476 T2 12 0

32288201 T1 4 0

32288234 T1 21 0

32288354 T1 35 0

32289699 T1 22 0

32290604 T1 8 0

32290786 T1 3 0

32291056 T1 11 0

32291808 T1 33 0

32292108 T1 13 0

32292163 T1 2 0

32292430 T1 17 0

32292572 T1 15 0

32292888 T1 16 0

32293264 T1 1 0

32293887 T1 10 0

32294055 T1 2 0

32294132 T1 7 1

32294721 T1 4 0

32295173 T1 36 0

32296628 T1 14 0

32296919 T1 7 0

32298183 T1 2 0

32298234 T1 11 0

32298600 T1 25 0

32298602 T1 13 4

32298874 T1 1 0

32299105 T1 22 0

32299106 T1 19 0

32299846 T1 26 0

32299996 T1 25 0

32300290 T1 16 0

32300672 T2 2 0

32300933 T1 13 0

32301019 T1 11 0

32302586 T1 1 0

32302672 T1 15 0

32302869 T1 6 0

32303390 T1 24 0

32303811 T1 24 0

32303956 T1 25 0

32304056 T1 6 0

32304526 T1 20 0

32304532 T1 14 0

32305261 T1 1 0

32305347 T1 24 0

32305352 T1 7 0

32305812 T1 1 0

32306663 T1 2 0

32306840 T1 20 0

32306845 T1 1 0

32306958 T1 30 0

32307189 T1 17 0

32307191 T1 8 0

32307394 T1 23 0

32307507 T1 10 2

32307569 T1 14 0

32307576 T2 3 0

32308278 T2 35 0

32309155 T1 16 0

32310801 T1 8 0

32311110 T1 20 0

32311460 T1 3 0

32311992 T1 11 0

32312342 T1 3 0

32312556 T1 20 1

32312718 T1 32 0

32313259 T1 18 0

32313747 T1 4 1

32314469 T1 12 0

32314795 T1 21 0

32314961 T1 1 0

32315035 T1 14 0

32316474 T2 6 0

32316679 T1 28 0

32316828 T1 28 0

32317384 T1 3 0

32317682 T2 18 0

32317683 T1 20 0

32317961 T2 29 0

32318081 T1 1 0

32318279 T1 1 0

32319721 T1 8 0

32320109 T1 23 0

32321354 T1 13 0

32321380 T1 3 0

32321491 T1 15 0

32321862 T1 16 0

32322198 T2 3 0

32322313 T1 8 1

32322388 T1 9 0

32322542 T1 35 0

32322627 T1 1 0

32322630 T2 10 0

32322864 T1 12 0

32323377 T2 9 0

32323798 T1 1 0

32324281 T2 9 0

32324417 T1 5 0

32326421 T1 21 0

32326422 T1 25 0

32326784 T1 7 0

32327284 T1 3 0

32327441 T2 6 0

32327567 T1 16 0

32328028 T1 49 0

32328085 T1 1 0

32328760 T1 7 0

32328882 T1 2 0

32329093 T1 15 0

32329691 T1 84 0

32329812 T1 5 0

32330069 T1 1 0

32330072 T1 3 0

32331165 T1 13 0

32331382 T1 3 0

32331446 T1 20 0

32331831 T1 11 0

32333180 T1 3 0

32333359 T1 2 0

32333815 T1 29 0

32333990 T1 5 0

32334400 T2 4 0

32334544 T1 6 0

32334662 T2 8 0

32334664 T1 29 0

32335270 T1 3 0

32335755 T1 16 0

32336387 T1 34 0

32337055 T1 28 0

32337840 T1 1 0

32337864 T1 12 0

32338008 T1 1 0

32338202 T1 22 0

32338345 T1 35 0

32338655 T1 25 0

32339326 T1 5 0

32339564 T2 5 0

32339626 T1 21 0

32340114 T1 27 0

32340155 T1 36 0

32340315 T1 15 0

32340389 T1 17 2

32340775 T1 18 0

32341731 T1 16 0

32342715 T1 10 0

32343860 T1 49 0

32344024 T1 13 0

32344028 T1 17 0

32344563 T1 1 0

32345144 T1 6 0

32345804 T1 17 0

32345991 T1 17 0

32346010 T1 18 0

32346198 T1 1 0

32346374 T1 23 0

32346419 T1 1 0

32346818 T1 28 0

32347017 T1 5 0

32347603 T3 3 0

32347711 T1 16 0

32347898 T1 10 0

32348302 T1 21 0

32348632 T1 30 0

32349971 T1 1 0

32350240 T1 2 0

32350306 T1 28 0

32350991 T1 20 0

32351403 T1 23 5

32351407 T1 1 0

32351516 T1 1 0

32352006 T1 11 0

32352007 T1 15 0

32352009 T1 9 0

32352328 T1 2 0

32352526 T1 1 0

32353453 T1 3 0

32354398 T3 9 0

32354596 T1 7 0

32354686 T1 15 0

32355352 T2 4 0

32355978 T1 27 0

32356884 T1 14 0

32357189 T1 3 0

32357247 T1 5 0

32357578 T1 1 0

32358153 T1 28 0

32358154 T1 1 0

32358185 T1 4 0

32358259 T1 2 0

32358458 T1 1 0

32359381 T1 7 0

32359546 T1 3 0

32359960 T2 22 1

32360127 T1 2 0

32360230 T1 20 0

32360836 T2 6 0

32361048 T1 1 0

32362458 T1 24 0

32363573 T1 9 0

32364818 T1 1 0

32364923 T1 18 0

32365140 T1 1 0

32365143 T1 29 0

32365328 T1 23 0

32365890 T1 17 0

32367028 T1 11 0

32367030 T1 4 0

32367108 T1 33 0

32367367 T1 15 0

32367470 T1 15 1

32367504 T1 18 0

32367721 T1 2 0

32367863 T1 23 0

32367864 T1 33 0

32367997 T1 7 0

32368106 T1 1 0

32369014 T2 7 1

32369374 T1 9 0

32370772 T2 16 0

32370850 T1 3 0

32371256 T1 8 0

32371463 T1 13 0

32371749 T1 19 0

32371823 T1 20 0

32372288 T1 23 0

32372325 T1 14 0

32372418 T1 8 0

32372422 T1 4 0

32372427 T1 10 0

32373232 T1 11 0

32373421 T2 1 0

32373713 T1 18 0

32374043 T1 11 0

32374471 T1 8 0

32374592 T1 20 0

32375147 T2 15 0

32375429 T2 10 0

32375431 T1 7 0

32375649 T1 12 0

32375652 T1 2 0

32377466 T1 16 0

32377682 T1 2 0

32377880 T1 29 0

32377883 T1 13 0

32377884 T1 32 0

32378021 T1 1 0

33333515 T1 3 0

33335678 T1 4 0

33335681 T1 8 0

33336109 T1 4 0

33337382 T1 27 0

33338347 T1 1 0

33338352 T1 3 0

33338556 T1 13 0

33338854 T1 26 0

33338994 T1 54 0

33339849 T1 24 5

33339968 T1 35 0

33340156 T1 12 0

33340371 T2 13 0

33340432 T1 17 0

33341373 T1 21 0

33341406 T3 29 0

33341636 T1 1 0

33342102 T1 2 0

33342459 T2 8 0

33342733 T1 14 0

33342736 T1 11 0

33342739 T1 66 0

33342740 T1 7 0

33343074 T1 15 0

33343189 T1 13 0

33343191 T1 20 0

33343655 T1 6 0

33343707 T1 8 0

33344097 T1 12 0

33344100 T1 9 0

33344559 T1 14 0

33345131 T2 2 0

33345293 T1 11 0

33346182 T1 8 0

33346747 T1 13 0

33346845 T2 3 0

33347048 T1 7 0

33347471 T1 1 0

33347737 T1 3 0

33348669 T1 24 0

33349291 T1 7 0

33350143 T1 4 0

33350398 T1 14 0

33350938 T1 13 0

33351424 T1 8 0

33351483 T1 24 0

33352006 T1 12 0

33352007 T1 25 0

33352602 T1 28 0

33352604 T1 7 0

33353157 T1 4 0

33353418 T1 18 0

33353606 T1 3 0

33353875 T1 6 0

33354182 T1 17 0

33354566 T1 24 1

33354669 T1 15 0

33354810 T2 5 0

33354884 T1 13 0

33355498 T1 36 0

33355560 T1 24 0

33355824 T1 22 0

33356151 T1 5 0

33356253 T1 1 0

33356501 T2 13 0

33356697 T1 20 1

33358427 T2 9 0

33358507 T1 25 0

33359061 T1 2 0

33359132 T1 23 0

33359424 T1 21 0

33359454 T1 22 0

33359561 T1 23 0

33359605 T1 13 0

33359964 T1 25 0

33360945 T3 13 0

33361176 T1 43 0

33361613 T1 9 0

33361966 T1 7 0

33364978 T1 22 0

33364983 T1 16 0

33365552 T1 2 0

33365965 T1 1 0

33366166 T1 8 0

33366558 T2 15 0

33366736 T3 23 0

33367425 T1 27 0

33367645 T1 18 0

33367994 T1 2 0

33368271 T1 12 0

33368312 T1 25 0

33368512 T1 11 0

33368781 T1 11 0

33369026 T1 24 0

33369129 T1 1 0

33369471 T1 4 0

33369729 T1 15 0

33370027 T1 9 0

33370232 T1 6 0

33370234 T2 5 1

33371331 T1 4 0

33371784 T1 33 0

33372655 T1 1 0

33373267 T1 17 0

33373380 T1 17 0

33373382 T1 32 0

33373384 T1 23 0

33373630 T1 11 0

33373632 T1 21 0

33373796 T1 13 0

33373942 T1 9 0

33374325 T1 9 0

33374978 T1 6 0

33375267 T1 2 0

33375431 T1 3 0

33375522 T1 4 0

33376498 T2 11 0

33376765 T1 8 0

33377073 T1 30 0

33377439 T1 1 0

33377502 T1 12 0

33377750 T1 10 0

33377894 T1 6 0

33377896 T1 14 0

33377984 T2 18 0

33378115 T1 15 0

33378662 T1 7 0

33379108 T1 6 0

33379820 T1 10 0

33379964 T1 7 0

33380789 T1 4 0

33380856 T1 25 0

33380858 T1 12 0

33381388 T2 7 0

33381713 T1 19 0

33381804 T1 26 0

33381826 T1 15 0

33381921 T1 2 0

33382071 T1 16 0

33382131 T1 11 0

33382375 T1 5 0

33383082 T1 35 0

33383282 T1 4 0

33383730 T1 1 0

33383868 T1 7 0

33384306 T1 4 0

33384307 T1 11 0

33384481 T2 7 0

33384614 T1 22 0

33384996 T1 29 0

33385172 T1 23 0

33385693 T1 1 0

33385913 T1 6 0

33385955 T2 10 0

33386054 T1 58 0

33386265 T1 4 0

33386943 T1 7 0

33388620 T2 11 0

33388785 T2 25 0

33389253 T1 16 0

33389764 T1 11 0

33389808 T1 26 0

33390120 T1 12 0

33390384 T1 13 0

33392205 T1 39 0

33392890 T1 19 0

33392986 T1 2 0

33393002 T1 23 0

33393247 T1 13 2

33393973 T1 7 0

33393976 T1 7 0

33394696 T1 10 0

33394814 T1 4 0

33395555 T2 20 0

33395559 T1 15 0

33395561 T1 16 0

33395564 T1 15 0

33395634 T1 8 0

33395881 T1 11 0

33396625 T2 23 0

33396782 T1 25 0

33396947 T1 14 0

33397310 T1 16 0

33397414 T3 26 0

33397535 T1 10 0

33398134 T1 14 0

33398400 T1 19 0

33398624 T1 3 0

33399882 T1 19 0

33400181 T1 2 0

33400380 T1 40 4

33400563 T1 26 0

33400604 T1 1 0

33401004 T1 39 1

33401599 T1 25 0

33401930 T1 12 0

33402121 T1 6 0

33402537 T1 22 0

33403294 T1 19 0

33403477 T1 19 0

33403778 T1 1 0

33404631 T1 11 0

33404664 T1 6 0

33404957 T1 5 0

33405384 T1 2 0

33405521 T1 1 0

33405824 T1 9 0

33406298 T1 26 0

33407869 T1 1 0

33408173 T1 13 0

33408452 T1 17 0

33408453 T1 4 0

33408756 T1 4 0

33409597 T1 87 0

33410380 T1 14 0

33410702 T1 7 0

33411454 T1 2 1

33412008 T2 11 0

33412088 T1 17 2

33413003 T1 9 0

33414180 T1 18 0

33414217 T1 10 0

33414339 T1 6 0

33414391 T1 1 0

33414584 T2 31 0

33414842 T1 27 0

33414977 T1 21 0

33415791 T1 2 0

33416034 T1 13 2

33416317 T1 26 0

33416505 T2 8 1

33417134 T1 9 2

33417138 T1 15 0

33417753 T1 17 0

33417984 T1 28 0

33418300 T1 32 0

33418440 T3 6 0

33418789 T1 24 0

33418870 T1 8 0

33419547 T1 12 0

33420652 T1 1 0

33421376 T1 1 0

33421582 T1 15 0

33421955 T1 19 0

33422428 T3 8 4

33422431 T1 21 0

33423197 T2 2 1

33423200 T1 1 0

33423377 T1 5 0

33423569 T1 10 0

33423955 T2 4 0

33424068 T1 14 0

33424071 T1 13 0

33426302 T1 13 0

33426304 T2 5 0

33426652 T1 3 0

33426655 T1 1 0

33427008 T2 18 0

33427257 T1 10 0

33427574 T1 29 0

33428017 T1 30 0

33428107 T1 30 0

33428970 T1 4 0

33430309 T2 20 0

33430534 T1 25 0

33431314 T2 12 0

33432706 T1 12 0

33432753 T1 21 0

33432967 T1 21 0

33433807 T1 25 0

33434578 T1 15 0

33434624 T1 3 0

33435012 T1 10 2

33435113 T1 12 0

33435239 T1 5 0

33435293 T1 12 0

33435364 T1 17 0

33435568 T1 14 0

33435572 T1 6 0

33436273 T2 23 0

33436276 T1 2 0

33436350 T1 23 0

33436352 T2 25 0

33436488 T1 1 0

33436816 T1 2 0

33437485 T1 10 0

33438374 T2 14 0

33438880 T1 2 0

33439922 T1 2 0

33439924 T1 21 0

33439970 T1 34 0

33440075 T1 9 0

33440129 T1 3 0

33440369 T1 8 0

33440474 T1 1 0

33440516 T1 9 0

33441090 T1 18 0

33441417 T3 12 2

33442263 T1 12 0

33442538 T1 26 0

33443336 T1 28 0

33444190 T1 6 0

33444253 T1 28 0

33444638 T1 15 0

33444639 T1 19 0

33445067 T1 9 0

33445142 T1 7 1

33445510 T1 33 1

33445704 T1 8 0

33445748 T1 8 0

33445752 T1 19 0

33446185 T3 21 0

33446379 T1 8 0

33446583 T1 24 0

33446750 T2 12 0

33446765 T1 6 0

33447274 T1 12 0

33447556 T1 24 0

33447781 T2 3 0

33448319 T1 18 0

33448751 T1 12 0

33449389 T2 34 0

33449563 T1 1 0

33449630 T1 40 0

33449820 T1 11 0

33449822 T1 22 7

33450978 T1 10 0

33451531 T1 24 0

33451835 T1 50 0

33451909 T1 37 0

33451962 T2 18 0

33452135 T1 11 0

33452470 T1 13 0

33452895 T1 21 0

33453180 T3 10 0

33453235 T1 1 0

33453894 T1 9 0

33453936 T1 22 0

33454234 T1 15 0

33454543 T1 36 0

33454954 T1 18 0

33454966 T1 21 0

33455345 T1 31 0

33455348 T1 23 0

33455407 T1 20 0

33455457 T1 23 0

33455463 T1 7 0

33456817 T1 19 0

33456818 T1 32 0

33457002 T2 30 0

33457167 T1 14 0

33457834 T1 2 0

33457942 T1 6 0

33458126 T1 11 0

33459278 T1 49 0

33460302 T2 17 0

33460832 T1 19 0

33461373 T1 26 0

33461979 T1 4 0

33462085 T1 2 0

33462187 T1 1 0

33462315 T1 7 0

33462438 T1 25 0

33462522 T1 46 0

33462911 T1 10 0

33463465 T1 12 0

33463881 T2 20 0

33464223 T1 8 0

33464619 T3 17 0

33464665 T1 29 0

33464724 T2 19 0

33464864 T1 20 0

33465158 T1 3 0

33465352 T1 38 0

33465497 T1 6 0

33465498 T3 9 2

33465887 T1 3 0

33466058 T1 12 0

33466117 T1 6 0

33466144 T1 1 0

33466305 T1 9 0

33466308 T1 17 0

33467441 T1 6 0

33467579 T1 16 0

33467857 T1 9 0

33468857 T3 15 2

33469029 T1 12 0

33469067 T1 17 0

33469544 T1 27 0

33469665 T1 14 0

33470109 T1 51 0

33470862 T2 15 0

33470878 T1 14 0

33471439 T1 24 0

33473360 T1 31 0

33473655 T1 2 0

33473738 T1 5 0

33474015 T1 16 0

33474770 T1 14 0

33475163 T1 12 0

33475203 T1 19 0

33475377 T3 7 0

33476071 T1 10 0

33476319 T1 16 0

33477148 T1 6 0

33477621 T1 20 0

33477927 T1 13 0

33478220 T1 4 0

33478514 T1 9 0

33478761 T1 35 3

33479411 T2 29 0

33479759 T1 11 0

33479816 T1 9 0

33479908 T1 16 0

33480924 T2 26 2

33481747 T1 15 0

33482009 T1 1 0

33482069 T1 12 0

33483137 T1 18 0

33483230 T1 5 0

33483306 T1 2 0

33483428 T1 1 0

33483433 T1 8 0

33483971 T1 6 0

33484336 T1 25 0

33485051 T1 24 0

33485905 T1 25 0

33486262 T1 24 0

33487096 T2 37 0

33487269 T2 48 3

33487296 T1 8 0

33487298 T1 7 0

33487299 T1 28 0

33487679 T1 7 0

33487918 T2 2 0

33488247 T1 27 0

33488940 T1 22 0

33489029 T1 9 0

33489109 T1 11 0

33489311 T1 18 0

33489908 T1 40 0

33490816 T1 13 0

33491011 T1 14 0

33491142 T1 1 0

33491383 T2 10 0

33491655 T1 1 0

33491899 T2 18 0

33492398 T1 18 3

33492445 T1 16 0

33492490 T2 9 0

33492705 T1 3 0

33492735 T1 22 0

33493059 T1 12 0

33493105 T1 5 0

33493833 T1 6 0

33493971 T1 9 0

33494370 T2 44 0

33494435 T1 8 0

33494680 T2 9 0

33494878 T1 23 0

33496877 T2 8 0

33496880 T1 18 0

33497574 T1 14 0

33497854 T1 16 0

33498118 T1 13 0

33498624 T2 27 1

33498627 T1 14 0

33499084 T3 14 0

33500004 T1 8 0

33500189 T1 26 0

33500192 T1 1 0

33500204 T1 39 0

33501317 T1 16 2

33501589 T1 15 0

33501591 T1 15 0

33502084 T1 12 0

33502283 T1 15 0

33503454 T3 6 0

33503457 T2 26 0

33504763 T1 22 0

33504941 T1 18 0

33505862 T1 1 0

33506728 T1 20 0

33506867 T2 3 0

33508053 T1 23 2

33508511 T2 12 0

33509702 T1 1 0

33510079 T1 8 0

33510136 T1 12 0

33510369 T1 20 0

33510447 T1 17 0

33510550 T1 12 0

33511291 T1 13 0

33511365 T1 24 0

33511905 T1 13 0

33512131 T1 35 0

33512547 T3 1 0

33512663 T1 15 0

33512731 T1 11 0

33512875 T1 14 0

33513188 T1 2 0

33513919 T1 32 0

33514054 T1 24 0

33514206 T1 14 0

33515196 T2 15 0

33515254 T1 18 0

33516109 T1 17 0

33516456 T1 34 0

33516526 T1 3 0

33518266 T1 1 0

33518339 T1 17 0

33518524 T1 28 0

33519150 T1 18 0

33519318 T1 11 0

33520150 T2 13 0

33520499 T1 20 0

33521780 T1 45 0

33522183 T1 38 0

33522830 T1 10 0

33523153 T1 19 0

33523203 T1 11 0

33523204 T1 14 0

33523492 T1 8 0

33524307 T2 9 0

33524506 T1 11 0

33524607 T1 3 0

33524879 T1 9 0

33525069 T1 15 0

33525633 T1 42 0

33525635 T2 18 0

33525638 T1 26 0

33525672 T1 17 0

33526310 T1 2 0

33526619 T1 21 0

33526664 T1 11 0

33527441 T1 16 0

33527447 T1 5 0

33529421 T2 11 0

33529535 T1 13 0

33529538 T1 18 0

33530077 T1 3 0

33530369 T1 2 0

33530778 T1 30 0

33531030 T1 3 0

33531392 T1 11 0

33531538 T1 15 0

33532117 T3 5 0

33532120 T1 4 0

33532217 T1 17 0

33532636 T1 56 0

33532980 T1 7 0

33533107 T1 16 0

33533164 T1 16 0

33533296 T1 18 0

33533762 T1 30 0

33533985 T1 2 0

33534380 T1 5 0

33534693 T1 26 0

33535148 T1 2 0

33535305 T1 20 0

33535541 T1 2 0

33535677 T1 6 0

33536262 T1 16 0

33536805 T1 26 0

33537131 T1 37 0

33537204 T1 15 0

33537394 T1 3 0

33537633 T1 39 0

33537635 T1 19 0

33537749 T1 18 0

33537918 T1 22 0

33538152 T1 35 0

33538577 T1 15 0

33539011 T2 3 0

33539113 T1 13 0

33539241 T1 26 0

33539492 T3 17 0

33539549 T1 22 0

33539926 T1 4 0

33540068 T1 21 0

33540082 T1 24 0

33540257 T1 1 0

33540315 T1 1 0

33540434 T1 27 0

33540519 T1 26 0

33540806 T1 31 0

33540808 T1 27 0

33540892 T1 3 0

33541149 T1 11 0

33541514 T1 37 0

33541842 T1 15 0

33542070 T1 11 0

33542114 T3 5 2

33542596 T1 6 0

33542840 T1 7 0

33542961 T1 17 0

33543125 T1 10 0

33543162 T3 3 0

33543945 T1 21 0

33544062 T1 19 0

33544195 T1 7 0

33544398 T3 26 0

33544402 T1 4 0

33544652 T1 26 0

33544694 T1 2 0

33544764 T3 29 0

33544874 T1 29 0

33545064 T1 1 0

33545122 T1 32 0

34500515 T1 8 0

34501023 T1 2 0

34501230 T1 3 0

34501234 T1 18 0

34501430 T1 10 0

34501927 T1 14 0

34502046 T1 9 0

34502320 T1 6 0

34502796 T1 6 0

34503143 T1 30 0

34503929 T1 9 0

34504433 T1 21 0

34505287 T1 13 0

34505637 T1 2 0

34505820 T1 22 0

34506243 T1 21 0

34506244 T1 17 0

34507136 T1 20 0

34507209 T1 11 0

34507554 T1 1 0

34507813 T1 36 0

34508074 T2 22 0

34508424 T1 5 0

34509355 T1 12 0

34509435 T1 16 0

34510176 T1 18 0

34510998 T1 4 0

34511206 T1 25 0

34511299 T1 10 0

34511920 T1 19 0

34512684 T1 8 0

34512879 T1 10 0

34512882 T1 25 1

34513287 T1 28 0

34513776 T1 5 0

34514563 T1 31 0

34514873 T1 11 0

34515202 T3 20 0

34516046 T1 28 0

34516109 T1 15 0

34516339 T1 32 0

34516635 T3 19 0

34516911 T1 22 0

34517032 T1 20 0

34517756 T3 18 0

34517864 T1 3 0

34518009 T1 11 0

34518914 T1 1 0

34519014 T1 25 0

34519016 T1 48 0

34520209 T1 14 0

34520625 T3 22 0

34520769 T1 12 0

34521115 T2 5 0

34521130 T1 6 0

34521679 T1 1 0

34522059 T1 18 0

34522700 T2 20 0

34522777 T1 30 0

34522905 T1 1 0

34523230 T1 4 0

34523760 T1 22 0

34524500 T1 20 0

34524938 T1 29 0

34525148 T1 7 0

34525255 T1 11 0

34525677 T2 10 0

34525836 T1 21 0

34525891 T1 12 0

34526444 T3 18 0

34526545 T1 20 0

34526668 T1 10 0

34527227 T2 29 0

34527934 T3 30 0

34528044 T1 25 0

34528238 T2 7 0

34528377 T2 28 0

34528623 T1 23 0

34529263 T1 1 0

34529357 T1 34 0

34530017 T1 14 0

34530060 T1 13 0

34530863 T1 22 0

34531277 T1 12 1

34531542 T1 2 0

34532511 T1 18 0

34534130 T1 18 1

34534627 T1 15 1

34534707 T1 13 0

34535026 T3 36 0

34535229 T1 7 0

34536593 T1 29 0

34536791 T1 5 0

34536939 T1 6 0

34537305 T1 27 0

34537309 T1 4 0

34537764 T2 18 0

34537949 T3 9 0

34538013 T1 3 0

34538656 T1 21 0

34538999 T1 19 0

34539665 T1 19 0

34540266 T1 29 1

34540717 T1 32 0

34541551 T1 16 0

34541699 T1 10 0

34543038 T3 6 0

34543157 T1 16 0

34543448 T2 22 1

34543933 T1 12 0

34544315 T1 19 0

34544585 T3 28 1

34544825 T1 17 0

34545093 T1 9 0

34545410 T1 30 0

34545519 T1 5 0

34545989 T1 25 0

34547045 T1 8 0

34547409 T1 37 0

34547618 T2 12 0

34547910 T1 2 0

34548345 T1 28 0

34548403 T1 23 0

34548531 T2 28 0

34548536 T1 8 0

34548688 T1 29 0

34549266 T1 19 0

34549274 T1 17 0

34549874 T1 9 0

34550457 T2 17 1

34550500 T1 18 0

34550802 T1 4 1

34550972 T1 21 0

34551548 T1 2 0

34552045 T1 11 0

34552141 T1 13 0

34552290 T3 2 0

34552427 T1 14 0

34552770 T2 33 0

34552915 T1 36 0

34552929 T1 17 0

34553360 T1 20 0

34553675 T1 26 0

34553888 T1 12 0

34553904 T1 7 0

34554072 T1 13 0

34554607 T3 11 0

34554767 T2 30 1

34555252 T1 23 0

34556250 T1 22 0

34556783 T1 26 0

34557037 T1 24 0

34557868 T1 24 1

34557959 T1 4 0

34558416 T1 19 0

34558417 T1 5 0

34559913 T1 9 0

34561083 T2 39 0

34561090 T1 15 0

34561203 T1 29 0

34561316 T1 11 0

34561611 T1 22 0

34561619 T1 30 2

34562007 T3 21 0

34562668 T2 8 0

34563968 T1 23 0

34564197 T1 29 0

34564203 T1 22 0

34565040 T2 8 1

34565872 T1 3 0

34566109 T1 9 0

34566261 T2 22 0

34566425 T1 6 0

34566630 T1 7 0

34566631 T1 9 0

34566936 T1 25 0

34567304 T1 1 0

34567362 T1 18 0

34567665 T1 20 0

34568281 T1 11 0

34568855 T1 7 0

34569181 T2 26 0

34569213 T2 24 1

34569344 T1 19 0

34569437 T1 26 0

34569481 T2 13 0

34569848 T1 8 0

34570137 T1 15 0

34570251 T3 1 0

34570391 T1 23 0

34570398 T1 22 0

34570400 T1 19 0

34570408 T2 9 0

34570455 T1 16 0

34570679 T2 29 2

34570894 T1 5 0

34571029 T1 1 0

34571145 T1 10 0

34571255 T1 8 0

34572076 T1 37 0

34572393 T1 9 0

34572441 T1 7 0

34572636 T1 22 0

34572738 T1 26 0

34572780 T1 45 0

34573404 T1 10 0

34573866 T3 15 1

34574445 T1 19 0

34574472 T1 12 0

34574589 T1 18 0

34574949 T1 6 0

34575500 T2 33 2

34575535 T1 24 0

34575694 T1 6 0

34576156 T1 20 0

34578201 T2 11 0

34578859 T1 26 0

34578958 T1 23 0

34579415 T2 16 0

34580015 T1 14 0

34580131 T1 17 0

34580153 T1 17 0

34580222 T1 12 0

34582160 T1 13 0

34582197 T1 26 0

34582312 T1 25 0

34583210 T1 6 0

34583316 T1 20 0

34584049 T2 15 0

34584053 T1 10 0

34584063 T1 24 0

34584143 T1 13 0

34584350 T1 19 0

34584508 T2 19 0

34584882 T1 18 0

34585062 T1 13 0

34585223 T1 9 0

34585660 T1 15 0

34585697 T1 16 1

34585719 T3 6 0

34586857 T1 24 0

34587309 T1 14 0

34587404 T1 14 0

34588212 T1 27 0

34588821 T1 11 0

34588929 T1 15 0

34588930 T1 9 0

34589014 T4 1 0

34589162 T1 38 0

34589530 T3 23 0

34589533 T1 13 0

34589762 T1 31 0

34589921 T1 2 0

34589924 T1 4 0

34589970 T1 3 0

34590015 T1 7 0

34590215 T1 13 2

34590941 T2 23 0

34591209 T1 24 0

34591482 T3 5 3

34591936 T1 19 0

34591946 T1 17 0

34592234 T1 18 0

34592272 T1 34 0

34592414 T1 8 0

34592582 T1 27 0

34592774 T1 14 0

34593270 T1 7 0

34593570 T1 2 0

34595386 T1 16 0

34595567 T1 11 0

34596004 T1 8 0

34596012 T2 7 0

34596180 T1 17 0

34596249 T1 23 0

34597279 T1 13 0

34597301 T1 10 0

34597769 T2 28 0

34598210 T2 19 0

34598239 T1 15 0

34598342 T1 9 0

34599177 T1 18 0

34599330 T1 3 0

34599793 T1 28 0

34599831 T1 25 0

34600290 T1 19 0

34600542 T1 11 0

34601741 T1 29 0

34602090 T1 25 0

34602443 T1 18 0

34602481 T1 26 0

34602732 T1 20 0

34602957 T1 22 0

34603282 T1 1 0

34603437 T3 28 0

34603647 T1 5 0

34603705 T1 25 0

34603884 T1 19 0

34604036 T1 19 0

34604423 T2 16 0

34604672 T2 14 0

34605096 T1 13 0

34605099 T1 4 0

34605372 T1 3 0

34605982 T1 21 0

34606744 T1 39 0

34606755 T1 16 0

34607368 T1 18 0

34607372 T1 4 0

34608558 T1 27 0

34609007 T2 8 0

34609074 T1 1 0

34609210 T1 1 0

34609356 T1 3 0

34609601 T1 10 0

34609949 T1 7 0

34611077 T1 14 0

34611177 T1 5 0

34611238 T1 4 0

34611464 T1 13 0

34611990 T1 14 0

34612119 T1 24 0

34612278 T1 9 0

34612610 T1 1 0

34613147 T1 11 0

34613284 T1 16 0

34613501 T2 30 0

34613625 T1 8 0

34613681 T1 29 0

34613696 T1 19 0

34614637 T1 30 0

34616141 T3 8 0

34616229 T1 36 0

34616314 T1 3 0

34616662 T1 15 0

34616815 T1 16 0

34617160 T1 9 0

34617496 T1 10 0

34618051 T1 16 0

34618183 T1 21 6

34618236 T1 4 0

34618246 T1 17 0

34618355 T1 15 0

34618509 T2 16 0

34618931 T1 1 0

34619016 T1 28 0

34619300 T1 9 0

34619818 T2 12 0

34619901 T3 13 0

34620094 T1 11 0

34620272 T1 1 0

34620708 T3 41 0

34621287 T2 7 0

34622703 T2 9 0

34622839 T1 4 0

34622960 T2 14 0

34623678 T1 19 0

34623692 T1 17 0

34623729 T1 1 0

34624204 T1 26 0

34624208 T1 15 0

34624344 T2 12 0

34624625 T2 12 0

34624983 T3 11 1

34625452 T1 37 1

34625698 T1 12 0

34626034 T1 2 0

34626298 T1 21 1

34626370 T1 17 0

34626806 T1 15 0

34626928 T1 10 1

34627453 T1 14 0

34627493 T1 12 0

34628750 T4 12 0

34628986 T1 14 0

34629078 T3 6 1

34629327 T1 23 0

34629457 T1 8 0

34629467 T2 2 0

34630118 T2 25 0

34630123 T1 13 0

34630270 T3 12 0

34630386 T1 19 0

34630435 T1 13 0

34631366 T2 20 0

34631773 T1 11 0

34632258 T1 40 0

34632330 T1 20 0

34632656 T1 13 7

34632837 T2 13 0

34632967 T1 8 0

34633132 T1 13 0

34633137 T3 13 0

34633202 T1 10 0

34633460 T2 20 0

34633749 T1 16 0

34633941 T1 13 0

34634972 T2 12 0

34634974 T1 31 0

34635027 T1 7 0

34635826 T1 28 0

34635887 T1 6 0

34636045 T1 19 0

34636047 T1 2 0

34636188 T3 40 0

34636498 T1 8 0

34636522 T1 23 0

34636571 T1 31 0

34636888 T1 1 0

34636932 T1 23 0

34637107 T1 16 0

34637464 T1 14 0

34637508 T1 23 0

34638012 T1 4 0

34639661 T3 6 0

34640206 T2 40 0

34640300 T3 19 0

34640322 T1 3 0

34640866 T2 27 0

34642106 T3 31 0

34642546 T1 26 0

34642571 T1 28 0

34642595 T1 15 0

34642605 T2 10 0

34643101 T1 11 0

34643303 T1 10 0

34643330 T3 8 0

34643787 T1 35 0

34644418 T2 17 1

34644606 T1 12 0

34644787 T1 5 0

34645055 T1 17 0

34645218 T1 2 0

34645455 T3 2 0

34645654 T1 18 0

34645714 T1 29 0

34645814 T1 22 0

34645898 T1 22 0

34645942 T1 15 0

34647178 T1 14 0

34647213 T1 1 0

34647577 T1 24 0

34647950 T1 13 0

34648177 T2 20 0

34648637 T1 14 0

34648674 T1 9 0

34649383 T1 13 0

34649407 T1 14 0

34650349 T1 3 0

34650540 T1 14 0

34650596 T1 17 0

34651050 T1 21 0

34651331 T1 6 0

34651655 T1 9 0

34651675 T1 30 1

34651972 T1 24 0

34652093 T1 30 0

34652447 T1 8 0

34652612 T3 3 0

34652977 T3 12 0

34653091 T1 13 1

34653105 T1 31 0

34653413 T1 22 0

34653874 T1 15 0

34654163 T1 2 0

34654223 T1 25 0

34654354 T1 13 0

34654756 T1 14 0

34654794 T2 10 0

34654922 T1 11 0

34655125 T4 17 0

34655426 T2 1 0

34655827 T1 18 0

34655858 T2 13 0

34655971 T1 6 2

34656385 T1 6 0

34656442 T3 22 0

34656654 T1 9 0

34656724 T1 43 0

34657135 T3 11 3

34657183 T1 19 0

34657192 T1 16 0

34657280 T1 29 0

34657713 T1 18 0

34657725 T1 21 1

34658264 T1 29 0

34658791 T1 13 0

34659297 T1 27 0

34659313 T1 25 0

34659360 T1 5 0

34659664 T1 18 0

34659772 T1 8 0

34659774 T2 4 0

34659813 T1 22 1

34659988 T1 15 0

34660309 T1 23 0

34660537 T3 38 6

34660794 T1 4 0

34660965 T1 15 0

34661093 T1 5 0

34661262 T2 34 0

34661319 T1 25 0

34661617 T1 2 0

34661681 T1 9 0

34661759 T1 5 0

34662814 T1 3 0

34662824 T2 17 0

34662900 T1 3 0

34663179 T1 15 0

34663234 T1 16 0

34663924 T1 12 0

34664474 T1 12 0

34664499 T1 14 0

34664643 T1 15 0

34664748 T1 31 0

34665015 T1 8 0

34665171 T2 19 0

34665196 T1 20 0

34665274 T1 41 0

34665972 T1 27 0

34666489 T1 27 2

34666985 T2 25 0

34667373 T3 17 0

34667375 T3 11 0

34667382 T1 23 0

34667556 T1 41 0

34667596 T1 8 0

34667850 T1 7 0

34667953 T1 18 0

34668141 T1 11 0

34668473 T1 16 0

34668657 T1 7 0

34668686 T3 24 0

34668771 T2 9 0

34669074 T2 1 0

34669165 T1 16 0

34669265 T1 60 0

34669896 T3 42 3

34669902 T1 25 1

34669923 T2 27 1

34669952 T1 19 0

34670019 T1 12 0

34670123 T3 15 2

34670267 T3 17 0

34670959 T1 18 0

34671017 T1 4 0

34671073 T1 19 0

34671426 T1 20 0

34671491 T1 35 0

34671525 T1 23 0

34671545 T1 11 0

34671684 T1 23 0

34672122 T1 12 0

34672156 T3 6 0

34672160 T1 16 0

34672217 T1 23 0

34672255 T1 19 0

34672373 T2 8 0

34672564 T2 6 1

34672599 T1 12 0

34672638 T1 22 2

34672915 T1 13 0

34672991 T1 2 0

34673183 T1 16 0

34673206 T1 10 0

34673505 T1 11 0

34673511 T1 9 0

34673581 T3 20 2

34673716 T1 10 0

34674003 T1 14 0

34674020 T1 8 0

34674688 T1 11 0

34674707 T1 8 0

34674773 T1 37 0

34674810 T3 26 0

34675430 T1 6 0

34675837 T3 27 3

34675976 T1 19 0

34676104 T1 14 0

34676190 T1 28 0

34676245 T1 15 0

34676295 T1 6 6

34676619 T1 2 0

34677413 T1 12 0

34677510 T1 18 0

34677593 T1 20 0

34677708 T1 6 0

34677709 T2 9 0

34677810 T1 1 0

34678198 T1 15 0

34678512 T2 13 2

34678673 T1 27 0

34678776 T1 18 0

34678926 T2 29 0

34679533 T1 16 2

34679541 T1 3 0

34679741 T1 23 0

34679926 T2 34 0

34680090 T1 17 0

34680138 T1 13 0

34680333 T1 13 0

34680359 T1 19 0

34680437 T3 1 0

34680576 T1 10 0

34680687 T3 34 2

34680693 T1 4 0

34680890 T1 21 0

34681190 T1 7 0

34681384 T1 16 0

34681590 T1 4 0

34681721 T1 3 0

34681743 T1 22 0

34682388 T1 7 0

34682846 T1 14 1

34682867 T1 3 0

34683148 T1 17 0

34683586 T1 4 0

34683647 T3 9 0

34683815 T2 8 0

34684099 T1 7 0

34684196 T1 11 0

34684248 T1 10 0

34684348 T1 1 0

34684540 T2 8 0

34684705 T1 7 0

34684708 T1 28 0

34684930 T1 27 0

34685210 T1 9 0

34685650 T1 13 0

34685768 T1 5 0

34685920 T1 5 0

34686180 T2 9 0

34686335 T2 14 0

34686594 T1 14 0

34686731 T3 20 0

34686852 T2 8 1

34686869 T1 16 0

34686896 T2 41 0

34686915 T1 33 0

34687031 T1 14 0

34687170 T1 10 0

34687514 T1 11 0

34687724 T1 24 0

34688094 T1 5 0

34688347 T1 1 0

34688658 T1 10 0

34688835 T1 3 0

34688895 T1 3 0

34688977 T1 11 0

34689060 T2 33 0

34689432 T1 9 0

34689465 T1 5 0

34689535 T1 4 0

34689886 T1 7 0

34689971 T3 47 4

34690140 T2 5 0

34690470 T1 22 0

34690773 T1 20 0

34691452 T1 21 0

34691591 T1 13 0

34691592 T1 4 0

34691595 T2 19 0

34691906 T1 7 0

34691920 T1 3 2

34691972 T1 29 0

34691987 T1 29 0

34692220 T1 15 0

34692268 T1 32 0

34692833 T1 13 0

34693207 T1 36 0

34693236 T2 54 0

34693583 T1 2 0

34693853 T3 8 0

34694370 T1 10 0

34694510 T1 17 0

34694550 T1 21 0

34694931 T1 8 0

34695301 T1 4 0

34695393 T2 8 0

34695500 T2 18 0

34695577 T3 11 0

34695824 T1 3 0

34695887 T1 16 0

34695930 T1 8 0

34696052 T1 1 0

34696408 T1 13 0

34696483 T3 6 1

34696542 T2 9 0

34696579 T1 16 0

34697046 T1 18 0

34697979 T1 24 0

34697996 T1 17 0

34698103 T1 14 0

34698775 T1 33 0

34698880 T1 19 0

34698951 T1 36 0

34699783 T1 21 0

34700421 T1 5 0

34700875 T1 13 0

34703606 T1 7 0

34705373 T1 2 0

34705598 T2 4 0

34707393 T1 20 0

34708381 T1 9 0

38001654 T1 5 0

38003334 T3 22 0

38052292 T1 69 0

38103706 T1 12 0

38103916 T1 19 0

38104464 T1 3 0

38107812 T1 3 0

38108051 T1 5 0

38109637 T1 20 0

38109990 T1 7 0

38110007 T1 2 0

38112176 T1 25 0

38115984 T1 2 0

38118599 T3 3 0

38118703 T1 2 0

38118715 T1 3 0

38119009 T1 9 0

38120263 T1 1 0

38120286 T1 3 0

38120969 T2 3 0

38121224 T1 6 0

38121537 T1 10 0

38121697 T1 13 0

38121815 T1 4 0

38123024 T1 1 0

38123423 T1 5 0

38125057 T1 9 0

38126656 T1 24 0

38126699 T1 5 0

38127090 T1 9 0

38127126 T1 24 0

38127251 T1 5 0

38127817 T1 1 0

38128184 T1 14 0

38129596 T1 9 0

38129768 T2 15 0

38130010 T1 9 0

38130439 T3 17 4

38131567 T1 17 0

38133491 T1 50 0

38133578 T1 45 0

38134025 T1 3 0

38135200 T1 3 0

38135787 T1 26 0

38135907 T2 16 1

38136167 T1 6 0

38136750 T1 11 0

38136841 T1 2 0

38136934 T1 7 0

38137455 T1 3 0

38137478 T1 16 0

38137848 T1 4 0

38138003 T1 29 0

38138371 T2 12 0

38139038 T1 12 0

38139191 T1 19 0

38139503 T1 1 0

38139742 T1 3 0

38140843 T1 10 0

38140845 T1 9 0

38140848 T1 3 0

38141112 T1 17 0

38141364 T1 2 0

38141429 T1 45 0

38141570 T3 3 0

38141745 T1 11 3

38141881 T1 14 0

38142104 T1 1 0

38142841 T1 10 0

38143137 T1 34 0

38143840 T1 1 0

38144687 T1 1 0

38145264 T1 4 0

38145528 T1 1 0

38145617 T1 2 0

38146360 T1 29 0

38146378 T1 1 0

38146394 T1 1 0

38146401 T1 4 0

38146427 T1 35 0

38146471 T1 22 0

38146497 T1 3 0

38146796 T1 6 0

38147183 T1 67 0

38147197 T1 38 0

38147606 T1 32 0

38147949 T1 18 0

38148871 T1 12 0

38149011 T1 5 0

38149648 T1 10 0

38150010 T1 8 0

38150492 T2 42 0

38150507 T2 36 0

38150624 T1 1 0

38150652 T1 11 0

38151106 T1 8 1

38151113 T1 19 0

38151320 T1 16 0

38151515 T1 4 0

38151648 T1 23 0

38153816 T1 55 0

38153857 T1 26 0

38153879 T1 38 0

38154174 T1 24 0

38154316 T1 26 0

38154930 T2 52 0

38155910 T1 10 0

38156270 T1 27 1

38156464 T1 2 0

38156504 T1 12 2

38156618 T1 13 0

38156698 T1 39 0

38157248 T1 15 0

38157419 T1 34 0

38158031 T1 3 0

38158205 T1 29 0

38159423 T1 9 0

38159855 T1 28 0

38159921 T1 2 0

38160558 T1 21 0

38161130 T1 17 0

38161292 T1 29 0

38161432 T1 7 0

38161496 T1 10 0

38163257 T1 13 0

38163464 T1 14 0

38163548 T1 15 0

38163639 T1 9 0

38163694 T1 18 0

38163705 T2 49 0

38164249 T1 9 0

38164729 T3 1 0

38165026 T1 2 0

38166279 T1 17 0

38166542 T1 66 0

38167174 T1 14 0

38167251 T1 26 0

38167336 T1 8 0

38167387 T1 23 0

38167446 T1 20 0

38167498 T1 38 0

38168057 T3 60 0

38168304 T1 1 0

38168583 T1 2 0

38168768 T2 7 0

38168984 T1 24 0

38168987 T1 17 0

38170169 T1 9 0

38170941 T1 5 0

38170958 T2 8 0

38171454 T1 10 0

38171889 T1 4 0

38172127 T1 5 0

38172320 T2 52 0

38172351 T1 1 0

38172948 T1 15 0

38173083 T1 8 0

38173713 T1 21 0

38173891 T1 21 0

38173923 T1 25 0

38173936 T2 21 0

38174080 T1 28 0

38174089 T1 26 0

38174382 T1 21 0

38174573 T1 30 0

38174884 T1 16 0

38175303 T2 9 0

38175410 T1 40 0

38175453 T1 4 0

38175561 T1 26 0

38175766 T1 16 0

38175851 T1 6 0

38176302 T3 11 0

38176348 T2 9 0

38176527 T1 9 0

38176763 T1 21 0

38177110 T1 26 0

38177112 T1 4 0

38177385 T2 57 0

38177560 T1 40 0

38177694 T2 7 0

38177787 T1 9 0

38177834 T1 3 0

38178036 T2 4 0

38178088 T1 23 0

38178093 T1 8 0

38178098 T3 29 0

38178185 T1 23 0

38178220 T1 16 0

38178493 T1 31 0

38178935 T1 22 0

38178939 T1 34 0

38178962 T1 4 0

38180459 T1 22 0

38180624 T1 15 0

38180831 T1 13 0

38180952 T1 27 0

38180981 T1 10 0

38181363 T2 14 1

38181789 T1 9 0

38181936 T1 15 0

38181961 T1 27 0

38182394 T1 32 0

38182674 T1 33 0

38182873 T1 32 1

38182907 T1 11 0

38183462 T1 15 0

38183736 T1 29 0

38183791 T1 15 0

38184055 T1 14 0

38184325 T2 33 0

38184380 T1 3 0

38184413 T1 33 0

38184419 T1 29 0

38184844 T1 30 0

38184850 T1 18 0

38185174 T1 6 0

38185262 T1 26 0

38185760 T1 17 0

38186137 T1 7 0

38186161 T1 22 0

38186232 T1 27 0

38186264 T1 3 0

38186455 T1 20 0

38186847 T1 24 0

38186943 T2 5 0

38187101 T1 23 0

38187165 T1 8 0

38187215 T1 18 0

38187285 T2 20 0

38187586 T1 37 0

38187953 T1 5 0

38188692 T1 7 0

38188776 T1 69 0

38189655 T1 14 0

38190068 T1 7 0

38190462 T1 19 0

38190496 T1 5 0

38191015 T1 21 0

38191172 T1 5 0

38191559 T2 6 0

38192549 T2 19 0

38192780 T1 3 0

38193313 T1 18 0

38193330 T1 2 0

38193545 T2 22 0

38193739 T1 9 0

38194736 T1 16 0

38194816 T1 13 0

38194892 T1 6 0

38195287 T1 8 0

38195368 T1 6 0

38195761 T1 4 0

38196282 T1 8 0

38196586 T1 16 0

38196620 T1 2 0

38196994 T1 11 0

38197203 T1 14 0

38197415 T1 1 0

38197503 T1 16 2

38197577 T1 26 0

38197685 T1 3 0

38197686 T1 3 1

38197716 T1 13 0

38198564 T1 5 0

38198769 T1 24 0

38198840 T2 1 1

38199004 T1 1 0

38199699 T1 4 0

38199720 T1 11 0

38199741 T1 10 0

38200176 T3 9 0

38200491 T1 21 0

38200940 T1 10 0

38201203 T3 7 1

38201233 T1 25 0

38201424 T1 18 0

38201769 T1 4 0

38202314 T1 9 0

38202999 T1 4 0

38203001 T1 29 0

38203037 T1 25 0

38203112 T2 12 0

38203660 T1 7 0

38203731 T1 18 0

38203921 T1 18 0

38204067 T1 22 0

38204357 T1 16 0

38204406 T1 22 0

38204501 T1 18 0

38204505 T2 12 0

38204623 T1 7 0

38205164 T1 6 0

38205375 T1 27 0

38205458 T1 3 0

38205492 T1 18 0

38205549 T1 16 0

38205563 T3 26 0

38205600 T4 5 0

38205850 T1 10 0

38205885 T2 1 0

38206337 T1 6 0

38206371 T1 28 0

38206531 T3 25 0

38206549 T1 9 0

38206866 T1 22 0

38206875 T1 8 0

38207094 T1 24 0

38207330 T1 30 0

38207535 T1 6 0

38208270 T1 16 1

38208365 T1 19 0

38208367 T1 5 0

38208419 T1 6 0

38208498 T1 12 0

38208542 T3 9 0

38208606 T1 24 0

38208700 T1 4 0

38208759 T1 38 0

38209252 T1 20 0

38209508 T1 12 0

38209629 T3 20 0

38209688 T1 2 0

38209750 T1 16 0

38209840 T3 4 0

38210058 T2 22 0

38210417 T1 11 0

38210939 T1 12 0

38211427 T1 25 0

38211525 T1 15 0

38211921 T1 8 0

38213008 T1 15 0

38213021 T1 8 0

38213076 T2 24 2

38213373 T1 6 0

38213384 T1 12 0

38213421 T2 5 0

38213664 T1 16 0

38213717 T1 20 0

38213781 T1 12 0

38213789 T1 5 0

38213871 T1 33 0

38214229 T2 52 0

38214651 T1 17 0

38214773 T1 53 0

38215042 T1 22 0

38215353 T1 19 0

38215745 T1 21 0

38215928 T1 8 0

38216149 T1 22 0

38216326 T1 22 0

38216401 T2 26 0

38216530 T1 38 0

38216585 T1 20 0

38216976 T1 17 0

38217004 T1 7 0

38217084 T1 29 0

38218019 T1 23 0

38218214 T1 11 0

38218606 T1 8 0

38218951 T1 6 0

38219358 T2 8 0

38219866 T1 12 0

38219955 T1 14 0

38220061 T1 10 0

38220369 T2 16 0

38220991 T1 7 0

38221407 T1 20 0

38221465 T1 24 0

38221498 T1 26 0

38221655 T1 11 0

38222088 T1 7 0

38222360 T2 25 0

38222508 T1 14 0

38223578 T1 10 0

38223698 T1 7 0

38223816 T1 18 0

38223872 T1 31 0

38223902 T1 14 0

38224132 T1 23 0

38224169 T1 28 1

38224549 T1 12 0

38224988 T1 22 0

38225085 T1 11 0

38225508 T1 15 0

38225695 T1 43 0

38225702 T1 18 0

38225896 T1 10 0

38226128 T3 15 0

38226285 T1 33 0

38226544 T2 23 0

38226719 T3 13 0

38226847 T3 11 0

38226877 T1 2 1

38227149 T1 28 0

38227960 T1 7 0

38228048 T2 14 1

38228121 T3 6 0

38229276 T1 6 0

39002764 T1 10 0

39032951 T4 4 0

39032981 T1 13 0

39033194 T1 1 0

39033562 T1 6 0

39034360 T1 2 0

39034797 T1 9 0

39035583 T1 10 0

39037164 T1 10 0

39037749 T1 15 0

39038064 T1 5 1

39038921 T1 1 0

39039218 T1 24 0

39039748 T1 6 0

39040245 T1 10 0

39040856 T3 1 0

39042288 T1 11 0

39042770 T1 6 0

39043385 T1 3 0

39044250 T1 1 0

39044257 T2 21 0

39045316 T1 13 0

39045649 T2 13 0

39046644 T1 3 0

39046651 T1 1 0

39047019 T1 2 0

39047226 T1 5 2

39047505 T1 6 0

39047646 T1 1 0

39049621 T1 21 0

39049966 T1 11 0

39050322 T1 1 0

39050447 T1 20 0

39051312 T2 1 1

39053855 T2 2 0

39054978 T1 2 0

39055071 T1 12 0

39055216 T1 10 0

39055601 T1 1 0

39056774 T1 7 0

39057857 T1 2 0

39057866 T1 4 0

39058265 T1 2 0

39059353 T1 31 0

39059561 T1 6 0

39060486 T1 7 0

39061617 T1 2 0

39062117 T3 16 0

39062687 T1 3 0

39063148 T1 10 0

39064210 T1 5 0

39064573 T1 6 0

39064855 T1 9 0

39064867 T1 10 0

39065309 T1 6 0

39065327 T1 3 0

39065750 T1 4 0

39069231 T1 5 0

39069846 T1 2 0

39069922 T1 5 0

39070489 T2 12 0

39071597 T1 4 0

39071605 T1 12 0

39073810 T1 3 0

39074560 T1 5 0

39075168 T1 18 0

39076389 T1 12 1

39076935 T1 1 0

39076938 T1 7 0

39077687 T1 2 0

39078010 T1 3 0

39078793 T1 5 0

39079134 T1 2 0

39079749 T1 1 0

39083757 T1 2 0

39083839 T2 34 0

39084779 T1 21 0

39085034 T1 2 0

39085369 T1 25 0

39085491 T1 10 0

39085834 T1 32 0

39087312 T1 18 0

39088741 T1 15 0

39089033 T1 17 0

39089057 T1 18 0

39089073 T1 7 0

39091544 T2 3 0

40135014 T1 15 0

40135690 T1 2 0

40141131 T1 7 0

40141446 T1 4 2

40141504 T1 1 0

40142235 T1 3 0

40145435 T1 15 0

40147107 T1 1 0

40148243 T1 7 1

40150072 T1 11 0

40152047 T1 11 0

40153198 T1 1 0

40154454 T1 1 0

40155213 T1 1 0

40155601 T1 1 0

40155681 T1 5 0

40156801 T1 2 1

40156824 T1 21 1

40157679 T1 14 0

40160941 T1 8 0

40161962 T1 2 0

40162933 T1 3 0

40163319 T1 6 0

40163748 T1 8 0

40163943 T1 12 0

40165534 T1 1 0

40165692 T1 18 0

40166259 T1 11 0

40168372 T1 15 0

40169584 T2 8 0

40169650 T1 1 0

40169833 T3 46 4

40169852 T1 10 0

40170140 T1 3 0

40170694 T1 2 0

40170755 T3 1 0

40171457 T1 44 0

40172000 T1 13 0

40172193 T1 4 0

40172520 T1 1 0

40172984 T1 18 0

40173012 T1 7 0

40173890 T2 10 0

40174408 T1 5 0

40174436 T1 2 0

40174813 T1 12 0

40176543 T1 2 0

40179747 T1 11 0

40182420 T1 1 0

40183137 T2 31 0

40183152 T1 18 0

40183424 T1 2 1

40183863 T1 35 0

40185741 T3 6 1

40186278 T1 5 0

40187081 T2 2 0

40187467 T1 11 0

40187886 T1 5 0

40188066 T1 5 0

40188839 T1 9 0

40189905 T1 3 0

40189990 T1 19 0

40190494 T1 5 0

40190638 T1 5 0

40191156 T2 2 0

40192467 T2 23 0

40192511 T1 2 0

40193122 T1 7 0

40193430 T1 5 0

40193998 T2 25 0

40194674 T1 5 0

40194954 T3 11 0

40195118 T2 1 0

40195868 T1 7 0

40195894 T1 16 0

40196864 T1 21 0

40197411 T1 8 0

40197743 T1 11 0

40198848 T1 1 0

40199446 T1 7 0

40199524 T2 3 0

40200137 T1 41 0

40200218 T1 6 0

40200291 T1 3 0

40200370 T1 8 0

40201177 T1 21 0

40201194 T1 8 0

40202164 T1 19 0

40202470 T1 10 0

40202558 T1 15 0

40202841 T1 8 0

40203142 T1 4 0

40203765 T1 2 0

40205084 T1 1 0

40207077 T1 8 0

40207277 T1 2 0

40207562 T1 27 0

40208210 T1 2 0

40209122 T1 4 0

40211751 T1 6 0

40211819 T2 2 0

40212288 T2 13 1

40212390 T1 10 0

40214761 T3 5 0

40214947 T1 24 5

40215178 T1 2 0

40215192 T1 13 0

40216377 T1 21 0

40216720 T1 4 0

40217552 T1 11 0

40217896 T1 1 0

40218819 T1 1 0

40218922 T1 13 0

40219692 T1 1 0

40219737 T2 15 0

40219937 T1 8 1

40220536 T1 2 0

40220886 T1 3 0

40221350 T1 11 0

40221973 T1 15 0

40222364 T1 5 0

40222643 T1 45 0

40223244 T1 35 0

40223412 T1 17 0

40223947 T1 11 0

40224052 T1 10 0

40224054 T2 1 0

40224565 T1 6 0

40224799 T1 16 0

40225064 T1 31 0

40833933 T1 7 0

40834333 T1 25 0

40834348 T1 23 0

40834514 T1 8 0

40834923 T1 9 0

40836256 T1 6 0

40836373 T1 2 0

40838612 T1 7 0

40839981 T1 12 0

40840392 T1 9 0

40840891 T3 18 0

40842031 T1 27 0

40842438 T1 23 0

40842702 T1 7 0

40843045 T1 14 0

40844268 T1 16 0

40844271 T1 5 0

40845380 T1 2 0

40845507 T1 13 0

40846279 T3 8 0

40846434 T2 13 0

40846690 T1 10 0

40847193 T1 2 0

40847318 T2 9 7

40847379 T1 8 0

40848423 T1 1 0

40848945 T1 30 0

40848967 T1 21 0

40849303 T2 4 0

40849522 T1 4 0

40850232 T1 5 0

40850440 T1 8 0

40850650 T2 5 2

40851863 T1 2 0

40851941 T1 18 0

40852008 T1 4 0

40852290 T3 10 0

40852343 T3 27 0

40852967 T1 18 0

40853603 T1 18 0

40853981 T1 27 0

40854899 T1 6 0

40855185 T1 1 0

40855217 T1 9 0

40855309 T1 2 0

40855370 T1 4 0

40855756 T1 4 0

40855862 T1 12 0

40856063 T2 9 0

40856178 T1 9 0

40856365 T1 11 0

40856399 T1 4 0

40856559 T1 12 0

40856995 T1 3 0

40857830 T2 24 0

40857857 T1 18 0

40858049 T1 16 0

40858615 T1 15 0

40858786 T2 9 0

40858819 T1 2 0

40858872 T1 9 0

40859400 T3 21 0

40859885 T2 20 1

40859892 T1 3 0

40859930 T1 7 0

40860395 T1 29 0

40860419 T1 13 0

40860533 T1 17 0

40860664 T1 22 0

40860963 T1 14 0

40860985 T1 14 0

40861778 T1 7 0

40861822 T1 11 0

40861925 T1 2 0

40861937 T3 15 0

40862657 T1 10 0

40863358 T1 6 0

40864051 T1 8 0

40864102 T1 3 0

40864426 T1 11 0

40864659 T3 6 0

40864891 T2 33 0

40864971 T1 6 0

40865748 T1 8 0

40865774 T1 10 0

40865816 T2 18 0

40865845 T1 11 0

40866143 T1 3 0

40867580 T1 10 0

40868170 T1 23 0

40870013 T2 24 0

40870186 T1 8 0

40871091 T3 8 0

40871393 T1 11 0

40871490 T1 8 0

40871757 T1 8 0

40872871 T1 19 0

40873129 T1 10 5

40873366 T1 26 0

40874055 T1 14 0

40874069 T1 5 0

40874835 T2 11 0

40874841 T2 13 0

40875787 T1 2 0

40876419 T1 19 0

40877201 T1 1 0

40877228 T2 19 0

40877759 T1 16 0

40877876 T1 6 0

40877890 T1 17 0

40878033 T1 10 0

40878155 T1 6 0

40878257 T1 6 0

40878276 T1 17 0

40878465 T3 5 0

40878836 T3 21 0

40879111 T1 21 0

40879181 T1 6 0

40879196 T1 27 0

40879225 T2 3 0

40879255 T1 32 0

40879256 T2 11 0

40879923 T1 20 0

40880622 T2 33 0

40880704 T1 9 0

40880806 T1 10 0

40880848 T1 19 0

40881014 T1 11 0

40881204 T1 9 0

40881241 T1 6 0

40881342 T2 9 0

40881479 T1 5 0

40881886 T1 19 0

40881889 T1 17 0

40881935 T1 26 0

40881989 T1 11 0

40882382 T1 5 0

40882392 T1 8 0

40882548 T1 4 0

40883513 T1 4 0

40883560 T1 7 0

40884220 T1 23 0

40884321 T1 8 0

40884428 T2 8 0

40885449 T1 19 0

40885654 T1 11 0

40885658 T1 9 0

40886129 T1 9 0

40886156 T1 10 0

40886274 T1 37 0

40886747 T1 17 0

40886990 T1 9 0

40887901 T1 9 0

40888012 T1 4 0

40888014 T2 10 0

40888348 T2 14 0

40888351 T1 13 0

40888529 T1 5 0

40888533 T1 6 0

40888853 T4 17 1

40889297 T2 17 2

40889438 T1 11 0

40889691 T1 20 0

40889819 T3 25 1

40889941 T1 21 0

40890282 T1 6 0

40890312 T1 12 0

40890458 T1 3 0

40890532 T1 9 0

40890577 T1 5 0

40891010 T1 14 0

40891258 T1 7 0

40891316 T1 17 0

40891402 T1 6 0

40891453 T1 11 0

40891606 T1 10 0

40891743 T1 7 0

40891773 T1 12 0

40891774 T1 31 0

40891972 T1 7 0

40892187 T1 5 0

40892551 T1 12 0

40892627 T1 32 0

40892893 T1 13 0

40893096 T1 4 0

40893187 T1 16 0

40893293 T1 24 0

40893766 T1 12 0

40893885 T1 11 0

40894632 T1 32 0

40894648 T2 7 0

40894818 T1 10 0

40894883 T1 6 0

40895387 T1 11 0

40895678 T2 8 2

40895786 T1 8 0

40896519 T1 6 0

40896823 T2 10 0

40896835 T1 9 0

40897057 T1 2 0

40897370 T2 1 0

40897441 T1 19 0

40897529 T1 7 0

40897685 T1 6 0

40897757 T1 6 0

40897786 T1 12 0

40897840 T1 11 0

40897967 T2 20 0

40898273 T1 11 1

40898353 T3 7 0

40899058 T3 1 0

40899218 T1 11 0

40901016 T1 1 0

40901222 T1 28 0

40901275 T1 12 0

40901311 T2 15 0

40901389 T1 11 0

40902098 T1 10 0

40902179 T2 11 0

40902397 T1 21 0

40902453 T2 17 0

40902591 T1 24 0

40902726 T1 10 0

40903047 T1 6 0

40903079 T1 13 0

40903187 T1 3 0

40903323 T1 8 0

40903424 T1 7 0

40903588 T1 1 0

40903630 T2 7 0

40903669 T1 14 0

40904585 T3 4 0

40904652 T1 12 0

40905106 T1 35 0

40905257 T1 6 0

40905345 T1 2 0

40905358 T1 16 0

40905444 T1 9 0

40905478 T1 4 0

40905553 T1 13 0

40905617 T1 4 0

40905654 T1 13 0

40906044 T1 6 0

40906994 T2 17 0

40908275 T2 14 0

40908740 T1 2 0

40908782 T2 11 0

40908829 T1 15 0

40908909 T2 27 0

40908952 T2 5 1

40908981 T1 6 0

40909112 T1 12 0

40909336 T1 8 0

40909737 T1 19 0

40909744 T1 8 0

40909961 T2 32 0

40910317 T1 9 0

40911126 T1 7 0

40911128 T1 12 0

40912506 T3 10 1

40913582 T1 17 0

40913630 T1 10 0

40913640 T1 15 0

40913696 T1 12 0

40913701 T1 6 0

40913827 T1 5 0

40913915 T1 16 0

40914022 T1 41 0

40914080 T1 14 0

40914171 T1 17 0

40914248 T1 18 0

40914281 T1 5 0

40914308 T1 9 0

40914462 T1 4 0

40914601 T2 6 0

40914679 T1 18 0

40914860 T1 9 0

40915701 T1 3 0

40916059 T1 28 0

40916213 T1 11 0

40916615 T1 11 0

40917043 T1 6 0

40917078 T1 8 0

40917161 T1 3 0

40918035 T2 1 0

40918054 T1 29 0

40918218 T1 7 0

40918521 T1 1 0

40918690 T1 1 0

40918692 T2 12 0

40918743 T1 11 0

40919344 T1 20 0

40919487 T1 7 0

40919488 T1 22 0

40919520 T1 5 0

40920246 T1 5 0

40920333 T2 9 0

40921444 T1 11 0

40921448 T1 10 0

40922095 T1 11 0

40922276 T2 10 0

40922292 T1 4 0

40922557 T1 9 0

40922702 T1 17 0

40922801 T2 13 0

40922817 T2 9 0

40922881 T1 7 0

40922939 T2 5 0

40923602 T1 13 0

40923622 T1 16 0

40923696 T1 9 0

40924141 T1 15 0

40924314 T1 6 0

40924418 T1 23 0

40924569 T1 2 0

40924692 T3 6 0

40924913 T1 7 1

40924926 T1 5 0

40925502 T4 10 5

40925575 T1 30 0

40925786 T1 15 0

40925812 T1 15 0

40925827 T1 15 0

40926051 T1 8 0

40926096 T1 3 0

40926189 T1 2 0

40926388 T1 3 0

40926768 T1 24 0

40926797 T1 8 0

40926806 T3 7 0

40927302 T1 17 0

40928598 T1 11 0

40929034 T1 19 0

40929107 T3 14 0

40929154 T1 8 0

40929395 T1 7 0

40929453 T1 17 0

40929522 T3 19 0

40929698 T1 12 0

40929834 T1 14 0

40929872 T2 11 0

40930078 T1 10 0

40931426 T2 9 0

40932535 T1 14 0

40932691 T1 4 0

40932895 T1 39 0

40933374 T1 9 0

40934060 T1 17 0

40934387 T1 10 0

40934603 T1 10 0

40934678 T1 12 0

40935040 T1 19 0

40935225 T1 7 0

40935649 T1 7 0

40935745 T1 24 0

40936019 T1 7 0

40936574 T1 12 0

40936883 T1 4 0

40936979 T1 8 0

40937226 T1 11 0

40937232 T1 12 3

40937635 T1 16 0

40937673 T2 12 6

40937701 T1 14 0

40937816 T1 7 0

40937836 T1 5 0

40937858 T1 17 0

40937918 T1 15 0

40938202 T1 10 0

40938353 T1 11 0

40938940 T1 2 0

40939019 T1 1 0

40939140 T1 21 0

40939322 T2 17 0

40939498 T2 9 0

40940110 T1 12 0

40940160 T1 7 0

40940206 T1 7 1

40940662 T3 9 0

40940819 T1 10 0

40940909 T1 7 0

40940929 T1 13 0

40940962 T1 24 0

40940969 T2 6 0

40941005 T1 10 0

40941031 T1 10 0

40941064 T1 15 1

40941099 T1 12 0

40941138 T1 5 0

40941669 T1 16 0

40941850 T2 5 0

40942559 T1 13 0

40942831 T1 13 0

40943048 T1 16 0

40943060 T1 23 0

40943523 T1 15 0

40943782 T1 15 0

40944446 T1 5 0

40944610 T1 21 0

40944634 T1 28 0

40944710 T1 10 0

40944838 T1 11 0

40945063 T1 17 0

40945279 T2 9 0

40945759 T1 4 0

40945898 T1 8 0

40945935 T1 9 0

40946145 T1 1 0

40946408 T1 11 0

40946561 T1 6 0

40946948 T1 6 0

40946960 T1 13 0

40947035 T1 12 0

40947369 T1 3 0

40947474 T1 2 0

40947574 T1 3 0

40947813 T1 12 0

40948345 T2 3 0

40948533 T1 11 0

40948581 T1 9 0

40948645 T1 19 0

40949337 T1 11 0

40949520 T2 4 0

40949582 T1 14 0

40949796 T2 1 0

40949827 T1 29 0

40950360 T1 14 0

40950381 T1 8 0

40951165 T1 9 0

40951483 T2 25 1

40951486 T1 13 0

40951818 T4 1 0

40951877 T1 4 0

40952100 T2 14 0

40952596 T2 30 0

40952704 T2 1 0

40952962 T2 21 0

40952974 T1 5 0

40953029 T1 13 0

40954177 T1 4 0

40954566 T1 4 0

40954576 T2 5 1

40954756 T1 6 0

40954899 T1 10 0

40955335 T2 9 0

40955451 T1 13 0

40955523 T2 15 0

40955531 T3 15 0

40955632 T2 8 0

40955759 T1 8 0

40955805 T3 2 0

40955851 T1 8 4

40955870 T2 5 0

40955961 T1 11 0

40955986 T1 16 0

40956020 T1 9 0

40956202 T1 2 0

40956354 T2 16 0

40956542 T1 3 0

40956560 T1 6 0

40956990 T1 12 2

40957040 T1 14 0

40957246 T1 6 0

40957296 T1 4 0

40957328 T2 7 0

40958236 T1 1 0

40958621 T1 3 0

40959288 T1 1 0

40959768 T1 2 0

40959777 T1 19 0

40959836 T1 10 1

40959917 T1 5 0

40960257 T1 12 0

40960840 T2 15 0

40961450 T1 5 0

40961552 T2 9 1

40962426 T1 5 0

40962867 T1 5 0

40962947 T1 12 0

40963783 T1 25 0

40963962 T1 10 0

40964172 T1 2 0

40964178 T1 20 0

40964315 T1 8 0

40964845 T1 16 0

40964929 T1 15 0

40964934 T1 3 0

40965177 T1 16 2

40965226 T3 13 0

40965249 T1 19 0

40965340 T2 16 3

40965666 T2 4 1

40965911 T1 17 0

40966323 T1 14 0

40966370 T1 8 0

40966408 T1 12 0

40966776 T1 5 0

40967077 T1 9 0

40967454 T3 3 0

40967791 T1 13 0

40967793 T1 13 0

40968111 T1 18 0

40968118 T1 10 0

40968172 T1 2 0

40968203 T1 4 0

40968234 T1 10 0

40968239 T1 13 0

40968249 T3 19 1

40968667 T1 17 0

40969042 T1 14 0

40969329 T1 9 0

40969334 T1 2 0

40969337 T1 15 1

40969836 T1 3 0

40970114 T1 20 0

40970123 T1 4 0

40970218 T1 9 0

40970361 T1 24 0

40970715 T1 10 0

40970876 T1 2 0

40970946 T1 3 0

40972071 T1 7 0

40972137 T1 14 0

40972524 T1 8 0

40973310 T1 4 0

40973335 T2 12 0

40973365 T1 18 0

40973386 T1 5 0

40973466 T1 10 0

40973515 T1 5 0

40973543 T1 11 0

40973736 T1 13 0

40974191 T1 2 0

40974365 T1 7 1

40974419 T1 3 0

40974937 T1 6 2

40975187 T2 3 0

40975278 T1 4 0

40975849 T1 2 0

40975992 T1 1 0

40976480 T1 23 0

40977616 T1 9 0

40977621 T3 1 1

40977695 T1 5 0

40977699 T1 13 0

40978361 T1 19 0

40978428 T1 9 0

40978719 T1 24 0

40978888 T1 16 0

40979390 T1 13 0

40979487 T2 12 0

40979560 T1 13 0

40979657 T1 13 0

40979686 T1 5 0

40980444 T1 5 0

40980452 T1 3 0

40980675 T1 4 0

40980690 T1 5 0

40980887 T1 24 0

40982378 T2 11 0

40982542 T1 1 1

40982586 T1 14 0

40982685 T1 11 0

40982926 T1 10 0

40983229 T1 1 0

40983326 T1 15 0

40983612 T1 12 0

40983650 T1 11 0

40983915 T1 7 0

40984414 T2 5 0

40984416 T1 14 0

40984417 T2 4 0

40984472 T1 8 0

40984588 T1 14 0

40985453 T2 6 0

40985597 T1 7 0

40985678 T1 12 0

40985754 T1 2 0

40985978 T1 1 0

40986119 T1 17 0

40986144 T1 11 0

40986162 T1 13 0

40986185 T1 11 0

40986261 T1 10 0

40986265 T1 6 0

40986296 T2 3 0

40986354 T1 7 0

40987292 T2 29 0

40987376 T3 25 0

40987507 T1 6 0

40987678 T1 8 0

40987895 T1 13 0

40988331 T1 6 0

40988341 T1 7 0

40988370 T1 1 0

40989013 T1 9 0

40989014 T1 17 0

40989025 T1 9 0

40989652 T1 5 0

40989867 T2 14 0

40989870 T1 16 0

40989936 T1 24 0

40990066 T1 2 0

40991352 T1 9 0

40991528 T1 22 0

40991665 T1 15 0

40991690 T1 6 0

40992182 T1 7 0

40992941 T1 3 0

40992994 T2 4 0

40993120 T1 6 0

40993232 T2 17 0

40993260 T1 4 0

40993262 T1 2 0

40993346 T2 5 0

40993359 T1 4 0

40993377 T1 15 0

40993385 T2 19 0

40993414 T1 11 0

40993419 T1 10 0

40993576 T1 8 0

40993829 T2 12 0

40993867 T1 1 0

40993950 T1 5 0

40994308 T2 5 0

40994463 T1 12 0

40994559 T1 6 0

40995179 T2 12 0

40995787 T2 2 0

40995808 T1 5 0

40996141 T3 8 4

40996805 T1 10 0

40996865 T1 4 0

40997615 T1 2 0

40997741 T1 14 0

40998095 T1 10 0

40998600 T1 19 1

40998743 T1 11 0

40998874 T1 7 0

40999747 T1 11 0

40999969 T1 12 0

41000012 T3 13 0

41000120 T1 8 0

41000208 T2 18 0

41000584 T1 4 0

41000672 T1 10 0

41000770 T1 8 0

41000821 T1 15 0

41001018 T1 7 0

41001252 T1 11 0

41002224 T1 14 1

41002543 T1 7 0

41002803 T1 14 0

41003326 T1 10 0

41003332 T1 5 0

41003333 T1 7 0

41003360 T1 5 0

41004489 T2 22 0

41004597 T1 16 0

41004724 T1 3 0

41004826 T1 4 0

41004879 T1 5 0

41004928 T1 16 0

41004991 T1 7 0

41005070 T1 2 1

41005190 T1 4 0

41005192 T1 13 0

41005196 T1 5 0

41005209 T1 6 0

41005219 T1 6 0

41005618 T1 5 0

41006172 T1 12 0

41006298 T1 3 0

41007144 T3 11 0

41007211 T1 3 0

41007215 T2 7 0

41007400 T1 2 0

41007479 T1 16 0

41007509 T2 5 0

41007574 T1 9 0

41007616 T3 1 0

41007709 T1 3 0

41007710 T1 4 0

41007769 T1 4 0

41007776 T1 7 0

41008149 T1 12 0

41008665 T3 6 0

41008807 T3 12 1

41009114 T1 9 0

41009462 T1 7 0

41009721 T1 9 0

41010476 T1 5 0

41010684 T1 12 0

41011342 T1 10 0

41011384 T1 8 0

41011738 T1 18 0

41012579 T1 5 0

41012998 T2 3 0

41013041 T1 4 0

41013465 T1 12 0

41013786 T1 5 0

41013940 T1 4 0

41014153 T1 17 0

41014204 T1 18 0

41014267 T1 8 0

41014268 T1 10 0

41014270 T1 12 5

41014466 T1 10 0

41014705 T2 5 0

41014845 T1 25 0

41014893 T1 22 0

41015041 T1 22 0

41015182 T1 2 0

41015244 T1 6 0

41015763 T1 7 0

41015784 T1 13 0

41015856 T1 9 1

41015944 T1 8 0

41016697 T1 7 0

41016720 T1 3 0

41017257 T1 16 0

41017512 T1 23 0

41017599 T1 7 0

41017621 T1 17 0

41017652 T1 3 0

41018289 T1 10 0

41018292 T3 1 0

41018374 T1 10 0

41018450 T2 11 0

41018579 T1 17 0

41018596 T1 1 0

41018601 T3 4 0

41018818 T1 13 0

41019041 T2 5 0

41019503 T1 17 0

41019691 T1 17 0

41020213 T1 7 0

41020688 T1 11 0

41020778 T1 3 0

41021275 T1 2 0

41021722 T1 24 0

41022243 T1 15 0

41022312 T1 5 0

41022404 T1 5 0

41022432 T1 1 0

41022447 T1 5 0

41022475 T1 14 0

41022566 T1 7 0

41023507 T3 2 0

41023525 T1 6 0

41024205 T1 10 0

41025156 T1 7 0

41025399 T1 9 0

41026090 T1 3 0

41027454 T1 2 0

41027576 T1 11 0

41027615 T1 6 0

41027654 T1 8 0

41027750 T1 4 0

41029643 T1 7 0

41030091 T2 6 0

41030318 T2 1 0

41030485 T1 3 0

41033090 T1 14 0

41037143 T1 13 0

41039867 T1 5 0

41040284 T1 19 0

43000435 T1 26 0

43000835 T1 6 0

43036364 T2 6 0

43037443 T2 8 0

43041167 T1 8 0

43043275 T1 47 0

43045399 T1 15 0

43051415 T1 29 0

43055895 T2 24 1

43057736 T1 13 0

43058142 T1 18 0

43060460 T4 8 0

43066158 T1 3 0

43066496 T1 28 0

43066563 T1 2 0

43066588 T1 15 0

43066794 T1 9 0

43067256 T2 17 0

43067415 T2 20 0

43068030 T1 17 0

43068662 T1 21 0

43069465 T1 11 0

43069646 T1 30 0

43069687 T1 11 0

43069939 T1 37 0

43070015 T1 15 0

43070435 T1 7 0

43070565 T1 40 0

43070656 T1 33 0

43070788 T1 32 0

43070971 T1 11 0

43071329 T1 4 0

43071678 T1 34 0

43071777 T1 28 0

43072908 T3 39 0

43073089 T1 57 0

43073268 T2 43 0

43073285 T1 13 0

43073339 T1 14 0

43073614 T1 16 0

43073689 T1 4 0

43073785 T1 39 0

43074687 T2 29 0

43075883 T1 32 0

43076037 T1 25 0

43076735 T3 15 0

43077225 T1 23 0

43077472 T1 31 0

43077659 T1 9 0

43077706 T2 54 0

43078009 T1 20 0

43078143 T1 25 0

43078222 T1 36 0

43078423 T1 5 0

43078428 T1 67 0

43078711 T1 13 0

43078786 T1 17 0

43079146 T1 9 0

43079424 T1 26 0

43079521 T1 21 0

43079643 T1 32 0

43079740 T1 9 0

43080261 T1 24 0

43080344 T1 90 0

43081531 T1 27 0

43081892 T1 6 1

43082174 T1 30 0

43082309 T1 9 0

43082880 T1 17 0

43083006 T1 28 0

43083618 T1 16 0

43083787 T1 17 0

43084018 T1 5 2

43084478 T1 3 0

43084732 T1 26 0

43084778 T1 48 0

43084794 T1 28 0

43084898 T1 33 0

43084914 T1 23 0

43084939 T1 16 0

43085027 T1 24 0

43085139 T1 26 0

43085294 T1 19 0

43085312 T1 22 0

43085496 T2 19 0

43086671 T1 17 0

43086682 T1 8 0

43087365 T1 16 0

43087716 T1 20 0

43088008 T1 42 1

43088061 T2 5 0

43088095 T1 39 0

43088422 T2 22 0

43088843 T1 11 0

43089327 T1 35 0

43089857 T1 26 0

43090054 T1 11 0

43090348 T2 2 0

43090714 T1 23 0

43091580 T1 5 0

43091718 T1 7 0

43091829 T2 18 0

43092058 T1 6 0

43092139 T1 15 0

43092781 T2 16 0

43092828 T1 18 0

43093770 T1 14 0

43093860 T1 6 0

43093866 T1 23 0

43093894 T1 8 0

43094420 T1 1 0

43094650 T1 21 0

43094692 T1 53 0

43095774 T1 7 0

43096383 T1 18 0

43097173 T1 11 0

43098365 T1 12 0

43099460 T1 41 0

43099564 T1 4 0

43099693 T1 13 0

43100231 T2 20 0

43100286 T1 11 0

43101142 T1 25 0

43101167 T1 20 0

43101195 T1 13 0

43101448 T1 8 0

43101852 T1 31 0

43102290 T1 7 0

43102429 T1 12 0

43102558 T1 12 0

43102767 T1 3 0

43103508 T1 37 4

43103683 T2 20 0

43103710 T1 12 0

43104115 T1 8 0

43104711 T1 11 0

43104894 T1 22 0

43105044 T1 3 1

43105046 T1 26 0

43106082 T1 10 0

43106248 T1 21 0

43106707 T1 4 0

43106750 T1 4 0

43107170 T1 21 0

43107377 T1 16 0

43107735 T1 3 0

43107983 T1 13 0

43109140 T1 16 0

43109212 T1 31 0

43110058 T1 1 0

43110140 T1 14 0

43111788 T1 37 0

43112378 T1 11 0

43112661 T1 21 0

43112685 T1 28 0

43112813 T1 19 0

43113552 T1 9 0

43113590 T1 31 0

43113846 T1 25 0

43113989 T1 16 0

43114177 T1 7 2

43114247 T1 25 0

43114374 T2 26 0

43114464 T1 27 0

43115088 T1 31 0

43115140 T1 15 0

43115229 T1 24 0

43115334 T1 13 0

43115449 T1 27 0

43115816 T1 32 0

43116057 T3 58 3

43116346 T1 71 0

43116409 T1 15 0

43116494 T1 16 0

43116536 T1 47 0

43116543 T1 19 0

43117057 T1 8 0

43117417 T1 23 0

43118847 T3 3 1

43119068 T1 58 0

43119084 T1 36 0

43120092 T1 29 0

43120102 T2 45 0

43120761 T1 35 0

43120856 T1 7 0

43121165 T1 29 0

43121463 T1 31 0

43121563 T1 7 0

43121704 T1 14 1

43122849 T1 34 0

43122952 T1 8 0

43123220 T2 14 0

43123913 T1 8 0

43125569 T1 39 0

43126040 T1 14 0

43126198 T1 16 0

43126394 T1 22 0

43127325 T1 18 0

43127417 T1 90 0

43127442 T1 2 0

43127645 T1 13 0

43127966 T1 9 0

43128361 T1 5 0

43128732 T1 15 0

43129678 T1 11 0

43129689 T1 16 0

43129767 T1 21 0

43129778 T1 35 0

43129935 T1 13 0

43129972 T1 28 0

43130638 T1 27 0

43131468 T1 18 0

43131568 T1 13 0

43132114 T1 12 0

43133317 T1 11 0

43133551 T1 16 0

43133555 T1 18 0

43133812 T1 42 0

43133853 T1 13 0

43134033 T2 34 0

43134211 T1 28 0

43134603 T2 8 0

43135635 T1 25 0

43136203 T1 33 0

43136294 T1 26 0

43136598 T1 14 0

43138025 T2 17 0

43138049 T1 25 0

43138222 T1 34 0

43138338 T1 26 0

43139293 T1 25 0

43139554 T1 36 0

43139591 T1 19 0

43140186 T1 17 0

43140206 T1 22 3

43140739 T1 17 0

43141138 T1 21 0

43141372 T1 16 1

43141618 T1 9 0

43141801 T1 16 0

43142178 T1 11 0

43142256 T2 36 0

43142544 T1 6 0

43142839 T1 40 0

43142933 T1 26 0

43143170 T1 45 0

43143579 T1 17 8

43143965 T1 10 0

43143979 T1 8 0

43144168 T1 22 0

43144399 T1 22 0

43144457 T1 23 0

43144729 T1 29 0

43145481 T1 8 0

43146028 T1 29 0

43146100 T1 35 0

43146133 T1 29 0

43146213 T1 32 0

43146228 T1 58 0

43146375 T2 19 0

43146387 T1 10 0

43146401 T1 36 0

43146520 T1 18 0

43146528 T1 72 0

43146607 T3 15 0

43146630 T1 29 0

43146655 T1 8 0

43146656 T1 45 0

43146795 T1 12 0

43146816 T1 15 0

43147004 T1 23 0

43147009 T1 17 0

43147865 T1 28 0

43147927 T1 14 0

43147986 T1 6 0

43148426 T1 41 0

43148476 T1 9 0

43148585 T1 23 1

43148609 T1 6 0

43148734 T1 14 0

43148740 T1 30 0

43148747 T1 7 0

43148771 T1 6 0

43148859 T1 21 0

43149332 T1 6 0

43149491 T1 23 0

43149925 T1 14 0

43149991 T1 14 0

43150005 T1 7 0

43150138 T1 33 3

43150162 T1 4 0

43150402 T2 38 0

43150406 T1 32 0

43150500 T1 18 0

43150760 T1 16 0

43150774 T3 34 4

43150805 T1 17 0

43150876 T1 19 0

43150887 T2 29 0

43150908 T1 21 0

43150992 T1 38 0

43151744 T1 14 0

43151755 T1 35 0

43151809 T1 20 0

43151973 T2 31 0

43152008 T1 15 0

43152099 T2 21 0

43152106 T1 41 0

43152163 T1 60 0

43152168 T1 5 0

43152178 T1 27 0

43152234 T1 14 0

43152335 T1 5 0

43152366 T1 27 0

43152453 T1 26 0

43153575 T1 10 0

43154117 T1 5 0

43154268 T1 23 0

43154500 T1 32 0

43154831 T1 3 0

43154983 T1 1 0

43155028 T1 13 0

43155264 T1 40 0

43155319 T1 8 0

43155632 T1 26 0

43156157 T1 21 0

43156438 T1 39 0

43156463 T1 22 0

43156913 T1 15 0

43157620 T1 20 0

43157656 T1 18 0

43157863 T1 6 0

43157895 T2 23 0

43158334 T1 10 0

43158354 T1 21 0

43158852 T1 17 0

43158943 T2 20 0

43158964 T1 8 0

43159193 T1 21 0

43159628 T1 14 0

43159636 T1 24 0

43159665 T1 12 0

43159684 T1 31 0

43159691 T1 37 0

43159717 T1 14 0

43159727 T1 30 0

43159743 T1 8 0

43159802 T1 5 0

43159986 T1 6 0

43160177 T1 33 0

43160186 T1 25 0

43160188 T2 69 0

43160282 T2 1 0

43160329 T1 28 0

43160772 T1 7 0

43161084 T1 56 0

43161095 T1 21 0

43161386 T1 23 0

43161834 T1 31 0

43162006 T1 21 0

43162012 T1 17 0

43162209 T1 25 0

43162255 T1 24 0

43162282 T1 14 0

43162307 T1 35 0

43162355 T1 38 0

43162362 T1 35 0

43162602 T2 6 0

43162693 T1 13 0

43162702 T1 30 0

43162727 T3 33 0

43162776 T1 14 0

43162878 T3 23 4

43163181 T1 8 0

43163348 T1 18 8

43163440 T1 16 0

43163561 T1 38 0

43163772 T1 28 0

43163825 T1 21 0

43164446 T3 17 0

43165579 T1 12 0

43166385 T1 18 0

43166672 T1 32 0

43167047 T1 17 0

43167102 T1 16 0

43167156 T1 34 0

43167157 T1 9 0

43167509 T1 32 0

43167833 T1 24 0

43168384 T1 6 0

43168478 T1 5 0

43168688 T2 23 2

43169088 T1 32 0

43169200 T2 2 0

43169260 T1 22 0

43169461 T1 5 0

43169885 T1 16 0

43169915 T1 44 0

43170175 T1 23 0

43170238 T1 24 0

43170245 T1 16 0

43170639 T1 29 0

43170644 T1 19 0

43170830 T3 9 0

43171811 T1 24 0

43171841 T1 5 0

43171913 T1 29 0

43172014 T1 24 0

43172032 T1 4 0

43172050 T1 40 0

43172118 T1 27 0

43172845 T2 10 0

43173053 T1 10 0

43173175 T1 5 0

43173217 T1 50 0

43173287 T1 44 0

43173971 T1 28 0

43174075 T2 31 0

43174356 T1 10 0

43174363 T1 12 0

43174908 T1 5 0

43175587 T1 44 0

43176046 T1 31 0

43176168 T1 27 0

43176172 T1 28 0

43176184 T1 33 0

43176407 T3 24 0

43176492 T3 17 0

43176728 T1 35 0

43176998 T1 21 0

43177322 T1 35 0

43177342 T2 42 0

43177350 T1 46 0

43177627 T1 48 0

43177694 T1 32 0

43177740 T2 30 0

43177762 T3 30 1

43177874 T1 28 0

43178486 T1 39 0

43178491 T1 12 0

43178519 T1 29 0

43178523 T1 14 0

43178529 T1 49 0

43178744 T1 25 0

43181263 T1 24 0

43181276 T2 7 0

43181694 T1 15 0

43181720 T1 26 0

43181781 T1 21 0

43181793 T1 21 0

43181896 T1 31 0

43181993 T1 4 0

43182749 T1 10 1

43182757 T1 18 0

43182790 T2 34 0

43182898 T1 1 0

43182907 T1 23 0

43183071 T1 18 0

43183310 T1 15 0

43183519 T2 33 1

43183584 T1 31 0

43184012 T1 2 0

43184013 T1 8 0

43184053 T1 15 0

43184244 T1 34 0

43185776 T1 56 0

43186083 T1 36 0

43186095 T1 42 0

43186650 T1 27 3

43186928 T1 7 0

43188665 T1 15 0

43189054 T1 21 0

43189097 T1 27 0

43189099 T2 30 0

43189123 T3 39 0

43189281 T1 1 0

43189364 T1 22 0

43189761 T1 9 0

43190030 T1 3 0

43190722 T1 11 0

43191796 T1 34 0

43192763 T1 24 0

43192792 T1 44 0

43192896 T2 33 0

43193575 T1 12 0

43193839 T1 43 0

43193855 T2 17 0

43193860 T1 23 0

43194052 T1 29 0

43194059 T1 42 0

43194072 T3 12 0

43194104 T1 43 0

43194154 T1 20 0

43194976 T1 27 0

43197226 T1 15 0

43197420 T1 4 0

43197732 T1 16 0

43197817 T1 29 0

43197840 T1 39 0

43197843 T1 42 0

43198028 T1 26 2

43198947 T1 4 0

43199461 T4 22 0

43199602 T1 12 0

43199614 T1 12 0

43199623 T1 36 0

43199732 T2 11 0

43199742 T1 44 0

43199879 T1 4 0

43200134 T1 22 0

43200145 T1 40 0

43200224 T1 34 0

43200392 T1 25 0

43200545 T1 4 0

43200784 T2 18 0

43200790 T3 24 0

43200844 T1 33 0

43201139 T1 15 0

43201169 T1 19 0

43201193 T1 18 0

43201270 T1 16 0

43201299 T1 22 0

43201387 T1 7 0

43201547 T1 27 0

43201611 T1 19 0

43202317 T2 20 0

43202474 T1 30 0

43202651 T1 9 0

43202722 T1 14 0

43202900 T2 9 0

43203282 T1 44 0

43203714 T1 9 0

43204162 T1 33 0

43204168 T1 1 0

43204231 T1 9 0

43204321 T2 1 1

43205424 T1 22 0

43206207 T1 23 0

43206656 T1 23 1

43206875 T1 30 0

43207081 T1 18 0

43207607 T3 30 0

43208324 T2 38 0

43208420 T1 16 0

43208736 T1 34 0

43208746 T3 11 0

43209073 T1 22 0

43209719 T1 24 0

43209983 T1 22 0

43210019 T1 7 0

43210072 T1 21 0

43210091 T1 22 0

43210102 T2 41 0

43210120 T1 24 0

43210142 T1 10 0

43210150 T1 24 0

43210170 T1 17 0

43210223 T1 22 0

43210254 T1 23 0

43210351 T1 25 1

43210368 T1 33 0

43210459 T1 34 0

43210599 T1 31 0

43210747 T1 20 0

43210748 T1 34 0

43210761 T1 30 0

43210816 T1 17 2

43210903 T2 40 0

43210923 T1 21 0

43210938 T1 6 0

43210939 T2 28 0

43210989 T1 35 0

43211049 T3 26 2

43211052 T1 24 0

43211223 T1 23 0

43211335 T1 38 0

43211338 T1 37 0

43211342 T1 13 0

43211346 T3 33 0

43211364 T1 24 0

43211404 T2 36 0

43211414 T1 16 0

43211417 T1 6 0

43211585 T1 23 0

43211979 T1 29 0

43212062 T1 23 0

43212152 T1 3 0

43212292 T1 4 0

43212595 T1 29 0

43212997 T1 21 0

43213092 T1 29 0

43213451 T1 14 0

43213675 T1 27 0

43213834 T1 19 0

43214395 T1 2 0

43214555 T1 12 0

43215098 T1 28 0

43215342 T1 14 0

43215353 T1 26 0

43215370 T1 16 0

43215377 T1 38 0

43215379 T1 26 0

43215381 T1 34 0

43215394 T1 21 0

43215396 T1 20 0

43215939 T3 28 0

43216278 T1 22 0

43216377 T2 15 0

43216422 T1 22 0

43216504 T1 26 0

43216572 T1 12 0

43216591 T1 28 0

43217149 T2 24 0

43217249 T1 18 0

43217660 T1 28 0

43219306 T1 8 0

43219326 T1 31 0

43220133 T1 24 5

43220158 T2 15 0

43220324 T1 16 0

43220441 T1 6 0

43220546 T2 34 0

43220899 T1 26 0

43221405 T1 41 0

43221456 T1 33 1

43221653 T1 24 0

43221690 T2 21 0

43221936 T1 38 0

43222209 T1 6 0

43222241 T1 10 0

43222286 T1 29 0

43222504 T1 16 0

43222521 T1 34 0

43222537 T1 27 0

43222650 T1 25 0

43223182 T1 54 0

43223229 T1 36 3

43223338 T1 33 0

43223347 T1 27 0

43223746 T1 25 0

43223812 T1 18 0

43223816 T1 20 0

43223820 T1 14 0

43223841 T1 23 0

43223941 T1 16 0

43223969 T1 25 0

43224098 T1 12 0

43224435 T1 21 1

43225241 T1 23 0

43225263 T1 21 0

43225330 T1 17 0

43225435 T1 27 0

43225620 T1 23 0

43225623 T1 15 0

43225624 T1 11 0

43225625 T2 36 0

43225627 T1 17 0

43226166 T1 6 0

43226487 T1 36 0

43226500 T1 45 0

43226757 T1 28 0

43227016 T1 28 0

43227140 T1 36 0

43227197 T1 20 0

43227225 T1 60 0

43227618 T1 32 0

43227928 T1 27 0

43228061 T1 18 0

43228082 T1 28 0

43228192 T1 1 0

43228590 T1 46 0

43229375 T1 24 0

43229972 T1 30 0

43230365 T1 10 0

43230385 T1 24 0

43230640 T1 8 0

43230830 T1 41 0

43230836 T1 13 0

43231255 T1 33 0

43231365 T1 44 0

43232204 T1 16 0

43232278 T1 37 0

43232334 T2 5 0

43232595 T1 12 0

43233086 T1 36 0

43233368 T1 28 0

43233592 T1 29 0

43233972 T1 29 0

43234053 T1 38 0

43234248 T1 7 0

43234309 T1 13 0

43234894 T1 19 0

43235193 T1 22 0

43235377 T1 17 0

43235388 T1 21 0

43235604 T2 19 0

43235833 T3 19 0

44000052 T1 6 0

44000725 T1 4 0

44001032 T1 39 0

44001036 T1 11 0

44001051 T1 10 0

44001100 T1 13 0

44001193 T1 11 0

44001209 T1 18 0

44001231 T1 26 0

44001321 T1 32 0

44001730 T2 40 0

44001859 T1 24 0

44001910 T1 17 0

44002101 T1 6 0

44002189 T1 5 0

44002552 T1 27 0

44002889 T1 12 0

44003016 T1 23 0

44003406 T1 14 0

44003552 T1 33 0

44003882 T1 34 0

44003920 T1 4 0

44004041 T1 12 0

44004102 T1 18 0

44004105 T2 16 0

44004107 T1 7 0

44004112 T1 18 0

44004137 T3 16 0

44004244 T1 23 0

44004660 T1 26 0

44004784 T1 29 0

44005191 T1 19 0

44005669 T1 14 0

44005887 T1 22 0

44005925 T1 14 0

44005981 T1 72 0

44006127 T1 14 0

44006137 T1 42 0

44006139 T2 14 1

44006147 T1 31 0

44006885 T2 17 0

44007117 T1 42 0

44007748 T3 12 0

44008135 T2 17 0

44008647 T2 20 0

44008744 T1 17 0

44009011 T1 27 0

44009318 T1 26 0

44009759 T1 3 0

44011016 T1 10 0

44011037 T1 20 0

44011095 T1 17 0

44011104 T1 16 0

44011118 T2 31 0

44011120 T2 17 0

44011123 T3 12 0

44011126 T2 32 0

44011128 T1 23 0

44011156 T1 16 0

44011226 T1 17 0

44011245 T1 36 0

44011411 T1 15 0

44011715 T1 5 0

44011862 T1 34 0

44012220 T1 8 0

44012248 T1 29 0

44012654 T1 49 0

44012679 T1 17 0

44012694 T1 25 0

44013050 T1 14 0

44013053 T1 11 0

44013061 T1 9 0

44013098 T1 9 0

44013111 T1 8 1

44013205 T1 35 0

44013270 T1 31 0

44013271 T1 17 0

44013687 T1 31 0

44013694 T1 21 0

44013850 T1 34 0

44013851 T1 21 0

44014025 T1 30 1

44014175 T1 26 0

44014314 T1 6 0

44014405 T1 31 0

44014496 T1 3 0

44014560 T2 27 0

44015323 T1 5 0

44016000 T1 39 0

44016107 T2 23 0

44016159 T1 18 0

44016314 T1 53 0

44016728 T1 7 0

44016749 T1 6 0

44017882 T1 7 0

44018239 T1 30 0

44019824 T1 41 0

44020649 T2 11 0

44023415 T1 24 0

44023837 T1 27 0

44025087 T1 16 0

44025408 T1 26 0

44025749 T1 21 0

44026003 T2 59 0

44026044 T1 10 0

44026102 T3 6 0

44026188 T1 27 0

44026258 T1 29 0

44026312 T1 19 0

44026373 T1 18 0

44026411 T1 20 1

44026425 T2 13 0

44026531 T1 13 0

44026937 T1 10 2

44027034 T1 16 0

44027061 T1 37 0

44027088 T1 11 0

44027135 T1 26 0

44027357 T1 2 0

44027379 T1 21 0

44027401 T1 10 0

44027530 T1 39 0

44027626 T1 44 1

44027958 T3 20 1

44028108 T3 22 1

44028309 T1 38 0

44028551 T1 24 0

44029858 T1 7 1

44030263 T2 21 0

44030303 T1 27 0

44030418 T1 2 0

44030455 T1 23 0

44030590 T1 5 0

44030593 T2 13 0

44030732 T1 35 0

44031048 T1 24 0

44031052 T1 24 0

44032503 T1 22 0

44032841 T3 36 0

44033245 T1 29 0

44033484 T1 13 0

44033485 T1 28 0

44033491 T1 33 0

44033713 T1 35 0

44033798 T1 21 0

44033800 T3 21 0

44033801 T1 25 0

44033913 T1 33 0

44033964 T2 26 0

44034049 T1 24 0

44034152 T1 39 1

44034178 T1 7 0

44034191 T1 32 0

44034196 T3 22 2

44034198 T1 37 0

44034324 T1 8 0

44034568 T2 46 0

44034618 T1 6 0

44034619 T1 24 0

44034637 T1 8 0

44034651 T1 4 0

44034703 T1 35 0

44034956 T2 35 0

44036729 T1 23 0

44037056 T1 21 0

44037082 T1 32 0

44051401 T1 2 0

44142415 T1 48 0

46001134 T1 3 0

46002962 T1 8 0

46006532 T1 41 0

46006739 T3 39 0

46006848 T1 60 0

46007260 T1 47 0

46007742 T1 7 0

46008492 T3 11 1

46009022 T2 2 0

46009046 T1 13 0

46009052 T1 9 0

46010080 T3 4 1

46010081 T1 16 0

46010282 T1 6 0

46010293 T1 8 0

46010306 T1 6 0

46010661 T2 7 0

46011948 T1 5 0

46011982 T2 23 0

46012073 T1 7 0

46012112 T2 16 1

46012748 T1 18 0

46013223 T1 25 0

46013320 T1 6 0

46013378 T1 3 0

46013958 T2 37 0

46014137 T1 32 0

46014192 T2 8 0

46014240 T1 6 0

46014615 T1 31 0

46014636 T1 17 0

46014652 T3 33 0

46015056 T1 2 0

46015728 T1 6 0

46015743 T2 45 0

46016300 T1 13 0

46016318 T2 21 0

46016854 T1 11 0

46017173 T2 36 0

46017932 T2 6 4

46017943 T1 6 0

46018385 T1 18 0

46019847 T1 21 0

46020318 T1 18 0

46020497 T2 17 1

46020518 T2 10 0

46020982 T1 16 0

46021798 T2 26 0

46022490 T1 14 1

46022517 T3 11 0

46022529 T1 2 0

46022737 T1 19 0

46024171 T1 2 0

46024355 T1 35 0

46025023 T1 29 0

46025146 T2 11 0

46025342 T1 17 0

46025946 T1 1 0

46026565 T1 25 0

46026895 T3 21 0

46026901 T1 24 0

46026981 T1 1 0

46027059 T1 14 0

46028091 T2 6 0

46028969 T3 52 0

46029813 T1 15 0

46029960 T1 11 0

46030231 T1 2 0

46030859 T1 2 0

46030948 T2 17 0

46031326 T1 3 0

46031440 T1 10 0

46031446 T3 24 0

46031684 T1 10 0

46031876 T1 9 0

46031998 T1 8 0

46032016 T3 22 3

46032055 T1 6 0

46032180 T1 6 0

46032196 T1 5 0

46032252 T1 29 0

46032358 T2 18 0

46032832 T1 6 0

46032833 T1 4 0

46033024 T1 3 0

46033251 T1 1 0

46033265 T1 20 0

46033315 T2 24 0

46033325 T1 5 0

46033420 T1 2 0

46033759 T1 20 0

46034750 T1 1 0

46034764 T1 14 0

46034803 T2 4 0

46035325 T2 26 0

46035752 T1 5 0

46035936 T1 3 0

46035990 T2 31 1

46036986 T1 8 0

46037329 T1 3 0

46037333 T1 5 0

46037736 T1 37 0

46037747 T2 24 0

46038600 T1 4 0

46038948 T1 18 0

46040011 T1 15 0

46040084 T1 7 0

46040092 T3 23 0

46040576 T1 36 0

46040623 T2 23 0

46040773 T1 7 0

46041021 T1 14 1

46042087 T1 10 0

46042259 T1 12 0

46042311 T1 8 0

46042548 T1 3 0

46042627 T1 3 0

46042632 T1 39 0

46042761 T1 5 0

46042849 T1 10 0

46043814 T1 23 0

46044036 T2 24 0

46045174 T1 7 0

46045406 T1 2 0

46045637 T2 7 0

46045678 T1 22 0

46045689 T1 4 0

46046238 T2 6 0

46046401 T1 4 0

46046409 T1 2 0

46046421 T1 5 0

46047155 T1 21 0

46047164 T1 9 0

46047165 T1 29 0

46047173 T1 30 0

46047235 T3 7 0

46048013 T1 15 0

46048494 T1 5 0

46049240 T3 10 0

46049900 T1 19 0

46050274 T1 3 0

46051026 T2 7 2

46051244 T1 23 0

46051551 T1 6 0

46052048 T1 2 0

46052065 T1 2 0

46052074 T1 4 0

46052111 T3 3 0

46052185 T1 4 0

46053059 T1 6 0

46053167 T1 22 0

46053251 T1 22 0

46053391 T1 21 0

46053435 T3 23 1

46053448 T3 2 0

46053519 T2 19 0

46053549 T1 4 0

46053791 T1 5 0

46054593 T3 27 0

46054639 T1 7 0

46054671 T1 2 0

46054685 T1 1 0

46055130 T1 6 0

46055614 T1 2 0

46055844 T1 2 0

46056032 T1 15 0

46056177 T1 25 0

46056187 T1 7 0

46056318 T1 18 0

46056964 T1 36 0

46056977 T1 28 0

46057331 T2 12 0

46057546 T1 16 0

46057597 T3 16 0

46058067 T2 4 0

46058402 T2 5 0

46058490 T1 11 0

46059309 T1 7 0

46059485 T1 6 0

46059791 T1 49 0

46060066 T1 1 0

46060162 T1 22 0

46061132 T1 22 0

46061485 T1 5 0

46061521 T1 12 0

46062086 T1 5 0

46062207 T2 23 0

46062571 T1 38 0

46063088 T1 2 0

46063105 T1 2 0

46063114 T1 2 0

46063593 T3 3 0

46063846 T1 31 0

46064100 T1 14 0

46064438 T1 3 0

46064444 T1 35 0

46065345 T1 4 0

46066234 T2 3 0

46066714 T3 1 1

46066833 T1 13 0

46066932 T1 3 0

46066954 T1 36 0

46066984 T2 17 0

46067094 T1 16 0

46067108 T3 19 0

46067266 T1 10 0

46067818 T1 12 0

46068007 T1 5 0

46069320 T1 24 0

46069808 T1 45 0

46070568 T1 14 0

46071281 T1 13 0

46071664 T1 36 0

46071970 T1 12 0

46073031 T1 2 0

46073044 T1 9 0

46073402 T1 1 0

46073514 T1 5 0

46073604 T1 11 0

46073702 T1 9 0

46073864 T1 50 0

46073878 T2 4 0

46073959 T2 20 0

46074595 T2 7 0

46075184 T3 7 0

46075198 T2 2 0

46075230 T1 10 0

46075830 T1 14 0

46076028 T2 4 0

46076919 T1 3 0

46077548 T1 7 0

46077859 T2 1 0

46077867 T3 4 1

46077902 T1 3 0

46077923 T1 2 0

46078113 T1 6 0

46078179 T2 10 0

46078645 T1 21 0

46078797 T1 16 0

46078808 T1 21 0

46079261 T2 2 0

46079357 T1 4 0

46079579 T1 12 0

46080134 T1 2 0

46080350 T1 32 0

46081131 T3 3 0

46081153 T1 26 0

46081243 T4 23 0

46081267 T1 38 0

46081316 T1 10 0

46081811 T1 6 0

46082557 T1 5 0

46082576 T3 10 0

46083059 T2 15 0

46083873 T3 34 1

46084156 T1 7 0

46084298 T1 27 0

46084352 T1 18 0

46084360 T1 2 0

46084363 T1 12 0

46084680 T1 7 0

46084694 T1 16 0

46084704 T1 1 0

46084718 T1 34 0

46084794 T1 6 0

46087189 T1 5 0

46087419 T1 22 0

46087421 T1 8 0

46088015 T1 4 0

46088515 T2 15 0

46088629 T1 2 0

46088907 T2 26 0

46089069 T4 1 0

46089083 T1 3 0

46089229 T1 1 0

46089314 T1 23 0

46089330 T2 8 0

46089527 T1 13 0

46090159 T1 6 0

46090235 T1 2 0

46090381 T1 24 0

46090642 T1 11 0

46090736 T1 24 0

46090746 T1 37 0

46090794 T2 1 1

46091129 T2 13 0

46091211 T3 18 1

46092167 T2 37 0

46092329 T3 27 3

46092486 T1 1 0

46092824 T1 2 0

46092869 T2 2 0

46093225 T1 6 0

46093240 T1 12 0

46093250 T2 35 0

46093738 T1 5 0

46094198 T2 33 2

46094495 T1 22 0

46094969 T1 15 0

46096145 T1 17 0

46096356 T1 5 0

46096643 T1 18 0

46096890 T1 19 0

46097376 T1 1 0

46097985 T1 5 2

46098112 T1 19 0

46098241 T1 20 0

46099469 T1 17 0

46099532 T1 13 0

46100076 T1 13 0

46100089 T1 6 0

46100183 T2 2 0

46100374 T1 29 0

46100524 T1 3 0

46100976 T2 4 0

46101045 T1 6 0

46101102 T1 20 0

46101147 T1 22 0

46101409 T1 36 0

46101672 T1 23 0

46102246 T1 26 0

46102760 T1 9 0

46103142 T1 2 0

46103161 T1 11 0

46103489 T1 1 0

46104323 T2 27 0

46104493 T1 2 0

46104823 T1 5 0

46105202 T2 10 0

46105209 T1 8 0

46105222 T1 3 0

46105225 T1 13 0

46105362 T1 1 0

46105411 T1 32 0

46105416 T1 2 0

46105426 T1 2 0

46105441 T1 3 0

46107253 T1 2 0

46107415 T1 2 0

46107424 T2 13 0

46107535 T1 19 0

46107692 T1 11 0

46107913 T2 10 1

46108073 T1 42 0

46108346 T3 21 0

46108396 T1 8 0

46108535 T1 18 0

46108548 T1 12 0

46108835 T1 10 0

46108842 T1 8 2

46108966 T1 7 0

46109082 T1 7 0

46110372 T1 25 0

46110396 T2 15 0

46110632 T1 11 0

46111769 T2 12 0

46112361 T1 14 0

46112577 T1 3 0

46112602 T1 37 0

46113017 T1 24 0

46113124 T1 26 0

46113260 T3 37 1

46113283 T1 41 0

46113299 T2 49 0

46113890 T1 2 0

46113992 T1 21 0

46114399 T1 2 0

46114722 T1 9 0

46115250 T1 35 0

46115625 T1 17 0

46115661 T1 19 0

46115701 T1 22 0

46115928 T1 35 0

46115939 T1 8 0

46116613 T1 28 0

46117000 T1 1 0

46117698 T1 20 0

46117939 T1 8 0

46118066 T1 10 0

46118417 T1 10 0

46118883 T1 24 0

46119383 T1 22 0

46119691 T1 2 1

46119778 T1 20 0

46120014 T1 4 0

46120050 T2 1 0

46120916 T1 4 0

46120931 T1 4 0

46121432 T1 20 0

46121779 T1 19 0

46122420 T1 14 0

46123486 T1 6 0

46123587 T1 4 0

46123918 T2 2 0

46124516 T1 34 0

46124664 T1 30 0

46124757 T1 21 0

46125118 T3 7 1

46125489 T1 4 0

46125492 T3 4 0

46125504 T1 2 0

46125932 T1 1 0

46126968 T3 9 0

46127700 T1 7 0

46127874 T1 2 0

46128470 T1 38 0

46128634 T1 26 0

46128863 T2 1 0

46128933 T1 1 0

46129113 T2 10 0

46129318 T1 8 0

46129345 T1 16 0

46129430 T1 32 0

46130257 T2 14 0

46130274 T1 8 0

46130725 T1 2 0

46130759 T1 5 0

46131333 T1 9 0

46131585 T1 22 0

46131658 T1 9 0

46131768 T1 32 0

46131987 T1 3 0

46132090 T1 21 0

46132926 T1 2 0

46133049 T1 2 0

46133245 T1 13 0

46134056 T1 13 0

46134765 T2 6 0

46135097 T1 32 0

46135199 T1 1 0

46135375 T3 72 0

46135593 T1 5 0

46135622 T3 7 0

46135700 T1 1 0

46135751 T1 6 0

46135873 T1 16 0

46137258 T2 8 0

46138031 T2 35 2

46138647 T1 15 0

46138697 T1 7 0

46139317 T1 12 0

46140097 T3 7 0

46140914 T1 2 0

46141473 T1 3 0

46142046 T1 10 0

46142311 T1 21 0

46142642 T1 17 0

46143087 T1 5 0

46143470 T1 19 0

46143824 T1 6 0

46143853 T1 26 0

46144081 T2 30 0

46144099 T1 11 0

46144306 T3 10 0

46144653 T1 16 0

46144706 T1 27 0

46144824 T2 14 0

46144843 T1 25 0

46144863 T1 23 0

46144864 T1 20 1

46145120 T1 5 0

46145364 T3 2 2

46145468 T1 7 0

46145780 T1 3 0

46145906 T2 28 0

46147128 T1 5 0

46147615 T1 2 0

46147988 T1 26 0

46148549 T1 3 0

46148832 T1 8 0

46148848 T1 3 0

46148987 T1 22 0

46149083 T1 40 0

46149124 T1 14 0

46149143 T1 3 0

46149866 T2 90 0

46149911 T1 6 0

46150011 T1 10 0

46150202 T1 12 0

46150321 T1 23 0

46150631 T1 15 0

46150695 T1 2 0

46151337 T1 4 1

46151757 T1 10 0

46151814 T1 32 0

46151961 T1 16 0

46152050 T3 36 0

46152177 T1 3 3

46153526 T1 18 0

46154173 T1 11 0

46154583 T1 18 0

46154613 T1 5 0

46154634 T1 5 0

46154661 T1 6 0

46154674 T1 3 0

46154907 T1 14 0

46155015 T1 23 0

46155028 T1 17 0

46155068 T1 37 0

46155819 T2 5 0

46155866 T1 8 0

46155956 T1 3 0

46156072 T2 22 0

46156164 T1 34 0

46156413 T1 15 0

46156647 T1 1 0

46156745 T1 15 1

46156865 T1 4 0

46156921 T1 4 0

46156994 T1 10 0

46156995 T2 2 0

46157037 T1 2 0

46157603 T1 2 0

46157623 T1 2 0

46157890 T2 4 0

46157972 T1 15 0

46158181 T1 10 0

46158276 T2 18 0

46158293 T1 19 0

46158371 T1 30 0

46158615 T1 28 0

46158619 T1 17 0

46158630 T1 15 0

46158747 T1 18 0

46158777 T1 10 1

46158846 T1 38 0

46159359 T2 5 0

46160108 T1 7 0

46160387 T3 24 2

46160433 T1 24 0

46160781 T1 16 0

46161546 T1 14 0

46161550 T1 2 0

46161655 T1 53 0

46161836 T1 3 0

46162073 T2 14 0

46162177 T1 8 0

46162726 T2 25 0

46163239 T1 21 0

46163249 T1 25 0

46163401 T1 6 0

46163437 T1 19 0

46163610 T1 42 0

46164207 T3 1 1

46164214 T1 6 0

46164443 T1 1 0

46164620 T1 65 0

46164635 T2 7 0

46164989 T1 53 0

46165266 T1 8 0

46165493 T1 29 0

46165509 T1 20 0

46166041 T1 2 0

46166300 T1 23 0

46166522 T1 2 0

46166769 T1 17 0

46166775 T1 1 0

46166960 T2 6 0

46167075 T1 36 0

46167260 T1 24 0

46169319 T1 6 0

46169431 T1 10 0

46169681 T2 28 0

46170000 T1 8 0

46170379 T1 16 0

46170497 T1 24 0

46170514 T1 1 0

46171595 T1 53 0

46171689 T2 11 0

46172045 T1 11 0

46172487 T2 8 0

46172586 T1 3 0

46173562 T2 13 0

46173592 T2 19 3

46173888 T1 16 0

46173917 T1 8 0

46174169 T1 14 0

46174180 T1 5 0

46174190 T3 18 0

46174204 T1 1 0

46174253 T1 3 0

46174492 T1 17 0

46174518 T2 6 0

46174523 T1 13 0

46174877 T1 7 0

46174973 T3 6 0

46175086 T2 7 0

46176007 T1 23 0

46176031 T1 19 0

46176046 T1 2 0

46176049 T1 23 0

46176098 T1 15 0

46176110 T1 24 0

46176388 T2 15 2

46176404 T1 8 1

46176469 T1 3 0

46176496 T1 5 0

46176593 T1 17 0

46176637 T2 16 0

46176647 T1 24 0

46177468 T2 4 0

46177519 T1 5 0

46178067 T1 3 0

46178340 T1 12 0

46178598 T1 10 0

46178778 T2 1 0

46179034 T1 2 0

46179083 T1 40 0

46179215 T1 10 1

46179227 T2 4 1

46179271 T1 2 0

46179466 T1 2 0

46179584 T1 2 0

46179594 T1 5 0

46180131 T1 4 0

46180355 T1 3 0

46180383 T2 7 0

46181101 T1 15 0

46181245 T1 4 0

46181933 T1 10 0

46182264 T1 33 0

46182284 T1 10 0

46182573 T1 15 0

46182812 T1 11 0

46182837 T3 9 0

46182928 T2 18 0

46183166 T1 22 0

46183583 T1 2 0

46184494 T1 7 0

46184873 T3 8 0

46184880 T3 6 0

46184915 T1 6 0

46184964 T1 11 2

46185153 T1 5 0

46185818 T1 41 0

46186230 T1 1 0

46186865 T2 7 0

46186909 T2 6 0

46187097 T1 15 0

46187218 T2 37 0

46187227 T1 13 0

46187455 T1 1 0

46187564 T1 1 0

46187656 T1 30 0

46188190 T1 1 0

46188263 T1 2 0

46188387 T1 2 0

46189370 T1 6 0

46189382 T1 22 0

46189495 T1 15 0

46189904 T3 5 0

46190718 T1 10 0

46190731 T3 8 1

46191271 T2 44 0

46191510 T1 2 0

46191969 T1 15 0

46192475 T1 1 0

46192601 T2 30 0

46192937 T1 51 3

46193367 T1 32 0

46193379 T1 7 0

46193682 T1 17 0

46194540 T1 1 0

46194584 T1 2 0

46195028 T1 2 0

46195108 T3 28 0

46195134 T2 25 0

46195162 T1 3 0

46195469 T2 45 0

46195543 T3 12 0

46195555 T1 6 0

46195901 T1 17 0

46196017 T1 8 0

46196077 T1 1 0

46196107 T1 17 0

46196170 T1 12 0

46196589 T1 1 0

46196654 T2 27 0

46196896 T4 15 0

46197025 T1 14 0

46197366 T1 20 0

46197488 T1 7 0

46197897 T1 27 0

46198348 T1 12 0

46198625 T1 12 0

46198801 T1 16 0

46199847 T1 3 0

46199911 T1 6 0

46200067 T1 1 0

46200076 T1 2 0

46200100 T3 1 0

46200169 T1 4 0

46200192 T1 6 0

46200258 T1 2 0

46200927 T1 16 0

46201068 T1 9 0

46202640 T1 1 0

46202688 T1 16 0

46202787 T2 10 0

46202830 T1 9 0

46202960 T1 3 0

46202969 T2 2 0

46203158 T1 4 0

46203218 T1 14 0

46203317 T1 7 0

46203478 T1 19 0

46203519 T1 5 0

46203536 T2 16 0

46203553 T1 10 0

46203618 T1 9 1

46204273 T1 9 0

46204698 T1 15 5

46204746 T1 1 0

46204990 T1 16 0

46205094 T1 11 0

46205351 T1 14 0

46206002 T1 3 0

46206403 T1 11 0

46206451 T1 7 0

46206908 T3 10 10

46207052 T1 37 0

46207320 T1 14 0

46208008 T1 2 0

46208170 T1 7 0

46208813 T1 12 2

46209006 T1 14 0

46209766 T1 4 0

46210576 T2 22 0

46210981 T1 12 0

46211335 T1 2 0

46211596 T2 6 0

46211872 T2 19 0

46211940 T1 2 0

46211945 T1 11 0

46212671 T1 9 0

46212905 T2 4 0

46213480 T1 34 0

46213921 T1 8 0

46213936 T1 2 0

46213938 T1 26 0

46213941 T1 2 0

46213992 T3 8 0

46213997 T1 10 0

46214236 T1 18 0

46214281 T1 15 0

46214287 T2 12 0

46214681 T1 14 0

46214931 T1 13 0

46215037 T1 8 1

46215246 T1 2 0

46215411 T1 4 0

46216070 T1 24 0

46216620 T1 12 0

46217601 T1 6 0

46217666 T1 6 0

46217686 T1 27 0

46217699 T1 22 0

46217710 T2 19 0

46217748 T1 11 0

46217885 T1 9 0

46218053 T1 51 0

46218282 T1 22 0

46218473 T1 12 0

46218896 T1 19 0

46218951 T2 28 0

46219011 T1 10 0

46220808 T1 5 0

46220997 T1 7 0

46222315 T1 7 0

46223550 T1 9 0

46223676 T1 8 0

46223695 T1 15 0

46223975 T1 7 0

46224020 T1 22 0

46224478 T2 33 0

46224481 T1 2 0

46224524 T2 1 0

46224719 T2 35 0

46224750 T2 18 0

46226081 T1 6 0

46226178 T1 15 0

46226621 T1 4 0

46227865 T1 4 0

46228110 T1 3 0

47000076 T2 10 0

47001477 T1 6 0

47001955 T1 4 0

47001973 T1 11 0

47002258 T1 1 0

47002274 T1 2 0

47002469 T1 21 0

47002703 T1 11 0

47002722 T1 25 0

47003372 T1 1 0

47003403 T3 1 0

47004208 T2 8 0

47004526 T2 4 1

47004950 T1 45 2

47006009 T2 9 0

47006143 T1 2 0

47006583 T2 8 0

47006597 T2 1 0

47007442 T1 4 1

47008643 T1 4 0

47010276 T1 6 0

47010331 T1 6 0

47010622 T1 13 0

47010754 T1 11 0

47011340 T1 19 0

47011542 T1 3 0

47011621 T1 1 1

47011632 T1 6 0

47011886 T1 15 0

47012068 T1 11 0

47012539 T1 6 0

47012876 T1 5 0

47013248 T1 20 0

47013334 T1 16 0

47013973 T3 2 0

47014197 T1 9 0

47014216 T2 10 0

47014312 T1 8 0

47014344 T1 26 0

47014589 T1 6 0

47014697 T1 11 0

47014898 T1 14 0

47015282 T4 1 1

47015979 T3 2 0

47016283 T1 40 0

47016293 T1 6 0

47016297 T1 2 0

47016308 T1 9 0

47016342 T1 16 0

47016663 T1 4 0

47016819 T1 13 0

47016844 T1 2 0

47016889 T1 18 0

47017216 T1 22 0

47017991 T1 2 0

47018687 T1 16 0

47019809 T1 15 0

47019849 T1 22 0

47020131 T1 13 0

47020285 T1 3 0

47020893 T1 4 0

47021249 T1 1 0

47021518 T1 15 0

47021994 T1 5 0

47022054 T1 5 0

47022206 T1 12 0

47022431 T1 3 0

47022449 T1 26 0

47022458 T1 10 2

47022878 T1 7 0

47023579 T1 24 0

47024177 T1 4 0

47024708 T1 6 0

47025654 T2 3 0

47025751 T1 13 0

47026289 T1 9 0

47026342 T1 19 0

47026410 T1 24 0

47026794 T2 14 0

47028104 T1 13 0

47028336 T1 3 0

47028419 T1 6 0

47028479 T1 10 0

47028582 T1 10 0

47029095 T1 19 0

47029124 T1 4 0

47029359 T1 3 0

47029788 T1 6 0

47029955 T1 23 0

47032328 T3 4 0

47032670 T1 9 0

47032816 T1 24 0

47033528 T3 38 0

47034052 T1 18 1

47034468 T1 6 0

47034935 T1 17 0

47035740 T1 7 0

47035873 T3 24 0

47035904 T1 4 0

47035917 T1 5 0

47035929 T1 3 0

47036210 T1 10 0

47036571 T1 3 0

47036624 T1 2 0

47038114 T1 18 0

47039279 T1 7 0

47039785 T1 5 0

47040996 T2 2 0

47041609 T1 3 0

47041728 T1 2 0

47042168 T1 4 0

47042467 T1 14 0

47042667 T1 9 0

47042670 T1 13 0

47042706 T1 10 0

47042712 T1 1 0

47042715 T3 7 0

47042727 T1 9 0

47042734 T1 5 3

47042737 T1 4 0

47042913 T1 32 0

47043243 T1 1 0

47043855 T1 14 0

47044055 T1 4 0

47045295 T1 26 0

47046158 T2 23 0

47046211 T1 4 0

47046468 T3 3 0

47046586 T1 8 0

47047325 T2 44 7

47047854 T1 25 0

47048500 T2 20 0

47049228 T1 5 0

47049291 T1 14 0

47049665 T1 6 0

47050215 T1 19 0

47050470 T2 19 0

47050582 T3 18 0

47050700 T3 5 0

47051259 T1 4 0

47051756 T1 16 0

47051989 T1 23 0

47053260 T3 31 0

47053514 T3 13 5

47053731 T1 25 0

47053784 T1 16 0

47053842 T2 30 0

47054718 T1 4 0

47054782 T1 4 0

47055039 T1 1 0

47055417 T1 10 0

47056769 T1 22 0

47056990 T1 41 0

47057425 T1 4 0

47058019 T1 15 0

47058082 T1 5 0

47058158 T1 18 0

47058164 T2 2 0

47058332 T1 10 0

47058656 T1 11 0

47059237 T1 4 0

47059447 T1 12 0

47059781 T1 21 0

47061933 T1 29 0

47062124 T1 7 0

47063506 T1 56 0

47063533 T1 24 0

47063632 T1 2 0

47063877 T1 22 0

47064125 T1 30 0

47064190 T3 13 0

47064365 T1 6 0

47064556 T1 14 0

47065406 T1 2 0

47065578 T1 3 0

47065759 T1 15 0

47066035 T1 2 0

47066087 T1 1 0

47066138 T1 10 0

47066785 T1 5 2

47066969 T2 20 0

47067179 T1 7 0

47067586 T1 22 2

47067780 T1 3 0

47067789 T1 4 0

47067902 T1 10 0

47068134 T1 11 1

47069489 T1 15 0

47069820 T1 1 0

47070583 T1 50 0

47070680 T1 4 0

47070683 T1 6 0

47070716 T1 23 0

47071106 T1 18 0

47071133 T3 28 0

47071682 T1 5 0

47071842 T1 10 0

47072238 T1 12 0

47072959 T1 8 0

47073045 T1 6 0

47073224 T1 10 0

47073283 T3 7 0

47073576 T1 4 0

47073656 T1 33 0

47073920 T1 5 0

47074086 T2 2 0

47074312 T1 22 0

47074363 T1 20 0

47074405 T1 8 0

47074668 T1 9 0

47075119 T3 15 1

47075120 T1 7 0

47075398 T1 4 0

47075695 T2 22 1

47075821 T1 5 0

47075878 T1 2 0

47075890 T2 8 0

47075902 T1 18 1

47075910 T2 5 0

47075965 T1 15 0

47076650 T1 13 0

47076691 T1 14 0

47077087 T1 10 0

47077179 T1 22 0

47077406 T1 6 0

47077471 T1 3 0

47078604 T1 17 0

47078949 T1 9 0

47079554 T1 16 0

47080280 T3 11 0

47080388 T1 6 0

47080738 T2 4 0

47081091 T1 3 0

47081111 T3 7 3

47081529 T1 3 0

47081592 T1 8 0

47081595 T1 17 0

47081749 T1 20 0

47082071 T1 12 0

47082424 T1 3 0

47083094 T2 15 0

47083126 T1 20 0

47083488 T1 8 0

47083978 T1 20 0

47084003 T1 3 0

47084068 T1 2 0

47084463 T3 4 0

47084953 T3 43 9

47085107 T1 7 0

47085509 T1 4 0

47086668 T3 2 2

47086716 T1 2 0

47087221 T3 5 0

47087643 T2 8 0

47087828 T1 9 0

47087837 T1 4 0

47087914 T1 14 0

47087969 T1 17 0

47088462 T3 22 1

47088491 T1 10 0

47088680 T1 2 0

47089247 T1 15 0

47089549 T2 16 4

47090020 T1 15 0

47090105 T1 13 1

47090361 T1 35 0

47090382 T1 2 0

47090417 T1 1 0

47090439 T1 30 0

47090889 T1 22 0

47090916 T1 10 0

47091118 T1 39 0

47091390 T2 3 0

47091636 T1 13 0

47092078 T1 1 0

47092171 T1 14 0

47092271 T1 8 0

47092429 T1 9 0

47092553 T1 4 0

47092810 T1 1 0

47093657 T1 3 0

47094143 T1 12 0

47094209 T1 2 0

47095068 T1 2 0

47095149 T1 3 0

47095634 T2 8 0

47096388 T1 16 0

47096729 T2 4 0

47096824 T1 26 0

47096874 T1 4 0

47097686 T1 4 0

47097795 T1 2 0

47097804 T1 2 0

47098279 T1 11 0

47099001 T1 7 0

47099037 T1 28 0

47099092 T1 29 0

47099866 T1 2 0

47100959 T1 4 0

47100978 T2 7 0

47101077 T1 25 0

47101455 T1 8 0

47101502 T1 44 0

47101684 T1 8 0

47102398 T3 7 0

47102462 T1 4 0

47102509 T1 2 0

47103446 T3 2 0

47104971 T1 1 0

47105231 T3 14 0

47105508 T3 5 0

47105520 T1 9 0

47105697 T1 16 0

47105889 T1 9 0

47106252 T3 23 1

47106330 T2 5 1

47107554 T1 9 0

47108482 T1 4 4

47108703 T1 7 0

47108758 T1 4 0

47109044 T1 8 0

47109225 T1 36 0

47109280 T1 11 0

47109473 T1 36 0

47109621 T1 21 4

47110064 T2 9 0

47110091 T1 30 0

47111300 T3 7 0

47111326 T1 14 0

47111971 T1 4 0

47112363 T1 34 0

47112800 T2 1 0

47113224 T1 16 0

47113299 T2 12 0

47114228 T1 22 0

47114362 T1 12 0

47115369 T1 12 0

47115815 T1 2 0

47115908 T3 14 0

47116339 T1 1 0

47117511 T1 17 0

47118516 T1 9 2

47118776 T1 21 0

47118932 T1 1 0

47119081 T1 3 0

47119388 T1 1 0

47119796 T1 12 0

47120022 T2 6 0

47120705 T1 40 0

47120857 T1 6 0

47120988 T1 6 0

47121464 T1 8 0

47122823 T1 20 0

47123647 T1 24 0

47123927 T1 3 0

47123951 T1 4 0

47124197 T1 1 0

47124471 T1 1 0

47125380 T1 17 0

47125697 T1 5 0

47126130 T1 6 0

47126153 T1 24 0

47126162 T1 18 0

47126241 T1 20 0

47126586 T1 5 0

47126608 T1 9 0

47127039 T2 17 0

47127343 T1 28 2

47127455 T1 18 0

47127556 T1 5 0

47127615 T1 13 0

47127751 T1 5 2

47128841 T1 24 0

47130011 T1 23 0

47130280 T1 20 0

47130689 T1 7 0

47131352 T1 10 0

47131937 T1 22 0

47132847 T1 16 0

47132857 T1 14 0

47133989 T1 24 0

47134412 T4 10 6

47134792 T1 3 0

47135722 T1 11 0

47135919 T1 39 0

47136109 T1 4 0

47136666 T1 8 0

47138619 T2 28 0

47139618 T1 12 0

47140800 T1 10 0

47141325 T1 23 0

47141471 T1 2 0

47141695 T1 5 0

47142551 T2 1 0

47143029 T1 21 0

47143366 T2 56 0

47143527 T1 26 0

47144394 T1 33 0

47145513 T1 2 0

47145707 T1 6 0

47145759 T2 8 2

47145828 T2 6 0

47146049 T1 18 0

47146138 T1 11 0

47146223 T1 1 0

47146506 T1 11 0

47147006 T1 20 0

47147335 T1 7 0

47148045 T1 2 0

47148057 T1 7 0

47148657 T3 35 0

47148801 T2 13 0

47148941 T1 57 0

47149286 T1 1 0

47149747 T1 7 0

47149846 T1 25 0

47150273 T1 9 0

47150683 T1 6 0

47151001 T3 6 0

47152016 T1 10 0

47152275 T1 4 0

47152708 T3 3 0

47152723 T1 5 0

47152734 T1 2 0

47153464 T1 2 0

47153843 T1 17 2

47154118 T1 3 0

47154137 T1 10 0

47154479 T1 6 0

47154681 T1 30 0

47155716 T1 12 1

47157448 T1 1 0

47157834 T1 6 0

47157973 T1 12 0

47158758 T1 2 1

47159050 T2 5 1

47160877 T1 7 0

47161488 T1 6 0

47161629 T1 2 0

47162075 T2 2 0

47162085 T1 3 0

47162337 T1 25 0

47162716 T1 21 0

47162957 T1 15 0

47163434 T1 4 0

47163656 T1 2 0

47164203 T1 11 0

47166453 T1 7 0

47166984 T2 17 0

47166991 T1 12 0

47167022 T1 10 0

47167075 T1 10 0

47167089 T1 7 0

47167476 T1 8 0

47167666 T1 12 0

47167908 T4 5 3

47168030 T1 2 0

47168563 T1 5 0

47168567 T1 16 0

47169288 T2 24 0

47169677 T1 17 0

47170357 T1 42 0

47170614 T1 9 0

47170923 T1 25 0

47171238 T1 6 0

47171409 T1 5 0

47171447 T1 32 0

47171736 T1 9 0

47171792 T1 34 0

47171793 T1 6 0

47171875 T1 37 0

47171884 T1 15 0

47172069 T1 1 0

47172983 T2 34 0

47173694 T1 18 0

47173928 T1 15 0

47174035 T1 4 0

47174630 T1 11 0

47174731 T1 2 0

47174763 T1 3 0

47175072 T1 31 0

47175119 T1 9 0

47175850 T1 2 0

47175959 T1 4 1

47176134 T1 8 0

47176507 T1 6 0

47176687 T1 3 0

47176785 T1 12 0

47177565 T1 13 0

47177576 T1 29 0

47177642 T1 14 0

47179500 T1 25 0

47179749 T1 5 0

47180612 T1 8 0

47181468 T1 4 0

47181573 T1 3 0

47181941 T3 33 0

47182419 T1 11 0

47182467 T1 3 0

47182629 T1 13 0

47182751 T2 10 0

47183024 T2 12 0

47183615 T1 1 0

47183727 T1 7 0

47185370 T1 19 0

47186654 T3 4 0

47187867 T1 31 0

47188029 T2 27 0

47188155 T1 1 0

47188584 T2 16 0

47190885 T1 8 0

47190886 T2 1 0

47190892 T1 11 0

47191152 T1 21 0

47191344 T1 5 0

47191830 T1 2 0

47191892 T1 47 0

47193510 T3 9 0

47195182 T3 6 0

47195693 T2 38 0

47195995 T1 30 0

47196821 T1 7 0

47197070 T1 20 0

47197891 T2 2 0

47198996 T1 57 0

47199155 T1 29 0

47199821 T1 2 0

47200835 T1 22 0

47201736 T1 22 0

47202204 T1 2 0

47203437 T1 12 0

47204687 T2 78 0

47205708 T1 7 0

47210768 T1 7 0

47212424 T1 4 0

47212650 T1 24 0

47213215 T1 7 0

47213618 T1 22 0

47213967 T3 6 0

47214805 T1 28 0

47214830 T1 13 0

47214880 T1 13 0

47215744 T1 25 0

47215789 T2 15 0

47215952 T1 2 0

47217444 T1 12 0

47217505 T2 5 0

47218620 T1 1 0

47218938 T1 3 0

47219454 T1 14 0

47219659 T1 5 0

47220721 T1 19 0

47220725 T1 1 0

47221769 T1 7 0

47223709 T1 2 0

47224352 T1 1 0

47224444 T1 2 0

47224731 T1 12 0

47225276 T1 7 0

47226226 T1 3 0

47227113 T1 24 0

47227123 T1 26 0

47228049 T1 19 0

48002377 T1 6 0

48002690 T1 19 0

48002819 T1 19 0

48002914 T1 7 0

48004396 T1 27 0

48004912 T1 22 0

48005574 T1 1 0

48005892 T2 4 0

48006297 T1 25 0

48006557 T1 18 0

48006721 T1 13 0

48006859 T1 2 0

48006916 T1 27 2

48007112 T3 7 0

48009576 T1 5 0

48009967 T1 24 0

48010551 T1 41 0

48010863 T1 9 0

48011951 T1 6 0

48015135 T1 23 0

48015475 T2 6 0

48016360 T1 12 0

48016785 T1 26 0

48019226 T1 9 0

48022388 T1 3 0

48022444 T1 2 0

48023896 T1 38 0

48024731 T1 11 0

48025255 T1 2 0

48026845 T1 3 0

48027709 T1 18 0

48028880 T1 23 0

48029452 T1 20 0

48031394 T1 38 2

48031511 T1 10 0

48032362 T1 16 0

48033239 T1 1 0

48033463 T1 7 0

48033834 T1 5 0

48034484 T2 1 0

48034894 T1 3 0

48035218 T1 26 0

48035552 T1 4 0

48035769 T1 15 0

48035885 T1 9 0

48036628 T1 14 0

48037469 T2 7 0

48037675 T1 1 0

48038465 T1 8 0

48039221 T1 4 1

48039744 T3 1 0

48039889 T1 4 0

48042435 T1 7 0

48042862 T1 14 0

48043496 T1 23 1

48045549 T1 5 0

48046393 T2 32 0

48046519 T1 1 0

48047643 T1 22 0

48050046 T1 8 0

48050896 T1 19 0

48052065 T1 25 0

48052706 T1 38 0

48055404 T1 8 0

48055612 T2 22 0

48057104 T1 11 0

48057489 T2 21 0

48058898 T1 1 0

48059321 T1 15 0

48059479 T1 40 0

48060185 T2 2 0

48060361 T1 29 0

48060712 T1 1 0

48064437 T1 4 0

48064786 T1 10 0

48065467 T1 19 0

48065648 T1 33 0

48065740 T1 17 0

48065968 T1 5 0

48068054 T1 1 0

48068705 T1 32 0

48069132 T1 10 0

48069220 T1 16 0

48070447 T1 2 0

48070762 T1 7 0

48070865 T1 4 0

48070966 T3 8 0

48072652 T1 9 0

48072653 T1 17 0

48073321 T3 6 0

48073774 T1 3 0

48074247 T1 1 0

48075570 T1 5 0

48075745 T2 49 0

48076302 T1 7 0

48076310 T3 18 0

48076349 T1 24 0

48076817 T1 25 0

48077130 T1 7 0

48077822 T1 16 0

48077988 T1 20 0

48078198 T1 17 0

48078350 T1 12 0

48078421 T1 6 0

48079231 T1 19 1

48080818 T2 18 0

48082707 T1 11 0

48083537 T2 18 0

48083629 T1 6 0

48085500 T1 4 0

48085877 T1 3 0

48087211 T1 43 2

48089319 T1 21 0

48089546 T1 17 1

48089648 T1 18 0

48090910 T1 3 0

48092760 T1 3 0

48092989 T1 33 0

48096182 T1 2 0

48097421 T1 15 0

48097449 T1 7 0

48097473 T1 15 0

48098145 T2 2 0

48098390 T1 2 0

48099968 T1 1 0

48101500 T1 1 0

48103581 T1 3 0

48103821 T1 2 0

48104617 T1 2 0

48106132 T1 13 0

48106376 T1 5 0

48106551 T1 7 0

48107571 T1 2 2

48109040 T1 8 0

48109418 T1 5 0

48110112 T1 4 0

48110915 T1 8 0

48111283 T1 9 0

48112837 T1 16 0

48113044 T1 22 0

48113418 T1 19 0

48113795 T1 2 0

48114469 T1 6 0

48115634 T3 8 0

48115817 T1 5 0

48116047 T1 2 0

48117486 T3 11 0

48118516 T1 9 0

48118872 T1 5 0

48121977 T1 2 0

48122163 T1 5 0

48123831 T1 10 0

48125208 T1 16 0

48125254 T1 6 0

48126303 T1 6 0

48126771 T1 5 0

48126780 T1 4 0

48127428 T1 21 0

48128320 T2 23 0

48128465 T1 2 0

48130244 T1 1 0

48131909 T1 11 0

48132343 T1 13 0

48132561 T1 27 0

48132826 T1 19 1

48135389 T1 11 0

48135672 T2 1 0

48136980 T1 16 0

48138866 T3 26 0

48139729 T1 24 0

48140345 T1 15 0

48141421 T1 6 0

48141449 T1 10 0

48141545 T1 6 0

48142203 T1 7 0

48142429 T1 19 0

48142749 T1 8 0

48143477 T1 3 0

48145300 T1 3 0

48146115 T2 33 7

48146119 T1 25 0

48146285 T1 19 0

48146411 T1 4 0

48146558 T1 22 0

48148377 T1 12 0

48149167 T1 4 0

48149705 T1 19 0

48150023 T1 7 0

48150026 T1 33 0

48150139 T1 8 0

48150359 T1 6 0

48152124 T1 7 0

48153282 T1 10 0

48153503 T1 6 0

48153546 T1 12 0

48153992 T1 5 0

48155395 T2 2 0

48156170 T1 22 0

48157455 T1 4 0

48158557 T1 7 0

48173925 T1 3 0

48175149 T1 13 0

49097893 T1 29 0

49097910 T2 26 0

49098138 T1 20 0

49098547 T1 35 0

49098704 T1 3 0

49098812 T1 24 0

49098834 T1 14 0

49098871 T1 15 0

49099000 T1 8 0

49099028 T1 7 0

49099040 T1 15 0

49099418 T2 6 0

49099629 T1 4 0

49099659 T1 4 0

49099750 T1 19 0

49100155 T1 4 0

49100306 T2 17 0

49100546 T1 15 0

49100657 T3 12 0

49100664 T1 14 0

49100682 T1 9 0

49100701 T3 9 0

49100735 T1 11 0

49100784 T1 6 0

49101531 T1 13 0

49101545 T1 8 0

49101807 T1 7 0

49102004 T1 7 0

49102012 T1 7 0

49102101 T2 24 0

49102115 T1 43 0

49102367 T1 9 0

49102567 T1 9 0

49102847 T1 5 0

49103001 T2 13 0

49104141 T1 6 0

49104231 T2 29 0

49104246 T1 22 1

49104253 T1 24 0

49104981 T1 30 0

49105202 T1 37 0

49105203 T1 18 0

49105422 T2 24 0

49105524 T1 43 0

49105550 T2 47 0

49105656 T1 32 0

49105713 T1 44 0

49105759 T1 44 0

49106281 T1 3 0

49106665 T1 17 0

49106681 T1 1 0

49106773 T1 13 0

49106835 T1 10 0

49106884 T1 20 0

49106894 T1 12 0

49107059 T3 21 2

49107508 T1 19 0

49107593 T1 33 0

49107778 T1 19 0

49108021 T1 19 0

49108580 T1 1 0

49108588 T1 5 0

49108759 T1 1 0

49109073 T2 11 0

49109078 T1 6 0

49109140 T2 11 0

49109236 T1 2 0

49109673 T1 9 0

49109936 T1 10 0

49110001 T1 16 0

49110005 T2 13 0

49110087 T3 2 0

49110877 T2 5 0

49110936 T1 5 0

49111070 T1 9 0

49111817 T1 25 0

49111830 T1 7 0

49112107 T1 20 0

49112889 T1 3 0

49113940 T1 43 0

49114327 T2 1 0

49114333 T1 3 0

49114341 T1 10 0

49115105 T1 11 0

49115350 T1 31 0

49115355 T1 14 0

49115602 T3 30 0

49115762 T1 15 0

49116206 T1 8 0

49116411 T1 13 0

49116419 T1 7 0

49116554 T1 5 0

49116563 T2 25 2

49116760 T1 20 0

49116804 T1 18 0

49116913 T1 24 0

49116931 T1 24 0

49117290 T1 2 0

49117292 T1 7 0

49117304 T1 9 0

49117310 T1 10 0

49117397 T1 5 0

49118077 T1 2 0

49118087 T2 9 0

49118101 T1 50 0

49118222 T1 7 0

49118405 T2 12 0

49118464 T1 2 0

49118515 T3 17 1

49118785 T3 38 0

49118866 T3 1 0

49119468 T1 4 0

49119748 T1 8 0

49119761 T1 32 0

49120329 T1 30 0

49120846 T1 7 0

49120863 T1 1 0

49120874 T1 22 0

49121009 T1 13 0

49121215 T1 14 0

49121358 T1 29 0

49121371 T2 34 1

49121969 T1 9 0

49122349 T3 1 0

49122431 T1 2 0

49122822 T1 60 0

49123081 T1 34 0

49123090 T2 30 0

49123312 T3 19 0

49123341 T1 35 3

49123358 T2 5 0

49124182 T1 4 0

49124197 T1 34 0

49124204 T1 7 0

49124643 T2 11 0

49124653 T3 3 3

49125161 T1 20 0

49125170 T1 22 0

49125608 T1 13 0

49125625 T1 11 0

49126320 T3 1 0

49126624 T1 40 0

49126663 T1 47 0

49126676 T1 19 0

49127060 T1 7 0

49127430 T2 32 0

49127469 T3 23 3

49127904 T2 20 0

49128410 T1 3 0

49128740 T1 20 0

49129131 T1 2 0

49129166 T1 6 0

49129704 T1 18 0

49129755 T1 4 0

49130296 T1 11 0

49130609 T1 12 0

49130737 T1 7 0

49131436 T1 1 0

49131652 T1 25 0

49132222 T1 7 0

49132254 T1 12 0

49132465 T1 29 0

49132726 T1 10 0

49132755 T2 25 0

49132951 T1 15 0

49133037 T1 6 0

49134545 T1 4 0

49134649 T1 34 0

49135207 T1 15 0

49135426 T1 35 0

49135492 T1 16 0

49135742 T1 9 0

49135748 T1 11 0

49135800 T1 17 0

49135890 T1 15 0

49135938 T1 29 0

49136079 T2 18 0

49136095 T1 16 0

49136126 T1 10 0

49136167 T2 24 0

49136361 T1 18 0

49137064 T1 2 0

49137264 T1 3 0

49137935 T2 20 0

49138447 T1 10 0

49138507 T2 7 0

49138578 T1 11 0

49138775 T1 3 0

49138975 T1 13 0

49139059 T1 2 0

49139368 T3 11 0

49139630 T1 1 0

49140482 T1 9 0

49140516 T1 20 0

49140913 T1 11 0

49141531 T1 12 0

49141677 T1 48 0

49141744 T1 25 0

49141831 T4 51 0

49142334 T1 23 0

49142335 T2 13 0

49142700 T1 8 0

49142705 T3 4 0

49142749 T1 6 0

49143380 T1 15 0

49143847 T1 18 0

49144000 T1 5 0

49144856 T1 27 0

49144860 T1 29 0

49145104 T1 3 0

49145137 T1 25 0

49145671 T1 19 0

49145753 T1 22 0

49146044 T3 23 0

49146136 T1 17 0

49146672 T1 3 0

49146995 T1 17 0

49147141 T1 4 0

49147242 T1 32 1

49148200 T1 20 0

49149226 T1 4 0

49150115 T1 33 0

49151257 T1 20 0

49151266 T3 16 0

49152707 T1 15 0

49153271 T1 13 0

49153323 T2 6 0

49153578 T1 6 0

49153677 T1 8 0

49153690 T1 21 0

49153799 T1 8 0

49154416 T1 7 0

49154432 T3 31 0

49154436 T1 36 0

49155067 T2 12 0

49155198 T1 4 0

49155927 T2 13 0

49155935 T1 15 0

49155951 T1 3 0

49156062 T1 32 0

49156379 T2 4 0

49156490 T2 49 0

49156505 T1 54 0

49156510 T1 29 0

49157612 T1 22 0

49158199 T1 8 0

49158208 T1 2 0

49158792 T1 14 0

49159137 T1 12 0

49159151 T1 16 0

49159377 T1 18 0

49159904 T2 11 0

49160187 T2 23 0

49160509 T1 4 0

49160542 T1 12 0

49160592 T2 14 0

49160686 T2 9 0

49161303 T1 10 0

49161338 T1 15 0

49161347 T1 11 0

49161564 T1 8 1

49161719 T1 12 0

49161959 T1 11 0

49162094 T1 7 0

49162106 T1 15 0

49162221 T1 17 0

49162352 T1 17 0

49162467 T1 2 0

49162654 T1 14 0

49163433 T3 17 0

49163461 T1 20 0

49163979 T1 15 0

49164452 T1 2 0

49164709 T1 2 0

49165564 T1 10 0

49165578 T1 24 0

49165595 T2 28 0

49165606 T1 31 0

49165733 T1 8 0

49165773 T1 4 0

49166174 T1 12 0

49166602 T1 16 0

49166685 T1 27 0

49167002 T1 28 0

49167042 T1 8 0

49167050 T1 28 0

49167069 T2 16 0

49167092 T1 8 0

49167116 T1 17 0

49167148 T1 18 0

49167338 T2 16 1

49167378 T2 6 0

49167875 T1 4 0

49168088 T1 6 0

49168290 T1 4 0

49168441 T1 8 0

49169342 T1 10 0

49169670 T1 13 0

49169777 T1 17 0

49169933 T1 14 0

49170046 T4 9 0

49170122 T1 28 0

49170300 T1 1 0

49170527 T2 23 0

49170599 T1 12 0

49170635 T1 18 0

49170934 T3 12 0

49172040 T1 19 0

49172219 T1 5 0

49172529 T1 22 0

49172749 T1 11 1

49173020 T3 4 0

49173534 T1 7 0

49173540 T1 5 0

49174672 T1 15 0

49175206 T1 17 0

49175528 T1 41 0

49175542 T1 26 0

49175547 T1 6 5

49176224 T1 6 0

49176328 T1 24 0

49176737 T1 4 0

49176748 T1 9 0

49176822 T2 17 0

49177167 T1 16 0

49177640 T1 32 0

49177647 T1 27 0

49178747 T1 40 1

49178768 T1 30 0

49178777 T1 38 0

49178924 T1 12 0

49179647 T1 6 0

49179740 T1 17 0

49180116 T1 10 0

49180628 T1 27 0

49180908 T1 22 0

49181181 T1 18 0

49181553 T2 35 0

49181560 T1 9 0

49181810 T1 31 0

49182392 T1 31 0

49182614 T1 7 0

49183396 T1 6 0

49183640 T2 15 0

49183885 T1 19 0

49183959 T1 6 0

49184029 T1 30 0

49184137 T1 5 0

49184148 T2 3 0

49184506 T1 4 0

49184594 T1 30 0

49185257 T3 4 0

49185501 T1 25 0

49185940 T1 14 0

49186170 T1 65 0

49186184 T1 27 0

49186308 T2 8 0

49186840 T1 4 0

49186944 T1 35 0

49187031 T2 7 0

49187248 T1 5 0

49187678 T1 25 0

49187911 T1 10 0

49187945 T1 4 0

49188050 T1 16 0

49188060 T1 8 0

49188077 T1 10 0

49188229 T1 5 0

49188547 T1 9 0

49188959 T1 28 0

49189132 T1 3 0

49189782 T1 15 0

49189796 T1 12 0

49189802 T1 8 0

49189882 T1 17 0

49190218 T1 28 0

49190731 T3 5 0

49190746 T1 7 0

49191433 T1 33 0

49192033 T1 8 0

49192903 T1 37 0

49193355 T1 8 0

49193880 T1 28 0

49193933 T1 20 0

49193957 T3 22 0

49194449 T2 6 0

49194811 T1 27 0

49195034 T1 5 0

49195416 T1 32 0

49195423 T1 24 0

49195435 T1 22 0

49195565 T1 13 0

49195592 T1 9 0

49196517 T1 59 0

49196544 T1 7 0

49196562 T1 12 0

49196806 T1 4 0

49197149 T3 22 0

49197161 T3 85 0

49197171 T3 17 0

49197660 T1 36 0

49197670 T1 28 0

49198272 T1 27 0

49198299 T2 34 0

49198511 T2 3 0

49198602 T1 20 0

49198827 T1 2 0

49199432 T1 15 0

49199701 T1 44 1

49199714 T1 31 0

49200610 T2 34 0

49201366 T1 16 0

49201701 T1 9 0

49202097 T1 1 0

49202182 T1 38 0

49202260 T2 2 0

49202276 T1 14 0

49203203 T2 47 0

49203544 T1 13 2

49204113 T1 26 0

49204123 T3 1 0

49204524 T1 14 0

49204905 T4 6 0

49205120 T1 14 3

49205321 T1 10 0

49205337 T1 23 0

49205634 T1 11 0

49205684 T1 28 0

49205787 T2 21 1

49205985 T1 2 0

49206066 T1 11 0

49206521 T1 35 0

49206556 T1 29 0

49207150 T1 13 0

49207256 T2 7 0

49207310 T1 3 0

49207565 T1 4 0

49207593 T1 22 0

49207676 T1 10 0

49207861 T1 20 0

49208121 T1 34 0

49208219 T1 16 0

49208484 T1 10 1

49208495 T2 20 0

49208616 T1 25 0

49208628 T1 17 0

49209017 T1 12 0

49209272 T1 69 0

49209445 T1 27 0

49209466 T1 30 0

49209476 T1 13 0

49209811 T1 18 0

49210361 T1 2 0

49210443 T2 7 0

49210631 T1 6 0

49210920 T1 6 0

49210988 T1 3 0

49211691 T1 12 0

49212554 T1 3 0

49212805 T1 27 0

49212877 T1 9 0

49213235 T1 20 0

49213248 T1 13 0

49214242 T1 23 0

49214904 T2 13 2

49214914 T1 3 0

49215050 T1 3 0

49215161 T2 38 0

49215166 T2 14 0

49215181 T2 5 0

49215318 T1 14 0

49216081 T1 42 0

49216498 T1 22 0

49216520 T1 28 0

49216612 T1 2 0

49216719 T1 4 0

49216745 T2 3 0

49217278 T1 4 0

49217537 T1 8 0

49217982 T1 11 0

49218133 T1 13 0

49218320 T1 5 0

49218945 T1 20 0

49219132 T1 12 0

49220052 T1 37 0

49220224 T1 12 0

49221090 T1 23 0

49221106 T1 27 0

49221123 T1 24 0

49221131 T1 2 0

49221464 T1 10 0

49222306 T1 15 0

49222457 T1 6 0

49222520 T1 20 0

49222814 T2 9 0

49223566 T3 6 0

49224612 T2 44 0

49224625 T2 90 0

49224635 T3 22 0

49224882 T1 28 0

49225557 T2 3 0

49225608 T1 3 0

49226007 T1 16 0

49226018 T1 30 0

49226154 T3 36 0

49226320 T1 12 0

49226850 T1 13 0

49227893 T1 1 0

50000365 T1 26 0

50000374 T1 37 0

50000732 T1 18 0

50000750 T1 13 0

50001001 T1 12 0

50001807 T1 14 0

50001850 T1 8 0

50002039 T1 32 0

50002710 T1 3 0

50003463 T1 42 0

50003529 T2 30 0

50003540 T1 44 0

50003848 T1 31 0

50003917 T1 8 0

50004467 T1 3 0

50004552 T1 19 0

50004585 T1 13 0

50004593 T1 8 0

50005138 T1 4 0

50005151 T4 27 1

50005629 T1 4 0

50005712 T3 4 0

50006327 T1 13 0

50006457 T1 3 0

50006513 T1 23 0

50006569 T1 19 0

50007350 T1 3 0

50007417 T1 6 0

50007647 T1 7 0

50007663 T3 2 0

50007887 T1 36 0

50007893 T2 6 0

50007991 T1 8 0

50008014 T1 10 0

50008098 T1 21 0

50008533 T1 10 0

50009198 T1 27 0

50009280 T3 31 0

50009481 T1 4 1

50009634 T1 15 0

50010026 T1 16 0

50010044 T1 5 0

50010068 T1 18 0

50010447 T1 25 0

50010976 T2 6 0

50010985 T1 2 0

50011159 T4 7 0

50011245 T1 12 0

50011474 T1 5 0

50011635 T1 11 0

50011650 T1 19 0

50012147 T1 18 0

50012892 T1 5 0

50013331 T1 19 0

50013351 T1 46 0

50013584 T3 30 0

50013749 T1 9 0

50014237 T1 2 1

50014489 T1 2 0

50014498 T1 8 1

50015290 T1 8 0

50015713 T1 11 0

50015744 T1 27 0

50015750 T1 21 0

50016746 T3 10 0

50016764 T1 5 0

50016795 T1 14 0

50017500 T1 4 0

50017523 T1 23 0

50017529 T2 16 0

50017533 T1 3 0

50017540 T1 22 0

50017541 T2 29 0

50017552 T1 6 0

50017602 T1 7 0

50017612 T1 10 0

50019147 T1 10 0

50019391 T1 5 0

50019414 T1 19 0

50019661 T1 10 0

50019774 T1 32 0

50020094 T1 9 0

50020128 T1 4 0

50020141 T1 10 0

50020247 T3 9 0

50020359 T1 2 0

50020365 T3 2 0

50020576 T1 7 0

50021282 T1 7 0

50021632 T1 9 0

50021693 T1 14 0

50022055 T1 1 0

50022107 T1 1 0

50022290 T3 21 0

50023371 T1 11 0

50023511 T1 9 0

50023920 T1 21 0

50024009 T2 17 0

50024094 T3 24 0

50024192 T1 1 0

50024635 T1 10 0

50024670 T1 12 0

50024678 T1 8 0

50025749 T1 21 0

50025897 T2 25 0

50026100 T1 25 0

50026341 T1 6 0

50026373 T1 12 0

50026425 T2 19 0

50026685 T1 5 0

50026826 T1 44 0

50026835 T1 19 0

50027220 T1 33 0

50027230 T1 7 0

50027305 T2 5 0

50027564 T1 23 0

50027575 T1 20 0

50027971 T3 9 0

50028280 T1 8 0

50028299 T1 18 1

50028306 T1 10 0

50028341 T1 20 0

50028365 T1 5 0

50028374 T1 5 0

50028719 T1 8 0

50028750 T1 7 0

50028821 T1 7 0

50029053 T3 7 3

50029065 T2 17 0

50029086 T1 10 0

50029640 T1 30 0

50029817 T1 9 0

50030034 T1 8 0

50030048 T2 10 0

50030754 T1 11 0

50031307 T1 13 0

50031965 T1 5 0

50031974 T1 38 0

50032079 T1 11 0

50032213 T2 19 0

50032931 T1 1 0

50032936 T1 32 0

50033178 T1 18 0

50033390 T1 32 0

50033634 T3 30 0

50034096 T2 8 0

50034240 T4 2 2

50034551 T1 6 0

50034711 T1 18 0

50034732 T1 5 0

50034802 T1 15 0

50035065 T1 25 0

50035831 T1 16 0

50036326 T1 18 0

50036672 T1 8 0

50036823 T1 32 0

50036834 T1 16 0

50036984 T3 6 0

50037094 T1 2 0

50037368 T1 4 0

50037373 T1 5 0

50037381 T1 13 0

50037388 T2 7 0

50037406 T1 3 0

50037784 T1 22 0

50037807 T2 24 0

50037957 T1 4 0

50038268 T1 3 0

50038314 T1 12 0

50038325 T2 14 0

50038727 T1 23 0

50039346 T1 7 0

50039540 T3 26 0

50039937 T2 42 0

50039942 T2 19 0

50040091 T2 12 0

50040099 T1 10 0

50041335 T2 25 0

50041345 T1 14 0

50041359 T2 28 0

50041563 T1 13 0

50042076 T1 10 0

50042463 T3 3 1

50042645 T1 59 1

50042762 T1 10 0

50042795 T1 5 0

50042987 T1 38 0

50043266 T1 3 0

50043277 T1 14 0

50043288 T1 9 0

50043291 T1 22 0

50043470 T1 3 0

50043505 T1 23 0

50044183 T1 4 0

50044506 T2 22 0

50045014 T1 3 0

50045042 T1 32 0

50045202 T1 2 0

50045482 T1 40 0

50045517 T1 27 0

50045960 T1 9 0

50045964 T1 40 0

50045969 T1 15 0

50046549 T1 26 0

50046970 T3 18 0

50046992 T1 14 0

50047124 T1 4 0

50047241 T3 25 0

50048270 T1 2 0

50048401 T1 2 0

50048418 T3 20 0

50048527 T2 28 0

50048659 T1 39 0

50048971 T1 12 0

50049053 T1 15 0

50049208 T2 27 3

50049276 T1 22 14

50049279 T1 13 0

50049384 T1 2 0

50050843 T1 4 0

50051015 T1 16 0

50051019 T1 29 0

50051791 T1 10 0

50051919 T1 23 0

50052127 T1 9 0

50052394 T1 10 0

50054768 T1 11 0

50055675 T1 31 0

50055973 T1 2 0

50055974 T1 2 0

50056472 T2 20 2

50056913 T1 9 0

50056955 T1 7 0

50057224 T1 3 0

50057544 T1 19 0

50058631 T2 45 0

50058731 T1 33 0

50059775 T2 2 0

50059780 T1 29 0

50059906 T1 17 0

50059934 T1 3 0

50059982 T1 36 0

50060349 T1 19 0

50060453 T1 29 0

50060457 T1 29 0

50060719 T1 6 0

50061124 T1 28 0

50061962 T1 4 0

50062275 T3 44 0

50062277 T1 17 0

50062762 T1 5 0

50062765 T1 13 0

50062768 T1 2 0

50062772 T1 6 0

50062807 T1 29 0

50062894 T1 30 0

50063021 T1 12 0

50063197 T1 11 0

50063202 T1 11 0

50063203 T1 6 0

50063626 T1 9 0

50063818 T3 10 0

50063990 T1 6 0

50064181 T1 3 0

50064498 T1 8 0

50064804 T2 19 0

50065332 T1 18 0

50065424 T1 21 0

50066635 T1 18 0

50066757 T1 8 0

50066952 T1 5 0

50066955 T1 3 0

50067008 T1 5 0

50067172 T1 12 0

50067174 T1 7 0

50067683 T1 5 0

50067871 T1 30 0

50067874 T1 37 0

50067875 T1 38 0

50067878 T1 25 0

50068042 T3 18 2

50068223 T2 4 0

50068265 T1 8 0

50068400 T1 4 0

50068486 T1 19 1

50068752 T2 7 0

50068864 T1 34 0

50069107 T2 9 0

50069201 T2 13 0

50069384 T1 3 0

50069422 T1 21 0

50069936 T1 15 0

50070102 T1 35 1

50070212 T3 36 0

50070216 T1 2 0

50070368 T1 15 0

50070408 T1 40 0

50070629 T1 9 0

50070921 T3 35 0

50070973 T2 8 0

50071476 T1 18 0

50071952 T1 9 0

50072216 T1 26 0

50072887 T1 32 0

50072990 T1 28 0

50073072 T2 21 0

50073135 T1 2 0

50073136 T1 4 0

50073581 T3 33 0

50073756 T1 5 0

50073840 T3 33 0

50074158 T1 21 0

50074161 T1 28 0

50074241 T1 21 0

50075066 T1 13 0

50075116 T1 16 0

50075213 T1 3 0

50075216 T1 2 0

50075523 T1 4 0

50075587 T3 18 0

50075616 T1 16 0

50075971 T1 8 0

50076142 T1 6 0

50076325 T1 3 0

50076752 T1 31 0

50076755 T1 20 0

50076824 T1 6 0

50076829 T1 6 0

50077032 T1 12 0

50077035 T2 10 0

50077038 T1 8 0

50077250 T1 3 0

50077254 T1 7 0

50077344 T1 21 0

50077558 T1 21 2

50077811 T2 7 0

50077997 T1 13 1

50079557 T1 24 0

50079769 T1 11 0

50080190 T3 4 0

50080346 T1 17 0

50080349 T1 22 0

50080351 T1 10 0

50080357 T3 13 0

50080490 T1 21 0

50080492 T1 28 0

50080497 T1 30 0

50080498 T1 22 0

50080941 T1 18 0

50081233 T1 26 0

50081386 T1 20 0

50081841 T1 22 0

50082291 T3 15 2

50082320 T1 16 0

50082321 T2 7 0

50082489 T1 5 0

50082577 T1 33 0

50082777 T1 2 0

50082779 T1 3 0

50082780 T1 4 0

50082781 T1 5 0

50083053 T1 25 0

50083111 T3 5 0

50083151 T3 13 0

50083289 T2 6 0

50083678 T1 15 0

50083803 T1 7 0

50084541 T1 7 0

50084544 T3 26 0

50084730 T1 34 0

50084731 T1 46 0

50084888 T3 15 0

50085107 T1 16 0

50085138 T1 7 0

50085166 T1 16 0

50085418 T2 14 0

50085534 T1 6 0

50085698 T1 8 0

50085791 T1 5 0

50085841 T1 5 0

50086115 T1 32 0

50086217 T2 25 0

50086698 T1 16 0

50087139 T1 14 0

50087613 T1 8 0

50087834 T1 7 0

50087899 T1 2 0

50087982 T2 14 0

50087985 T1 25 0

50088139 T1 5 0

50088333 T1 3 0

50088386 T1 14 0

50088608 T1 3 0

50088652 T1 11 0

50088935 T1 11 0

50089068 T1 1 0

50089069 T2 1 0

50089184 T1 15 0

50089249 T3 14 0

50089252 T1 6 0

50089255 T1 10 0

50089589 T1 12 0

50089779 T1 10 0

50089936 T1 6 0

50090028 T1 37 0

50090029 T1 17 0

50090783 T1 3 0

50091296 T1 38 0

50091915 T1 15 0

50092517 T1 2 0

50092720 T1 26 9

50093021 T1 25 0

50093225 T1 18 0

50093997 T1 8 0

50093999 T1 1 0

50094098 T1 84 0

50094100 T1 18 0

50094320 T2 33 1

50094534 T1 16 0

50094588 T2 6 0

50094832 T1 21 1

50095120 T1 4 0

50095202 T1 34 1

50095412 T4 9 0

50095571 T1 19 0

50096010 T1 11 0

50096013 T1 3 0

50096206 T1 9 0

50096208 T1 5 0

50096210 T1 6 0

50096276 T3 21 0

50096279 T1 20 0

50096282 T1 22 0

50096388 T1 11 0

50096500 T1 12 0

50097190 T1 12 0

50097277 T1 7 0

50097320 T1 4 0

50097426 T1 12 0

50097613 T1 7 0

50097842 T1 23 0

50097854 T2 2 0

50097951 T3 4 0

50098203 T1 14 0

50098408 T1 23 0

50098650 T1 15 0

50098818 T2 45 0

50099229 T1 27 1

50099811 T1 17 0

50100103 T1 24 0

50100170 T1 16 0

50101067 T1 1 0

50101364 T3 7 0

50101936 T2 4 0

50102075 T2 5 0

50102286 T2 20 0

50102508 T2 27 0

50102684 T1 4 0

50102901 T1 10 0

50103138 T1 9 0

50103196 T2 10 0

50103201 T2 25 5

50103740 T1 8 0

50103967 T1 7 0

50103968 T2 9 0

50103969 T1 24 0

50103972 T3 2 0

50104098 T3 7 0

50104575 T1 21 0

50104618 T1 8 0

50104710 T1 17 0

50104924 T1 2 0

50105307 T1 8 0

50105519 T1 22 0

50105522 T3 23 0

50105523 T1 1 0

50105694 T1 34 0

50105935 T1 6 0

50106337 T1 21 0

50106684 T1 20 0

50106773 T3 26 0

50106955 T1 19 0

50106962 T1 3 0

50107116 T1 24 0

50108098 T1 10 0

50108522 T1 16 0

50108552 T1 5 0

50108934 T1 10 0

50108936 T1 3 0

50109029 T1 18 0

50109139 T1 22 0

50109140 T1 5 0

50109333 T1 47 4

50109591 T1 42 0

50109593 T1 5 0

50109680 T2 23 0

50109704 T2 7 0

50109766 T2 59 0

50109905 T1 5 0

50109908 T1 9 0

50109962 T1 25 0

50110164 T1 7 0

50110367 T1 8 0

50110798 T1 13 0

50110893 T1 14 0

50110894 T1 3 0

50110897 T1 8 0

50110980 T3 12 0

50111156 T1 3 0

50111285 T1 32 0

50111723 T1 3 0

50112088 T1 10 0

50113284 T2 2 0

50113712 T1 16 0

50115073 T2 29 0

50115812 T1 9 0

50115814 T1 8 0

50115815 T1 17 0

50115818 T1 9 0

50115960 T3 9 0

50115979 T1 11 0

50116089 T1 3 0

50116307 T1 14 0

50116350 T3 9 0

50116459 T1 4 0

50116849 T1 26 0

50117136 T1 11 0

50117217 T1 13 0

50117844 T1 12 0

50118276 T3 20 0

50118471 T2 46 0

50118477 T1 36 0

50119348 T3 25 0

50119760 T1 6 0

50119761 T1 15 0

50120025 T1 8 0

50120256 T3 29 0

50120271 T1 23 0

50120697 T2 36 0

50120947 T1 6 0

50121091 T1 2 0

50121199 T2 16 2

50121208 T2 4 0

50121363 T1 2 0

50121365 T1 2 0

50121421 T1 22 0

50121920 T1 4 0

50122177 T1 7 0

50122243 T1 3 0

50122252 T1 4 0

50122844 T1 19 0

50123758 T1 19 0

50123777 T1 3 0

50124591 T1 10 0

50126021 T1 9 0

50127254 T1 21 0

50127258 T1 22 0

50127457 T1 13 0

50127460 T1 4 0

50127547 T1 6 0

50127981 T1 40 0

50128075 T1 4 0

50128078 T1 5 0

50128368 T1 18 0

50128369 T2 17 0

50128413 T1 10 1

50128493 T1 32 0

50128841 T1 5 0

50129137 T3 5 0

50129152 T1 47 0

50129304 T1 30 1

50129502 T3 18 0

50129749 T1 13 0

50130582 T2 20 0

50130591 T1 25 0

50130884 T1 20 0

50130885 T2 13 0

50130887 T1 21 0

50131142 T1 13 0

50131304 T1 3 1

50131429 T1 17 0

50131550 T2 12 0

50131557 T1 9 0

50131571 T1 4 0

50131657 T1 13 2

50131782 T1 8 0

50131856 T2 9 0

50131857 T1 10 0

50131858 T1 12 0

50131897 T2 11 1

50131899 T2 15 0

50132163 T1 2 0

50132209 T2 17 0

50132277 T1 3 1

50132750 T1 14 1

50132872 T1 12 3

50133466 T1 25 0

50133561 T1 28 0

50133664 T3 15 0

50133769 T2 18 0

50133841 T3 11 0

50133842 T1 4 0

50133843 T1 7 0

50133965 T2 35 0

50134065 T1 8 0

50134402 T1 26 0

50134492 T2 38 0

50134506 T1 53 1

50134557 T1 19 0

50134630 T3 58 0

50134838 T1 13 0

50134839 T1 6 0

50134945 T1 14 0

50134980 T2 13 0

50134983 T1 4 0

50135331 T1 12 0

50135334 T1 21 0

50135410 T1 23 0

50135683 T1 12 0

50135729 T1 7 0

50135770 T1 5 0

50135773 T1 9 0

50135889 T1 15 0

50136317 T1 36 0

50136885 T1 3 0

50137091 T3 9 1

50137094 T3 15 0

50137360 T1 27 0

50137422 T1 31 0

50137425 T1 25 0

50137486 T1 12 0

50137489 T1 22 0

50137891 T2 30 2

50138485 T2 16 0

50138710 T2 10 0

50139143 T1 10 0

50139238 T1 14 0

50139755 T1 3 0

50139871 T1 22 0

50139915 T1 25 0

50140213 T1 6 0

50140216 T1 4 0

50140903 T1 4 0

50140906 T1 7 0

50140909 T1 26 0

50140910 T1 8 0

50140912 T2 9 0

50141222 T1 18 0

50141387 T1 2 0

50141750 T1 12 0

50142081 T1 3 0

50142119 T1 10 0

50142210 T1 11 0

50142369 T1 24 0

50142371 T1 20 0

50142661 T1 9 0

50142682 T2 5 0

50142954 T1 5 0

50143349 T4 1 1

50143416 T1 26 0

50143727 T3 6 0

50143733 T1 6 0

50144074 T1 9 0

50144077 T1 1 0

50144079 T1 3 0

50144081 T1 6 0

50144226 T1 3 0

50144733 T2 32 0

50144737 T1 11 0

50144739 T1 20 0

50144934 T1 14 0

50145470 T1 28 0

50145522 T1 7 0

50145707 T2 31 0

50146055 T1 10 0

50146371 T1 32 0

50146420 T1 9 0

50146572 T1 5 0

50146624 T3 11 0

50146791 T2 3 0

50146859 T1 6 0

50146860 T1 5 0

50146861 T1 6 0

50147752 T1 36 0

50147755 T1 9 0

50147872 T1 2 0

50148035 T1 3 0

50149063 T1 2 0

50149438 T1 14 0

50149495 T2 8 0

50149741 T1 26 0

50150265 T1 16 0

50150268 T1 28 0

50150374 T1 18 0

50150632 T1 12 0

50150988 T1 10 0

50151076 T1 37 0

50151463 T2 8 0

50151606 T1 1 0

50151722 T1 12 0

50152222 T1 4 0

50152225 T2 4 0

50152231 T1 9 0

50152303 T1 14 0

50152314 T1 7 0

50152469 T1 9 0

50152757 T1 12 0

50152758 T1 17 0

50152883 T1 5 0

50153138 T1 50 0

50153227 T1 42 0

50153369 T2 7 0

50153476 T1 18 0

50153739 T1 9 0

50153960 T1 5 0

50153963 T1 8 0

50154076 T1 17 0

50154249 T2 27 0

50154250 T3 25 1

50154710 T3 15 0

50155591 T1 26 0

50155721 T1 9 0

50155723 T1 18 0

50156001 T1 4 0

50156599 T2 1 0

50156823 T1 1 0

50157259 T1 11 0

50157368 T3 6 0

50157485 T1 13 0

50157511 T1 19 0

50157606 T3 13 0

50157725 T1 44 0

50158293 T1 8 0

50158797 T1 7 0

50158823 T1 3 0

50158957 T1 12 0

50159054 T1 25 0

50159203 T1 11 0

50159676 T1 2 0

50159848 T1 17 0

50159851 T1 23 0

50160209 T1 14 0

50160419 T2 7 0

50160688 T1 5 0

50161035 T1 39 0

50161132 T1 5 0

50161422 T1 29 0

50161931 T1 10 0

50162480 T1 8 0

50162529 T1 26 0

50162557 T1 29 0

50162565 T1 21 0

50162566 T1 17 0

50162567 T2 14 0

50163100 T1 9 0

50163105 T1 2 0

50163606 T3 1 1

50163609 T3 38 0

50163660 T1 20 0

50164549 T1 16 0

50167614 T1 6 0

50170026 T1 5 0

50170290 T1 22 0

50171873 T1 38 0

50171923 T2 1 0

50171928 T1 5 0

50172055 T2 10 0

50172060 T1 10 0

50176438 T1 40 0

50176981 T1 33 0

50177042 T1 15 0

50180435 T1 5 0

50183383 T1 24 0

50183738 T2 35 0

50184793 T1 35 0

50191915 T1 1 0

50197314 T1 2 0

50231318 T1 7 0

50232574 T1 24 0

51011195 T1 15 0

52106096 T1 3 0

42502935 T1 2 0

42503542 T1 6 1

42503904 T1 11 0

42504553 T1 17 0

42504895 T1 16 0

42505356 T1 13 0

42505443 T1 8 0

42505686 T1 12 0

42505749 T1 5 0

42505947 T3 4 0

42507076 T1 3 0

42507508 T1 3 0

42507818 T1 8 0

42508535 T1 7 0

54009781 T2 6 0

54010417 T1 29 0

54010476 T1 2 0

54010614 T1 4 0

54011563 T3 5 0

54011712 T1 6 0

54012033 T1 1 0

54013080 T1 10 0

54013083 T1 6 0

54013338 T1 9 0

54013479 T1 12 0

54014240 T2 2 0

54014574 T1 11 0

54015049 T1 2 0

54015589 T1 3 0

54015744 T1 11 0

54015752 T1 9 0

54016391 T1 2 0

54016408 T1 11 0

54016659 T2 10 0

54016707 T1 11 0

54016873 T1 16 0

54017939 T1 3 0

54018596 T1 11 0

54019538 T1 2 0

54020377 T2 4 0

54020510 T1 13 0

54020634 T2 4 0

54021376 T1 7 0

54021632 T2 18 0

54023288 T1 1 0

55188528 T1 3 0

55191355 T1 15 0

55192909 T3 4 0

55193275 T1 5 0

55193642 T1 25 0

55193757 T1 24 0

55194683 T1 7 0

55196556 T1 19 0

55196560 T1 7 0

55196777 T1 2 0

55197222 T1 3 0

55198148 T1 3 0

55198152 T1 4 0

55199089 T1 12 0

55199461 T1 46 0

55200495 T1 4 0

55200505 T1 22 0

55201037 T1 9 0

55201831 T1 7 0

55202666 T1 5 0

55202980 T1 3 0

55204436 T1 14 0

55204441 T1 5 0

55204448 T1 12 0

55204840 T1 5 0

55205200 T4 12 5

55205915 T2 10 0

55206047 T1 3 0

55206887 T1 15 0

55207074 T1 25 0

55207675 T1 6 0

55207682 T1 11 0

55207822 T1 9 0

55208613 T1 4 1

55208657 T1 27 0

55209192 T1 19 0

55209398 T1 40 0

55210634 T1 32 0

55215321 T1 12 0

55216441 T1 55 0

55218613 T1 12 0

55218625 T1 9 0

55219756 T2 37 0

55220017 T2 15 0

55220035 T2 5 0

55220614 T1 29 0

55221875 T1 1 0

55221881 T2 21 0

55221973 T1 59 0

55222364 T3 9 0

55222623 T2 24 0

55222841 T1 7 0

55223126 T1 2 0

55224050 T1 4 0

55224915 T2 10 0

55226039 T1 16 0

55227294 T1 21 0

55227296 T1 6 0

55227424 T3 12 0

55227851 T1 4 0

55228036 T1 16 0

55228050 T1 33 0

55228432 T1 9 0

55228481 T3 31 0

55229021 T1 38 0

55229408 T1 2 0

55229641 T2 4 0

55229651 T2 14 0

55229670 T1 14 0

55229915 T1 8 0

55230356 T1 34 0

55230365 T3 22 0

55230773 T1 32 0

55230928 T1 18 0

55231094 T1 7 0

55231098 T3 13 5

55231609 T1 12 0

55232437 T1 25 0

55232507 T3 4 0

55233292 T1 47 0

55233296 T1 39 0

55233513 T3 3 0

55233518 T1 7 0

55233694 T1 6 0

55234781 T1 3 0

55234785 T1 19 0

55235618 T1 7 0

55235627 T1 7 0

55235833 T1 10 0

55236767 T1 9 0

55236794 T2 14 0

55237062 T2 13 0

55238186 T2 36 0

55238629 T1 1 0

55242695 T1 5 0

55242774 T1 3 0

55242780 T1 3 0

55243063 T1 10 1

55243404 T1 32 0

55243559 T1 13 0

55244688 T1 13 0

55244692 T1 5 0

55245163 T1 8 0

55245612 T1 32 0

55245637 T1 27 0

55246149 T1 8 0

55246226 T2 3 0

55246423 T1 15 0

55247494 T1 15 0

55247563 T1 7 0

55247778 T3 10 0

55247967 T1 30 0

55249280 T1 12 0

55250805 T1 6 0

55250807 T1 5 0

55251184 T2 8 0

55251304 T1 11 0

55251305 T1 66 0

55251487 T1 6 0

56500383 T1 10 0

56500551 T1 8 0

56501593 T1 17 0

56503755 T1 5 0

56505727 T1 5 0

56507054 T1 7 0

56508504 T1 12 0

56508885 T3 28 0

56511307 T1 4 0

56515269 T1 8 0

56515552 T1 32 0

56517657 T1 4 0

56517817 T1 26 0

56518595 T1 1 0

56519208 T1 17 0

56519700 T1 12 0

56521165 T1 7 0

56521225 T1 2 0

56522757 T1 18 0

56523325 T1 25 0

56524027 T1 9 0

56525023 T1 14 0

56525806 T1 34 0

56526389 T1 31 0

56527046 T3 8 0

56528274 T1 13 0

56531513 T1 2 0

56531848 T1 5 0

56532303 T1 8 0

56535016 T1 8 0

56535061 T1 4 0

56535068 T1 21 0

56535513 T1 9 0

56539644 T1 37 7

56539653 T1 9 0

56540125 T1 9 0

56541370 T1 24 1

56542090 T1 40 0

56542097 T1 28 0

56542114 T1 43 0

56542147 T3 61 0

56542153 T1 42 0

56544892 T1 2 0

56547440 T1 23 0

56547760 T1 33 0

56550689 T1 13 0

56553994 T1 21 0

56554272 T1 13 0

56555665 T1 11 0

56556060 T1 14 0

56557869 T1 9 0

56557949 T1 13 0

56559162 T3 2 1

56561721 T2 23 0

56562956 T1 9 0

56562986 T1 4 0

56563221 T1 13 0

56564504 T1 6 0

56564516 T1 19 0

56565328 T1 1 0

56570523 T1 14 0

56571512 T2 6 0

56572029 T1 15 0

56572534 T1 9 0

56573040 T1 9 0

56573537 T1 13 0

56575373 T1 4 0

56575581 T4 4 2

56579471 T1 5 0

56579500 T1 8 0

56587192 T1 9 0

56587225 T1 20 0

56592517 T1 19 0

56594117 T1 15 0

56595061 T1 9 0

56595256 T2 35 0

56595265 T2 6 0

56596913 T1 13 0

56596926 T1 20 0

56599830 T1 4 0

56600120 T1 6 0

56600835 T1 16 0

56600894 T1 42 2

56603971 T1 26 0

56604312 T1 12 0

56605111 T1 25 0

56605474 T1 19 0

56605921 T1 11 0

56606516 T1 4 0

56607057 T1 24 0

56607065 T1 13 0

56608320 T1 13 0

56608366 T3 17 2

56608370 T1 4 0

56608440 T1 19 0

56609745 T1 4 0

56610539 T1 3 0

56611345 T1 8 0

56611930 T1 25 1

56612958 T1 15 0

56613833 T1 6 0

56613961 T3 51 0

56614951 T1 10 0

56617179 T1 16 0

56617473 T1 14 0

56617485 T3 1 0

56618316 T1 16 0

56618373 T1 2 2

56618584 T1 9 0

56618931 T1 17 0

56619142 T1 8 0

56619275 T1 21 0

56619728 T1 7 0

56619854 T2 26 3

56620165 T1 43 0

56621808 T1 26 0

56622470 T3 17 0

56622684 T1 4 0

56624142 T1 9 0

56624763 T1 10 0

56624989 T1 11 0

56625327 T1 1 0

56625348 T3 1 1

56626324 T1 13 0

56626476 T1 6 0

56626643 T1 14 0

56626953 T4 27 5

56626971 T2 9 3

56628289 T1 11 0

56628804 T1 34 1

57714553 T1 10 0

57714642 T1 14 0

57716562 T1 35 0

57718486 T1 12 0

57719026 T1 2 0

57719124 T1 12 0

57720234 T1 8 0

57721673 T1 2 0

57721823 T1 32 0

57722188 T1 8 0

57722219 T2 35 1

57723141 T1 19 0

57723155 T1 12 0

57723769 T1 26 0

57724755 T3 24 0

57724933 T1 30 0

57727383 T1 9 1

57728370 T1 9 0

57728377 T1 17 0

57730408 T3 21 2

57735004 T1 5 0

57736089 T1 45 0

57738178 T1 8 0

57738183 T1 15 0

57739163 T1 15 0

57758908 T1 7 0

57761655 T1 29 0

57761686 T1 28 0

57764578 T1 3 0

57773702 T1 4 0

57776354 T1 7 0

57777129 T1 19 0

57777559 T1 32 0

57778461 T1 26 0

57778487 T1 28 0

57781738 T1 16 0

57784242 T1 4 0

57784280 T1 4 0

57787572 T1 19 0

57787609 T3 11 0

57788790 T1 4 0

57789469 T1 4 0

57793712 T1 6 0

57793726 T1 6 0

57794591 T1 45 0

57796142 T1 41 0

57798106 T1 25 0

57798899 T1 2 0

57800812 T1 32 0

57800821 T2 27 0

57801947 T1 19 0

57801966 T1 17 0

57803792 T3 25 0

57803837 T1 5 0

57803897 T1 22 0

57804174 T1 11 0

57804529 T1 11 0

57804540 T1 3 0

57807679 T2 10 0

57812561 T2 25 1

57817792 T1 5 0

57817818 T3 3 0

57818557 T1 1 0

57819642 T1 5 0

57820994 T1 5 0

57821001 T1 24 1

57823321 T1 6 0

57827060 T1 2 0

57827672 T1 5 0

57827857 T2 3 0

57828586 T3 1 0

57831013 T3 5 0

57831884 T1 2 0

57831958 T2 7 0

57833390 T1 32 0

57834692 T1 42 0

57836818 T1 19 0

57840362 T1 9 0

57840378 T1 11 0

57840406 T2 49 0

57840451 T1 5 0

57840464 T1 4 0

57841625 T1 25 0

57842795 T1 22 0

57844628 T3 18 1

57846262 T1 9 0

57847072 T2 2 0

57847083 T1 12 0

57848713 T1 38 0

57850240 T1 9 0

57851172 T3 1 0

57852582 T1 24 0

57853127 T1 3 0

57854413 T1 7 0

57855250 T1 6 2

57855266 T2 45 0

57856516 T1 7 0

57857128 T1 23 0

57858536 T1 10 0

57859828 T1 9 0

57861981 T2 4 0

57863159 T2 2 0

57864598 T1 10 0

57865913 T1 2 0

57870792 T1 5 0

57870879 T1 2 0

57872383 T1 49 0

57872994 T1 17 0

57874671 T1 6 0

57874689 T1 6 0

57876277 T1 13 0

57876290 T1 16 0

57876335 T1 16 0

57876945 T1 14 0

59170903 T1 2 0

59173960 T2 6 0

59184726 T2 16 0

59184901 T1 10 0

59186948 T1 6 0

59187844 T1 9 0

59187851 T1 3 0

59187876 T2 22 0

59191689 T1 21 0

59191942 T1 11 0

59193707 T1 4 0

59194367 T1 16 0

59195285 T1 14 0

59196017 T1 10 0

59197667 T1 16 0

59199419 T1 9 0

59199449 T1 12 0

59199472 T1 12 0

59199483 T2 25 1

59199491 T1 2 0

59199502 T1 9 0

59201029 T1 6 0

59202233 T1 12 0

59202238 T1 24 0

59202491 T1 13 1

59202507 T1 10 0

59204000 T1 8 0

59205170 T1 1 0

59205451 T1 35 0

59205780 T1 13 0

59205787 T1 26 0

59209338 T1 13 0

59210523 T1 38 0

59211138 T1 2 0

59211246 T1 12 0

59211334 T1 8 0

59211399 T1 1 0

59211760 T3 7 0

59211901 T1 15 0

59212199 T1 3 0

59214791 T3 10 0

59214866 T1 16 0

59215851 T1 15 1

59218880 T2 8 0

59220408 T1 10 0

59220728 T1 11 0

59220842 T1 22 0

59221779 T1 17 0

59223272 T3 14 1

59223749 T1 12 0

59225624 T1 5 0

59226363 T2 4 0

59226379 T1 20 0

59226387 T1 12 0

59226399 T2 37 0

59228012 T1 22 0

59228093 T2 27 3

59228292 T1 6 0

59228461 T4 19 0

59229691 T3 7 0

59230723 T2 4 0

59230733 T1 10 0

59231499 T1 15 0

59231522 T1 7 3

59231527 T1 34 0

59233115 T1 4 0

59234330 T3 12 0

59234673 T1 31 0

59237726 T1 38 0

59239220 T3 2 0

59239801 T1 33 0

59240630 T1 9 0

59241220 T2 7 0

59242045 T1 12 0

59242065 T1 2 0

59242131 T1 7 0

59242962 T1 10 0

59243476 T1 7 0

59246799 T1 8 0

59248323 T1 10 0

59251373 T1 17 0

59254004 T1 21 0

59255563 T1 17 0

59256316 T1 16 0

59257114 T1 7 0

59257445 T1 24 0

59259666 T1 15 0

59259670 T1 10 0

59259799 T1 6 0

59259817 T1 6 0

59259832 T1 17 0

59261411 T1 6 2

59262897 T2 11 0

59262945 T2 7 0

59263018 T1 8 0

59263756 T1 9 0

59264832 T2 6 1

59267749 T1 11 0

59269355 T1 9 0

59269363 T1 19 0

59269374 T1 18 0

59269381 T1 27 0

59269411 T1 41 0

59269650 T2 7 0

59269680 T1 8 0

59269683 T1 4 0

59271251 T1 1 0

59271269 T2 23 0

59272762 T1 5 0

59273078 T1 13 1

59275225 T1 21 0

59278677 T1 20 0

59278689 T2 50 0

59278837 T1 16 0

59281391 T1 10 3

59283760 T1 1 0

59290265 T1 1 0

59294985 T1 12 0

59297992 T2 7 0

59298037 T3 1 0

59300238 T1 13 0

59301210 T1 30 0

59304938 T1 8 0

59306664 T1 4 0

59311373 T2 8 0

59311672 T1 7 0

59315030 T1 30 0

59319812 T1 28 0

59323455 T1 24 0

59332248 T1 17 0

59335083 T1 7 1

59335122 T1 6 0

59335134 T1 3 0

59336178 T1 25 0

59336189 T3 37 1

59339623 T1 8 0

59340104 T1 31 0

59341585 T1 30 0

59341596 T2 22 0

59341629 T1 3 0

59341646 T1 4 0

59342354 T3 18 2

59343751 T1 4 0

60431719 T1 34 0

60431744 T2 38 0

60432400 T1 17 0

60437145 T1 14 0

60440226 T1 6 0

60440744 T1 9 0

60441827 T1 45 0

60442003 T1 7 0

60449094 T1 10 0

60449539 T2 46 3

60449564 T2 15 2

60453076 T1 7 0

60453095 T1 19 0

60460675 T1 10 0

60462133 T2 1 0

60462898 T1 7 0

60464940 T1 3 0

60464952 T1 47 0

60465234 T1 20 0

60465247 T1 15 0

60465376 T3 13 0

60472539 T1 5 0

60473830 T1 9 0

60474736 T1 30 0

60475920 T1 5 0

60476692 T2 18 0

60479902 T1 30 0

60480363 T1 15 0

60480370 T1 33 0

60482613 T1 17 0

60484288 T1 47 0

60484608 T1 12 0

60487597 T1 4 0

60491394 T2 21 0

60498090 T1 8 0

60499155 T1 47 0

60500783 T1 8 0

60500793 T1 31 0

60504813 T1 7 1

60509407 T1 2 0

60511754 T1 31 0

60517154 T1 16 0

60517197 T1 12 0

60517220 T1 26 1

60517245 T2 34 0

60530508 T3 11 0

60534598 T1 23 0

60537042 T2 1 1

60537418 T1 33 0

60548683 T1 14 0

60549394 T1 22 0

60550110 T2 2 0

60550440 T3 23 0

60550490 T1 16 0

60551006 T1 23 0

60551292 T1 6 0

60552229 T2 33 0

60552232 T1 9 0

60552234 T1 4 0

60552236 T1 20 8

60552308 T1 20 0

60552330 T1 22 0

60552343 T1 32 0

60552632 T1 33 0

60552944 T1 25 0

60554244 T1 36 0

60554633 T1 32 0

60555276 T1 7 2

60555628 T1 25 0

60555734 T1 25 0

60557088 T1 18 0

60557343 T1 5 0

60557344 T1 5 0

60557349 T1 6 0

60557354 T2 4 0

60557588 T1 27 0

60557624 T1 8 0

60557811 T3 2 0

60558652 T1 9 0

60558736 T1 34 0

60558744 T1 15 0

60559244 T1 3 0

60560477 T1 8 0

60560498 T1 1 0

60560575 T1 28 0

60560772 T1 12 0

60561114 T1 2 0

60561399 T1 8 4

60561447 T1 3 0

60561601 T1 21 0

60561723 T2 30 1

60561736 T1 10 0

60561831 T1 7 0

60562182 T1 14 0

60562655 T1 6 0

60562661 T1 28 0

60562664 T1 13 0

60562675 T3 16 0

60562680 T1 3 0

60562683 T1 25 0

60562821 T1 13 0

60562909 T1 11 2

60563065 T1 11 0

60563176 T1 8 0

60563207 T1 16 0

60563210 T1 19 0

60563307 T1 15 0

60563328 T1 27 0

60563329 T1 21 0

60563358 T1 17 0

60563372 T1 14 0

60563681 T1 9 0

60563695 T2 31 0

60565488 T1 17 0

60565522 T2 11 0

60565735 T1 41 0

60566581 T1 17 0

60567132 T1 2 0

60567137 T1 4 0

60567234 T1 38 0

60568422 T1 16 0

60570365 T1 18 0

60571095 T1 1 0

60571111 T1 2 0

60571801 T1 65 0

60571913 T1 28 0

60572135 T1 1 0

60573912 T1 12 0

60575184 T1 7 0

60575204 T1 19 0

60575213 T1 6 0

60575523 T1 28 0

60575949 T1 3 0

60577098 T1 2 0

60577394 T1 7 0

60578159 T1 19 0

60578505 T3 5 0

60579870 T1 2 0

60580186 T1 7 0

60581687 T1 13 0

60582211 T3 19 0

60582489 T1 19 0

60582886 T3 10 0

60582900 T1 15 0

60582906 T1 7 0

60583363 T1 8 0

60583417 T3 8 0

60583726 T2 4 0

60583762 T2 17 0

60583786 T1 6 0

60583795 T1 14 0

60583852 T2 8 0

60584384 T1 5 0

60584405 T1 1 0

60584437 T1 2 0

60584446 T1 25 0

60585053 T1 5 0

60585060 T1 26 0

60585675 T1 32 0

60585953 T1 12 0

60586302 T3 25 10

60586305 T1 10 0

60586308 T1 17 0

60586669 T1 81 0

60586678 T1 87 0

60586702 T1 7 0

60586713 T1 6 0

60587162 T1 8 0

60587170 T1 13 0

60587187 T1 28 0

60587212 T1 22 0

60587217 T1 33 0

60587389 T3 50 0

60587474 T3 37 0

60587477 T3 19 0

60587760 T1 20 0

60587868 T1 14 0

60588571 T1 8 0

60588702 T3 10 0

60589140 T3 9 0

60589200 T1 18 0

60589287 T1 15 0

60589373 T1 52 0

60589530 T1 3 0

60589860 T1 11 0

60590504 T1 40 0

60590906 T1 37 7

60591396 T1 7 0

60591628 T1 25 0

60591636 T1 14 0

60592134 T2 10 0

60592149 T1 17 0

60592259 T1 32 0

60592418 T2 12 1

60592544 T1 2 0

60592645 T1 14 0

60592685 T1 2 0

60592703 T1 2 0

60592771 T1 30 0

60592775 T1 53 0

60592829 T1 58 0

60592832 T2 43 0

60592866 T1 8 0

60593104 T2 30 0

60593107 T1 30 0

60593111 T2 6 0

60593281 T1 63 0

60593291 T1 9 0

60593321 T1 8 0

60594025 T1 14 0

60594076 T1 36 0

60594517 T1 26 0

60595154 T1 28 0

60595644 T1 9 0

60595834 T1 14 0

60595874 T4 28 1

60596039 T1 20 0

60596051 T1 13 0

60596422 T1 35 0

60596524 T1 29 0

60596528 T1 5 0

60596538 T1 5 0

60596600 T1 15 0

60597117 T1 18 0

60597150 T1 31 8

60597317 T1 3 0

60597425 T2 8 0

60597488 T2 35 0

60598079 T1 16 0

60598091 T1 10 0

60598627 T1 10 0

60598961 T1 9 0

60599012 T2 9 0

60599038 T1 10 0

60599052 T3 4 0

60599374 T2 21 0

60599903 T1 10 0

60599912 T2 39 0

60599953 T1 20 0

60600361 T1 12 0

60600373 T1 10 0

60600431 T1 12 1

60600902 T1 5 0

60600917 T1 5 0

60601165 T1 19 0

60601648 T2 11 0

60602012 T1 9 3

60613628 T1 13 0

60614182 T1 5 0

60614983 T2 10 0

60615432 T1 3 0

60615498 T2 18 0

60615889 T1 6 0

60615923 T1 10 0

60615927 T3 22 0

60616770 T1 24 0

60616988 T1 20 0

60617701 T1 35 0

60618056 T1 4 0

60618125 T1 23 0

60619355 T1 18 0

60619716 T2 28 0

60619760 T1 15 0

60620039 T1 47 0

60620316 T3 17 7

60620318 T1 10 0

60620647 T1 11 0

60621858 T1 37 0

60622295 T1 10 0

62054123 T1 23 0

62054673 T2 10 0

62057941 T1 14 0

63143128 T1 13 0

63144539 T3 24 1

63145308 T1 22 0

63146937 T1 13 0

63149298 T1 2 0

63149604 T3 16 0

63150427 T1 28 0

63155447 T1 7 0

63157172 T3 10 0

63160971 T1 25 0

63161527 T1 1 0

63163979 T2 8 0

63164090 T1 1 0

63166578 T1 9 0

63166611 T1 23 0

63166626 T1 12 0

63168963 T3 23 10

63169237 T1 15 0

63170657 T1 9 0

63170961 T1 4 0

63172088 T1 5 0

63172751 T1 6 0

63175878 T3 11 0

63175903 T1 2 0

63177035 T1 12 0

63179302 T3 37 0

63183280 T1 14 0

63183290 T1 5 0

63183298 T1 13 0

63184422 T1 9 0

63187090 T1 8 0

63187483 T1 17 0

63188124 T1 9 0

63189163 T1 31 0

63190677 T2 21 0

63190796 T1 10 0

63196106 T1 3 0

63198340 T2 19 0

63198395 T1 10 0

63199331 T1 18 0

63200496 T1 3 0

63202455 T1 20 0

63203556 T1 10 0

63204332 T2 21 0

63204825 T1 2 0

63204841 T2 12 0

63204855 T1 4 0

63205574 T1 4 0

63206328 T1 28 0

63206353 T1 13 0

63209267 T2 6 0

63210086 T1 11 0

63210101 T1 4 0

63211465 T1 12 0

63211497 T1 13 0

63211511 T1 1 0

63211546 T1 1 0

63211991 T1 4 0

63212002 T1 7 0

63212406 T2 25 0

63214819 T1 12 0

63214838 T1 36 0

63215718 T1 25 0

63216100 T1 39 0

63217062 T1 7 0

63217491 T2 10 1

63217863 T3 1 0

63222428 T1 24 0

63222437 T1 18 0

63223793 T1 6 0

63224857 T1 9 0

63228112 T1 13 0

63230967 T1 13 0

63232896 T1 13 0

63233789 T1 30 0

63233929 T1 47 0

63235368 T3 25 0

63238446 T1 3 0

63240119 T1 7 0

63240425 T1 10 0

63240690 T1 9 0

63241154 T1 9 0

63241637 T1 3 0

63241940 T2 1 0

63241955 T1 1 0

63242313 T1 9 0

63242493 T1 6 0

63244233 T1 17 0

63245497 T1 7 0

63245520 T1 4 0

63249444 T1 5 0

63249460 T1 15 0

63253254 T2 29 0

63254244 T2 3 0

63256532 T2 10 0

63256537 T1 15 0

63256809 T1 20 0

63257473 T1 26 0

63260761 T1 30 0

63260951 T1 37 0

63263862 T1 24 0

63264340 T1 9 0

63264662 T3 2 0

63266196 T3 23 0

63269726 T1 20 0

63269758 T3 23 0

63269797 T1 28 0

63271540 T1 19 0

63273731 T3 29 0

63275769 T2 36 0

63275799 T1 28 0

63275821 T3 36 0

63276339 T2 19 0

63277400 T1 64 0

63277416 T1 26 0

63278041 T2 6 0

63279737 T1 7 0

63280119 T1 4 0

63280468 T1 12 0

63280747 T1 16 0

63281038 T1 15 0

63281136 T1 3 0

63283254 T1 20 0

63284575 T1 5 0

63285208 T1 4 0

63286608 T1 8 0

63287082 T3 3 2

63287641 T2 12 0

63289010 T1 12 0

63289022 T1 11 0

63291138 T1 9 0

63293351 T1 12 0

63295359 T1 22 0

63296285 T1 12 0

63296324 T1 6 0

63296332 T3 11 7

63298607 T1 6 0

63301748 T1 10 0

63302494 T1 11 0

63303037 T1 4 0

63304976 T1 26 4

63305395 T1 13 1

63305694 T1 26 0

63305874 T1 11 0

63306917 T1 6 0

63307542 T1 12 0

63308286 T3 13 0

63309777 T1 10 0

63309836 T1 14 3

63309996 T1 9 0

63310233 T1 28 0

63311654 T1 29 0

63312285 T1 17 0

63315480 T1 14 0

63315656 T1 34 0

63315963 T2 27 0

63316700 T1 3 0

63319136 T1 14 0

63319141 T2 35 0

63319607 T1 11 0

63319975 T1 11 0

63320392 T3 4 0

63321248 T1 9 0

63321554 T1 18 1

63324447 T1 30 0

63325772 T1 3 0

63325896 T1 12 0

63327379 T3 15 1

63327381 T2 16 1

63327928 T1 29 0

63328362 T3 1 1

63328995 T1 9 0

63329187 T1 20 0

63329667 T1 23 0

63330468 T1 34 0

63330808 T1 30 0

63331463 T1 6 0

63331831 T1 3 0

63332018 T1 13 0

63333430 T3 2 0

63334081 T1 8 0

63334611 T1 7 0

63334613 T1 9 0

63335175 T1 37 2

63335179 T1 17 0

63335492 T1 19 0

63338453 T1 11 0

63338455 T2 30 0

63339084 T1 8 0

63343652 T1 3 0

63345690 T1 17 0

63346813 T3 8 0

63348308 T1 19 0

63349623 T3 2 1

63350046 T1 21 0

63351079 T1 15 0

63352122 T1 19 0

63353378 T1 4 0

63353476 T2 23 0

63353484 T1 6 0

63353486 T1 16 0

63353736 T1 3 0

63354007 T1 7 0

63354365 T1 1 0

63354465 T1 27 0

63354487 T2 43 0

63355016 T1 3 0

63355110 T1 4 0

63355114 T1 19 0

63355176 T1 4 1

63355673 T2 10 0

63355867 T2 15 1

63356020 T1 4 0

63356025 T1 7 0

63356029 T1 19 0

63356033 T1 1 0

63356065 T1 18 0

63356664 T1 11 0

63356949 T2 18 0

63357148 T1 11 0

63357284 T3 10 1

63357330 T2 30 0

63357601 T1 2 0

63357759 T1 4 1

63357875 T2 15 0

63358047 T1 13 0

63358064 T1 7 0

63358432 T1 5 0

63358987 T1 4 0

63359696 T1 5 0

63359700 T1 30 0

63359995 T1 17 0

63361142 T1 4 0

63361751 T1 2 0

63362228 T2 16 0

63362546 T1 5 0

63362617 T1 16 0

63363088 T1 16 0

63363097 T2 24 0

63363105 T1 30 0

63363555 T1 20 0

63365158 T1 2 0

63365278 T2 2 0

63365710 T1 21 0

63365884 T1 9 1

63366264 T1 33 0

63366514 T1 24 0

63366860 T1 15 0

63367644 T1 7 0

63367874 T1 25 0

63368057 T1 3 0

63368352 T1 14 0

63368703 T1 6 0

63368889 T1 12 1

63368975 T1 6 0

63369327 T1 11 0

63369574 T1 28 0

63369575 T1 13 0

63369789 T1 3 0

63369790 T1 7 0

63369946 T1 19 0

63369950 T1 16 0

63370322 T1 4 0

63370325 T1 10 0

63370329 T1 1 0

63371108 T2 25 0

63371814 T2 37 0

63371890 T1 12 0

63371892 T1 6 0

63371894 T1 28 0

63372184 T1 7 0

63372227 T1 9 0

63372311 T1 29 0

63372313 T1 38 0

63372426 T2 25 0

63372429 T1 26 0

63372460 T2 1 0

63372461 T1 13 0

63372463 T1 11 0

63372853 T1 25 0

63373140 T1 14 0

63373350 T1 1 0

63373442 T1 23 0

63373722 T1 21 0

63374184 T1 17 0

63374879 T1 9 0

63375015 T1 14 0

63375223 T3 4 0

63375312 T1 16 0

63375314 T1 9 0

63375855 T3 22 0

63376418 T1 12 0

63376420 T1 26 0

63376560 T1 12 0

63377056 T1 6 0

63377060 T1 8 0

63377063 T1 11 0

63377115 T1 8 0

63377146 T1 7 0

63377225 T3 12 6

63377570 T1 15 0

63377955 T1 47 0

63377957 T3 21 2

63378561 T1 7 0

63378563 T1 2 0

63378565 T1 11 0

63378619 T1 9 0

63378621 T1 17 0

63378778 T1 5 0

63378862 T1 24 0

63379097 T1 5 0

63379621 T1 15 0

63379814 T1 24 0

63379997 T1 19 0

63380074 T1 8 0

63381249 T1 11 0

63381586 T1 34 0

63381928 T1 7 0

63381929 T3 6 0

63382358 T1 22 0

63382364 T1 62 0

63382745 T2 11 0

63383834 T1 10 0

63385775 T2 1 1

63386151 T1 19 0

63386668 T1 8 0

63387097 T1 7 0

63387734 T1 12 0

63388568 T1 14 0

63389027 T1 3 0

63389172 T1 19 0

63389842 T1 4 0

63389846 T1 8 0

63390787 T1 6 0

63390800 T1 10 0

63390819 T2 11 0

63391205 T1 10 0

63391616 T1 1 0

63392018 T1 24 0

63393580 T1 7 0

63405855 T1 4 0

63405865 T1 27 0

63406017 T1 14 0

63406024 T1 25 0

63406977 T1 3 0

63407052 T1 12 0

63407144 T1 28 0

63408014 T1 18 0

63408131 T1 3 0

63408586 T1 8 0

63408768 T2 33 0

63408918 T1 30 0

63409168 T1 50 0

63409261 T1 5 0

63409294 T2 23 0

63409484 T1 20 0

63409520 T1 19 0

63409914 T3 5 0

63410425 T2 28 0

63410861 T1 20 0

63410906 T1 22 0

63411180 T1 31 0

63411247 T1 27 0

63411301 T1 12 0

63411744 T1 27 0

63412147 T2 37 0

63412612 T1 1 0

63413267 T1 7 0

63413342 T1 16 0

63413369 T1 1 0

63413415 T3 11 0

63413467 T1 18 0

63414198 T1 5 3

63414506 T1 14 0

63414581 T1 14 0

63414778 T1 3 0

63415178 T1 18 0

63415183 T1 2 0

63415909 T1 7 0

63416077 T1 3 0

63416832 T1 10 0

63416848 T2 9 1

63416852 T1 5 0

63416855 T3 10 0

63416871 T1 2 0

63416958 T1 15 0

63417143 T1 3 0

63417145 T1 22 0

63417521 T2 45 0

64500522 T1 23 0

64501036 T1 6 0

64501530 T1 8 0

64502067 T1 38 0

64502152 T1 16 0

64502587 T1 9 0

64502985 T1 18 0

64503002 T1 21 0

64503443 T1 10 0

64503494 T1 10 0

64503698 T1 28 0

64504087 T2 22 0

64504208 T2 15 0

64504249 T2 2 0

64504657 T1 12 0

64505467 T1 7 0

64505720 T1 9 0

64506692 T1 32 0

64507538 T1 13 0

64507688 T2 25 0

64508129 T1 22 0

64508375 T1 18 0

64508388 T1 26 0

64509145 T1 53 0

64509844 T1 4 0

64509915 T1 20 0

64509921 T1 13 0

64509924 T1 37 0

64511163 T1 13 0

64511202 T1 6 6

64511273 T1 5 0

64511625 T1 50 0

64511650 T1 31 0

64511656 T1 33 0

64511666 T1 47 0

64511681 T1 62 0

64511684 T1 53 0

64511692 T1 59 0

64511699 T1 3 0

64511742 T1 42 0

64511747 T1 51 0

64512242 T1 65 0

64512681 T1 5 0

64512760 T1 8 0

64512912 T2 7 0

64512922 T1 17 0

64513672 T2 5 0

64513940 T1 6 0

64514173 T3 9 0

64514578 T1 4 0

64514763 T1 12 0

64514771 T2 7 0

64514787 T1 16 0

64514800 T1 22 0

64514829 T1 6 0

64515518 T1 20 1

64516242 T1 29 5

64516382 T1 1 0

64516898 T1 17 0

64517508 T3 3 0

64517995 T1 29 0

64518018 T1 30 0

64518388 T1 37 1

64518440 T1 12 0

64518463 T2 10 0

64518470 T1 25 0

64518516 T1 1 0

64518566 T1 18 0

64518754 T1 1 0

64520429 T1 32 0

64520436 T1 10 0

64520518 T3 15 0

64520628 T1 13 0

64521228 T1 2 0

64522178 T2 7 0

64522832 T1 7 0

64523412 T1 3 0

64523426 T1 1 0

64523590 T3 8 0

64523693 T1 9 0

64523735 T1 19 0

64523747 T1 12 0

64523960 T1 3 0

64524032 T1 13 0

64524258 T2 16 0

64524313 T1 10 0

64524865 T2 22 1

64525282 T2 10 1

64525702 T1 45 0

64526443 T1 5 0

64526449 T1 4 0

64526836 T3 8 0

64526940 T1 48 0

64526975 T1 19 0

64527328 T1 11 0

64527399 T1 30 0

64528152 T1 8 0

64528236 T1 16 0

64528544 T1 31 0

64528549 T1 23 0

64528552 T1 5 0

64529296 T1 18 0

64530056 T1 4 0

64531126 T1 8 0

64531156 T1 3 0

64531157 T2 13 0

64531158 T1 12 0

64531160 T2 7 0

64531248 T4 10 3

64531300 T1 5 0

64531331 T1 11 0

64531341 T1 8 0

64531346 T1 21 0

64531465 T1 8 0

64531670 T1 17 0

64532557 T2 7 0

64533283 T3 6 0

64536125 T1 8 0

64536127 T1 9 0

64536132 T1 3 0

64536133 T1 4 0

64536228 T1 6 0

64536372 T3 5 0

64536446 T2 7 0

64536524 T1 7 0

64536980 T1 15 0

64536985 T1 9 0

64536987 T1 3 0

64536990 T1 16 0

64537086 T1 5 0

64537087 T1 24 0

64537088 T1 31 0

64537734 T1 12 0

64537990 T1 7 0

64538095 T1 8 0

64538184 T1 10 0

64538960 T1 8 0

64539237 T1 21 0

64539578 T1 5 0

64539754 T1 7 0

64539943 T1 51 0

64539969 T1 39 0

64540191 T1 9 0

64540681 T1 41 0

64540687 T3 27 0

64541103 T1 48 0

64541108 T2 25 0

64541317 T3 27 0

64541456 T1 4 0

64541552 T3 7 2

64541618 T1 32 0

64541914 T1 15 0

64542596 T1 38 1

64543746 T1 10 0

64544144 T1 18 0

64544150 T1 7 0

64544631 T2 6 0

64544786 T1 3 0

64544870 T1 26 0

64545345 T1 22 0

64545596 T1 11 0

64545745 T1 20 0

64545923 T1 22 0

64545926 T1 14 0

64545929 T1 19 0

64546359 T1 25 0

64546821 T1 12 0

64546909 T1 15 0

64547140 T1 5 0

64547141 T1 40 0

64547537 T1 24 0

64547565 T2 8 0

64547584 T1 7 0

64547605 T1 15 0

64547629 T1 7 0

64547672 T1 6 0

64547764 T1 18 0

64547846 T1 20 0

64547965 T2 15 1

64548033 T1 2 0

64548034 T1 4 0

64548167 T2 37 0

64548536 T3 13 0

64548537 T1 34 0

64548731 T1 14 0

64549587 T1 16 0

64549876 T1 13 0

64550045 T1 30 0

64550130 T1 1 0

64550255 T1 6 0

64550370 T1 21 0

64550483 T2 9 0

64550703 T2 5 0

64551341 T1 36 0

64551507 T3 4 0

64552128 T1 9 0

64552387 T1 4 0

64552502 T3 14 0

64552714 T1 12 0

64552878 T1 2 0

64552920 T1 1 0

64553245 T1 16 0

64553276 T1 12 0

64553472 T1 13 0

64554022 T1 6 0

64554460 T1 14 0

64554463 T1 2 0

64554467 T1 12 0

64554472 T1 8 0

64555667 T1 29 0

64555701 T1 2 0

64556504 T1 9 0

64556545 T2 19 1

64556663 T1 8 0

64556998 T2 18 0

64557110 T1 5 1

64557711 T1 21 0

64557712 T1 34 0

64557907 T3 10 0

64560035 T3 3 0

64560179 T2 6 0

64560254 T1 5 0

64560992 T1 12 0

64561014 T2 3 0

64561016 T2 18 0

64561153 T1 9 0

64561685 T1 9 0

64561836 T1 14 0

64562086 T1 59 0

64562450 T1 14 0

64562490 T1 25 0

64562543 T1 4 0

64562546 T3 17 0

64562597 T1 16 0

64562634 T1 10 0

64562658 T1 21 0

64562940 T1 18 0

64563085 T1 11 0

64563160 T1 1 0

64563163 T1 17 0

64563191 T1 4 0

64563742 T1 6 0

64563850 T1 12 0

64564396 T1 14 0

64564479 T1 2 0

64564694 T4 1 0

64565901 T1 9 0

64566652 T3 23 0

64566715 T1 4 0

64566778 T1 17 0

64567054 T1 25 0

64567178 T1 3 0

64567221 T1 1 0

64567260 T1 6 0

64568164 T1 9 0

64568248 T3 2 1

64568386 T1 13 0

64569122 T1 2 0

64569679 T1 30 0

64570664 T1 11 0

64571575 T1 21 0

64572038 T1 8 0

64572176 T1 3 0

64572229 T1 3 0

64572513 T1 8 0

64572723 T1 6 0

64572936 T1 5 0

64574040 T1 12 0

64574113 T1 44 0

64574184 T2 14 0

64574663 T1 23 0

64574777 T1 23 0

64575659 T2 21 1

64575688 T1 17 0

64576492 T1 19 0

64576695 T1 4 0

64577178 T2 17 0

64577268 T1 2 0

64577519 T2 32 0

64577626 T1 9 0

64578135 T1 4 0

64578137 T3 7 0

64578583 T3 2 0

64579249 T1 23 0

64580184 T1 16 0

64580291 T1 18 0

64580763 T1 10 0

64581181 T1 6 0

64581712 T1 27 0

64582114 T1 15 0

64583355 T1 15 0

64583598 T1 15 0

64583793 T1 25 0

64583799 T1 6 0

64584006 T1 7 0

64584017 T1 10 0

64585263 T1 67 0

64586649 T1 5 0

64587956 T1 19 0

64588294 T1 16 0

64589064 T1 14 4

64589296 T1 5 0

64589298 T1 10 0

64589301 T1 1 0

64589318 T1 1 0

64589321 T1 3 0

64589346 T1 10 0

64589348 T2 12 0

64589553 T1 1 0

64589559 T3 8 0

64589733 T1 14 0

64589745 T1 33 0

64590881 T1 9 0

64590884 T1 10 0

64590909 T1 6 0

64590922 T1 9 0

64592392 T1 10 0

64592746 T1 12 0

64592945 T2 36 0

64593592 T1 21 0

64593665 T1 16 0

64593682 T1 20 0

64594178 T1 3 0

64594246 T1 39 1

64594327 T1 8 0

64594373 T1 44 0

64594429 T1 48 0

64594506 T1 29 0

64594701 T1 6 0

64594789 T1 4 0

64595264 T1 2 0

64595507 T1 19 0

64596620 T1 8 0

64597146 T1 13 0

64597157 T1 22 0

64597174 T1 44 0

64597483 T1 10 0

64597485 T1 7 0

64598674 T1 2 0

64598676 T1 49 0

64598736 T1 61 0

64599340 T1 10 0

64599362 T1 7 0

64599506 T1 16 0

64599861 T3 10 0

64600885 T3 25 1

64601119 T2 3 0

64601137 T1 4 0

64601139 T1 2 0

64601237 T1 15 0

64602583 T1 9 0

64602594 T1 16 0

64602607 T1 1 0

64602648 T1 47 5

64602775 T3 19 0

64602791 T1 5 0

64602795 T1 2 0

64603087 T2 13 0

64603282 T3 17 0

64603626 T1 8 0

64603851 T1 15 0

64605331 T1 11 0

64605356 T1 18 0

64605368 T1 14 0

64605378 T3 24 0

64605730 T1 4 0

64605743 T1 24 0

64607409 T2 40 0

64608583 T1 9 0

64609458 T1 16 0

64609516 T1 33 0

64609655 T1 37 0

64610457 T1 2 0

64610753 T1 15 0

64610891 T1 23 0

64611219 T1 8 0

64611221 T1 33 0

64611376 T1 7 0

64611649 T2 24 0

64611718 T1 15 0

64611832 T1 32 0

64611833 T1 15 0

64611877 T1 14 0

64612141 T3 15 0

64612541 T1 22 0

64613277 T1 31 0

64613944 T1 37 0

64614227 T1 47 0

64614851 T1 9 0

64615147 T1 4 0

64616058 T3 13 0

64616064 T1 28 0

64616801 T3 13 4

64616956 T1 4 0

64617266 T1 16 0

64617307 T3 17 0

64617479 T1 14 0

64617690 T1 22 0

64618009 T1 29 0

64618136 T2 35 0

64618400 T3 18 0

64618402 T1 19 0

64619136 T1 2 0

64620036 T1 9 0

64620460 T2 5 0

64621264 T1 6 0

64621813 T1 3 0

64621852 T1 21 0

64621948 T1 2 0

64622246 T1 5 0

64622768 T2 16 3

64623434 T1 4 0

64623475 T1 25 0

64623604 T1 40 0

64623817 T1 23 0

64623957 T1 8 0

64625055 T3 12 0

64625368 T1 7 0

64625385 T2 10 0

64625389 T1 13 0

64625576 T1 9 0

64625746 T1 15 0

64625831 T1 32 0

64625858 T1 24 0

64626390 T4 29 0

64626454 T1 1 0

64626495 T1 7 0

64626594 T1 6 0

64626702 T2 7 0

64627323 T1 22 0

64627335 T1 16 0

64627438 T1 23 0

64627511 T1 44 0

64627512 T1 19 0

64627751 T4 7 0

64627914 T1 7 0

64627928 T3 10 0

64628004 T1 5 0

64628125 T1 5 0

64628600 T1 6 0

64628605 T1 6 0

64629310 T1 26 0

64629314 T2 11 0

64629317 T2 20 0

64629529 T3 32 0

64629769 T1 9 0

64630127 T1 17 0

64630499 T2 25 0

64630941 T2 14 0

64630943 T3 26 1

64631046 T1 22 0

64631417 T1 22 0

64631750 T2 14 0

64631975 T1 32 0

64632050 T1 29 0

64632565 T2 30 0

64632671 T1 19 0

64633200 T1 18 0

64633841 T1 16 1

64634047 T1 27 0

64634050 T1 7 0

64634458 T1 27 0

64634671 T1 26 0

64634674 T1 23 0

64636076 T1 2 0

64636338 T1 29 0

64636384 T1 12 0

64636792 T3 22 0

64636840 T1 11 0

64636869 T1 5 0

64637360 T1 27 0

64637459 T1 2 0

64638071 T1 16 0

64638516 T1 8 0

64638526 T1 2 0

64639185 T1 16 0

64639356 T2 11 0

64639723 T2 15 0

64639988 T1 13 4

64640029 T3 2 0

64640407 T1 4 0

64641129 T1 4 0

64641626 T2 15 0

64642614 T2 39 0

64642647 T1 21 0

64642814 T1 11 0

64643181 T1 13 0

64643210 T3 13 0

64643222 T2 26 0

64643224 T1 16 0

64643228 T2 11 0

64643239 T2 30 0

64643305 T1 18 0

64643513 T1 9 0

64643515 T1 1 0

64643703 T1 2 0

64643785 T1 5 0

64643800 T1 42 0

64645451 T1 21 0

64645894 T1 10 0

64646029 T1 29 0

64646329 T3 51 0

64646636 T1 21 0

64646643 T1 4 0

64646673 T1 14 0

64647039 T1 38 0

64647102 T1 1 0

64647222 T1 2 0

64647750 T1 2 0

64647754 T1 8 0

64648409 T1 32 0

64648505 T1 17 0

64648710 T1 12 0

64648731 T1 9 0

64648810 T2 6 3

64649080 T1 7 0

64650524 T1 9 0

64650738 T1 16 0

64651124 T1 13 0

64651373 T1 17 0

64651525 T1 9 0

64652279 T1 13 0

64653125 T1 3 0

64653701 T1 4 0

64654259 T1 4 0

64654651 T2 31 0

64654668 T1 26 0

64654989 T3 41 0

64654994 T1 31 0

64655007 T1 33 0

64655489 T1 25 0

64655550 T2 11 0

64656473 T1 17 0

64656989 T1 9 0

64656993 T3 9 1

64656998 T1 3 0

64657060 T1 13 0

64657734 T1 24 0

64658302 T1 5 1

64658339 T1 29 0

64658404 T1 19 2

64658633 T1 4 0

64658712 T1 12 0

64659187 T1 13 0

64660075 T1 21 0

64660083 T1 42 0

64660704 T1 8 0

64660705 T1 2 0

64660877 T1 26 0

64661075 T1 15 2

64661159 T3 51 0

64661866 T2 37 0

64661867 T1 12 0

64662040 T1 13 0

64662185 T1 12 0

64662281 T1 11 0

64663024 T3 15 0

64663049 T3 28 0

64663158 T1 16 0

64663320 T1 13 0

64663327 T1 25 0

64663329 T1 61 0

64663336 T1 38 0

64663348 T2 53 0

64663408 T1 29 0

64663412 T1 52 0

64663430 T1 21 0

64663497 T1 34 0

64663635 T1 8 0

64663905 T1 3 0

64663908 T3 3 0

64663994 T2 24 0

64664433 T1 5 0

64664839 T1 18 0

64666089 T1 31 0

64666408 T1 27 1

64666851 T1 10 0

64666857 T1 5 0

64667216 T1 15 0

64667248 T1 16 0

64667601 T2 17 0

64667933 T1 15 0

64667937 T1 6 0

64668006 T3 28 0

64668025 T1 31 0

64668195 T1 14 0

64668204 T3 10 2

64669080 T1 18 0

64669254 T3 56 0

64669551 T2 9 0

64669553 T1 14 0

64669630 T3 16 0

64669644 T1 18 0

64669811 T1 6 0

64670670 T1 6 0

64670746 T1 33 0

64670748 T1 24 0

64671225 T2 9 0

64671276 T1 23 0

64671280 T1 19 0

64671288 T2 15 0

64671388 T1 12 0

64671404 T1 17 0

64671419 T3 18 0

64671597 T2 26 0

64671744 T1 7 0

64672431 T1 41 0

64672602 T1 17 0

64672654 T1 2 0

64672839 T1 12 2

64672843 T1 11 0

64673076 T2 44 0

64673290 T1 34 0

64673311 T1 22 0

64673471 T1 25 0

64674859 T1 17 0

64675094 T3 21 0

64675117 T1 1 0

64676452 T1 3 0

64676486 T1 7 0

64676493 T1 17 0

64676722 T1 7 0

64676800 T1 26 0

64676914 T2 8 0

64677836 T1 17 0

64678030 T1 1 0

64678064 T1 9 0

64678123 T1 14 0

64678372 T1 8 0

64678373 T1 10 0

64678378 T1 11 0

64678702 T1 21 0

64679188 T1 6 0

64679270 T2 29 0

64679271 T1 20 0

64679463 T1 14 0

64679935 T1 18 0

64680155 T1 25 0

64680432 T1 47 0

64680433 T1 41 0

64680443 T1 86 0

64680451 T1 11 0

64680703 T1 9 0

64680931 T1 22 0

64681607 T1 23 0

64682168 T1 1 0

64682641 T1 16 1

64682750 T1 9 0

64682994 T1 11 0

64683163 T2 28 0

64683543 T2 11 0

64683791 T2 18 0

64683796 T1 3 0

64683801 T3 11 0

64683910 T1 10 0

64683985 T1 9 0

64684066 T2 42 0

64684173 T1 44 0

64684176 T2 26 0

64684304 T2 13 0

64684305 T2 21 0

64684752 T2 20 0

64685697 T1 21 0

64685934 T3 34 0

64685938 T1 50 0

64685951 T1 15 0

64686356 T1 8 0

64687140 T3 19 3

64687385 T1 58 0

64687386 T2 37 0

64687390 T1 28 0

64687407 T3 50 0

64687473 T2 26 0

64687973 T1 19 0

64687978 T1 13 0

64688411 T1 18 0

64688547 T2 7 0

64689161 T1 47 0

64689164 T1 1 0

64689379 T1 24 0

64689385 T3 33 0

64689433 T2 46 0

64689497 T1 3 0

64689598 T2 37 0

64689731 T2 25 0

64689815 T1 7 0

64689818 T1 3 0

64689823 T1 13 0

64690070 T1 1 0

64690714 T3 10 0

64691175 T1 17 0

64691327 T3 6 0

64692379 T1 5 1

64692481 T1 37 0

64693771 T2 14 0

64694705 T1 15 0

64695293 T1 43 0

64696819 T1 19 0

64697021 T3 3 0

64697027 T1 6 0

64697347 T3 21 0

64697550 T1 2 0

64698250 T1 12 0

64698256 T1 41 0

64698525 T2 14 0

64698691 T1 20 0

64698959 T1 2 0

64699152 T1 34 0

64699678 T1 23 0

64699937 T2 10 0

64699973 T2 16 4

64700199 T3 9 0

64700308 T1 4 0

64700539 T1 17 0

64700546 T1 29 1

64701168 T1 1 0

64701653 T1 14 0

64702070 T1 16 0

64702589 T2 36 0

64702691 T1 7 0

64703414 T1 4 0

64704110 T1 9 0

64707783 T1 5 0

64708119 T1 6 1

64710298 T2 27 0

64710409 T3 77 0

64711370 T2 12 0

64711761 T1 39 0

64713529 T1 17 0

64716761 T1 23 0

64716902 T1 18 0

64717078 T1 19 0

64717154 T1 24 0

64717157 T1 14 0

64717177 T1 38 6

64717183 T1 10 0

64717361 T2 13 0

64717629 T2 21 0

64717778 T1 13 0

64717780 T1 34 0

64717782 T1 9 0

64718365 T1 17 0

64718372 T1 7 0

64718397 T1 10 0

64718406 T1 3 0

64718416 T2 10 0

64718539 T2 20 0

64718572 T1 11 0

64718581 T1 13 0

64718600 T1 20 0

64718979 T2 9 0

64718984 T2 37 0

64719337 T1 7 0

64721069 T1 17 0

64721122 T2 18 0

64721511 T1 45 0

64721619 T1 20 0

64723225 T1 4 0

64723609 T2 12 0

64723625 T2 17 1

64723792 T1 23 0

64724270 T1 37 0

64725151 T2 34 0

64725180 T3 1 0

64725185 T1 29 0

64725425 T1 23 0

64725450 T1 4 0

64725557 T1 20 0

64726107 T1 12 0

64726122 T1 25 0

64726237 T2 19 0

64726524 T1 27 0

64726946 T1 4 0

64727051 T1 24 0

64727162 T2 18 0

64727292 T1 2 0

64728544 T1 6 0

64729316 T1 12 0

64729717 T1 4 0

64729860 T1 21 0

64730510 T1 16 0

64730530 T2 8 0

64731485 T1 44 0

64731495 T1 11 0

64732187 T1 11 0

64732271 T1 4 0

64732288 T2 7 0

64733078 T2 38 2

64733097 T1 5 0

64733280 T2 6 2

64733583 T1 31 0

64733626 T1 15 0

64734547 T1 36 0

64734902 T1 20 0

64735385 T1 5 0

64737202 T1 33 0

64739047 T2 30 0

64740311 T1 37 0

64740314 T1 27 0

64741387 T2 54 0

64741659 T1 7 0

64741665 T1 18 0

64741802 T1 15 0

64741814 T1 11 0

64741822 T1 13 0

64741853 T1 12 0

64741857 T1 5 0

64741876 T1 1 0

64742130 T1 21 0

64742186 T1 2 0

64742193 T1 24 2

64742712 T1 4 1

64743409 T1 12 0

64745293 T1 1 0

64745306 T1 16 0

64746039 T1 10 0

64746119 T1 8 0

64746129 T1 7 0

64747302 T3 31 0

64747488 T1 63 0

64750298 T2 21 0

64750801 T1 12 0

64750803 T1 14 0

64750805 T1 11 0

64750808 T2 16 0

64751107 T1 12 0

64752438 T3 28 0

64753164 T1 19 0

64753217 T1 3 0

64753243 T1 7 0

64754496 T2 36 0

64756036 T1 47 0

64756739 T1 3 0

64757450 T1 43 0

64758216 T1 47 0

64758378 T1 20 0

64760202 T1 20 0

64760465 T4 5 0

64761905 T1 4 0

64763135 T1 36 0

64763363 T1 22 0

64763364 T1 24 0

64763369 T1 6 0

64764507 T1 6 0

64766341 T1 23 0

64767607 T2 54 0

64768277 T3 10 0

64773528 T1 4 1

65858455 T1 2 0

65864758 T1 7 0

65873023 T3 36 0

65900309 T3 2 0

65901666 T1 27 0

65910039 T2 2 0

65925842 T1 31 0

65925885 T3 6 0

65930380 T1 15 0

65933114 T1 4 0

65933493 T1 21 0

65937694 T1 2 0

65940969 T1 11 0

65943360 T2 11 0

65948857 T3 2 0

65966794 T3 7 0

65980574 T1 28 0

66062353 T1 22 0

66062840 T1 13 0

66075852 T1 18 0

66075860 T1 6 0

66075864 T1 12 0

66077127 T1 10 0

66116040 T1 13 0

67282466 T3 8 0

74194251 T2 15 0

74194868 T1 4 0

74196264 T3 23 0

74197518 T2 4 0

74198304 T2 5 0

74199041 T1 5 0

74199083 T1 7 0

74201805 T1 3 0

74202230 T1 24 0

74202969 T1 18 0

74204200 T1 9 0

74205033 T1 9 0

74206693 T1 17 0

74206824 T2 10 0

74207011 T1 14 0

74207175 T1 6 0

74207509 T1 21 0

74207527 T2 18 0

74208133 T2 13 0

74208180 T1 14 0

74209346 T1 4 0

74209950 T1 15 0

74210056 T1 6 0

74211303 T1 11 0

74211849 T1 15 0

74212302 T1 8 0

74212585 T1 7 0

74212646 T1 5 0

74213108 T1 4 0

74213111 T2 13 0

75000076 T1 13 0

75000077 T1 10 1

75000079 T1 12 0

75000082 T1 10 0

75000111 T1 18 0

75000186 T1 7 0

75000215 T1 18 0

75000238 T1 11 0

75001100 T1 2 0

75002208 T1 12 0

75002254 T1 7 0

75002736 T4 19 1

75002931 T1 12 0

75003129 T1 14 0

75003150 T1 16 0

75003440 T1 15 0

75003563 T2 1 0

75003916 T2 21 0

75004123 T2 17 0

75004646 T1 1 0

75005330 T1 5 0

75005344 T2 7 0

75006268 T1 7 0

75006355 T1 14 0

75008006 T3 1 0

75008265 T2 57 0

75008267 T1 25 0

75008270 T1 32 0

75008276 T1 11 0

75008411 T1 3 0

75008432 T1 14 0

75008455 T1 3 0

75008476 T1 27 0

75008612 T1 19 1

75009150 T1 3 0

75009184 T1 3 0

75010015 T1 18 0

75010496 T1 5 0

75011131 T1 7 0

75011610 T1 13 0

75011787 T1 8 0

75012213 T2 2 0

75012701 T1 8 0

75013253 T1 5 0

75013426 T1 5 0

75013610 T1 7 0

75013755 T1 24 0

75014317 T1 7 0

75014341 T1 13 0

75014861 T1 22 0

75015899 T3 4 0

75015923 T3 14 0

75016319 T1 4 0

75016337 T1 6 0

75016452 T1 5 0

75018970 T1 9 0

75020432 T1 5 0

75021647 T1 8 0

75021685 T1 16 0

75022000 T1 6 0

75022315 T1 11 0

75022327 T1 6 0

75022336 T1 6 0

75022337 T1 10 0

75022340 T1 4 0

75023128 T2 13 1

75023226 T1 9 0

75023479 T1 7 0

75023709 T1 10 0

75023805 T1 6 0

75023870 T1 6 0

75023878 T1 10 0

75024071 T2 25 0

75024335 T2 10 1

75024362 T1 14 0

75024402 T1 15 0

75024594 T1 5 0

75024703 T1 4 0

75024776 T2 9 0

75024875 T1 11 0

75025068 T1 6 0

75025535 T1 10 0

75025631 T2 10 1

75026054 T1 6 0

75026110 T2 3 0

75026546 T1 3 0

75026766 T1 36 0

75027037 T1 14 0

75028128 T1 3 0

75029145 T1 5 0

75029338 T1 2 0

75029356 T1 20 0

75029637 T1 2 0

75029640 T1 6 0

75029667 T1 5 0

75029677 T1 1 0

75029757 T1 3 0

75030279 T1 5 0

75030442 T1 11 0

75030448 T1 16 0

75030484 T1 4 0

75030923 T1 11 0

75030980 T1 26 0

75031698 T1 9 0

75032562 T1 3 0

75032610 T1 5 0

75032615 T1 4 0

75032976 T1 12 0

75033184 T1 13 0

75033187 T1 5 0

75033191 T1 8 0

75033211 T1 3 0

75034832 T1 8 0

75035344 T2 18 0

75035379 T1 20 0

75035951 T1 2 0

75036409 T1 3 0

75036460 T1 20 0

75036983 T1 7 0

75037050 T1 23 0

75037332 T1 2 0

75038569 T1 6 0

75038850 T2 20 0

75038860 T2 23 0

75038882 T1 13 0

75039170 T1 5 0

75040279 T2 22 0

75040772 T1 3 0

75041108 T1 9 0

75041336 T1 16 0

75041502 T1 6 0

75042769 T1 30 0

75042772 T1 6 0

75042788 T1 3 0

75042793 T1 5 0

75042796 T1 2 0

75042832 T3 11 0

75042853 T1 21 0

75042870 T1 5 0

75042933 T1 7 0

75043237 T1 8 0

75043398 T2 9 0

75043425 T1 17 0

75043626 T2 10 1

75045067 T1 6 0

75046163 T2 13 0

75046316 T2 3 0

75047178 T1 11 0

75047283 T1 14 0

75047381 T1 5 0

75047528 T1 1 0

75048711 T1 1 0

75048892 T1 15 0

75048904 T1 12 0

75048907 T1 21 0

75048972 T1 8 0

75049047 T1 4 0

75049056 T1 8 0

75049728 T1 19 0

75049974 T1 19 0

75050774 T1 4 0

75051615 T1 11 0

75051624 T1 17 0

75051626 T1 16 0

75051912 T1 1 0

75051917 T1 6 0

75052025 T1 3 0

75052157 T1 33 0

75052158 T1 12 0

75052228 T3 22 0

75052459 T2 41 0

75052882 T1 7 0

75053150 T1 5 0

75053931 T1 3 0

75054379 T3 21 0

75054668 T2 50 0

75055137 T1 19 0

75055233 T1 2 0

75055257 T1 13 0

75055408 T2 7 0

75055440 T1 21 1

75055480 T1 18 0

75055491 T1 14 0

75055507 T1 16 0

75055512 T1 5 0

75055542 T1 14 0

75056508 T1 14 0

75056517 T1 12 0

75056919 T1 16 0

75057596 T1 7 0

75058047 T1 26 0

75058461 T1 13 0

75058816 T1 4 0

75059656 T1 12 0

75059659 T1 15 0

75059663 T1 1 0

75059665 T1 18 0

75059738 T1 12 0

75060798 T1 44 0

75060804 T1 17 0

75061413 T1 4 0

75061519 T1 2 0

75061537 T1 15 0

75061548 T3 10 0

75063214 T1 27 0

75063500 T1 20 0

75064203 T1 5 0

75064677 T1 8 0

75066040 T1 6 0

75066100 T1 13 0

75066137 T1 11 0

75066188 T1 5 0

75066238 T2 7 0

75066273 T1 5 1

75066677 T3 15 0

75066710 T1 4 0

75067137 T1 12 0

75067237 T1 19 0

75067283 T1 12 0

75067901 T1 7 0

75068699 T1 16 0

75068704 T1 5 0

75068802 T1 18 0

75069212 T3 20 0

75070440 T1 14 0

75070454 T3 1 0

75070746 T2 29 0

75071293 T2 6 0

75071914 T1 2 0

75071917 T1 1 0

75071987 T1 8 0

75072004 T2 8 0

75072023 T1 6 0

75072040 T1 5 0

75072097 T1 22 0

75072182 T1 22 0

75073431 T1 12 0

75073601 T1 21 0

75073675 T1 8 0

75074128 T1 8 0

75074405 T1 2 0

75074854 T1 23 0

75074872 T1 41 0

75075127 T2 1 0

75075292 T1 4 0

75075295 T1 5 0

75075630 T1 10 0

75076244 T2 18 0

75077285 T1 27 0

75077371 T1 7 0

75077454 T1 16 0

75077480 T1 10 0

75077506 T3 9 4

75077896 T1 2 0

75077984 T1 18 0

75078040 T1 8 0

75078084 T1 2 0

75078138 T1 5 0

75078378 T1 6 0

75078562 T1 3 0

75079736 T1 28 0

75079778 T2 8 0

75079781 T1 11 0

75080910 T2 5 0

75080963 T1 4 0

75081101 T1 22 0

75081882 T1 3 0

75082154 T1 7 0

75082157 T1 4 0

75082668 T1 4 0

75083379 T1 14 0

75083427 T2 8 0

75083835 T1 4 0

75083836 T1 5 0

75084747 T2 4 0

75085704 T1 2 0

75085735 T1 20 0

75086329 T1 30 0

75086587 T1 4 0

75087557 T1 4 0

75087799 T1 9 0

75087830 T3 8 0

75087900 T1 4 0

75088123 T1 8 0

75088665 T1 17 0

75088960 T1 21 0

75091052 T1 2 0

75091316 T1 12 0

75091381 T1 23 0

75091387 T1 6 0

75091837 T1 9 0

75091963 T1 5 0

75092089 T1 11 0

75092705 T1 14 0

75093585 T1 7 0

75093592 T1 14 0

75093663 T1 3 0

75093789 T1 19 0

75093859 T1 28 0

75094440 T1 5 0

75094751 T1 19 0

75094775 T1 7 0

75095766 T1 12 0

75096467 T1 9 0

75096817 T1 4 0

75096885 T1 4 0

75099705 T1 13 0

75099789 T1 1 0

75100994 T1 7 0

75101124 T1 11 0

75101187 T1 6 0

75101263 T1 13 0

75101337 T1 8 0

75101607 T1 13 0

75101955 T1 15 0

75102008 T1 7 0

75102131 T1 14 0

75102134 T1 13 0

75102192 T1 14 0

75102227 T1 19 0

75103083 T1 37 0

75103099 T1 39 0

75103438 T1 39 0

75103441 T1 7 0

75103562 T1 21 0

75110406 T1 19 0

75110593 T1 8 0

75110743 T1 1 0

75111135 T1 5 0

75111150 T1 16 0

75111522 T1 31 0

75111594 T1 2 0

75112036 T1 6 0

75112117 T1 6 0

75112977 T1 3 0

75112989 T2 2 0

75113129 T4 3 1

75113224 T2 2 0

75113755 T1 3 0

75113991 T1 13 0

75114354 T1 6 0

75115369 T2 3 0

75116158 T1 5 0

75116197 T1 2 0

75116205 T1 12 0

75116220 T3 7 0

75116975 T1 21 0

75117137 T3 6 0

75117175 T1 10 0

75117386 T1 4 0

75117746 T1 7 0

75117851 T3 32 0

75118037 T1 2 0

75118198 T1 2 0

75118635 T1 6 0

75118839 T1 16 0

75121014 T1 4 0

75121079 T1 2 0

75121081 T1 27 0

75122221 T1 10 0

75122235 T1 5 0

75122419 T1 8 0

75122427 T1 18 0

75122477 T1 5 0

75122654 T1 5 0

75122658 T1 2 0

75122896 T2 23 0

75123086 T1 29 0

75123163 T1 26 0

75123344 T1 8 0

75123832 T1 17 0

75124321 T1 27 0

75124471 T1 30 0

75124647 T1 14 0

75125795 T1 7 0

75125838 T1 8 0

75125848 T2 9 0

75125902 T3 15 6

75125948 T1 29 0

75127072 T2 10 0

75127176 T1 12 0

75127607 T1 20 0

75127626 T1 8 0

75127631 T1 15 0

75127652 T2 5 0

75127660 T1 4 0

75127699 T1 6 0

75127708 T1 1 0

75128045 T1 5 0

75128259 T1 28 0

75128347 T1 12 0

75128360 T2 66 1

75128397 T2 21 0

75128499 T1 9 0

75128794 T1 4 0

75129135 T1 32 1

75129447 T1 7 0

75129472 T1 7 0

75129934 T1 5 0

75130136 T1 15 0

75130180 T1 12 0

75130258 T1 21 0

75130263 T1 8 0

75130268 T1 22 0

75130356 T1 4 0

75130385 T3 6 0

75130866 T1 1 0

75132469 T1 10 0

75133179 T1 7 0

75133669 T1 6 0

75134035 T1 25 0

75134293 T1 6 0

75134310 T1 19 0

75134360 T1 3 0

75134389 T1 7 0

75134393 T2 9 0

75134405 T1 11 0

75134467 T1 28 0

75135243 T1 24 0

75136436 T1 11 0

75137363 T2 20 0

75137378 T1 2 0

75137430 T1 3 0

75138089 T1 3 0

75138110 T1 5 0

75138727 T1 6 0

75138729 T1 10 0

75138844 T1 31 0

75139124 T2 1 0

75139251 T1 8 0

75139312 T1 6 0

75139591 T1 13 0

75139632 T1 17 0

75139691 T1 19 0

75139692 T1 32 0

75139695 T1 11 0

75140229 T1 7 3

75140300 T1 40 0

75140366 T1 17 0

75140369 T1 46 0

75140549 T1 33 0

75140658 T1 6 0

75140720 T1 3 0

75141709 T1 16 0

75142249 T1 7 0

75142490 T1 2 0

75142572 T1 1 0

75143351 T1 2 0

75144449 T2 11 0

75144601 T1 7 0

75144988 T1 13 0

75145061 T3 2 0

75146209 T1 11 0

75146229 T1 7 0

75146281 T1 16 0

75146301 T1 13 0

75146951 T1 21 0

75147182 T1 22 0

75147200 T1 13 0

75147205 T1 7 0

75147219 T1 14 0

75147243 T1 17 0

75147287 T1 16 0

75147356 T1 25 1

75147951 T1 25 0

75147985 T1 49 0

75148353 T1 9 0

75149448 T1 9 0

75149455 T1 6 0

75149492 T1 8 0

75149551 T3 3 0

75149593 T1 9 0

75150970 T1 16 0

75154521 T1 9 0

75154601 T1 26 0

75154604 T1 7 0

75154848 T1 6 0

75155124 T2 3 0

75155555 T1 8 0

75155897 T1 7 0

75155916 T1 5 0

75155941 T3 5 0

75156089 T1 14 0

75158503 T1 1 0

75160933 T1 11 0

75172947 T2 14 0

75193290 T1 35 0

75193824 T1 2 0

75193981 T1 13 0

75194133 T1 4 0

75194153 T1 2 0

75194867 T1 9 0

78000684 T1 31 0

78077875 T1 24 0

78079358 T1 7 0

78079361 T1 5 0

78079369 T1 13 0

78080795 T1 6 1

78080801 T1 4 0

78081702 T3 9 1

78081845 T1 30 0

78081936 T3 37 0

78082214 T1 14 0

78082572 T1 7 0

78082757 T1 13 0

78083787 T1 48 0

78084957 T1 2 0

78084960 T1 31 0

78085530 T1 8 0

78086920 T1 9 0

78086921 T1 24 0

78086923 T1 9 0

78087554 T1 1 0

78088623 T1 23 0

78088626 T1 5 0

78088635 T1 1 0

78088853 T1 9 0

78089081 T1 5 0

78089083 T1 5 0

78089555 T1 29 0

78090661 T1 5 0

78090902 T3 4 0

78090905 T1 19 0

78092526 T1 36 0

78092626 T1 15 0

78092805 T1 11 0

78093315 T2 14 3

78093598 T1 7 0

78093718 T1 9 0

78094607 T1 17 0

78095970 T1 20 0

78097013 T1 19 0

78097015 T2 9 0

78097791 T1 4 0

78098288 T1 4 0

78098859 T1 9 0

78099632 T1 6 0

78099633 T1 6 0

78099638 T1 7 0

78099782 T3 1 1

78100076 T2 18 5

78100901 T3 20 0

78101094 T1 6 0

78102393 T1 7 0

78102466 T3 14 0

78102885 T1 4 0

78102895 T1 8 0

78103093 T1 15 0

78105454 T2 8 0

78109835 T1 23 0

78109970 T1 6 0

78110409 T1 2 0

78111174 T1 4 0

78112033 T1 5 0

78113116 T3 14 0

78113124 T1 8 0

78113380 T1 3 0

78113711 T1 21 0

78115206 T1 14 0

78115512 T1 12 0

78115812 T1 17 1

78116126 T1 5 1

78116348 T1 7 0

78116559 T1 19 0

78117635 T1 1 0

78117803 T1 15 0

78117887 T3 9 0

78118018 T1 27 4

78119024 T1 11 0

78120164 T1 10 0

78121322 T1 1 0

78121535 T1 3 0

78121537 T1 10 0

78121544 T1 6 0

78121548 T1 9 0

78123361 T2 31 2

78123473 T1 4 0

78124215 T1 4 0

78124722 T1 22 0

78124991 T1 9 0

78125010 T2 5 3

78125297 T1 11 0

78125312 T1 11 0

78125350 T1 22 0

78125534 T1 14 0

78125588 T1 29 0

78125628 T1 4 0

78126620 T1 10 0

78126881 T1 4 0

78127361 T3 14 0

78127749 T1 11 0

78128865 T1 12 0

78130235 T3 24 0

78130380 T1 29 0

78130566 T1 5 0

78130603 T1 5 0

78130745 T1 12 0

78131031 T1 13 0

78131623 T1 7 0

78131884 T1 3 0

78133782 T3 10 0

78135382 T1 28 0

78135395 T1 7 0

78135413 T1 13 0

78135447 T2 3 0

78135474 T1 21 0

78135938 T1 16 0

78136600 T1 6 0

78136730 T1 12 0

78136805 T1 11 0

78137797 T1 11 0

78138193 T1 14 0

78138410 T1 6 0

78138418 T1 6 0

78139843 T1 5 0

79022993 T1 7 0

79023115 T1 11 0

79024114 T1 8 0

79024157 T2 4 2

79024162 T1 25 0

79024691 T2 5 0

79025748 T2 11 0

79027081 T1 6 0

79028315 T1 3 0

79029650 T1 19 0

79029652 T1 13 0

79029701 T2 7 0

79029702 T1 6 0

79029714 T1 6 0

79029729 T1 15 0

79029794 T2 18 0

79029829 T2 5 0

79029834 T3 6 0

79030321 T1 9 0

79030568 T1 9 0

79030611 T1 5 0

79030771 T1 2 0

79032357 T1 7 0

79032363 T1 6 0

79034547 T1 10 0

79034604 T1 3 0

79034619 T2 6 0

79034697 T1 11 0

79034770 T1 2 0

79034791 T1 7 0

79034792 T1 9 0

79034798 T1 15 0

79034895 T3 3 3

79034949 T1 4 0

79035507 T1 9 0

79035553 T1 5 0

79035557 T1 4 0

79035561 T1 13 0

79035562 T1 2 0

79035568 T1 12 0

79035809 T1 3 0

79036716 T1 6 0

79036752 T1 1 0

79037890 T1 1 0

79038033 T1 6 1

79038035 T4 11 0

79038120 T1 15 0

79038125 T1 5 0

79038126 T1 9 0

79038819 T1 4 0

79041121 T1 4 0

79041200 T1 7 0

79041211 T1 4 0

79041214 T1 6 0

79041231 T1 8 0

79041513 T1 2 0

79041730 T1 18 0

79041804 T2 14 0

79042132 T1 3 0

79042472 T1 7 0

79042560 T1 4 0

79044742 T1 4 0

79045434 T1 7 0

79047074 T1 3 0

79047899 T2 5 4

79048617 T1 2 2

79101897 T2 3 0

79103338 T1 25 0

79103804 T1 4 0

79106006 T1 14 0

79106499 T1 1 0

79107000 T1 17 0

79109504 T2 4 0

79111209 T1 4 0

79111615 T1 2 0

79113413 T2 5 0

79114499 T1 5 0

79116617 T1 12 0

79116971 T1 5 0

79117498 T1 2 0

79118888 T1 6 0

79119632 T1 11 0

79120450 T1 4 0

79155030 T1 11 0

79155436 T1 2 0

79155594 T1 9 0

79161735 T2 2 0

79188484 T1 1 0

79190559 T1 2 0

79190955 T1 13 0

79192065 T1 1 0

79192253 T1 25 0

79192420 T2 3 0

79192557 T1 15 1

79196263 T1 30 0

80003378 T1 24 0

80032630 T2 16 0

80032782 T1 2 0

80033466 T1 9 0

80036086 T1 1 0

80036189 T2 1 0

80037463 T1 10 0

80038044 T1 8 0

80041642 T1 16 0

80041868 T1 38 0

80042022 T1 19 0

80042039 T1 23 0

80042101 T1 12 0

80042312 T1 13 1

80042415 T1 18 0

80043061 T1 20 0

80043123 T1 20 0

80043137 T4 24 0

80043431 T3 15 0

80043523 T1 15 0

80043873 T1 25 0

80044167 T2 45 0

80044428 T1 14 0

80044719 T1 6 0

80044750 T1 1 0

80044834 T1 8 0

80046383 T1 6 0

80046791 T1 7 0

80047908 T1 27 0

80048083 T1 18 0

80048789 T2 3 0

80049023 T1 6 0

80049029 T1 4 0

80049053 T1 8 0

80049144 T1 27 0

80049479 T1 27 0

80049487 T1 14 0

80049552 T2 5 0

80049653 T1 1 0

80049711 T1 7 0

80050007 T1 12 0

80052160 T1 11 0

80052359 T2 24 0

80052496 T2 3 0

80052746 T1 4 0

80053859 T3 10 0

80054582 T1 24 0

80055248 T1 6 0

80055445 T2 5 0

80055632 T1 3 0

80095380 T1 12 0

80095895 T1 25 0

80097143 T1 4 0

80097156 T1 11 0

80097497 T1 2 0

80097530 T1 7 0

80098064 T1 5 0

80098067 T1 9 0

80098107 T1 15 0

80098734 T1 3 0

80099169 T1 6 0

80100569 T1 4 0

80101121 T1 3 0

80101488 T1 6 0

80101789 T2 20 0

80103891 T1 7 0

80104940 T1 2 0

80105109 T1 15 0

80105424 T1 1 0

80118710 T1 4 0

80118753 T1 3 0

80119214 T1 20 0

80123431 T1 47 0

83256171 T1 8 0

83324631 T1 11 0

87017018 T3 20 0

87057540 T1 19 0

89585900 T2 15 0

89600554 T1 4 0

89606373 T1 6 0

89610499 T1 2 0

89610945 T1 9 0

89611058 T1 2 0

89611945 T2 7 0

89612988 T1 6 0

89617872 T1 10 0

89620289 T1 11 0

89620292 T1 5 0

89622073 T1 6 0

89626687 T1 10 0

89631406 T1 2 0

89632368 T1 11 0

89633210 T1 1 0

89633263 T2 2 0

89633923 T1 23 0

89640106 T1 2 0

89641942 T1 8 0

89642060 T1 3 0

89642101 T1 2 0

89642187 T2 4 0

89642203 T1 6 0

89643879 T1 15 0

89643994 T1 2 0

89644022 T1 5 0

89644054 T1 22 0

89645256 T1 20 0

89645628 T1 1 0

89646904 T1 6 0

89648191 T1 19 0

89648505 T1 11 0

89648897 T2 5 0

89650122 T2 8 3

89650386 T3 2 0

89650471 T1 13 0

89650618 T3 2 0

89650700 T1 3 0

89651177 T2 6 0

89651308 T1 1 0

89651474 T1 15 0

89651575 T1 6 0

89651700 T1 16 0

89651992 T1 4 0

89652073 T3 23 0

89652470 T1 7 0

89652522 T1 4 0

89652631 T1 9 0

89652755 T1 24 0

89652871 T1 11 0

89652915 T1 8 0

89653564 T1 6 0

89653842 T2 4 0

89654003 T1 12 0

89655162 T1 8 0

89655170 T1 7 0

89655196 T1 17 0

89655204 T1 3 0

89655317 T1 6 0

89655813 T1 16 0

89655859 T1 15 0

89656008 T1 14 0

89656093 T1 11 0

89656129 T1 2 0

89656340 T3 6 0

89656633 T1 18 0

89656670 T1 22 0

89656739 T2 9 0

89656812 T1 6 0

89657076 T1 9 0

89657286 T1 6 0

89658262 T1 2 0

89658926 T1 2 0

89658980 T1 1 0

89659042 T1 4 0

89659145 T1 3 0

89659244 T1 15 0

89659351 T1 5 0

89660591 T1 3 0

89661513 T1 6 0

89661919 T1 3 0

89662153 T1 8 0

89662427 T1 4 0

89662755 T1 3 0

89662772 T1 17 0

89663293 T1 11 0

89663529 T1 25 0

89663558 T1 12 0

89663563 T1 9 0

89663641 T1 23 0

89663924 T1 16 0

89663965 T1 11 0

89664713 T2 17 0

89664941 T1 2 0

89665264 T1 9 0

89665358 T1 2 0

89666077 T1 6 0

89667159 T2 4 0

89667216 T1 1 0

89667634 T1 3 0

89667743 T1 8 0

89667938 T3 37 0

89668101 T1 1 0

89668175 T1 35 0

89668295 T1 18 0

89668733 T1 11 0

89669885 T1 3 0

89670107 T1 2 0

89670193 T1 3 0

89670663 T1 15 0

89670806 T1 15 0

89671381 T1 2 0

89671543 T1 12 0

89671751 T1 14 0

89672156 T1 1 0

89672531 T1 11 0

89672552 T1 8 0

89672681 T1 6 0

89672768 T1 7 6

89673096 T3 18 0

89673930 T1 16 0

89674258 T1 5 0

89674284 T1 18 0

89674382 T3 8 0

89674430 T1 9 0

89674435 T1 25 0

89675540 T1 4 0

89676017 T1 8 1

89676294 T1 12 0

89676513 T1 4 0

89676565 T1 6 0

89676658 T1 2 0

89677114 T1 11 0

89677263 T1 2 0

89677366 T3 4 0

89677925 T2 10 3

89677984 T1 11 0

89678161 T1 8 0

89679181 T1 7 0

89679550 T1 2 0

89680322 T1 1 0

89680413 T1 3 0

89680618 T1 12 0

89681080 T1 8 0

89681245 T1 29 0

89681318 T1 1 0

89682198 T1 3 0

89683089 T1 9 1

89683212 T1 2 0

89683747 T1 3 0

89683748 T2 29 0

89684330 T1 6 1

89684594 T1 20 0

89684710 T1 7 0

89684715 T1 5 0

89684746 T1 6 0

89685315 T1 21 0

89685359 T1 9 0

89685548 T1 5 0

89685565 T1 8 0

89686076 T1 9 0

89686090 T1 23 0

89686138 T3 6 0

89686181 T1 5 0

89686414 T2 4 0

89687039 T1 16 0

89687276 T1 22 0

89687433 T1 11 0

89688279 T1 12 0

89689180 T1 20 0

89689363 T1 24 0

89689794 T1 8 0

89690103 T1 8 0

89690127 T1 16 0

89690164 T1 19 0

89690640 T1 33 0

89691158 T1 10 0

89691339 T1 11 0

89691374 T1 20 0

89691401 T1 6 0

89691829 T1 15 3

89691891 T1 12 0

89692134 T1 12 0

89693015 T1 20 0

89693510 T1 8 0

89694088 T1 9 0

89694400 T2 20 0

89694748 T1 3 0

89694752 T1 1 0

89694830 T3 16 0

89695046 T3 18 0

89695823 T1 7 0

89696719 T1 1 0

89696847 T1 1 0

89697655 T1 17 0

89698133 T1 5 0

89698194 T1 7 0

89698997 T1 17 0

89700000 T1 13 0

89702943 T1 9 0

89704676 T1 4 0

89705870 T1 21 0

89706323 T1 6 0

89706365 T1 8 0

89706444 T1 4 0

89706460 T2 18 0

89706897 T1 5 0

89707046 T1 13 0

89707101 T1 3 0

89707108 T1 13 0

89707148 T1 14 0

89708069 T2 27 0

89708356 T2 3 0

89708605 T1 4 0

89709004 T1 2 0

89709540 T1 6 0

89709978 T1 17 0

89710001 T1 16 0

89710016 T1 2 0

89710205 T1 1 1

89710618 T3 39 0

89710646 T1 3 0

89710893 T2 5 0

89710942 T1 26 0

89711052 T1 3 0

89711370 T1 9 0

89711695 T1 5 0

89711949 T3 3 1

89712233 T1 1 0

89712520 T1 14 0

89713039 T1 1 0

89713522 T1 1 0

89713876 T1 1 0

89713931 T4 4 0

89714168 T3 25 0

89714338 T1 6 0

89714391 T1 15 0

89714890 T3 3 1

89714928 T1 14 0

89715027 T1 13 0

89715397 T1 11 0

89715437 T3 10 0

89715687 T1 1 0

89716631 T1 3 0

89717204 T1 18 0

89718094 T1 4 0

89718103 T1 5 0

89718522 T1 2 1

89719541 T1 11 0

89720146 T1 1 0

89720481 T1 14 0

89720487 T1 8 0

89720957 T1 1 0

89721095 T1 12 0

89721143 T1 2 0

89721413 T1 1 0

89721693 T2 2 0

89722429 T2 29 0

89723082 T1 9 0

89723418 T1 11 0

89723453 T1 5 0

89724570 T2 2 0

89724740 T1 5 0

89724937 T1 13 0

89725228 T3 1 1

89725311 T1 11 0

89725388 T1 21 0

89725490 T1 1 0

89725709 T2 4 0

89726025 T1 1 0

89727048 T1 19 0

89727052 T2 2 1

89727113 T1 1 0

89727143 T1 13 1

89727207 T1 2 0

89727225 T1 5 0

89727257 T1 10 0

89727467 T2 17 0

89727534 T1 10 2

89727737 T1 11 0

89727945 T2 28 0

89728656 T1 4 0

89728673 T1 14 0

89728744 T1 4 0

89728784 T1 5 0

89728803 T1 6 0

89728835 T1 10 0

89728837 T1 5 0

89728971 T1 5 0

89729119 T1 5 0

89729237 T1 1 0

89729256 T1 6 0

89729323 T1 3 0

89729592 T1 7 0

89729724 T1 16 0

89730808 T1 8 0

89730888 T1 6 0

89731075 T1 5 0

89731865 T1 3 0

89732521 T1 18 0

89732652 T1 2 0

89732831 T1 23 0

89732892 T1 6 0

89733144 T1 6 0

89733183 T1 19 0

89734266 T1 10 0

89734368 T1 5 0

89734933 T1 3 0

89735050 T1 22 0

89735378 T1 6 0

89735411 T1 4 0

90752339 T1 4 0

90752471 T2 8 0

90752583 T1 5 0

90752586 T1 1 0

90753127 T1 9 0

90753330 T1 11 0

90753378 T1 3 0

90753444 T1 6 0

90753484 T2 19 0

90753674 T2 10 0

90753862 T1 15 0

90754029 T1 2 0

90754714 T2 6 1

90755103 T1 5 0

90755902 T1 4 0

90755948 T1 9 0

90755953 T1 4 0

90756384 T1 29 0

90756972 T1 2 0

90757100 T1 19 0

90757347 T3 1 0

90757742 T1 2 0

90757974 T1 25 0

90758036 T2 2 0

90758129 T1 6 0

90758578 T1 1 0

90759015 T1 16 0

90759156 T1 5 0

90759307 T1 11 0

90759508 T1 7 0

90760592 T1 16 0

90760621 T1 16 0

90760891 T1 9 0

90761132 T1 11 0

90761176 T1 5 0

90761957 T1 16 0

90762254 T1 2 0

90762379 T1 6 0

90762431 T1 23 0

90762724 T1 9 0

90762735 T1 9 0

90763581 T1 16 0

90763853 T3 1 0

90764089 T1 8 0

90764095 T2 10 0

90764437 T1 9 0

90764444 T1 7 0

90765952 T1 2 0

90766014 T1 12 0

90766325 T1 16 0

90766352 T2 14 0

90766892 T1 3 0

90766927 T1 22 0

90767056 T1 1 0

90767370 T1 14 0

90767412 T1 19 0

90768141 T1 6 0

90768354 T2 12 0

90768810 T1 2 0

90769188 T1 13 0

90770172 T1 16 0

90771610 T1 10 0

90771611 T1 6 0

90771715 T1 5 0

90771969 T1 3 0

90772013 T1 5 0

90772430 T3 13 0

90772458 T2 7 0

90772560 T1 11 0

90772812 T1 7 0

90773317 T1 8 0

90773528 T1 3 0

90773633 T1 9 0

90774121 T1 13 0

90774466 T1 3 0

90774554 T2 4 0

90774672 T2 14 0

90774751 T2 20 0

90774835 T1 33 0

90774838 T1 17 0

90774946 T1 14 0

90774984 T1 8 0

90775074 T1 8 0

90775294 T1 1 0

90775429 T1 8 0

90775563 T1 1 0

90775710 T1 5 0

90776106 T1 11 0

90776888 T1 7 0

90777159 T1 6 0

90777163 T1 3 0

90778260 T1 5 0

90778596 T1 11 0

90779050 T1 10 0

90779497 T1 20 0

90779501 T1 3 0

90780021 T3 27 0

90780553 T1 27 0

90780957 T1 36 0

90781084 T2 12 0

90781260 T1 13 0

90782333 T1 21 0

90782602 T2 14 0

90783170 T1 6 0

90783307 T1 19 0

90784180 T2 8 2

90784441 T1 2 0

90784444 T1 4 0

90784655 T1 11 0

90784757 T1 16 0

90784872 T1 21 0

90785368 T1 11 0

90786138 T1 8 0

90786259 T1 4 0

90786536 T1 3 0

90787781 T1 2 0

90787951 T1 8 0

90788063 T1 13 0

90788069 T1 11 0

90788238 T1 8 0

90788282 T1 3 0

90791790 T1 3 0

90792125 T1 6 0

90792998 T2 46 0

90793101 T1 15 0

90793340 T1 20 0

90793478 T1 2 0

90793596 T2 9 0

90793717 T1 1 0

90793884 T1 14 0

90793888 T3 12 0

90794506 T1 5 0

90795351 T3 5 0

90795431 T1 2 0

90795859 T1 1 0

90795903 T1 2 0

90796142 T2 18 1

90796242 T1 4 0

90796461 T1 5 0

90797103 T1 4 0

90797534 T2 2 0

90797674 T1 3 0

90797704 T1 12 0

90797833 T1 11 0

90797882 T1 10 0

90797924 T1 8 0

90798102 T1 22 0

90798610 T1 10 0

90798677 T1 3 0

90798934 T1 19 0

90799061 T1 21 0

90799481 T1 5 0

90799532 T1 1 0

90799762 T1 11 0

90799784 T2 10 0

90799872 T1 12 0

90800198 T2 6 0

90800560 T1 12 0

90800570 T1 5 0

90800852 T1 4 0

90800952 T1 9 0

90801329 T1 1 0

90801599 T1 8 0

90801610 T1 21 0

90801795 T1 3 0

90801876 T1 15 0

90801954 T2 5 0

90802041 T1 5 0

90802937 T3 1 0

90802978 T3 5 0

90803202 T1 3 0

90803216 T3 10 0

90803259 T1 18 0

90803319 T1 10 0

90803390 T1 4 0

90803543 T1 23 0

90803655 T1 2 0

90803752 T2 35 0

90803773 T2 10 0

90803774 T2 11 0

90803916 T3 8 0

90804998 T1 12 2

90805306 T1 11 0

90805431 T2 11 0

90806399 T1 9 0

90807207 T3 17 0

90807450 T1 1 0

90808300 T1 10 0

90808746 T1 19 0

90808893 T1 3 0

90809399 T2 13 0

90809758 T1 6 0

90810151 T1 5 0

90810748 T1 11 0

90810768 T2 12 0

90811305 T1 2 0

90811852 T1 4 0

90812018 T1 30 0

90812838 T1 13 0

90812850 T1 3 0

90813294 T1 6 0

90813725 T1 2 0

90813823 T1 23 0

90813963 T1 30 0

90813980 T1 19 0

90813987 T1 15 0

90814029 T1 2 0

90814321 T1 2 0

90814615 T1 14 0

90815528 T1 2 0

90816029 T1 3 0

90816866 T3 5 2

90816968 T1 15 0

90816975 T1 13 0

90817229 T1 2 0

90817265 T1 8 0

90817408 T1 2 0

90817706 T1 25 0

90818539 T1 2 0

90818626 T1 9 0

90818754 T1 7 0

90818799 T1 4 0

90818956 T1 4 0

90819156 T3 3 0

90819211 T1 9 0

90819426 T3 9 0

90819448 T1 1 0

90820395 T3 26 0

90820454 T1 12 0

90821135 T1 11 1

90821146 T2 11 0

90821187 T2 13 0

90821333 T1 15 0

90821498 T2 6 0

90821737 T1 16 0

90822096 T1 7 0

90822196 T1 5 0

90822219 T1 6 0

90822437 T1 18 0

90822725 T1 18 0

90822873 T1 5 0

90822961 T1 10 0

90823432 T1 14 0

90823911 T1 10 0

90824451 T1 4 0

90824455 T2 5 2

90824512 T1 9 0

90824563 T2 2 0

90824806 T1 1 0

90825183 T1 1 0

90825227 T1 5 0

90825234 T1 10 0

90825847 T1 10 0

90826767 T1 18 0

90826828 T1 4 0

90827333 T1 8 0

90827676 T1 12 0

90827681 T1 5 0

90827902 T3 14 0

90828266 T1 17 0

90828283 T1 17 0

90828308 T1 17 0

90828450 T1 2 0

90828495 T1 2 0

90828553 T2 4 0

90828677 T1 5 1

90828702 T1 4 0

90828847 T1 7 0

90829098 T1 6 0

90829198 T1 6 0

90829354 T1 2 0

90829394 T1 14 0

90829481 T1 12 0

90829646 T1 11 0

90829664 T1 7 0

90829674 T1 9 0

90829709 T1 12 0

90829766 T1 8 0

90829784 T1 8 0

90829860 T1 8 0

90829937 T1 17 0

90829951 T1 9 0

90830040 T1 14 0

90830895 T1 5 0

90831030 T1 11 0

90831059 T3 14 0

90831195 T1 1 0

90831724 T2 17 0

90832282 T1 23 0

90832427 T1 16 0

90833089 T1 4 0

90833678 T1 2 0

90834074 T1 13 0

90834440 T1 2 0

90834855 T1 15 0

90834864 T1 10 0

90835078 T1 21 0

90835347 T2 1 0

90835866 T2 5 0

90836135 T1 14 0

90836160 T1 16 0

90836197 T1 7 0

90836205 T1 9 0

90836294 T1 9 0

90836324 T1 2 0

90836365 T1 12 0

90836508 T2 16 0

90836664 T1 13 0

90836863 T2 15 0

90837788 T1 10 0

90838422 T1 11 0

90838527 T1 10 0

90838730 T3 6 0

90838759 T1 14 0

90838858 T1 16 0

90838881 T1 2 0

90838944 T1 6 0

90838975 T1 7 0

90838980 T1 10 0

90839001 T1 4 0

90839077 T1 3 0

90839080 T1 2 0

90839235 T1 9 0

90839311 T1 21 0

90839371 T2 6 0

90839537 T1 13 0

90839610 T1 1 0

90839697 T1 4 0

90839770 T1 8 0

90839886 T1 12 3

90840006 T1 15 0

90840085 T1 14 0

90841154 T1 16 0

90841541 T3 10 0

90841592 T1 18 0

90842061 T1 7 0

90842508 T2 14 0

90842573 T1 2 0

90842960 T1 7 0

90843388 T1 6 0

90843596 T2 4 0

90843677 T1 3 0

90843892 T1 1 0

90843984 T1 2 0

90844151 T2 14 0

90844524 T1 9 0

90844999 T1 8 0

90846055 T1 10 0

90846554 T3 9 0

90846557 T2 16 0

90846683 T1 3 0

90846829 T1 7 0

90847384 T1 3 0

90850370 T1 1 0

90851322 T1 1 0

90851676 T1 3 0

90851711 T1 3 0

90852542 T1 3 0

90852870 T1 15 0

90853044 T1 2 0

90853327 T1 5 0

90853400 T1 16 0

90853463 T2 11 0

90853466 T1 11 0

90853468 T1 5 0

90853771 T1 14 0

90854093 T1 12 0

90855115 T1 6 0

90856179 T1 24 0

90856218 T1 4 0

90857285 T3 19 0

90857439 T2 44 0

90857528 T1 18 0

90857592 T1 33 0

90858057 T1 7 0

90858259 T3 13 0

90858429 T1 7 0

90858644 T1 9 0

90859446 T1 5 0

90859546 T1 11 0

90859746 T2 2 1

90860325 T1 3 0

90860643 T2 2 0

90860793 T1 8 0

90861014 T1 12 0

90861041 T1 6 0

90861103 T1 13 1

90861512 T1 8 0

90861660 T1 1 0

90861749 T1 6 0

90861762 T1 4 0

90861787 T1 9 0

90861885 T1 2 0

90862093 T1 1 0

90862404 T1 12 0

90862515 T1 7 0

90862659 T1 8 0

90862696 T1 6 0

90862730 T1 5 0

90862743 T1 5 0

90862839 T1 14 0

90862917 T1 10 0

90864869 T1 34 3

90865124 T2 29 0

90865250 T2 1 0

90865306 T1 2 0

90865569 T1 46 0

90865824 T1 23 2

90865884 T1 25 0

90865892 T1 34 0

90866261 T1 12 0

90866679 T1 1 0

90866703 T1 15 0

90866715 T1 6 0

90866927 T1 6 0

90867048 T1 16 0

90867139 T3 13 2

90867334 T1 8 0

90867602 T2 2 0

90868189 T1 10 0

90868665 T2 13 0

90868876 T1 27 0

90869099 T1 12 0

90869131 T1 8 0

90869151 T1 2 2

90869179 T1 13 0

90869268 T1 6 0

90869367 T1 4 0

90869572 T1 4 0

90869588 T1 6 0

90869649 T1 5 0

90870241 T1 3 0

90870773 T1 23 0

90870774 T1 9 1

90870831 T1 9 0

90871201 T1 2 0

90871340 T1 2 0

90871603 T1 7 0

90871714 T1 12 0

90871963 T3 1 0

90871992 T1 10 0

90872332 T1 13 0

90872353 T1 4 0

90872639 T1 7 0

90872726 T1 5 0

90872764 T1 22 0

90872834 T2 12 0

90872980 T1 4 0

90873089 T1 16 0

90873138 T2 8 0

90873313 T2 5 0

90873379 T1 10 0

90873405 T1 9 0

90873540 T1 14 0

90873746 T1 17 0

90873757 T4 8 0

90873813 T1 28 0

90873823 T1 29 0

90873868 T1 22 0

90874337 T1 8 0

90874425 T1 26 0

90874501 T2 3 0

90875331 T1 11 0

90875721 T2 21 11

90876121 T1 12 0

90877888 T1 14 0

90877987 T1 5 0

90878028 T1 10 0

90878581 T3 16 0

90878733 T3 13 0

90878882 T1 19 0

90878913 T1 5 0

90879252 T2 16 4

90879322 T1 4 0

90880077 T1 6 0

90880324 T1 49 0

90880474 T1 15 0

90880629 T1 4 0

90880639 T1 2 0

90880890 T1 14 0

90880943 T1 9 0

90881059 T2 2 0

90881122 T1 13 0

90881125 T1 11 0

90881156 T2 17 0

90881348 T3 7 0

90881730 T1 11 0

90881747 T1 7 0

90881803 T1 10 0

90881828 T2 11 2

90881837 T1 4 0

90881841 T1 4 0

90882448 T1 9 0

90883026 T1 7 0

90883029 T1 18 0

90883528 T1 5 0

90883674 T1 6 0

90883860 T2 1 0

90883947 T1 30 0

90884081 T1 1 0

90884241 T1 2 0

90884268 T2 9 0

90884393 T1 1 0

90884825 T1 12 0

90885138 T1 11 1

90885162 T1 14 0

90885328 T1 2 0

90885540 T1 12 0

90885738 T1 18 0

90885836 T1 21 0

90885858 T1 13 0

90885974 T1 2 0

90886179 T1 14 0

90886249 T1 9 0

90886709 T1 15 0

90887013 T1 9 0

90887259 T2 13 0

90888060 T2 37 11

90888088 T2 16 0

90888396 T3 8 1

90888523 T1 28 0

90888645 T1 6 0

90888990 T1 1 0

90889027 T1 1 0

90889120 T1 1 0

90889380 T1 30 0

90889723 T1 3 0

90889975 T2 23 0

90890372 T3 11 0

90890723 T2 8 0

90892447 T2 38 4

90892463 T1 15 0

90892473 T1 18 0

90892566 T1 5 0

90892761 T1 1 0

90893304 T2 10 0

90894135 T1 3 1

90894640 T1 7 0

90894911 T1 4 0

90894965 T1 9 0

90895003 T1 11 0

90895049 T2 10 0

90895152 T1 4 2

90895213 T1 7 0

90895480 T1 4 0

90895510 T2 17 1

90896085 T1 10 0

90896114 T3 2 0

90896372 T1 5 0

90896637 T2 16 0

90897216 T1 2 1

90897939 T1 10 0

90897957 T3 8 0

90898010 T2 9 0

90898109 T1 15 0

90898378 T1 1 0

90898426 T1 14 0

90899344 T1 7 0

90900964 T1 7 0

90902034 T1 2 0

90902230 T1 15 0

90902500 T3 5 0

90902751 T1 9 0

90902763 T1 1 0

90903065 T2 33 0

90903511 T1 14 3

90903850 T1 26 0

90904197 T1 13 0

90904318 T1 1 0

90904574 T1 13 0

90904598 T1 20 0

90904628 T2 10 0

90904705 T2 3 0

90904760 T1 1 0

90905248 T2 4 0

90905546 T1 2 0

90905822 T1 3 0

90906298 T1 24 0

90906520 T1 7 0

90906917 T1 7 0

90907002 T1 2 0

90907053 T1 11 0

90907563 T1 9 0

90907683 T1 15 0

90907766 T1 15 0

90907933 T1 7 0

90908033 T1 7 0

90908192 T1 11 0

90908402 T1 13 0

90908403 T1 3 0

90908526 T1 17 0

90909396 T1 10 0

90909573 T1 1 0

90910153 T1 12 0

90910236 T1 9 3

90910649 T1 10 0

90910762 T1 7 0

90910859 T1 12 0

90911244 T1 1 1

90911269 T1 6 0

90911597 T1 3 0

90912122 T1 15 0

90912123 T2 15 1

90912326 T1 16 0

90912341 T1 1 0

90912634 T1 7 0

90912771 T1 5 0

90914449 T1 5 0

90914622 T1 9 0

90914923 T1 6 0

90915416 T1 17 0

90915426 T1 10 0

90915899 T2 14 0

90916179 T1 16 0

90917961 T1 12 0

90918163 T1 16 0

90918572 T1 7 0

90918598 T1 24 0

90920101 T1 10 0

90920609 T1 6 0

90920674 T1 2 0

90920800 T1 7 0

90921033 T2 6 0

90921578 T3 13 0

90921619 T2 11 1

90921705 T1 21 0

90923806 T1 9 0

90923878 T1 7 0

90923939 T1 11 0

90924077 T2 56 0

90925034 T2 5 0

90925209 T1 15 0

90925330 T1 26 0

90925443 T1 30 0

90925546 T1 28 0

90925586 T1 13 0

90925696 T1 25 0

90925846 T1 20 0

90926197 T1 5 0

90926210 T1 19 0

90926215 T1 27 0

90926447 T1 14 0

90927460 T1 25 0

90930479 T4 44 0

90930925 T1 6 0

90930945 T2 7 0

90931016 T1 8 0

90931344 T3 2 0

90931423 T1 19 0

90931424 T1 12 0

90931425 T1 18 0

90931446 T1 10 0

90931620 T1 2 0

90931775 T1 17 0

90932254 T1 21 0

90932292 T1 17 0

90932306 T1 1 0

90932677 T1 8 0

90933449 T1 18 0

90933461 T1 1 0

90933743 T1 25 0

90933941 T1 13 0

90934347 T1 8 0

90934438 T1 1 0

90934639 T1 10 0

90934642 T1 1 0

90935053 T1 3 0

90936150 T3 6 0

90936461 T1 14 0

90936827 T1 14 0

90936882 T3 1 0

90937536 T1 2 0

90938093 T1 6 0

90938685 T1 2 0

90938726 T1 9 0

90939058 T3 1 0

90939130 T3 2 0

90939735 T2 1 0

90940137 T2 14 0

90940176 T1 10 0

90940476 T1 4 0

90940490 T2 10 0

90940550 T1 1 0

90940553 T1 6 0

90940677 T1 7 0

90940680 T1 2 0

90940926 T1 9 0

90940949 T3 6 0

90941012 T1 14 0

90941062 T2 7 0

90941105 T1 9 0

90941185 T1 10 0

90941496 T1 19 0

90942355 T2 17 0

90942370 T2 1 0

90942962 T1 6 0

90943074 T1 8 0

90943179 T1 2 0

90943188 T1 22 0

90943377 T1 9 0

90943409 T1 15 0

90943498 T1 1 0

90944027 T1 14 0

90944135 T1 7 0

90944191 T1 5 0

90944332 T1 17 0

90944594 T2 17 0

90945078 T1 6 0

90946416 T1 13 0

90946430 T1 2 0

90947035 T1 10 0

90947210 T1 13 0

90947607 T1 2 0

90947623 T3 17 0

90947941 T2 14 0

90948095 T1 5 2

90948506 T1 12 1

90948935 T1 49 0

90949026 T1 6 0

90949209 T3 10 0

90949756 T1 7 0

90949947 T1 9 0

90949963 T1 13 0

90949969 T1 21 0

90949992 T1 10 0

90950012 T1 7 0

90950019 T3 23 0

90950071 T1 10 0

90950124 T1 4 0

90950185 T3 23 8

90950245 T1 7 0

90950259 T1 4 0

90950273 T2 21 0

90950291 T1 16 1

90950351 T1 5 0

90950387 T1 20 0

90950405 T3 21 0

90950468 T1 5 0

90950488 T1 8 0

90950544 T3 1 0

90950633 T1 11 0

90950690 T1 9 0

90950715 T1 17 1

90950755 T3 5 0

90951064 T1 3 0

90951494 T1 2 0

90951650 T1 7 0

90952232 T1 13 0

90952355 T1 5 0

90952429 T1 20 0

90952501 T3 7 2

90952545 T1 11 0

90952574 T1 10 0

90952841 T1 24 0

90952844 T2 11 0

90952852 T1 10 0

90952899 T1 8 0

90952931 T1 15 0

90953008 T1 21 0

90953040 T1 5 0

90953109 T1 8 0

90953209 T1 7 0

90953387 T1 11 0

90953435 T1 12 0

90953946 T1 7 0

90953976 T1 12 0

90954147 T1 6 0

90954311 T1 23 0

90954397 T1 3 0

90954523 T1 15 0

90954552 T1 5 0

90954685 T1 13 0

90954784 T4 10 0

90954859 T1 16 0

90954876 T1 12 0

90954890 T2 16 0

90954944 T2 17 0

90955029 T1 6 0

90955033 T1 7 0

90955344 T2 25 0

90955504 T1 2 0

90955660 T1 5 0

90955970 T1 14 0

90956091 T1 4 0

90956195 T1 11 0

90956202 T1 22 0

90956350 T1 4 0

90956618 T1 4 0

90957074 T1 1 0

90957125 T3 1 0

90957947 T1 15 0

90958324 T1 16 0

90960317 T1 6 0

90960514 T1 9 0

90961207 T1 4 0

90961495 T1 19 0

90961523 T1 17 0

90961525 T1 19 0

90961564 T1 29 0

90961639 T1 18 0

90961643 T1 10 0

90961666 T1 26 0

90961713 T1 10 0

90961719 T1 3 0

90961758 T1 18 0

90961933 T1 3 0

90962937 T1 40 0

90963710 T1 4 0

90964390 T1 17 0

90965085 T1 21 0

90965198 T1 4 0

90965676 T1 18 0

90965713 T1 4 0

90966359 T1 14 0

90966376 T1 19 0

90966379 T1 13 0

90966653 T1 18 0

90966707 T1 6 0

90966891 T1 10 0

90967180 T1 15 0

90967480 T4 17 0

90967506 T3 7 0

90967594 T2 2 0

90968381 T1 8 0

90969106 T1 8 0

90969118 T1 9 0

90969454 T1 8 0

90969480 T1 1 0

90969975 T2 9 0

90970150 T1 9 0

90970312 T1 16 0

90970363 T2 16 0

90970597 T1 33 0

90970855 T1 34 0

90971897 T2 1 1

90971975 T1 5 0

90972063 T1 2 0

90972105 T1 10 0

90972303 T1 27 0

90972741 T1 4 0

90973067 T1 5 0

90973787 T3 6 0

90973907 T1 12 0

90974810 T1 25 0

90974913 T1 16 0

90974950 T1 14 0

90974972 T1 3 0

90975050 T1 3 0

90975093 T1 9 0

90976174 T1 2 0

90976281 T1 16 0

90976288 T1 11 0

90976470 T1 22 1

90976491 T1 6 0

90976678 T1 18 0

90977807 T1 4 0

90977956 T1 4 0

90977973 T1 7 0

90978070 T1 6 0

90978369 T1 20 0

90978373 T1 12 0

90978385 T1 13 0

90978626 T1 5 0

90978636 T1 22 0

90978806 T1 16 0

90979185 T1 1 0

90979440 T1 15 0

90979470 T1 7 0

90979586 T1 6 0

90979833 T1 14 0

90979892 T1 10 0

90980080 T1 8 0

90980213 T1 17 0

90980320 T1 5 0

90980555 T1 3 0

90980574 T1 22 0

90980658 T1 7 0

90980809 T1 12 0

90980812 T1 8 0

90980814 T1 4 0

90980826 T1 7 0

90980854 T2 6 0

90981030 T1 8 0

90981031 T1 12 0

90981223 T1 10 0

90981328 T1 4 0

90981387 T2 12 0

90981451 T1 12 0

90981614 T1 30 0

90981692 T1 5 0

90982334 T1 8 0

90983232 T1 1 0

90984089 T1 16 0

90984256 T1 33 0

90984348 T1 6 0

90984718 T2 20 0

90985053 T3 54 0

90985524 T1 16 1

90985886 T1 8 0

90985888 T2 17 0

90986314 T2 17 0

90986575 T1 31 0

90986762 T1 15 0

90986766 T1 12 0

90987323 T1 4 0

90987350 T1 26 0

90988092 T1 4 0

90988171 T1 3 0

92000069 T1 27 0

92000098 T1 16 0

92000128 T1 18 0

92000130 T1 19 0

92000154 T2 29 0

92000180 T1 9 0

92000222 T3 24 0

92000738 T1 2 0

92001129 T1 12 0

92001854 T3 10 0

92002078 T3 11 0

92002110 T1 4 0

92002293 T1 19 0

92002298 T2 11 0

92002724 T1 1 0

92002742 T2 29 1

92002816 T1 1 0

92002852 T1 9 0

92003420 T1 10 0

92003491 T1 26 0

92003499 T3 16 0

92004105 T1 8 0

92004316 T1 2 0

92004553 T1 6 0

92004562 T1 8 0

92005277 T3 6 0

92005597 T1 2 0

92005738 T1 4 0

92005860 T1 3 0

92005928 T2 15 0

92006698 T2 20 0

92006730 T1 4 0

92006853 T1 19 0

92006891 T1 18 0

92006940 T1 24 0

92006961 T1 16 0

92006963 T1 22 0

92006998 T1 13 1

92007165 T1 5 0

92007229 T2 13 0

92007397 T1 14 0

92007408 T1 2 0

92007526 T2 2 0

92007562 T1 20 0

92008212 T1 2 0

92009418 T1 22 0

92010149 T1 13 0

92010377 T1 2 0

92010467 T1 20 0

92010482 T1 10 0

92010526 T1 11 0

92010642 T1 5 0

92010670 T1 14 0

92010789 T1 8 0

92010843 T1 11 0

92011023 T1 1 0

92011063 T1 7 0

92011335 T1 7 0

92012508 T1 6 0

92012537 T1 16 0

92012577 T1 3 0

92012592 T1 4 0

92012687 T2 22 2

92012725 T1 10 0

92012751 T2 8 0

92012807 T1 18 0

92012873 T1 3 0

92013892 T1 3 0

92014168 T1 11 0

92014699 T2 6 0

92014750 T1 7 0

92014938 T1 8 0

92015291 T1 8 0

92015825 T1 18 0

92015918 T1 6 0

92016228 T3 3 0

92016772 T1 12 0

92016812 T4 8 0

92016850 T1 11 0

92016885 T1 21 0

92016955 T1 13 0

92017741 T1 5 0

92018174 T1 15 0

92018558 T1 6 0

92019126 T1 4 0

92019344 T3 1 0

92019359 T3 2 0

92019770 T1 2 0

92020534 T1 47 0

92021176 T2 17 0

92021550 T1 7 0

92022058 T4 9 1

92022277 T1 6 0

92022350 T1 16 0

92022420 T1 4 0

92022422 T2 13 2

92024524 T1 15 0

92024600 T1 3 0

92024667 T1 2 0

92024682 T1 18 0

92024687 T1 12 0

92024747 T1 2 0

92024761 T1 6 0

92024837 T1 2 0

92025040 T1 4 0

92025222 T1 32 0

92025249 T1 18 0

92025289 T1 20 0

92025413 T1 6 0

92025530 T1 2 0

92026055 T1 17 0

92026251 T1 14 0

92026688 T1 6 0

92026805 T2 15 0

92027058 T2 37 0

92027257 T1 7 0

92028056 T1 22 0

92028203 T1 5 0

92028495 T3 22 0

92028499 T1 22 1

92028519 T1 17 0

92028596 T1 9 0

92029372 T1 5 0

92030139 T1 8 0

92030151 T1 10 2

92030435 T1 1 0

92030475 T1 3 0

92031647 T1 22 3

92035874 T1 3 0

92036783 T1 7 0

92038369 T1 3 0

92040042 T1 6 0

92040152 T1 15 0

92040180 T1 26 0

92040226 T1 32 0

92040307 T1 7 0

92040345 T1 2 0

92040798 T1 50 0

92041521 T3 6 1

92041698 T1 6 0

92041967 T1 6 0

92044172 T1 4 0

92049410 T1 4 0

92053086 T1 11 0

92055795 T1 15 0

92055862 T1 14 0

92056782 T2 7 0

92058795 T3 15 0

92089449 T1 5 0

92126925 T1 2 0

97017749 T1 20 0

97018870 T1 5 0

97019718 T1 19 1

97020723 T1 6 0

97020742 T2 11 0

97021270 T1 7 0

97021721 T1 15 0

97021887 T1 21 0

97021969 T1 4 0

97022084 T1 8 0

97022390 T1 1 0

97022442 T3 32 0

97022620 T1 8 0

97023974 T2 4 1

97024245 T1 1 0

97024508 T1 7 0

97024573 T1 5 0

97025626 T1 12 0

97025717 T1 2 0

97026016 T1 2 0

97026224 T1 32 0

97026229 T2 3 0

97026236 T1 4 0

97027320 T1 7 0

97027411 T1 14 0

97027562 T1 14 0

97027615 T2 22 0

97029134 T3 9 0

97029181 T1 23 1

97029485 T1 5 0

97029691 T1 2 0

97030062 T1 10 0

97030065 T1 26 0

97030575 T1 6 0

97031147 T1 7 0

97031905 T1 1 0

97032373 T2 13 0

97033139 T1 15 0

97033576 T1 25 0

97033793 T1 15 0

97034100 T1 6 0

97035178 T1 1 0

97035948 T1 6 0

97036631 T1 17 2

97036896 T1 5 0

97036986 T1 3 0

97037598 T1 48 0

97037696 T1 5 0

97038175 T1 7 0

97038317 T1 1 0

97039088 T1 1 0

97039264 T1 18 0

97039746 T1 11 0

97039752 T1 9 3

97039920 T2 15 4

97039923 T2 23 0

97040094 T1 21 0

97040327 T1 9 0

97040380 T1 1 1

97040969 T2 3 0

97041218 T1 16 0

97041244 T1 7 0

97041847 T3 8 0

97041858 T1 3 0

97042417 T1 8 0

97042624 T1 8 0

97042668 T1 10 0

97042678 T1 13 0

97043039 T1 2 0

97043063 T1 7 0

97044016 T1 14 0

97044943 T1 2 0

97044946 T1 4 0

97044985 T1 3 0

97045346 T1 11 0

97045787 T1 13 0

97046018 T1 21 0

97046595 T1 9 0

97046883 T1 2 0

97047409 T2 4 0

97048739 T4 6 1

97048759 T1 8 0

97049847 T1 3 0

97050586 T4 9 4

97050889 T1 8 0

97050977 T3 1 0

97051187 T1 33 0

97051203 T1 8 0

97051204 T1 4 0

97051622 T1 5 0

97053290 T3 20 1

97053297 T1 5 0

97053309 T2 3 0

97053311 T1 7 0

97053319 T1 18 0

97053404 T1 3 0

97053690 T1 26 0

97053788 T1 3 0

97054043 T1 22 0

97054047 T1 6 0

97054335 T3 7 0

97054368 T1 5 0

97054710 T1 12 0

97054746 T1 8 0

97054849 T1 13 0

97055177 T1 13 0

97055690 T1 5 0

97056791 T1 5 0

97056823 T1 7 0

97056941 T1 6 0

97057425 T1 9 0

97057431 T1 12 0

97057512 T1 17 0

97057584 T1 6 0

97057740 T1 2 0

97057954 T1 5 0

97058080 T1 11 0

97058086 T1 25 4

97058100 T1 9 0

97058264 T1 11 0

97059125 T1 7 0

97059157 T1 5 0

97059176 T1 23 0

97059214 T3 7 1

97060177 T1 12 0

97060659 T1 18 0

97060871 T3 10 0

97060990 T1 19 0

97062632 T1 3 0

97063772 T1 3 0

97063778 T1 9 0

97064502 T1 10 0

97064709 T1 7 0

97065739 T1 7 0

97066377 T1 10 0

97066692 T1 16 0

97067488 T1 15 0

97067882 T1 6 0

97067994 T1 3 0

97068247 T1 19 0

97068264 T1 14 0

97068419 T1 2 0

97068429 T1 2 0

97069680 T1 5 0

97069739 T1 3 0

97069844 T1 16 0

97070097 T1 10 0

97070150 T1 5 0

97070210 T1 23 0

97070418 T1 6 0

97070883 T1 23 0

97072057 T3 7 0

97072061 T3 17 1

97072137 T1 19 0

97072866 T1 7 0

97073211 T2 46 0

97073896 T3 4 0

97074135 T1 11 0

97074424 T2 8 0

97074524 T1 5 0

97078277 T1 3 0

97078331 T1 17 0

97078379 T1 10 0

97078645 T2 1 0

97079082 T1 14 6

97079085 T1 16 0

97079151 T1 2 0

97079173 T1 4 0

97080775 T3 6 0

97081306 T1 20 0

97081895 T1 12 0

97082649 T1 9 0

97082936 T1 8 0

97083043 T1 1 0

97083526 T1 3 0

97083771 T1 5 0

97085197 T1 15 0

97085213 T1 2 0

97085468 T1 18 1

97085525 T1 7 0

97087059 T3 5 0

97087259 T2 11 0

97087625 T1 5 0

97088921 T1 26 0

97089035 T2 3 0

97089089 T1 2 0

97089136 T1 6 0

97089182 T1 5 0

97089478 T1 17 0

97089482 T1 6 0

97090225 T1 3 0

97090433 T1 11 0

97090440 T1 23 0

97090933 T2 1 0

97091105 T1 9 0

97091413 T1 10 0

97091497 T1 9 0

97091785 T1 3 0

97091863 T1 1 0

97091917 T1 3 0

97092167 T1 2 0

97092391 T1 21 0

97092874 T1 6 2

97094026 T2 26 0

97094498 T2 26 0

97094611 T2 7 0

97094757 T1 26 0

97094927 T1 4 0

97094936 T1 2 0

97095021 T1 20 0

97095385 T2 20 2

97095539 T2 16 1

97095935 T2 40 0

97096106 T1 2 0

97096180 T1 5 0

97096552 T2 22 0

97096862 T1 4 0

97097207 T1 16 0

97098336 T1 12 0

97098384 T1 10 0

97098454 T1 9 2

97098794 T1 26 0

97098796 T1 18 0

97099688 T1 7 0

97099934 T1 9 0

97100054 T2 8 0

97100412 T1 10 0

97100869 T2 8 0

97101094 T1 1 0

97101829 T1 16 0

97103263 T1 2 0

97103530 T1 32 0

97104452 T1 9 0

97106605 T1 10 0

97106774 T1 21 0

97106930 T2 15 0

97107340 T1 1 0

97107349 T1 8 0

97107576 T1 6 0

97107981 T1 10 0

97108674 T1 12 0

97108783 T1 5 0

97109337 T2 11 0

97109735 T1 25 0

97110047 T1 4 0

97110330 T1 5 0

97110633 T1 1 0

97111458 T1 4 0

97111628 T1 3 0

97112536 T1 17 0

97112875 T1 2 0

97112889 T2 12 0

97112890 T1 19 0

97113027 T1 5 0

97113546 T1 1 1

97114007 T1 2 0

97114453 T1 9 0

97114480 T2 1 0

97114529 T1 11 0

97114635 T1 28 0

97114708 T1 4 0

97114712 T1 4 0

97114722 T1 7 0

97114723 T1 1 0

97114989 T1 21 0

97115108 T1 29 0

97115140 T1 3 0

97115235 T1 13 0

97115375 T1 11 0

97115759 T1 4 0

97115841 T1 17 0

97115853 T1 23 0

97116023 T3 2 0

97117064 T2 5 0

97117093 T2 8 0

97118173 T2 5 0

97118189 T2 16 0

97118794 T1 15 0

97118980 T1 6 0

97119063 T1 9 0

97119066 T1 8 0

97119186 T1 16 0

97119794 T2 16 0

97120167 T1 3 0

97120466 T1 10 0

97120688 T2 8 0

97120703 T1 10 0

97120748 T1 16 0

97120993 T1 15 0

97121006 T1 17 0

97121026 T1 21 0

97121106 T1 14 0

97121111 T1 4 0

97121180 T1 32 0

97121903 T1 7 0

97122433 T1 15 1

97123400 T1 13 0

97123443 T1 7 0

97123666 T3 5 1

97124258 T1 13 0

97125564 T1 32 0

97125701 T1 2 0

97126097 T3 3 0

97126883 T1 8 0

97126960 T1 10 0

97127238 T3 13 0

97127385 T1 3 0

97127641 T1 4 0

97127891 T1 15 0

97128100 T1 6 0

97128512 T1 22 0

97128978 T1 15 0

97129016 T1 14 0

97129091 T3 16 1

97129141 T1 10 0

97129343 T1 12 0

97129612 T1 11 0

97130267 T1 4 0

97130651 T1 29 0

97131527 T1 7 0

97131880 T1 10 0

97132053 T1 6 0

97133531 T3 7 0

97133550 T1 11 0

97133706 T1 16 0

97134513 T1 16 0

97134535 T1 5 0

97134861 T1 8 0

97135146 T1 2 0

97135271 T1 12 0

97135828 T1 5 0

97135966 T1 6 0

97135994 T1 8 0

97136011 T1 15 0

97136066 T1 16 0

97136792 T1 35 0

97136807 T1 6 0

97136890 T1 11 0

97139545 T3 17 0

97139593 T2 3 0

97139631 T1 7 0

97140875 T2 2 0

97141276 T2 2 1

97142742 T1 12 0

97143545 T3 2 0

97144311 T1 15 0

97144467 T1 11 0

97144827 T2 10 0

97145060 T1 9 0

97146619 T1 17 0

97147045 T1 17 0

97147199 T1 7 0

97147988 T1 12 0

97148058 T1 1 0

97148246 T1 2 0

97148903 T1 24 0

97150048 T1 4 0

97150388 T1 21 0

97150677 T1 18 0

97151332 T4 5 0

97152036 T1 4 0

97152635 T1 8 0

97152880 T1 2 0

97152946 T2 2 0

97153351 T1 10 0

97153353 T1 7 0

97153809 T2 15 0

97154136 T1 6 0

97154358 T1 5 0

97154372 T1 8 0

97154718 T1 15 0

97155204 T1 14 0

97155486 T1 6 0

97156270 T1 3 0

97156362 T2 9 1

97156472 T1 7 0

97156921 T1 7 0

97157243 T1 10 0

97158243 T1 10 0

97158795 T1 1 0

97158881 T1 7 0

97159192 T1 3 0

97159258 T1 2 0

97159654 T1 4 0

97160168 T1 6 0

97160614 T1 1 0

97160737 T1 3 0

97161130 T1 13 0

97161205 T1 9 0

97161276 T1 17 0

97161787 T1 17 12

97161903 T1 6 0

97162396 T2 5 0

97162421 T1 7 0

97162540 T1 18 0

97162572 T1 5 0

97162754 T4 7 0

97163012 T1 11 0

97163196 T1 8 0

97163420 T1 8 0

97163820 T1 3 0

97165175 T1 2 0

97165354 T1 3 0

97166099 T3 7 0

97166411 T1 3 2

97166709 T1 1 0

97166779 T2 8 0

97167048 T1 2 0

97167679 T1 8 0

97167833 T1 5 0

97169168 T1 9 0

97169464 T1 8 0

97169653 T3 11 0

97169817 T1 6 0

97170034 T1 27 0

97170241 T2 14 0

97170541 T1 1 0

97170650 T2 6 0

97170658 T1 3 0

97170749 T2 3 0

97170803 T3 4 0

97171207 T1 4 0

97171735 T1 12 0

97172242 T1 2 0

97172340 T1 14 0

97172489 T1 30 0

97172750 T1 15 1

97172886 T1 2 0

97173333 T1 2 0

97173881 T1 7 0

97173903 T3 12 0

97174065 T1 22 0

97174470 T1 7 0

97174652 T1 11 0

97174812 T1 9 0

97174830 T1 4 0

97174874 T1 2 0

97174886 T1 7 0

97175131 T2 4 0

97175214 T1 5 0

97175406 T1 10 0

97175555 T1 11 0

97175712 T1 25 0

97176468 T1 3 0

97176560 T1 11 0

97176917 T1 13 0

97176979 T1 2 0

97177081 T1 3 0

97177100 T3 10 2

97177112 T1 2 0

97177115 T1 7 0

97177182 T1 8 0

97177291 T1 12 0

97177295 T1 14 0

97177440 T1 29 0

97177867 T1 4 0

97177988 T2 4 0

97178031 T1 11 0

97178671 T1 50 1

97179000 T1 4 0

97179527 T1 12 0

97179805 T1 15 0

97180233 T1 9 0

97181276 T1 18 0

97181846 T2 7 0

97182367 T1 5 0

97182370 T1 2 0

97182399 T1 17 0

97183183 T1 10 0

97184354 T1 5 0

97186088 T1 15 0

97186363 T1 4 0

97188854 T1 5 0

97189130 T1 9 0

97189678 T2 11 0

97189692 T1 13 0

97189990 T1 15 0

97190546 T1 2 0

97190682 T1 10 0

97191634 T1 8 0

97193524 T1 12 0

97193629 T1 5 0

97194115 T1 18 0

97750035 T1 4 0

97750454 T1 11 0

97755682 T1 15 0

97755721 T1 21 0

97755728 T1 11 0

97756542 T1 2 0

97758046 T1 6 0

97758757 T1 13 0

97760409 T1 10 0

97762721 T1 25 0

97765958 T1 7 0

97767864 T1 10 0

97767909 T1 9 0

97769139 T1 2 0

97776477 T2 19 0

97776480 T3 23 2

97781036 T1 17 0

97781094 T3 18 0

97782523 T1 29 0

97786214 T1 13 0

97786219 T1 10 0

97787521 T2 15 0

97788801 T1 7 0

97797748 T1 5 0

97798486 T2 1 0

97803520 T1 14 0

97863508 T1 26 0

97863542 T2 17 0

97863562 T1 5 0

97863569 T1 13 0

97863619 T2 14 0

97863794 T1 23 0

97863824 T1 17 0

97864006 T1 13 0

97864045 T1 18 0

97864081 T1 10 0

97864083 T1 9 0

97871963 T1 12 0

889 T2 48 0

3644 T2 11 2

4330 T1 13 0

6635 T3 8 3

8262 T1 4 0

1030015 T1 1 0

1750935 T1 1 0

1753639 T2 17 1

1771269 T1 1 0

1780263 T2 15 0

1829214 T2 3 0

1830725 T3 1 1

1835932 T1 1 0

1839034 T1 12 0

1840173 T1 2 1

1843129 T1 2 0

1843449 T1 1 0

1850025 T1 13 0

1853022 T1 10 0

1853684 T1 22 0

1854043 T1 5 0

1857493 T1 17 0

1859560 T1 2 0

1866852 T1 4 0

1868124 T3 4 1

1873188 T4 12 0

1889454 T3 7 0

1890219 T1 9 0

1891209 T1 9 0

1891951 T4 2 1

1894527 T1 52 6

1895933 T1 2 0

1896377 T1 12 1

1897420 T1 9 0

1898222 T2 6 0

1899501 T3 6 1

1899740 T1 1 0

1899823 T2 4 1

1899872 T1 8 0

1900549 T1 1 0

1902482 T2 28 2

1911034 T1 3 0

1911118 T1 1 0

1914389 T1 1 0

2627474 T1 17 0

2631317 T1 12 0

2632976 T1 21 0

2634293 T1 11 0

2634800 T1 9 0

2636760 T1 1 0

2639273 T1 3 0

2644158 T1 3 0

2644515 T3 13 0

2649232 T1 11 1

2654260 T3 21 8

2656880 T4 11 0

2662669 T3 12 0

2665015 T3 90 0

2669436 T1 11 2

2672185 T1 2 0

2672288 T1 12 0

2674733 T1 10 0

2680011 T1 3 0

2688111 T1 25 0

2688942 T1 26 0

2691954 T1 2 0

2694345 T3 4 0

2701145 T2 17 0

2701735 T2 19 0

2706202 T1 44 0

2707558 T3 1 0

2708262 T1 34 0

2711172 T2 17 0

2712202 T1 10 0

2712946 T1 21 0

2714173 T2 9 2

2715908 T2 1 0

2729675 T3 21 0

2731053 T2 31 0

2731266 T1 5 0

2731946 T2 1 0

2732818 T1 5 0

2737100 T1 6 0

2740207 T1 41 0

2742015 T2 1 0

2747163 T2 10 0

2748642 T2 14 0

2750237 T1 4 0

2751158 T2 9 0

2753460 T1 5 0

2758258 T4 31 0

2758649 T1 1 0

2761027 T2 22 0

2763351 T1 2 0

2766951 T1 3 0

2768788 T3 90 0

2769895 T3 10 0

2771070 T1 16 0

2771400 T2 27 1

2773432 T1 23 0

2773613 T1 6 0

2779338 T1 17 0

2784312 T1 26 0

2785039 T1 13 0

2787481 T1 19 0

2793321 T1 22 0

3505842 T1 5 0

3508800 T3 26 1

3509844 T3 30 0

3509879 T3 9 2

3511881 T1 6 0

3511893 T1 3 0

3513177 T1 12 0

3516320 T3 11 0

3516933 T2 5 0

3517163 T3 16 2

3517644 T2 36 1

3519811 T1 39 0

3521268 T1 18 0

3523178 T1 26 0

3524555 T1 6 0

3526295 T1 6 0

3526828 T1 17 0

3528236 T1 20 0

3528443 T1 3 0

3528908 T4 1 0

3529072 T3 10 0

3530249 T3 32 3

3530268 T1 2 0

3531056 T1 12 0

3532240 T3 2 0

3533726 T1 8 1

3534138 T1 10 0

3534147 T2 25 1

3536493 T1 13 0

3536810 T3 8 2

3536924 T3 11 5

3544071 T1 5 0

3545278 T1 11 0

3545565 T1 11 0

3545985 T1 7 0

3548489 T3 13 0

3548849 T3 5 2

3551166 T1 18 0

3556594 T1 22 0

3557722 T1 6 0

3558105 T1 8 0

3560517 T3 8 0

3561562 T3 10 0

3562936 T2 45 0

3563320 T3 26 0

3564819 T1 16 0

3573251 T1 7 0

3577003 T1 8 0

3577059 T1 2 0

3577214 T2 22 0

3577893 T1 13 0

3578779 T3 14 4

3580562 T3 4 0

3583093 T1 14 0

3589353 T3 12 0

3591924 T2 10 0

3592646 T3 15 0

3593268 T3 18 0

3593820 T3 20 5

3594256 T3 6 0

3594684 T2 3 0

3594715 T1 6 0

3596278 T2 8 0

3597466 T1 2 0

3597632 T2 2 0

3600075 T3 2 2

3601539 T1 24 0

3601572 T2 23 0

3601697 T4 22 5

3601835 T1 4 0

3601855 T3 2 0

3603342 T1 2 0

3603856 T1 2 0

3604320 T2 31 0

3604533 T3 28 0

3608173 T1 11 0

3612022 T1 29 0

3612606 T4 1 1

3614413 T2 16 0

3615000 T3 2 0

3616550 T3 23 0

3622072 T1 14 0

3622239 T4 1 0

3622860 T1 9 1

3623385 T1 15 0

3624038 T1 9 0

3624044 T1 6 0

3626435 T1 28 0

3626733 T2 2 2

3629384 T3 22 10

3630684 T1 20 0

3633218 T1 1 0

3634926 T1 17 0

3635252 T1 4 1

3635854 T1 12 0

3641928 T1 19 0

3646140 T1 2 1

3646676 T1 23 3

3652370 T1 7 0

3652499 T3 21 3

3652790 T1 52 0

3654918 T1 7 0

3655886 T1 6 0

3656639 T3 12 0

3656728 T3 2 0

3659594 T1 21 0

3660518 T1 1 0

3662040 T1 6 0

3664808 T1 28 0

3664974 T3 27 6

3664982 T3 33 0

3665450 T1 4 0

3666799 T2 5 0

3666959 T3 1 0

3670709 T4 24 2

3670805 T1 12 0

3670814 T1 4 0

4378151 T4 10 0

4380353 T1 4 0

4380537 T3 1 1

4380579 T2 15 0

4380587 T1 5 0

4381330 T2 24 0

4387034 T2 1 0

4389270 T1 8 0

4389492 T1 7 0

4389955 T1 27 0

4392196 T1 12 1

4394001 T1 16 0

4394916 T2 27 0

4396059 T3 28 0

4401769 T1 5 0

4401893 T3 34 2

4402394 T1 7 0

4402588 T3 8 0

4405345 T2 3 1

4406174 T3 13 8

4406244 T1 20 0

4407016 T1 57 1

4407200 T1 6 1

4411298 T3 7 0

4412101 T4 11 0

4413702 T4 16 0

4415851 T1 9 0

4416790 T3 42 0

4417544 T2 7 0

4418768 T2 21 0

4420003 T1 2 0

4421354 T1 22 0

4421520 T1 32 1

4428139 T2 18 1

4429127 T3 43 12

4435573 T1 51 0

4435828 T1 12 0

4442114 T2 42 11

4443179 T1 8 0

4445598 T1 35 0

4451811 T2 9 2

4456816 T1 31 0

4459177 T2 6 2

4459509 T1 36 0

4459938 T1 11 0

4459943 T1 13 0

4460016 T1 28 0

4461059 T1 7 0

4461170 T2 19 0

4461494 T1 3 0

4461678 T4 10 0

4461714 T1 19 2

4461756 T2 11 3

4462113 T3 14 4

4462553 T1 24 0

4463021 T3 13 1

4463713 T1 40 0

4463804 T1 19 0

4463995 T1 28 0

4464738 T2 11 0

4464853 T3 5 0

4469456 T2 55 0

4471312 T1 3 0

4474987 T2 27 0

4475889 T1 46 0

4476386 T3 1 0

4476453 T3 9 1

4476509 T2 30 1

4477344 T2 35 0

4477415 T3 1 0

4477425 T1 23 0

4477943 T1 10 0

4477955 T3 6 0

4478068 T1 21 0

4478199 T1 1 0

4478408 T1 19 0

4478588 T2 14 0

4478617 T1 10 0

4478937 T3 6 2

4479235 T3 19 0

4479241 T2 26 1

4479339 T1 12 0

4479351 T4 2 0

4480637 T2 6 1

4482432 T1 18 1

4487018 T1 29 0

8341412 T1 1 0

8342695 T2 2 0

8344303 T1 5 0

8349361 T1 3 0

8354218 T2 13 0

8363542 T2 2 1

8367072 T1 14 0

8368851 T1 1 0

8369352 T1 13 0

8369410 T3 15 3

8370219 T1 1 0

8370244 T1 9 0

8370670 T2 1 0

8371924 T1 10 0

8372564 T4 4 0

8374707 T1 7 0

8379226 T2 4 4

8379699 T1 3 0

8381537 T1 1 0

8382168 T1 1 0

9338521 T2 2 0

9341768 T1 3 0

9346650 T1 2 0

9349963 T2 11 1

9350183 T2 28 0

9352135 T1 1 0

9352863 T1 2 0

9353066 T1 1 0

9353205 T2 10 0

9360298 T1 9 0

9362845 T3 23 0

9366049 T1 3 0

9368831 T1 1 1

9370360 T1 2 0

9377044 T1 1 0

9380500 T1 3 0

9380609 T1 9 0

9380872 T1 1 0

9381389 T1 1 0

9382427 T1 3 0

9386674 T3 6 2

9386902 T1 3 0

9392546 T1 13 0

9395935 T1 6 0

9398299 T1 4 0

9405124 T1 11 0

9407730 T3 13 6

9409921 T1 9 0

9412330 T1 3 0

9415416 T2 2 0

9417872 T1 1 1

9425449 T1 1 0

9425876 T1 12 0

9426731 T2 16 0

9427168 T1 1 0

9428356 T1 4 0

9436307 T1 3 0

9437717 T3 8 0

9438293 T1 23 0

9440851 T1 6 0

9447092 T1 35 0

9451156 T1 2 0

9451675 T1 1 0

9456252 T1 2 0

9456256 T2 15 0

9456992 T3 8 0

9462715 T3 2 0

9463267 T1 9 0

9463327 T1 16 0

9466343 T1 3 0

9466355 T1 6 0

9474547 T1 1 0

9476600 T1 17 0

9480594 T2 4 0

9480605 T2 8 4

9481508 T2 5 0

9486151 T1 31 0

9488962 T1 1 1

9489935 T2 3 0

9489958 T1 2 0

9491195 T1 8 1

9493205 T1 1 1

9494048 T1 6 0

9494909 T1 6 4

9496750 T2 7 0

9498813 T1 5 0

9500732 T1 3 0

9503984 T1 22 0

9507709 T2 6 2

9507712 T3 1 0

9509579 T1 14 0

9511617 T1 5 0

9511625 T1 15 0

9514142 T1 2 0

9516068 T1 20 0

9520684 T1 3 0

9520801 T3 4 0

9524028 T1 11 0

9528369 T3 1 1

9528921 T3 3 0

9528940 T3 9 5

9531294 T1 4 0

9531976 T3 2 2

9539723 T1 1 0

9543812 T3 6 0

9543843 T1 17 3

9544215 T4 19 0

10500110 T1 2 0

10501031 T1 1 0

10503425 T1 5 1

10503505 T2 8 0

10503798 T3 1 1

10504141 T2 10 0

10504952 T1 6 0

10505008 T3 7 0

10505686 T1 7 0

10505755 T1 12 0

10505941 T2 2 0

10509960 T1 29 0

10511855 T1 4 0

10512008 T1 3 0

10513421 T1 20 0

10513955 T1 9 0

10514297 T2 24 2

10515943 T3 2 1

10517682 T1 5 2

10518004 T1 13 0

10521311 T1 13 0

10521761 T3 6 0

10522066 T1 16 1

10524972 T1 9 0

10525807 T1 13 0

10527850 T2 21 0

10528959 T1 8 0

10529094 T2 3 1

10535438 T1 17 3

10535734 T1 32 0

10535739 T2 5 0

10535812 T3 3 1

10537409 T2 19 0

10541220 T2 15 0

10541477 T3 3 1

10541644 T1 12 0

10543367 T1 14 0

10547856 T1 9 5

10548336 T1 11 1

10549619 T1 5 0

10551201 T1 3 0

10551240 T1 12 0

10552612 T1 9 0

10561092 T1 13 0

10561411 T1 18 3

10562253 T1 6 0

10564326 T1 20 5

10565717 T3 18 3

10566074 T1 7 0

10572431 T1 25 0

10572774 T2 17 4

10577188 T3 6 3

10578611 T1 13 0

10580635 T2 3 0

10582083 T2 11 4

10582657 T1 11 4

10582813 T3 5 0

10583872 T1 8 0

10584520 T3 5 0

10585092 T3 1 0

10586917 T3 22 1

10586984 T3 5 1

10589340 T3 2 0

10589569 T1 24 0

10590190 T1 9 0

10590957 T1 9 1

10592949 T1 15 1

10593200 T2 4 0

10594027 T1 3 0

10594160 T1 11 0

10598448 T1 13 0

10599253 T2 9 0

10601021 T3 10 1

10601275 T1 12 0

10605249 T1 4 0

10606542 T1 11 0

10607517 T4 1 0

10608938 T3 22 1

10612051 T3 32 1

10612105 T1 11 0

10613769 T1 1 0

10614602 T2 8 7

10614778 T3 16 0

10616395 T2 9 0

10619169 T2 1 0

10620485 T3 34 6

10621479 T1 13 0

10621737 T1 16 0

10624595 T2 19 0

10627489 T1 13 0

10628923 T4 1 1

10630995 T1 38 1

10632495 T1 7 0

10635268 T1 1 0

10639442 T1 4 0

10639659 T3 1 0

10644258 T3 12 6

10644620 T4 12 0

10644739 T2 17 0

10648963 T3 15 1

10653561 T3 16 0

10654550 T2 12 1

10654951 T1 16 0

10655246 T3 1 0

10655349 T3 7 0

10655783 T4 1 0

10656686 T1 4 0

10657559 T1 5 0

10657817 T1 21 0

10658799 T2 19 0

10659358 T1 13 0

10659461 T1 3 0

10660447 T2 28 0

10660593 T1 13 1

10660797 T2 25 0

10661940 T3 5 3

10665233 T3 2 0

10666109 T1 7 0

10667359 T2 3 1

10672516 T1 20 0

10673004 T2 17 0

10673171 T1 28 0

10673192 T2 16 0

10674318 T4 8 0

10677704 T3 16 0

10678230 T2 27 0

10678635 T3 29 0

10679289 T4 28 1

10682211 T1 17 4

10682540 T3 2 0

10683243 T2 9 1

10683280 T3 6 3

10684863 T1 22 0

10686291 T3 9 2

10686691 T1 1 0

10689047 T1 12 0

10690141 T2 13 1

10692095 T1 9 0

10692138 T4 10 0

10692561 T1 11 0

10694244 T2 5 0

10695821 T2 21 0

10696141 T1 6 0

10696368 T2 20 1

10697410 T1 25 0

10698531 T1 9 0

10699346 T1 12 0

10701340 T3 7 1

10702237 T2 45 0

10702595 T3 17 0

10704210 T1 31 0

10705237 T2 14 0

10705610 T1 2 1

10705731 T1 11 1

10707805 T1 7 0

10709793 T1 12 0

10711187 T3 6 0

10713027 T1 11 0

11668823 T1 51 5

11671542 T3 8 5

11671759 T3 27 0

11672337 T3 4 0

11674496 T1 19 0

11674615 T4 24 0

11677929 T1 12 0

11680169 T1 15 0

11681680 T3 35 0

11682707 T3 11 0

11683445 T1 21 0

11683592 T1 8 0

11687444 T1 33 0

11687497 T1 23 0

11688850 T1 21 0

11689001 T1 22 1

11691207 T3 4 2

11691741 T3 13 4

11693214 T1 11 0

11695605 T1 28 0

11703262 T3 3 0

11703894 T3 27 4

11705416 T3 4 3

11707713 T1 16 0

11708628 T1 13 0

11709800 T2 4 0

11713400 T1 37 0

11714193 T1 17 0

11714297 T3 22 0

11715251 T2 9 0

11719925 T3 16 0

11720855 T1 12 0

11721383 T3 2 1

11723623 T3 27 0

11724435 T2 30 0

11724941 T3 28 0

11725545 T1 20 0

11727222 T1 23 1

11728028 T3 18 3

11728236 T3 7 0

11728562 T3 3 1

11730046 T1 9 0

11733575 T4 19 1

11746356 T1 19 0

11761601 T1 11 0

11761808 T2 13 3

11806822 T1 19 0

12939993 T2 2 0

15240342 T3 1 0

15353097 T3 4 1

15370323 T3 1 0

15371051 T1 1 0

15381881 T1 11 0

15382814 T3 4 0

15384866 T1 3 1

15385450 T1 2 0

15390205 T1 1 0

15392196 T3 32 0

15395181 T1 3 0

15399496 T2 2 0

15407063 T2 1 0

15407523 T2 4 0

15410552 T3 1 1

15411388 T1 11 0

15412733 T1 9 0

16333491 T2 22 0

16333612 T3 10 3

16334521 T1 3 0

16334561 T1 15 0

16334760 T1 6 0

16336002 T1 7 0

16339783 T1 2 0

16339811 T1 10 0

16341389 T2 5 0

16342893 T1 3 0

16346529 T3 9 0

16347114 T2 5 0

16347818 T1 25 0

16348233 T1 7 0

16349633 T3 21 0

16353415 T1 3 0

16364361 T2 12 0

16365201 T1 18 0

16365569 T2 6 2

16365574 T1 2 2

16365909 T1 10 0

16366564 T1 28 0

16369261 T1 2 0

16369360 T1 10 0

16370159 T2 8 0

16370359 T1 6 0

16371562 T1 5 1

16371939 T1 8 0

16372295 T1 17 0

16373285 T3 8 0

16376866 T3 24 2

16379340 T2 8 1

16382260 T4 6 4

16388462 T3 8 0

16388888 T2 3 0

16391678 T1 1 0

16393862 T1 8 0

16400944 T2 21 0

16400960 T2 17 2

16401777 T3 1 0

16402768 T1 10 0

16402771 T1 65 3

16404488 T1 8 0

16407522 T1 8 2

16413997 T1 2 0

16414005 T1 7 0

16418914 T1 4 0

16423191 T1 11 0

16424877 T1 9 1

16430908 T1 23 0

16433276 T1 12 0

16437161 T1 11 0

16438890 T1 10 0

16439091 T1 19 0

16439501 T1 6 0

16440106 T1 11 0

16441277 T2 15 4

16441751 T1 7 0

16444012 T3 7 0

16445891 T3 16 0

16445930 T1 15 0

16449665 T1 37 0

16456197 T1 4 0

16456677 T3 19 0

16459197 T2 3 0

16460180 T3 2 2

16460209 T4 15 0

16461180 T3 14 2

16461192 T1 7 0

16461859 T1 3 0

16461866 T2 3 0

16461896 T1 11 0

16464737 T2 25 0

16464993 T1 14 0

16465430 T3 1 1

16466021 T2 7 5

16466860 T1 6 0

16476662 T1 7 0

16478392 T2 4 0

16479032 T1 8 0

16481664 T3 1 1

16483325 T2 19 0

16485935 T4 1 0

16485949 T1 5 0

16486131 T1 13 0

16487338 T2 7 0

16491054 T2 9 0

16492997 T3 5 0

16494135 T1 7 0

16496195 T1 10 0

16496392 T1 25 0

16497869 T2 18 0

16502259 T1 7 0

16505888 T1 5 4

16508729 T3 2 0

16516913 T1 2 0

16516921 T1 18 0

16517853 T1 8 0

16518514 T2 1 0

16520698 T2 11 0

16524939 T4 3 2

16525429 T1 1 0

16527710 T3 29 25

16529935 T2 7 0

16530176 T1 1 0

16535995 T1 23 1

16535997 T2 5 0

16536020 T1 8 0

16536622 T3 4 3

16540232 T2 21 0

16541404 T3 2 0

16541743 T2 8 1

16544444 T3 6 0

16544611 T1 16 0

16548077 T1 23 1

16552762 T1 3 0

16552834 T1 44 0

16564682 T2 4 0

16569391 T1 3 0

16570966 T1 3 0

16574201 T1 1 0

16574607 T1 12 1

16576213 T2 25 0

17500051 T1 21 0

17503371 T1 3 0

17503605 T1 6 0

17506000 T1 9 0

17507186 T1 18 0

17507202 T2 4 0

17507496 T1 3 0

17508799 T3 19 2

17510502 T1 15 0

17511185 T2 6 0

17512348 T1 3 0

17517809 T2 25 0

17518882 T3 4 0

17519130 T3 1 1

17520337 T1 4 0

17523751 T1 7 1

17529592 T1 14 1

17529894 T2 4 0

17531867 T1 11 0

17533323 T1 12 0

17541196 T2 7 3

17545225 T3 2 1

17545286 T1 16 0

17546283 T2 13 0

17547179 T3 17 2

17548615 T3 1 1

17548882 T1 5 0

17549120 T1 25 0

17550446 T2 5 0

17550460 T1 7 0

17550874 T1 1 0

17551151 T2 38 0

17552012 T1 17 0

17552199 T1 2 0

17552562 T2 5 0

17558899 T1 5 0

17561791 T2 19 3

17562144 T2 10 0

17563086 T1 3 0

17568384 T1 1 0

17569409 T2 11 0

17570029 T3 5 0

17571692 T2 2 0

17575039 T2 19 1

17575054 T2 16 1

17577061 T1 5 0

17580198 T2 10 0

17581213 T3 5 0

17581227 T2 22 0

17585482 T1 21 5

17586012 T4 1 1

17586044 T1 12 0

17588497 T1 2 0

17594269 T1 28 0

17595546 T1 17 1

17596630 T2 3 0

17600284 T1 11 1

17601436 T4 15 4

17602194 T3 10 0

17602427 T1 20 0

17604856 T2 10 0

17605801 T1 12 0

17605811 T2 4 0

17606560 T1 16 0

17608010 T1 13 1

17609232 T2 8 0

17610637 T3 11 0

17611593 T1 15 0

17611749 T3 17 0

17613451 T1 6 0

17614248 T3 9 0

17614255 T2 10 0

17614805 T1 12 0

17617488 T1 4 0

17617876 T3 6 0

17618735 T1 6 0

17621313 T4 20 0

17625289 T3 15 5

17625326 T2 19 4

17627565 T2 22 0

17627588 T1 5 0

17630593 T4 1 0

17630601 T2 15 0

17630602 T1 11 0

17632253 T1 10 0

17632921 T2 14 2

17634351 T2 14 0

17634352 T1 10 0

17634507 T1 37 0

17635390 T1 17 0

17637982 T1 23 1

17639661 T1 24 9

17640830 T3 10 0

17640835 T3 5 0

17645530 T3 1 1

17645632 T2 20 0

17646262 T1 6 0

17646269 T1 2 0

17646272 T1 10 0

17648653 T1 17 0

17648663 T2 11 3

17648675 T3 12 0

17650551 T1 37 0

17650559 T1 38 5

17650565 T3 2 0

17650574 T3 19 0

17651485 T1 5 0

17651865 T2 2 0

17653420 T2 17 0

17653752 T2 2 0

17653780 T1 3 0

17655627 T1 2 0

17657972 T3 12 1

17658224 T1 6 0

17659823 T1 11 0

17659830 T1 7 2

17661248 T2 13 0

17662349 T1 7 0

17663841 T3 5 0

17664988 T3 1 0

17666467 T1 9 0

17666848 T2 7 0

17668375 T1 20 0

17668839 T1 4 0

17671357 T4 6 0

17671392 T2 16 0

17672184 T2 20 0

17675385 T2 8 0

17676496 T1 21 0

17677628 T1 10 0

17678750 T3 41 0

17683134 T1 10 1

17687060 T4 28 12

17689465 T1 10 0

17690495 T3 18 5

17690497 T1 6 0

17692008 T1 1 0

17692012 T2 10 0

17697774 T2 27 10

17697782 T2 20 1

17699761 T2 3 0

17700582 T2 17 0

17700897 T3 1 1

17700915 T3 15 1

17704443 T1 13 0

17708937 T1 13 0

17709079 T3 4 0

17710632 T2 31 0

17710642 T3 10 0

17718444 T1 16 0

17718448 T3 15 1

17718455 T1 17 0

17718463 T3 22 2

17724745 T1 28 0

17725117 T1 2 2

17727366 T1 7 2

17727390 T1 17 0

17730726 T1 20 0

17731419 T1 9 0

17731429 T2 5 0

17732021 T3 15 0

17734145 T2 7 0

17734149 T1 9 0

17734850 T3 4 0

17738029 T1 12 0

17738713 T2 23 0

17738840 T3 6 6

17739602 T1 6 0

17739896 T1 27 0

17740985 T1 9 2

17741922 T2 18 0

17743874 T1 8 0

17744420 T1 1 1

18668690 T3 19 1

18674215 T2 14 0

18674250 T1 12 0

18674279 T2 20 0

18674349 T3 10 0

18674406 T3 13 0

18674441 T2 6 0

18676742 T2 9 1

18679441 T1 4 2

18680137 T2 3 1

18680794 T2 25 0

18682922 T3 7 0

18684241 T2 2 0

18684356 T3 13 0

18686621 T1 34 1

18686993 T3 22 0

18687003 T3 1 0

18687905 T1 14 0

18688570 T2 4 0

18688877 T3 20 0

18690852 T3 18 0

18691630 T3 32 0

18692066 T2 5 0

18692080 T2 26 0

18692592 T1 6 0

18693719 T1 13 0

18693789 T3 3 0

18693844 T1 3 0

18693988 T1 20 0

18694722 T2 12 0

18700298 T3 1 1

18702606 T1 15 0

18705528 T1 1 0

18705553 T3 7 0

18708659 T3 6 0

18711719 T1 7 0

18712957 T2 15 5

18713305 T1 18 3

18715368 T2 12 0

18715723 T1 21 1

18715726 T3 15 0

18716725 T1 9 0

18717362 T3 5 0

18719410 T3 36 1

18720857 T1 23 0

18721547 T3 5 1

18723403 T1 11 0

18725079 T3 6 1

18728501 T1 3 0

18728502 T1 1 0

18728503 T3 19 3

18728964 T1 23 0

18728971 T3 3 1

18729410 T3 22 5

18729747 T3 28 0

18729767 T2 5 0

18729994 T1 12 0

18731012 T1 24 0

18734013 T2 2 0

18735236 T1 3 0

18736643 T1 1 0

18739257 T2 3 0

18740737 T3 6 2

18742106 T1 22 0

18742189 T3 9 4

18742800 T1 45 0

18746404 T1 31 0

18747335 T2 9 3

18749391 T1 8 0

18751223 T2 18 0

18755827 T1 4 0

18756523 T2 12 0

18760513 T1 11 0

21088409 T3 6 0

21092752 T1 2 0

21098082 T1 1 0

21098394 T1 4 0

21098897 T1 16 0

21099811 T1 14 0

21104836 T1 15 0

21105170 T3 2 1

21106260 T2 9 1

21111393 T1 5 0

21112354 T1 3 0

21118785 T1 2 0

21123538 T2 6 0

21123635 T1 3 0

21125148 T1 8 0

21126904 T1 6 0

21131614 T1 10 0

21133097 T1 7 0

21133960 T1 8 2

21140595 T1 9 0

21143808 T1 12 0

21144124 T3 24 1

21146128 T1 5 3

21147312 T1 5 0

21147910 T1 4 0

21149352 T3 19 5

21150522 T1 6 0

21152406 T1 9 1

21154204 T1 16 0

21154899 T3 7 0

21155372 T1 2 0

21155435 T1 16 0

21155480 T1 7 0

21158502 T1 1 0

21158858 T1 3 2

21159143 T1 4 0

21159394 T3 1 0

21161616 T3 6 0

21162569 T1 8 0

21164826 T3 2 0

21167485 T1 5 0

21168176 T3 16 0

21169002 T2 2 1

21169324 T1 3 0

21169668 T1 8 0

21171924 T1 1 0

22002002 T3 1 0

22002163 T3 5 0

22002855 T1 2 0

22004315 T3 5 0

22008219 T3 27 16

22011090 T1 10 0

22012982 T3 4 2

22014901 T2 15 0

22017832 T1 20 0

22018982 T1 4 0

22020726 T2 26 1

22023733 T3 4 0

22029027 T1 1 0

22030165 T3 3 0

22031614 T3 2 2

22037298 T1 1 0

22038621 T1 4 0

22038823 T2 21 6

22038930 T3 2 1

22041185 T1 11 0

22041666 T1 53 0

22045547 T1 5 0

22048038 T3 14 0

22048910 T1 23 0

22048968 T3 17 0

22049866 T1 14 0

24314686 T3 1 0

24322766 T3 1 1

24325247 T2 2 0

24326457 T1 1 0

24326521 T1 12 0

24331177 T1 2 0

24331203 T1 7 0

24331440 T1 9 0

24335730 T1 1 0

24337740 T1 4 0

24338121 T1 1 0

24341437 T2 2 0

24341600 T1 9 0

24342685 T1 1 0

24343886 T1 2 0

24343906 T1 2 0

24345031 T2 5 1

24353850 T1 2 0

24353854 T3 2 0

24353855 T1 2 0

24357155 T1 4 0

24358081 T1 46 1

24359305 T1 3 0

24361346 T2 4 0

24363529 T3 1 0

24363819 T1 6 0

24369995 T1 10 1

24374260 T2 4 0

24375760 T1 10 0

24375811 T1 12 1

24376679 T2 42 0

24377277 T1 2 0

24380628 T2 12 0

24381429 T1 2 0

24381733 T3 2 0

24381908 T1 15 0

24382043 T1 4 1

24382521 T1 1 0

24393498 T1 12 0

24394358 T1 11 0

24397797 T1 3 2

24399410 T2 2 0

24401186 T2 2 0

24407826 T1 6 2

24409041 T2 6 0

24412845 T1 10 0

25401715 T1 26 0

25402388 T1 1 0

25403233 T2 2 0

25403234 T1 13 4

25405070 T2 5 0

25407016 T1 1 0

25408169 T1 9 0

25419529 T3 1 0

25419721 T1 1 0

25422591 T2 2 0

25423546 T1 1 0

25424245 T1 8 3

25426127 T1 3 0

25426196 T2 10 0

25426495 T1 2 0

25439238 T1 8 0

25440148 T1 4 0

25445442 T2 3 0

25445854 T3 1 0

25447966 T2 15 0

25449512 T1 4 0

25452961 T2 27 3

25453963 T2 2 0

25454079 T3 5 0

25454335 T1 10 0

25457136 T1 5 0

25461837 T1 2 0

25462934 T1 18 0

25467357 T1 11 0

25467498 T2 20 1

25467638 T1 7 0

25468814 T1 10 0

25471965 T1 7 0

25472260 T1 6 0

25473595 T1 14 0

25478025 T1 4 0

25478126 T1 3 0

25483611 T1 3 0

25493866 T1 4 4

25495680 T1 11 3

25495870 T3 3 0

25497044 T1 4 0

25499558 T1 49 0

25502519 T1 1 0

25503824 T1 23 0

25506626 T1 6 0

25507819 T1 2 0

25508023 T2 12 1

25508743 T1 7 0

25510387 T3 2 0

25510738 T1 2 0

25511927 T1 1 0

25520346 T2 9 2

25520651 T3 4 2

25522523 T1 2 0

25523397 T3 1 1

25528003 T2 63 0

25528493 T1 8 0

25528954 T3 4 0

25529358 T2 4 0

25532581 T1 3 0

25533095 T1 32 0

25533783 T1 2 0

25533882 T1 17 0

25537918 T3 2 1

25539414 T1 10 0

25543204 T1 1 0

25546771 T1 8 0

25547059 T3 4 2

25547641 T2 31 0

25548933 T1 8 0

25551225 T1 4 0

25551681 T1 2 0

25551853 T1 10 0

25552539 T1 4 0

25554289 T1 7 0

25558383 T4 40 0

25558839 T1 1 0

25560883 T2 10 0

25580023 T1 3 0

25582676 T2 4 2

25584637 T1 1 0

25585896 T2 3 0

25587178 T1 11 0

25587810 T1 2 0

25588090 T1 50 0

25589098 T1 11 0

25589481 T2 18 3

25589694 T1 9 0

25590289 T1 6 0

25592506 T1 38 1

25593051 T1 3 0

25593889 T3 2 0

25597617 T2 2 0

25598362 T2 7 0

25600702 T2 16 0

25600944 T1 16 0

25603682 T1 5 0

25604368 T4 1 1

25606504 T1 11 0

25611017 T1 15 0

25611130 T1 13 0

25611594 T3 45 0

25613686 T4 4 0

25615372 T1 3 0

25615724 T3 3 0

25616507 T2 10 0

25617557 T3 27 0

25617597 T4 1 0

26600228 T3 5 1

26601642 T2 14 0

26604047 T3 1 1

26608685 T1 7 0

26609351 T1 30 4

26610971 T1 12 0

26611003 T2 12 0

26611848 T2 7 1

26613074 T1 17 0

26619486 T2 2 0

26620264 T1 41 3

26624015 T2 12 0

26624521 T3 2 0

26624926 T1 2 0

26626044 T1 8 0

26632254 T1 5 0

26633620 T1 7 0

26636732 T1 23 0

26637656 T1 7 0

26640846 T1 11 0

26641583 T1 2 0

26643738 T1 12 0

26646548 T3 13 0

26648137 T2 7 1

26648669 T1 14 2

26650378 T2 23 0

26652960 T3 4 0

26654188 T1 1 0

26654766 T4 1 0

26654909 T2 2 0

26655002 T3 6 3

26655481 T1 21 0

26656168 T1 49 0

26656268 T1 10 0

26656845 T3 3 0

26658806 T1 3 0

26658927 T3 37 0

26661056 T2 34 0

26663870 T4 3 0

26666396 T1 2 0

26666534 T2 22 0

26666733 T1 21 0

26666789 T1 7 0

26667789 T1 3 0

26669796 T4 2 2

26671424 T3 4 0

26671444 T1 15 0

26671559 T3 4 0

26674862 T1 8 0

26677705 T3 26 2

26678933 T1 11 1

26680151 T4 5 1

26680517 T2 12 3

26682731 T1 24 0

26684135 T2 7 1

26685218 T1 10 0

26688277 T1 4 0

26691321 T3 3 2

26697068 T2 3 0

26699405 T1 51 1

26699435 T3 5 0

26704615 T2 9 0

26709578 T1 15 0

26709649 T3 13 1

26711594 T1 8 0

26713622 T2 8 0

26714456 T1 20 0

26715126 T1 2 0

26715293 T1 20 0

26716669 T3 2 0

26716934 T1 1 0

26718700 T3 34 8

26719436 T1 24 0

26728810 T3 1 0

26732182 T1 6 0

26732463 T3 3 0

26737842 T1 21 1

26739512 T1 18 0

26753187 T1 18 0

29111764 T1 1 0

29111902 T2 3 0

29112755 T1 40 0

29113702 T3 20 0

29114352 T1 3 0

29122437 T1 4 0

29122850 T1 16 0

29125077 T2 4 2

29130583 T3 16 0

29131640 T3 1 0

29132248 T1 1 0

29132432 T2 3 0

29142708 T3 5 2

29147262 T2 5 0

29147270 T1 3 0

29148632 T3 1 0

29155055 T1 3 0

29162827 T2 13 0

29163207 T1 15 5

29164947 T1 2 0

29165787 T1 1 0

29165868 T2 7 5

29169375 T1 1 0

29170325 T1 13 0

29175086 T2 6 1

29176540 T2 4 3

29178129 T1 7 0

29178744 T1 2 0

29180196 T1 4 0

29180804 T3 6 1

29185487 T2 4 2

29185950 T2 3 1

29186250 T2 17 0

29186745 T1 7 1

29192804 T1 11 7

29196623 T1 1 0

29196963 T1 1 0

29197069 T1 1 0

29199764 T1 5 1

29203120 T2 10 0

29203127 T1 6 0

29206355 T1 8 1

29206566 T1 9 4

29207635 T3 4 0

29217738 T3 7 0

29217825 T3 2 0

29221295 T2 1 0

29229344 T3 4 0

29232386 T1 4 0

29233134 T3 1 1

29236052 T1 1 0

29237058 T1 4 0

29240623 T3 19 0

30000233 T2 18 0

30000570 T3 2 0

30000575 T2 22 1

30001431 T3 1 0

30004105 T3 17 3

30005521 T2 20 0

30006862 T3 2 1

30007683 T1 1 0

30014591 T2 17 0

30014739 T1 15 0

30016315 T1 10 0

30016728 T1 2 0

30022662 T4 1 0

30022720 T2 17 6

30023512 T2 1 0

30024329 T1 26 0

30028565 T1 3 0

30030129 T1 6 0

30030441 T3 14 0

30032355 T1 4 0

30034247 T3 2 0

30038443 T1 1 0

30043718 T3 19 4

30044888 T1 1 0

30047762 T2 15 0

30048952 T1 15 0

30049490 T3 5 0

30051103 T1 11 0

30055869 T1 26 1

30055872 T1 14 0

30059985 T1 32 0

30060824 T4 7 1

30065790 T2 11 3

30071254 T3 28 4

30075312 T3 16 3

32243273 T2 4 2

32255612 T1 4 0

32256322 T1 13 0

32260175 T1 6 0

32262899 T2 12 0

32270642 T1 22 0

32271585 T2 19 0

32271989 T1 14 0

32272549 T2 26 0

32276146 T1 14 0

32276305 T2 21 4

32276310 T3 6 0

32277614 T1 9 0

32278695 T1 22 0

32280278 T1 9 0

32281771 T1 22 0

32283116 T4 1 0

32291348 T1 1 0

32291351 T2 3 0

32291498 T1 8 0

32291499 T1 1 0

32291647 T1 2 0

32292166 T1 23 0

32293889 T1 2 0

32296355 T1 20 0

32302585 T1 2 0

32302867 T1 26 0

32303288 T1 30 0

32303439 T1 3 0

32307960 T1 6 0

32314468 T1 3 1

32316678 T4 4 4

32317773 T1 16 0

32321497 T1 1 0

32324416 T1 11 0

32325366 T1 4 0

32326365 T3 6 3

32334103 T1 3 0

32336822 T1 17 0

32336906 T2 5 0

32340368 T1 3 0

32341963 T1 3 0

32343143 T2 2 0

32344943 T1 18 0

32345216 T1 34 0

32345801 T1 10 0

32349601 T1 14 2

32354397 T1 3 0

32355712 T1 19 0

32356022 T3 37 0

32361224 T1 10 0

32366553 T1 24 1

32366750 T1 27 0

32368344 T2 11 0

32373229 T1 19 0

32374040 T1 16 1

33339594 T1 8 0

33340793 T1 14 0

33341901 T1 18 0

33345540 T2 27 0

33351236 T1 1 0

33352450 T1 1 0

33354739 T1 14 0

33358270 T1 8 0

33359134 T1 20 2

33362882 T1 26 0

33366737 T1 15 0

33371966 T1 24 0

33374245 T1 7 0

33375629 T2 11 1

33379189 T1 11 0

33381385 T1 18 0

33384309 T1 35 0

33386656 T1 10 0

33386999 T2 9 0

33389257 T2 3 0

33393494 T1 17 0

33394970 T2 18 0

33396976 T1 19 0

33398778 T2 8 0

33399670 T1 8 0

33400852 T1 25 0

33402742 T2 29 20

33403480 T1 21 0

33406911 T1 33 0

33407052 T1 12 0

33410850 T2 1 0

33410935 T1 12 0

33411103 T1 14 0

33412300 T1 5 0

33416267 T1 23 0

33419468 T3 7 5

33420649 T1 11 4

33422813 T1 11 0

33422890 T3 1 1

33426014 T3 18 0

33427346 T4 5 3

33430397 T2 2 2

33433240 T1 49 1

33433582 T1 15 1

33433941 T1 10 1

33434583 T1 14 0

33434625 T3 1 0

33437217 T3 2 0

33438837 T2 22 0

33440483 T1 15 0

33440742 T3 6 0

33442303 T1 1 0

33443816 T2 2 0

33444642 T1 1 0

33444956 T1 11 0

33447100 T1 8 0

33449036 T1 6 1

33450056 T1 5 0

33451567 T1 13 0

33452582 T1 24 0

33453596 T1 10 0

33456155 T1 30 0

33461174 T3 1 0

33461376 T1 10 0

33462075 T1 3 1

33466309 T1 9 0

33471763 T2 36 0

33475781 T1 13 0

33475969 T2 18 0

33477816 T2 25 0

33479414 T1 16 1

33481323 T1 8 0

33486264 T3 19 6

33487034 T1 5 0

33490137 T1 16 1

33493118 T1 16 2

33493940 T1 35 0

33495027 T1 29 0

33496162 T1 10 0

33496657 T1 9 0

33499502 T1 5 0

33500201 T1 11 0

33501089 T2 22 0

33502855 T1 4 0

33503226 T3 2 1

33504037 T1 19 0

33505181 T1 12 1

33509204 T1 8 0

33511957 T1 29 1

33513395 T3 2 1

33518884 T3 5 3

33519294 T1 29 0

33520427 T3 25 0

33522726 T1 32 0

33525225 T2 25 3

33527025 T2 13 0

33527136 T2 15 11

33529996 T2 2 0

33533918 T1 6 0

33534088 T1 2 0

33535146 T1 27 0

33536982 T2 13 0

33538287 T2 3 0

33538422 T2 28 0

33542913 T2 1 0

33545593 T3 41 6

34500770 T1 5 0

34502923 T2 16 0

34503035 T2 28 0

34504999 T1 19 0

34505181 T1 14 0

34506247 T1 10 0

34506343 T2 4 0

34507403 T2 14 4

34507983 T1 18 0

34508992 T1 16 0

34509260 T1 32 0

34511530 T1 23 3

34512789 T1 4 0

34513539 T2 17 0

34513629 T1 2 0

34518274 T3 9 0

34520390 T4 2 2

34521084 T1 37 0

34525516 T2 14 0

34527646 T3 22 1

34531019 T1 22 1

34536567 T1 24 0

34537198 T4 1 1

34538612 T3 3 0

34538900 T1 31 0

34539281 T1 4 0

34540626 T3 23 0

34542154 T3 21 4

34542332 T2 15 0

34542462 T1 26 0

34543182 T3 18 0

34546422 T1 24 0

34548400 T3 7 0

34550806 T2 20 0

34553252 T1 17 1

34554070 T1 16 0

34554756 T1 12 0

34556341 T3 26 0

34558980 T3 2 0

34559186 T1 8 0

34561327 T3 44 0

34562704 T1 14 0

34563688 T1 22 0

34564965 T2 33 0

34567830 T2 23 0

34570410 T3 22 6

34570704 T3 37 0

34572776 T1 12 0

34578531 T1 11 0

34586869 T1 32 0

34592944 T3 43 0

34593287 T3 13 3

34598053 T1 7 0

34602979 T1 20 0

34605065 T1 24 0

34607313 T1 6 0

34608469 T1 31 0

34612086 T3 1 0

34613731 T4 7 0

34614216 T3 5 0

34614406 T1 10 0

34615291 T1 26 0

34617696 T2 1 0

34618886 T3 17 0

34620209 T1 2 0

34620485 T1 1 0

34625529 T4 30 0

34625626 T1 18 0

34626129 T3 1 0

34627474 T1 16 0

34628989 T3 1 1

34629465 T1 13 0

34635077 T2 28 3

34635099 T1 31 0

34635995 T1 21 0

34636656 T2 23 0

34637258 T1 14 0

34637832 T1 10 0

34638653 T3 1 0

34639501 T2 9 1

34640816 T3 18 0

34643431 T4 1 1

34645776 T1 11 0

34645935 T1 18 0

34646542 T3 6 3

34647281 T1 2 0

34647567 T1 12 0

34648745 T3 12 0

34650782 T4 29 3

34651920 T3 16 0

34652304 T4 7 6

34654154 T3 13 0

34654159 T3 19 0

34655031 T3 6 0

34655364 T2 18 1

34659416 T3 9 0

34660113 T3 8 0

34661160 T2 19 0

34662197 T1 18 0

34663761 T3 18 0

34664277 T3 34 2

34664296 T2 22 10

34664307 T3 11 2

34665137 T2 19 0

34665719 T1 5 0

34665753 T3 19 2

34667107 T3 10 0

34667585 T4 10 4

34667888 T2 29 0

34668408 T3 22 0

34671005 T1 4 0

34673199 T1 18 1

34674476 T1 1 0

34676837 T2 3 0

34677199 T3 7 1

34681034 T3 7 1

34681228 T1 16 0

34682715 T1 12 0

34682928 T2 14 0

34683122 T2 28 0

34684051 T1 10 2

34684130 T1 13 0

34685186 T1 40 0

34685899 T1 14 0

34689694 T4 8 1

34689717 T3 16 0

34689789 T1 35 0

34692775 T4 4 0

34693990 T1 9 0

34694861 T1 2 0

34695395 T3 1 0

34695531 T2 23 0

34696503 T2 18 0

34696812 T4 4 1

38101694 T1 3 2

38108983 T3 5 1

38112094 T1 6 0

38112227 T1 1 0

38114049 T1 3 2

38121625 T1 20 0

38123938 T2 5 4

38125565 T1 15 0

38129680 T1 3 1

38130997 T1 4 0

38131186 T1 3 0

38131231 T1 3 0

38131424 T1 20 0

38131978 T1 8 0

38132801 T1 1 0

38133293 T3 3 0

38133459 T1 5 0

38133683 T2 45 9

38135463 T1 1 0

38136008 T4 26 10

38136739 T4 1 0

38137143 T3 1 0

38137296 T1 2 0

38138229 T2 4 0

38138803 T1 10 0

38139248 T1 8 0

38140554 T1 36 0

38141497 T3 16 0

38141846 T1 7 0

38143695 T1 28 0

38145274 T1 8 0

38147241 T1 9 0

38148149 T1 48 0

38149231 T2 22 0

38150030 T3 15 0

38150059 T1 26 0

38153852 T1 26 0

38154006 T2 1 0

38156991 T1 9 0

38160457 T3 6 0

38160690 T3 29 2

38161564 T3 12 0

38161955 T1 30 0

38162471 T3 1 0

38166562 T1 5 0

38171599 T3 19 7

38172981 T3 14 0

38177792 T3 11 0

38178340 T4 19 4

38180235 T1 8 0

38180312 T1 21 0

38181791 T3 33 3

38182253 T1 12 0

38184083 T1 19 0

38184483 T2 15 1

38185353 T1 19 0

38185442 T3 1 1

38186973 T1 17 0

38188741 T1 8 0

38190160 T2 25 0

38192561 T3 41 2

38192730 T1 6 0

38193397 T1 16 0

38195419 T1 4 1

38196758 T1 8 1

38197841 T1 16 0

38200242 T1 5 0

38202838 T1 10 0

38203030 T3 20 0

38206696 T1 30 0

38209420 T3 20 11

38211417 T1 17 0

38215414 T3 16 1

38215733 T3 1 0

38215926 T1 30 0

38216915 T1 19 0

38217895 T3 10 1

38218162 T3 23 3

38219882 T1 45 0

38220557 T3 5 0

38224314 T2 15 2

38226346 T1 16 1

38226583 T1 6 0

38226589 T2 9 0

38226711 T2 28 1

38226814 T3 15 6

39035167 T4 5 2

39037007 T4 12 9

39037833 T1 21 0

39038973 T2 3 0

39042069 T3 2 2

39062313 T2 1 1

39065274 T1 14 1

39067403 T1 2 0

39070723 T1 6 0

39074304 T4 4 0

39074825 T1 4 0

39076348 T1 27 0

40137465 T2 1 0

40148507 T3 2 1

40149645 T1 6 0

40150200 T3 1 0

40155015 T2 1 0

40155158 T1 11 0

40155795 T1 7 0

40156741 T3 6 0

40164582 T1 1 0

40167780 T2 27 0

40172800 T2 7 1

40173823 T1 6 0

40174646 T3 9 0

40175555 T1 21 0

40177137 T1 15 0

40193757 T1 2 0

40198572 T1 2 0

40201357 T2 15 0

40202570 T1 3 0

40206512 T2 5 0

40207587 T1 3 0

40208011 T1 2 0

40209120 T1 10 0

40211318 T1 2 0

40211462 T1 3 0

40214383 T1 8 0

40214524 T1 12 0

40219510 T2 10 0

40224618 T1 6 0

40225111 T2 24 0

40837676 T1 4 0

40839818 T3 16 1

40839911 T1 11 2

40841458 T2 18 0

40844216 T3 13 0

40853182 T2 9 0

40854312 T1 18 0

40857938 T4 7 3

40859610 T2 7 1

40859793 T1 22 0

40860368 T1 7 0

40860689 T2 13 0

40860988 T1 6 0

40864291 T3 18 1

40869419 T2 10 2

40870624 T1 22 0

40872051 T2 5 0

40872588 T3 6 0

40872789 T1 19 0

40874580 T1 11 0

40875422 T1 10 0

40880975 T1 9 0

40882268 T1 29 0

40882387 T2 4 0

40882388 T3 5 0

40882743 T3 17 0

40883942 T3 1 0

40884322 T1 1 0

40885678 T1 3 0

40886414 T1 23 1

40889362 T1 14 0

40891340 T2 21 0

40892430 T1 13 2

40893138 T3 17 0

40894170 T4 12 12

40896863 T3 7 0

40897171 T3 9 0

40898291 T1 23 0

40899259 T1 3 0

40902341 T3 12 1

40904542 T3 37 17

40905485 T1 6 0

40906143 T1 3 0

40906776 T1 8 0

40908763 T3 3 0

40910475 T1 8 0

40913300 T3 4 0

40913691 T3 6 0

40914321 T2 12 5

40915450 T1 24 0

40915453 T3 2 0

40918703 T1 10 0

40920327 T2 9 1

40921481 T1 8 2

40923102 T2 11 0

40925538 T2 7 2

40925818 T2 5 2

40926372 T3 7 0

40928051 T1 8 0

40928260 T3 3 3

40929003 T1 7 0

40929167 T1 11 0

40930162 T1 5 0

40931433 T3 7 0

40933093 T1 14 0

40934015 T2 16 2

40934922 T1 17 0

40937850 T1 13 0

40939060 T3 13 2

40939617 T4 2 2

40941273 T1 11 0

40942569 T1 14 0

40943930 T1 7 0

40951220 T1 17 0

40952788 T1 5 1

40954515 T3 14 4

40955903 T1 5 0

40956187 T4 4 0

40956757 T1 19 0

40959434 T1 10 0

40959863 T3 4 1

40961324 T1 16 0

40963064 T2 6 0

40963278 T3 2 1

40963336 T1 3 0

40963704 T3 2 0

40965369 T1 13 0

40966755 T2 3 1

40967124 T2 12 0

40968274 T1 8 0

40969152 T1 5 1

40971761 T3 14 4

40973418 T1 9 0

40973469 T4 16 0

40974500 T1 11 0

40978365 T3 12 0

40978427 T1 6 0

40983336 T3 7 3

40985601 T1 12 0

40986341 T3 31 0

40986578 T4 37 0

40991492 T1 11 0

40995575 T3 7 6

40995671 T1 20 0

41000178 T1 6 0

41000579 T3 30 10

41001033 T3 5 0

41001195 T1 1 0

41003006 T2 13 0

41003108 T2 6 0

41006559 T1 9 0

41007422 T3 31 0

41009115 T3 15 0

41009964 T2 3 0

41010176 T1 9 0

41010211 T1 1 0

41010240 T3 21 0

41011626 T1 9 0

41013047 T2 12 0

41014032 T1 9 1

41015807 T2 10 0

41017547 T1 7 1

41020204 T1 2 0

41021076 T3 6 0

41025158 T1 5 0

41028071 T1 4 0

41039621 T3 1 0

43002278 T2 39 0

43070132 T1 26 0

43070202 T1 36 0

43070262 T3 59 0

43070942 T1 14 0

43073461 T1 36 0

43074502 T1 3 0

43082559 T1 30 0

43082987 T1 34 0

43084051 T1 9 0

43084968 T3 25 0

43085633 T1 23 0

43090194 T2 16 1

43091984 T1 14 0

43095311 T1 2 0

43100654 T3 26 6

43101268 T1 2 0

43102332 T1 19 0

43103516 T1 9 0

43107491 T1 24 0

43107677 T2 20 1

43113853 T1 4 0

43114183 T1 15 0

43114780 T1 76 0

43116061 T1 64 0

43116339 T1 27 0

43116535 T3 6 2

43116962 T1 19 1

43117081 T1 7 1

43118549 T3 23 0

43118842 T4 18 1

43119138 T3 14 0

43119161 T3 4 0

43121581 T1 24 0

43122883 T1 49 0

43124907 T1 5 3

43128358 T1 9 1

43133593 T1 31 1

43133911 T3 17 8

43134384 T2 5 2

43134925 T3 27 0

43140439 T3 7 1

43143870 T3 13 2

43144453 T3 11 2

43146024 T1 30 0

43146047 T1 21 0

43146240 T2 44 2

43146295 T1 48 0

43146719 T2 29 0

43146904 T3 3 2

43146961 T3 16 0

43147488 T1 16 0

43147911 T1 47 0

43147977 T3 18 0

43148409 T1 15 0

43148595 T1 20 2

43151135 T3 14 0

43152998 T4 11 0

43156067 T1 3 0

43156329 T1 20 0

43158527 T3 3 3

43158881 T2 52 0

43160375 T2 23 0

43161226 T1 44 0

43163228 T1 19 5

43165452 T1 20 0

43165516 T2 9 0

43166002 T4 2 0

43166825 T3 48 5

43168255 T1 20 0

43170096 T3 26 0

43171720 T1 40 0

43174596 T1 17 4

43176196 T1 31 0

43178755 T1 49 0

43181886 T1 27 0

43186893 T1 14 0

43192058 T1 2 0

43195916 T1 11 0

43196809 T1 25 0

43197784 T1 30 0

43200277 T4 25 5

43202502 T1 19 0

43203866 T1 4 0

43210733 T2 31 0

43211397 T1 24 0

43211453 T1 18 0

43213298 T1 26 0

43216564 T1 40 0

43220328 T1 33 0

43221686 T3 23 0

43221774 T1 2 0

43224421 T3 2 2

43225127 T4 16 0

43225659 T1 25 0

43226224 T3 6 1

43226900 T4 20 0

43227146 T1 16 0

43228745 T1 27 0

43229083 T2 10 0

43229268 T3 1 0

43231678 T4 21 0

43231684 T1 40 0

44000370 T2 10 0

44001197 T1 27 0

44001200 T1 19 0

44003365 T1 20 0

44004502 T1 17 0

44005219 T1 5 0

44005849 T1 21 0

44008629 T1 21 0

44008646 T1 9 0

44009501 T1 10 0

44012309 T3 44 0

44014838 T1 21 0

44015196 T1 43 0

44015996 T2 34 0

44016110 T1 24 0

44022942 T1 30 0

44023844 T4 23 2

44024256 T1 27 0

44024541 T1 26 0

44024865 T3 27 0

44025584 T3 25 0

44027636 T2 24 0

44027857 T2 27 0

44029905 T1 39 0

44030260 T1 31 0

44030786 T1 5 0

44032586 T1 31 2

44033488 T3 36 5

44034157 T3 30 10

44034345 T1 25 1

44034437 T2 25 2

44037054 T4 12 0

46002731 T1 6 0

46007324 T1 16 0

46009912 T3 11 3

46010272 T2 13 0

46010433 T3 32 1

46010779 T3 4 0

46012036 T1 10 0

46012041 T1 15 0

46012242 T1 28 0

46014386 T1 5 0

46015044 T1 3 0

46019084 T1 7 0

46019436 T2 1 0

46023395 T1 2 0

46028779 T1 2 0

46029795 T3 3 0

46030259 T3 3 0

46030438 T2 2 0

46031397 T2 1 0

46034783 T3 11 0

46038935 T1 16 0

46040918 T1 4 1

46041462 T1 28 0

46043266 T2 1 0

46043509 T1 7 0

46045913 T3 36 0

46047135 T1 22 0

46049227 T3 13 0

46050120 T1 26 0

46050261 T1 20 0

46051226 T1 43 0

46058078 T1 21 0

46058128 T1 34 1

46058381 T1 25 0

46058415 T3 14 0

46058660 T1 2 0

46059121 T1 6 0

46059541 T3 1 0

46064186 T1 5 0

46065452 T1 2 0

46065638 T1 54 0

46065698 T2 13 0

46066251 T1 10 0

46066823 T1 1 0

46068079 T1 2 0

46075172 T3 3 0

46077906 T2 27 2

46078290 T2 14 0

46079923 T1 12 2

46080337 T3 2 1

46081908 T3 9 0

46088619 T1 3 0

46092573 T1 10 0

46092820 T1 12 0

46096864 T2 2 0

46098608 T1 5 0

46099590 T1 9 0

46100008 T2 17 0

46103245 T1 6 0

46105383 T1 9 0

46105392 T3 2 0

46107516 T3 24 0

46113660 T1 17 0

46114532 T1 10 0

46114794 T1 5 0

46115261 T3 17 0

46115822 T3 25 0

46117650 T2 14 3

46121238 T1 4 0

46121805 T3 15 0

46123773 T3 4 0

46125033 T2 20 4

46128507 T1 31 0

46132350 T1 14 0

46141232 T2 7 0

46142478 T1 33 0

46143796 T1 32 0

46148930 T2 6 0

46150347 T1 29 0

46150640 T1 2 0

46156172 T1 2 0

46157260 T1 2 0

46157584 T1 8 0

46160731 T3 21 4

46161380 T1 13 0

46162086 T1 8 0

46163337 T1 18 0

46164186 T3 11 0

46164639 T1 15 0

46164833 T3 3 0

46164868 T2 22 0

46168050 T1 7 0

46169396 T1 20 0

46170432 T2 2 0

46171583 T1 80 0

46172611 T1 9 0

46173900 T1 6 2

46175408 T3 21 3

46175715 T1 3 0

46176123 T2 18 0

46179258 T1 4 0

46179448 T2 1 0

46179544 T1 7 0

46179663 T1 4 0

46180240 T1 14 0

46181223 T3 20 11

46181527 T1 6 0

46181760 T2 19 1

46182711 T2 6 0

46183304 T1 7 0

46187005 T3 2 0

46187536 T3 2 1

46188203 T2 23 1

46189812 T3 12 0

46191085 T2 36 1

46192463 T1 1 0

46197167 T3 2 0

46198378 T3 7 0

46199516 T1 24 0

46200536 T1 11 0

46204168 T1 13 0

46204252 T3 25 5

46206197 T3 13 3

46207425 T3 15 0

46208915 T3 14 0

46208926 T1 22 0

46208994 T3 11 3

46209504 T3 5 0

46213926 T2 15 0

46215639 T1 4 0

46217614 T1 3 0

46217744 T1 6 0

46219015 T1 2 0

46224286 T1 16 0

46224366 T2 27 0

46224662 T1 8 0

47000411 T3 18 9

47002698 T1 48 0

47003069 T1 1 0

47005631 T1 11 0

47006384 T1 8 0

47010920 T1 7 0

47013181 T1 5 0

47017200 T2 19 0

47018752 T3 3 0

47019297 T3 5 0

47019604 T3 2 1

47019673 T1 4 0

47020857 T3 22 0

47020952 T3 2 0

47021257 T2 3 2

47021384 T1 8 1

47023231 T1 4 0

47026766 T1 15 0

47027238 T2 9 0

47027529 T2 11 0

47029728 T1 6 0

47030774 T1 17 0

47033191 T2 5 0

47041778 T3 21 0

47042690 T1 19 0

47042829 T1 13 0

47044997 T3 5 1

47046428 T1 6 1

47047172 T1 13 0

47048851 T1 3 0

47052346 T1 2 0

47054575 T1 2 1

47055783 T1 12 0

47055981 T1 8 0

47056803 T1 20 0

47059771 T3 19 0

47061322 T1 1 0

47067294 T3 3 0

47068045 T1 14 2

47069626 T2 2 0

47072414 T1 22 0

47077581 T3 2 0

47078796 T1 18 0

47080914 T1 2 0

47081505 T1 3 0

47082024 T3 31 0

47083106 T1 10 2

47089235 T1 17 0

47092122 T2 1 1

47092261 T3 3 0

47092579 T1 27 0

47096681 T1 3 0

47096988 T3 43 0

47097016 T1 7 0

47097167 T1 1 0

47098703 T1 12 0

47101646 T1 6 0

47101710 T3 1 0

47102259 T3 7 0

47102545 T1 22 0

47105042 T2 3 0

47105083 T1 13 0

47105121 T1 1 0

47108524 T1 10 0

47109115 T2 36 0

47112918 T2 24 0

47114574 T1 7 0

47117472 T2 43 0

47118318 T3 5 0

47118754 T1 17 0

47121954 T3 4 0

47122080 T3 1 0

47124301 T1 8 0

47124998 T1 2 0

47125496 T3 10 4

47125596 T2 1 0

47133282 T2 15 0

47145722 T1 25 0

47148100 T1 15 0

47151424 T2 2 0

47154082 T1 22 0

47159149 T1 5 0

47162445 T1 6 0

47164424 T1 17 1

47166439 T2 2 1

47166983 T1 12 1

47167102 T3 2 1

47169009 T2 19 3

47173371 T1 6 0

47174936 T1 2 0

47176601 T1 11 0

47178782 T1 12 1

47179482 T1 7 0

47180074 T3 3 2

47181506 T1 1 0

47183117 T2 14 3

47183414 T3 4 0

47185984 T2 1 0

47189487 T1 36 0

47189674 T1 3 0

47192071 T2 13 0

47192327 T1 19 0

47192453 T1 12 0

47197729 T2 35 0

47213358 T1 6 0

47216115 T3 5 1

47217550 T2 1 0

47221055 T1 15 0

47226241 T1 5 2

47227788 T4 1 1

48001238 T1 19 0

48011241 T1 29 0

48012047 T1 7 0

48013381 T1 12 0

48020191 T1 18 0

48020868 T2 10 0

48031639 T2 1 0

48033468 T1 8 0

48038292 T1 3 0

48047651 T4 28 1

48054793 T2 2 0

48055078 T3 1 1

48056485 T2 2 0

48061199 T3 15 7

48061717 T1 25 0

48062490 T1 4 0

48063175 T2 7 3

48067263 T1 24 0

48071432 T1 3 0

48073047 T3 10 0

48076548 T2 1 1

48077180 T1 12 0

48079297 T1 6 0

48096597 T2 8 2

48096652 T1 15 0

48097792 T1 6 0

48098010 T3 10 3

48098514 T1 4 0

48100060 T2 1 0

48100247 T2 24 0

48101413 T2 4 1

48105600 T1 12 0

48106537 T1 6 0

48107203 T1 2 0

48109008 T1 42 0

48114316 T2 18 0

48118122 T1 13 8

48118838 T3 12 0

48135087 T1 22 0

48139451 T2 9 4

48142322 T2 10 0

48143558 T2 30 0

48147152 T2 4 0

48148784 T1 6 0

48150112 T1 8 0

48150932 T2 2 0

48158501 T3 3 1

48160869 T1 11 0

49098410 T1 2 0

49099546 T3 6 0

49102835 T3 4 4

49103616 T2 4 0

49104131 T3 13 2

49105514 T1 16 0

49105570 T3 14 4

49107040 T1 24 0

49107746 T1 2 0

49113608 T3 7 0

49115113 T3 19 3

49117406 T2 6 0

49118878 T2 19 0

49119493 T1 8 0

49122542 T1 8 0

49123170 T1 38 0

49123535 T1 16 1

49125431 T1 14 0

49126310 T4 16 4

49132942 T2 20 0

49136114 T1 9 0

49140526 T1 31 0

49141646 T1 5 0

49141732 T3 18 0

49142343 T1 23 0

49143842 T3 38 24

49145505 T2 30 2

49146064 T1 15 1

49147408 T3 39 0

49155361 T2 22 2

49158801 T1 10 0

49159982 T1 14 0

49160480 T1 23 0

49163822 T2 33 4

49164439 T4 4 0

49165618 T1 25 0

49165710 T1 25 0

49166669 T1 19 1

49166797 T1 28 0

49167453 T1 9 3

49168074 T3 4 4

49168171 T1 18 0

49169653 T2 20 3

49170257 T2 26 0

49170939 T3 37 0

49173249 T1 17 0

49174274 T2 17 0

49174723 T1 7 3

49175540 T3 34 0

49176167 T2 2 0

49176233 T2 21 0

49179289 T1 11 0

49181542 T1 29 0

49183663 T1 26 0

49184603 T1 22 2

49190199 T3 15 0

49198834 T2 13 0

49201527 T3 29 0

49203525 T1 31 0

49203872 T1 11 0

49204569 T3 15 0

49206980 T2 5 2

49208112 T1 15 0

49212292 T2 18 0

49212507 T1 4 0

49213240 T1 13 0

49214601 T1 8 0

49215215 T2 7 1

49215312 T1 8 0

49215667 T1 30 0

49216767 T4 1 1

49218952 T1 20 0

49218972 T2 49 0

49220040 T1 25 2

49221075 T1 28 0

49222820 T1 6 0

49223633 T1 3 0

49226022 T3 25 6

50000737 T3 46 0

50000766 T3 13 0

50003079 T2 5 0

50003925 T2 3 0

50004560 T3 3 1

50006006 T1 16 0

50007141 T1 22 0

50007795 T1 7 0

50009775 T2 1 0

50010584 T1 11 0

50013571 T3 18 0

50020085 T1 3 0

50021150 T1 12 1

50021491 T1 13 0

50025402 T2 5 1

50028161 T1 32 0

50030043 T3 34 1

50030931 T2 8 0

50033808 T1 47 0

50033815 T2 26 0

50033888 T1 33 0

50034723 T2 2 1

50037328 T3 6 0

50037339 T1 24 0

50037794 T3 1 0

50038150 T2 20 0

50040108 T1 28 0

50043268 T1 18 0

50043415 T3 11 0

50043515 T2 22 1

50043524 T1 43 0

50044557 T3 6 0

50045497 T3 12 10

50049371 T1 6 0

50049375 T1 11 0

50049510 T1 9 3

50052430 T1 1 0

50058252 T1 5 0

50059778 T3 2 2

50062276 T2 4 0

50062770 T1 23 0

50066996 T1 4 0

50066999 T1 16 0

50067626 T4 19 3

50068751 T1 16 0

50071616 T3 13 0

50072176 T1 25 2

50073027 T1 10 0

50073142 T1 7 0

50074557 T1 13 0

50075211 T1 1 0

50076619 T3 10 1

50077252 T1 5 0

50080119 T2 23 2

50082290 T1 4 1

50082580 T3 24 0

50083616 T1 25 0

50086117 T3 50 0

50086340 T1 45 0

50088383 T3 36 0

50093174 T1 17 0

50095634 T1 61 1

50095962 T2 1 0

50096084 T1 3 2

50098037 T1 31 0

50098527 T3 40 0

50100106 T2 21 1

50101426 T1 5 0

50104832 T2 23 0

50105324 T2 16 0

50106796 T1 30 0

50108386 T2 3 0

50109292 T1 11 0

50109334 T2 9 0

50110528 T3 40 0

50110778 T3 22 0

50110983 T2 11 2

50111492 T1 2 0

50112559 T2 5 0

50113372 T1 4 1

50118481 T1 22 0

50118482 T3 46 5

50118483 T1 22 0

50124185 T3 39 0

50125728 T1 8 0

50127961 T1 43 0

50127978 T3 15 1

50128074 T1 8 0

50128371 T3 20 7

50129005 T3 22 16

50129751 T1 2 0

50129754 T1 2 1

50129890 T3 12 0

50136307 T3 22 16

50137490 T4 12 0

50138942 T1 16 13

50139362 T1 19 0

50141479 T1 29 0

50143226 T1 6 0

50144932 T1 13 5

50145258 T2 48 0

50145501 T2 2 0

50147597 T3 4 0

50147756 T3 6 0

50149217 T1 4 0

50150059 T3 3 0

50156812 T3 39 2

50157262 T1 22 0

50157578 T3 7 0

50158295 T1 5 0

50158827 T2 20 0

50161058 T1 12 0

50162575 T2 28 0

50172052 T2 3 0

50172205 T2 24 0

50173123 T2 4 0

50230765 T1 16 0

50231050 T3 8 0

51010017 T2 3 0

42507124 T2 7 1

54011697 T1 5 0

54012814 T2 1 0

54013127 T1 19 0

54013185 T2 2 1

54019210 T3 16 7

55191937 T1 3 0

55194012 T2 6 0

55195354 T1 25 0

55200345 T3 3 0

55200844 T2 36 0

55205361 T1 21 0

55205367 T1 13 0

55214880 T2 12 1

55221350 T1 2 0

55224906 T3 4 0

55225500 T3 16 4

55231604 T1 8 0

55232491 T3 24 0

55232530 T3 2 2

55234693 T2 8 0

56503542 T1 39 0

56513797 T3 13 0

56520233 T1 9 0

56521884 T3 11 2

56523281 T3 9 6

56523305 T4 10 0

56526189 T1 29 0

56527532 T3 8 0

56529300 T3 5 1

56533478 T1 9 0

56536436 T1 11 0

56557958 T1 6 0

56568036 T3 3 0

56574604 T1 37 0

56585769 T2 14 0

56589305 T1 45 0

56593852 T4 4 3

56605406 T2 6 0

56608292 T1 1 0

56624740 T1 41 0

56629545 T1 27 0

57714649 T1 27 0

57718455 T2 26 0

57721830 T1 8 0

57723387 T1 24 0

57730921 T1 9 0

57731661 T2 14 0

57734827 T1 11 0

57736106 T1 35 0

57737071 T3 4 2

57737906 T1 5 0

57739154 T3 3 0

57739444 T3 6 0

57782262 T4 1 0

57817825 T4 8 0

57825007 T2 4 3

57826111 T1 21 0

57837516 T1 7 0

57843485 T1 33 0

57873616 T1 12 0

57876302 T3 4 0

59177508 T3 1 0

59182707 T1 15 2

59186725 T1 2 0

59191403 T1 28 0

59199477 T1 33 0

59206092 T1 11 0

59208924 T3 20 0

59208960 T3 23 0

59218901 T1 5 0

59225640 T2 11 0

59230026 T1 4 0

59231507 T4 20 9

59232076 T1 7 0

59235211 T1 16 0

59245334 T1 24 0

59251597 T3 5 0

59255886 T2 17 2

59269479 T1 15 1

59269499 T3 32 16

59270401 T2 5 0

59272753 T1 22 0

59278742 T1 25 0

59278795 T1 16 0

59278996 T1 15 0

59300022 T1 1 0

59335106 T1 35 3

60437127 T3 13 0

60449553 T2 8 0

60453309 T2 13 0

60459072 T1 15 1

60462767 T3 23 2

60462786 T2 4 1

60474813 T1 24 3

60490192 T1 7 0

60491759 T1 2 0

60514301 T1 20 0

60517260 T1 13 0

60522669 T2 15 1

60551295 T1 34 0

60551449 T1 19 0

60555569 T1 1 0

60557590 T2 7 1

60558742 T1 19 0

60558787 T3 1 1

60562662 T3 8 0

60562933 T1 23 1

60577628 T2 8 0

60582098 T1 37 0

60582301 T1 44 2

60583883 T3 5 1

60584403 T1 2 0

60587207 T3 50 9

60587761 T1 27 0

60591652 T1 10 0

60592529 T3 2 1

60592822 T1 27 2

60593315 T1 31 0

60593417 T1 18 0

60596284 T3 30 4

60597183 T3 10 9

60598083 T1 8 1

60598783 T1 21 0

60599024 T1 5 0

60599027 T2 9 1

60600259 T2 7 2

60601964 T1 3 0

60614362 T1 41 0

60615646 T1 6 0

60617572 T4 11 1

60617801 T2 7 0

60618321 T1 16 0

62054642 T1 6 0

63163529 T1 18 0

63173073 T1 15 0

63182098 T2 14 0

63212320 T2 20 0

63214630 T1 2 0

63218220 T1 5 0

63227306 T3 9 0

63233718 T1 28 0

63233849 T1 8 0

63236999 T2 42 1

63246866 T1 16 0

63247122 T1 15 0

63248899 T3 6 0

63254724 T3 18 1

63262906 T1 8 1

63266954 T1 11 0

63271515 T1 21 0

63277410 T1 22 1

63279904 T2 34 1

63287637 T1 11 0

63291732 T1 16 5

63296340 T4 13 4

63303035 T1 40 0

63305381 T3 8 2

63312788 T3 16 0

63316252 T1 42 0

63316467 T3 7 0

63318053 T1 3 0

63321647 T1 14 0

63323141 T1 20 0

63324918 T1 8 0

63326606 T3 7 2

63327753 T3 27 0

63327992 T1 19 0

63328576 T3 15 0

63331309 T3 16 0

63331591 T2 4 1

63336659 T1 9 0

63336980 T1 11 0

63348891 T1 16 2

63349096 T1 9 0

63353311 T1 21 0

63357852 T1 5 0

63357858 T1 21 0

63358844 T4 7 1

63359610 T1 5 2

63359893 T3 12 3

63360607 T2 20 0

63363547 T2 1 1

63363850 T1 14 0

63364537 T3 4 0

63366582 T1 13 0

63371898 T3 8 0

63382741 T1 23 0

63389506 T2 2 1

63390812 T3 19 0

63393851 T3 11 4

63393936 T1 4 0

63407182 T1 26 0

63408427 T1 27 0

63416964 T2 23 0

63417136 T1 20 0

64508385 T1 24 0

64511689 T3 18 1

64513423 T1 20 0

64513788 T3 2 2

64514813 T1 30 0

64516895 T3 9 0

64517259 T3 16 0

64517675 T3 3 0

64524633 T3 10 0

64525587 T2 10 0

64525761 T3 1 0

64526988 T3 8 0

64527006 T1 10 0

64529067 T1 12 0

64530341 T1 12 0

64530432 T3 12 3

64535054 T1 4 0

64536183 T2 16 0

64541228 T1 12 0

64541620 T3 6 3

64541904 T1 4 0

64544147 T1 15 2

64545126 T3 3 1

64545156 T3 2 1

64550172 T2 10 0

64552716 T2 29 2

64553469 T1 7 0

64554869 T3 6 4

64556755 T2 16 0

64560253 T1 4 0

64560258 T1 1 0

64563747 T1 5 0

64564723 T3 3 0

64569692 T1 24 2

64570150 T1 19 0

64572498 T1 3 0

64572808 T4 9 0

64572927 T2 5 0

64578081 T2 5 0

64583147 T3 6 0

64584027 T1 2 0

64585969 T1 20 0

64588779 T2 43 0

64589290 T1 21 0

64589326 T2 19 0

64589539 T1 21 0

64590629 T2 16 3

64594179 T1 36 0

64594375 T1 3 0

64594859 T2 10 4

64595194 T1 12 0

64598287 T1 42 15

64599521 T1 4 0

64600052 T3 26 1

64601131 T1 38 0

64603338 T3 1 1

64604359 T1 2 0

64605272 T1 6 0

64606561 T3 5 0

64607337 T1 15 0

64608327 T1 26 0

64610590 T3 10 3

64613762 T1 53 0

64614402 T3 4 2

64617002 T2 3 2

64621257 T3 2 2

64623935 T1 11 0

64624080 T2 5 0

64624799 T3 61 0

64625911 T1 25 0

64626557 T1 18 0

64627342 T1 14 0

64627509 T3 19 1

64628002 T2 3 0

64628269 T2 12 0

64628425 T1 18 0

64628606 T2 4 0

64633692 T3 24 1

64634029 T1 14 0

64634052 T1 3 0

64634053 T3 9 9

64637469 T1 13 0

64643195 T3 11 0

64645969 T4 8 0

64646308 T1 2 0

64646561 T1 7 0

64647685 T4 27 22

64648516 T1 13 0

64648738 T1 51 0

64649021 T3 7 1

64649806 T1 21 0

64654604 T1 23 0

64656986 T3 22 1

64657002 T1 2 0

64658018 T1 16 0

64658711 T1 45 0

64658719 T3 16 0

64659841 T1 18 0

64663045 T1 17 0

64664789 T1 16 0

64671389 T1 1 0

64671810 T2 21 11

64672863 T3 2 0

64672946 T1 8 1

64676587 T1 2 0

64676724 T1 6 0

64677531 T1 16 0

64677997 T1 2 0

64678987 T3 34 0

64679780 T1 20 0

64680187 T3 2 0

64682909 T3 33 0

64684890 T1 22 0

64685920 T1 17 0

64687663 T2 24 1

64688586 T3 16 3

64691166 T3 4 1

64694136 T1 12 0

64694979 T1 14 0

64697129 T3 40 0

64700146 T1 25 0

64703408 T1 3 0

64707978 T3 10 0

64716765 T1 14 0

64717267 T1 19 0

64717289 T3 15 2

64717631 T1 28 0

64718553 T2 16 0

64721600 T1 25 0

64722089 T1 13 0

64723648 T1 25 0

64726158 T1 4 0

64728047 T2 31 5

64729800 T1 4 3

64730824 T1 1 0

64735361 T2 24 0

64740528 T1 8 0

64741885 T1 44 0

64752882 T1 5 0

64760861 T1 19 0

64767688 T4 2 1

74199141 T4 6 3

74202237 T3 5 2

74204043 T3 11 0

74204917 T1 7 0

74209522 T1 3 0

74210362 T2 6 0

74210471 T2 16 0

74213333 T3 4 2

75006283 T2 20 0

75008308 T2 48 0

75009336 T1 10 0

75010352 T1 6 0

75010669 T1 9 0

75010671 T1 11 0

75011544 T3 3 0

75011764 T3 8 2

75013817 T1 13 0

75016130 T3 14 0

75017172 T3 12 4

75018366 T1 4 0

75022066 T2 1 0

75022421 T1 4 0

75022445 T1 17 0

75024532 T1 8 0

75024757 T3 1 0

75030727 T3 1 1

75035474 T1 6 0

75041046 T3 9 3

75042993 T1 24 0

75047535 T4 2 0

75048015 T1 11 0

75048965 T3 21 0

75049021 T2 1 1

75052984 T3 22 0

75055476 T2 7 0

75055498 T3 10 2

75055640 T3 5 1

75056123 T2 7 0

75059628 T1 17 0

75059741 T1 8 0

75061542 T4 4 0

75061807 T4 1 0

75063027 T1 2 0

75067320 T1 11 0

75067571 T3 5 2

75074734 T1 14 1

75075116 T1 5 1

75075180 T1 2 0

75075320 T1 3 0

75077491 T1 13 0

75079777 T1 3 0

75079899 T2 7 0

75082198 T3 3 3

75084945 T3 2 0

75085798 T2 16 0

75087055 T1 6 4

75088216 T1 10 0

75089826 T1 33 0

75092702 T2 7 0

75093496 T1 8 4

75096445 T1 6 0

75099087 T1 6 0

75101048 T4 25 0

75101053 T1 3 0

75102110 T4 3 0

75102943 T1 33 0

75103481 T1 27 0

75103672 T1 9 0

75113764 T1 3 0

75113859 T3 13 0

75116146 T4 17 0

75116522 T1 8 0

75117747 T3 16 0

75117761 T3 12 4

75118205 T3 9 0

75125851 T1 18 0

75128442 T3 25 3

75128811 T1 7 0

75132521 T3 9 0

75132881 T1 5 0

75134187 T3 6 4

75135028 T4 39 1

75137671 T2 24 0

75140223 T3 1 1

75140284 T2 8 0

75140364 T2 16 0

75140761 T4 9 0

75147262 T1 31 0

75149469 T3 3 2

75149544 T1 2 0

75149930 T1 13 0

75151888 T1 3 0

75154634 T3 19 0

75156004 T3 7 0

75163847 T1 7 0

78070961 T3 13 1

78071656 T1 7 5

78076926 T3 4 0

78079606 T1 12 0

78080797 T2 4 0

78083995 T3 16 2

78085531 T1 10 0

78088629 T1 6 0

78088633 T3 9 1

78090904 T1 16 0

78090906 T4 2 0

78092712 T1 16 0

78093225 T2 6 0

78096488 T3 13 9

78099790 T3 1 0

78100078 T3 2 1

78100081 T1 8 0

78102887 T1 9 0

78103869 T4 1 1

78106326 T1 13 6

78112034 T2 13 2

78114194 T1 7 0

78114413 T3 35 10

78118005 T1 4 1

78119935 T3 2 2

78120069 T4 3 0

78124838 T1 7 0

78125036 T1 19 0

78125391 T1 6 0

78129106 T2 13 0

78130938 T3 1 1

78131882 T1 10 0

78135416 T2 7 7

78137590 T1 9 2

78138145 T1 2 0

78142037 T1 20 0

79022372 T3 10 3

79029831 T1 19 2

79034638 T1 5 0

79034802 T1 14 0

79035565 T2 1 1

79041201 T1 2 0

79041207 T1 2 0

79041228 T2 5 0

79041749 T2 5 0

79042433 T1 7 0

79103011 T1 3 0

79104139 T2 12 0

79109399 T1 3 0

79116435 T1 7 4

79118886 T3 17 0

79161588 T1 19 0

80032828 T1 2 0

80038656 T1 9 0

80038946 T1 6 0

80045099 T1 6 0

80049266 T2 21 0

80054051 T1 7 0

80055888 T3 38 0

80095389 T2 4 1

80102231 T1 13 0

80103497 T3 1 0

80120944 T3 1 0

89611119 T1 20 0

89636391 T1 17 0

89641767 T4 2 2

89641907 T1 4 0

89642152 T1 10 0

89645935 T1 3 2

89648359 T2 24 0

89652622 T3 7 0

89653981 T1 12 1

89656048 T3 4 0

89656649 T2 5 0

89660106 T1 10 0

89661657 T1 11 0

89661734 T1 17 0

89663095 T1 4 0

89664647 T1 3 2

89665208 T3 8 1

89667655 T3 16 0

89669856 T2 3 0

89670084 T3 3 0

89670817 T1 1 0

89671871 T3 8 0

89673603 T4 6 4

89674200 T3 8 0

89674411 T4 9 0

89674436 T1 19 0

89674454 T3 2 2

89676417 T3 9 1

89677534 T3 1 0

89677844 T1 2 0

89678413 T1 15 2

89678725 T1 9 0

89678751 T3 20 2

89678801 T2 4 0

89680453 T1 1 0

89681734 T1 10 0

89683864 T3 15 0

89684502 T1 22 0

89686947 T1 8 0

89687424 T1 1 0

89687575 T1 1 0

89687955 T3 6 0

89688866 T3 7 1

89689306 T4 14 2

89692647 T1 10 0

89692929 T2 2 0

89693835 T3 16 1

89696593 T1 10 1

89696731 T2 4 0

89702957 T2 9 0

89704760 T1 10 0

89705622 T1 18 0

89705643 T1 19 0

89706144 T3 3 2

89706351 T3 9 0

89706434 T1 5 0

89706712 T4 6 4

89709059 T3 14 11

89709587 T2 23 0

89712619 T1 12 0

89714470 T1 5 0

89714650 T3 12 0

89718340 T3 1 0

89722735 T1 7 0

89723993 T1 2 0

89724055 T4 14 1

89725208 T3 4 0

89726053 T1 4 0

89729129 T1 9 0

89729319 T3 13 0

89729610 T1 13 0

89732168 T2 2 0

89732509 T1 25 0

89733562 T1 7 0

89734489 T2 11 3

90755447 T1 2 0

90756000 T2 26 0

90756054 T1 2 0

90756657 T1 12 1

90757146 T1 11 0

90757817 T1 4 0

90761031 T1 30 0

90762371 T1 7 0

90762392 T1 6 0

90763465 T3 10 0

90765060 T1 1 0

90765674 T1 5 0

90766209 T3 4 0

90766805 T1 35 0

90768029 T1 9 0

90769106 T1 13 0

90770200 T1 8 0

90771120 T2 1 0

90771402 T1 7 1

90772233 T1 7 0

90772520 T3 4 1

90773086 T1 9 0

90776887 T3 5 1

90778251 T1 11 0

90779572 T1 5 0

90780163 T3 22 22

90780735 T1 22 0

90780815 T1 27 0

90781302 T2 7 0

90782671 T1 37 0

90782710 T2 22 6

90783783 T1 12 0

90784069 T3 29 0

90784676 T3 5 0

90785871 T1 12 1

90787795 T1 7 2

90788075 T1 9 0

90788228 T4 1 0

90789651 T1 2 0

90789847 T1 11 0

90792064 T3 2 0

90792093 T1 5 0

90792598 T3 8 0

90795381 T2 6 0

90796458 T1 5 0

90797873 T3 19 0

90798694 T3 12 0

90800199 T3 4 3

90801006 T1 6 0

90803622 T1 4 0

90804888 T1 13 0

90807652 T3 1 0

90807746 T2 5 0

90808603 T3 2 0

90809890 T3 13 0

90811463 T3 1 0

90812034 T1 6 0

90813181 T3 4 0

90814019 T3 2 0

90814602 T1 21 0

90814871 T2 16 1

90815022 T1 11 0

90815712 T1 14 0

90815884 T3 2 1

90815999 T2 1 0

90816617 T4 1 1

90818802 T1 5 0

90819162 T3 6 1

90821722 T2 7 0

90822165 T3 9 0

90822201 T3 42 0

90823333 T1 17 0

90824626 T2 2 0

90826052 T3 16 0

90827451 T1 8 0

90827487 T2 1 0

90829748 T3 5 0

90829900 T1 1 0

90833960 T2 36 0

90834691 T3 3 0

90835983 T3 3 1

90838914 T3 4 0

90838972 T3 2 0

90839010 T1 1 0

90839156 T1 9 0

90840884 T3 3 3

90842564 T2 24 0

90843064 T3 8 0

90843858 T1 30 0

90844140 T1 9 0

90847819 T1 6 0

90850689 T4 8 3

90850972 T1 5 4

90851246 T1 9 0

90851665 T3 14 2

90852347 T2 11 0

90853235 T2 3 0

90855563 T3 1 0

90860087 T2 15 1

90860814 T3 12 1

90861725 T2 11 4

90863818 T3 12 0

90866222 T2 9 0

90867069 T1 14 0

90871717 T3 3 2

90872091 T1 6 0

90873041 T2 16 0

90873650 T3 33 2

90874531 T1 22 0

90877851 T1 4 0

90879117 T3 30 0

90879254 T1 4 0

90883976 T1 8 0

90884492 T1 1 0

90885693 T1 2 2

90886671 T2 8 0

90888116 T1 32 0

90888125 T1 13 1

90889209 T1 24 0

90889273 T3 35 0

90892358 T1 10 0

90892401 T3 9 0

90892425 T3 2 0

90892741 T1 7 0

90894039 T3 9 1

90895550 T1 3 0

90897936 T3 5 2

90900421 T2 52 0

90905880 T2 5 0

90906172 T2 18 0

90906203 T2 11 0

90908355 T2 10 0

90910166 T3 8 7

90910490 T1 6 0

90910927 T1 2 2

90912322 T1 11 0

90912752 T3 8 0

90912797 T1 13 0

90913394 T1 10 0

90914805 T2 9 0

90915600 T1 3 0

90915737 T1 7 0

90916367 T1 14 0

90917949 T1 5 0

90920985 T1 3 1

90924556 T1 13 0

90925892 T1 39 0

90932374 T3 8 0

90933339 T2 8 0

90936067 T1 9 6

90936731 T2 2 0

90937507 T3 9 0

90939019 T3 5 5

90939084 T3 15 11

90939216 T3 4 0

90940805 T2 8 0

90940865 T3 6 1

90941438 T1 10 0

90943079 T2 16 0

90944090 T1 21 0

90945330 T1 12 0

90946217 T2 1 1

90947935 T3 5 0

90950007 T1 8 0

90950335 T1 5 0

90950491 T1 9 0

90951377 T1 4 0

90951676 T2 5 0

90954381 T3 13 0

90954695 T1 5 0

90954906 T1 2 0

90955022 T1 3 0

90955154 T1 8 0

90956182 T1 14 0

90956196 T1 34 0

90956976 T3 7 0

90958273 T1 2 0

90960482 T2 1 0

90961463 T3 11 4

90961575 T1 21 2

90961687 T1 17 0

90962807 T2 11 0

90963854 T3 14 0

90965838 T2 1 0

90966792 T3 6 2

90966850 T1 3 0

90967794 T3 12 0

90968124 T3 8 4

90970012 T2 23 0

90970943 T4 29 0

90971428 T2 45 0

90972065 T1 17 0

90972499 T2 11 1

90972745 T3 5 0

90973127 T1 13 0

90973737 T1 11 0

90975154 T1 9 0

90975798 T1 11 0

90978011 T4 2 2

90978029 T1 3 0

90978761 T1 8 0

90981026 T3 11 1

90983135 T3 6 5

90985272 T1 11 0

92000112 T2 39 3

92001218 T4 2 0

92005030 T3 6 0

92006052 T1 11 1

92007485 T3 18 0

92012515 T3 14 0

92012659 T1 3 0

92014530 T1 9 0

92016844 T2 10 1

92016875 T3 13 0

92024893 T1 25 0

92025202 T4 5 0

92028529 T1 5 0

92030206 T1 10 0

92060952 T1 5 0

92064250 T1 13 0

92082733 T2 4 0

93359520 T1 16 0

97017718 T1 10 0

97020919 T1 2 0

97023733 T1 3 0

97024799 T1 30 0

97027568 T3 16 0

97029218 T1 27 0

97030132 T3 17 0

97033098 T3 2 0

97033846 T2 11 0

97035836 T2 8 1

97035989 T2 8 0

97038759 T2 12 0

97039985 T3 8 2

97040246 T1 1 0

97044163 T1 17 0

97045197 T1 1 0

97045342 T4 20 0

97046524 T3 3 0

97048288 T4 8 0

97050167 T1 14 0

97053652 T3 38 0

97053931 T1 14 1

97055561 T3 5 0

97055661 T2 8 0

97056807 T2 4 0

97056938 T1 1 0

97058289 T1 2 0

97060973 T3 24 1

97063729 T1 2 0

97065526 T1 9 0

97065566 T1 8 0

97066001 T3 26 8

97066077 T2 1 0

97070786 T3 35 0

97071355 T3 30 7

97071507 T1 5 0

97071951 T1 8 0

97072068 T2 9 1

97074358 T3 5 0

97077909 T2 8 0

97079841 T3 16 2

97082438 T4 5 1

97086107 T2 19 0

97086111 T1 17 0

97089092 T4 13 0

97090047 T1 4 0

97090233 T2 12 0

97091980 T1 2 0

97094877 T3 3 1

97095328 T3 4 0

97095966 T2 11 0

97097446 T2 2 2

97102570 T3 3 1

97110978 T1 5 1

97114621 T1 29 0

97119215 T2 20 0

97119447 T3 12 1

97121033 T1 28 0

97121061 T1 24 0

97121092 T2 6 0

97121323 T1 4 0

97122061 T1 4 0

97123314 T2 8 0

97132051 T1 6 0

97134507 T1 3 0

97138839 T1 8 6

97139866 T1 20 0

97141398 T1 5 0

97148730 T2 12 0

97149911 T2 14 0

97153349 T1 5 0

97154394 T1 2 0

97157112 T2 9 4

97159842 T2 21 0

97161361 T2 23 8

97163955 T3 7 1

97164288 T1 13 0

97165125 T1 7 0

97171798 T1 9 1

97172863 T3 15 0

97174543 T4 10 6

97175717 T3 21 4

97176543 T1 10 0

97177107 T1 4 0

97177570 T2 2 0

97177764 T1 4 0

97180242 T1 9 1

97183228 T3 5 0

97188024 T1 6 0

97189725 T3 21 0

97755383 T1 2 0

97755830 T4 13 0

97781090 T3 12 0

97797737 T2 8 0

97856806 T1 12 0

97863870 T3 25 2
